# South African nose flies (Diptera, Calliphoridae, Rhiniinae): taxonomy, diversity, distribution and biology

**DOI:** 10.3897/BDJ.11.e72764

**Published:** 2023-01-13

**Authors:** Arianna Thomas-Cabianca, Martin H. Villet, Anabel Martínez-Sánchez, Santos Rojo

**Affiliations:** 1 Department of Environmental Sciences and Natural Resources, University of Alicante, E-03080, Alicante, Spain Department of Environmental Sciences and Natural Resources, University of Alicante, E-03080 Alicante Spain; 2 Rhodes University, Southern African Forensic Entomology Research Laboratory, Grahamstown, South Africa Rhodes University, Southern African Forensic Entomology Research Laboratory Grahamstown South Africa

**Keywords:** biology, checklist, distribution maps, new combinations, new synonym, Oestroidea, Rhiniinae

## Abstract

**Background:**

Rhiniinae (Diptera, Calliphoridae) is a taxon of nearly 400 known species, many of them termitophilous. Approximatelly 160 valid species in 16 genera are Afrotropical, with over 60 of them occurring in South Africa. The taxonomy of this group is outdated, as most studies of the South African taxa were conducted 40 to 70 years ago (mostly by Salvador Peris and Fritz Zumpt). Published information on their biology and ecology is also scarce.

**New information:**

An annotated checklist of 73 species of Rhiniinae for South Africa was developed, based on the holdings of sixteen entomological collections in Africa, Europe and North America. Over 3,700 specimens were examined, revealing nine new species records for South Africa (*Cosminaundulata* Malloch, 1926, *Isomyiacuthbertsoni* (Curran, 1938), *Rhyncomyabotswana* Zumpt, 1974, *R.tristis* Séguy, 1933, *Stomorhinaapta* Curran, 1931, *S.malobana* (Lehrer, 2007), *Thoraciteskirkspriggsi* Kurahashi, 2001, *Th.sarcophagoides* Kurahashi, 2001 and *Trichoberialanata* (Villeneuve, 1920)). We propose one new combination *Eurhyncomyiametzi* (Zumpt, 1981) **comb. nov.** (= *Rhyncomyametzi* Zumpt, 1981)). Additionally, evidence is presented to remove *Rhyncomyaviduella* Villeneuve, 1927 **stat. rev.** from synonymy with *Rhyncomyacassotis* (Walker, 1849). Relevant novel biological and seasonality information, historical occurrence maps and high-definition photographs for each species are compiled.

## Introduction

Rhiniines, recently re-established as a subfamily of Calliphoridae, comprise around 400 recognised species within 30-39 genera (Kutty et al. 2010, Lehrer 2011, Pape et al. 2011, Singh and Wells 2013, Marinho et al. 2016, Cerretti et al. 2017, Cerretti et al. 2019, Kutty et al. 2019, Yan et al. 2021). The subfamily is distributed mainly in the Afrotropical Region, extending into the Palaearctic and the Oriental and Australasian Regions. With approximately 190 described species, the Afrotropical Region contains the largest known diversity of Rhiniinae. Approximately 60 of these species occur in South Africa (Zumpt 1958, Pont 1980, Lehrer 2011, Pape et al. 2011), which harbours the most diverse and distinctive Diptera fauna in the region (Kirk-Spriggs and Muller 2017). This is not surprising considering that South Africa is an endemism and diversity hot-spot (Janion-Scheepers et al. 2016). Such diversity is related to the ecological, topographic and climatic complexity of the country, expressed in 35 bioregions contained in nine biomes, many of which are unique to South Africa (Mucina and Rutherford 2006).

Rhiniinae are represented in South Africa by 12 genera belonging to two tribes, Rhiniini and Cosminini. Rhiniini include the genera *Rhinia* Robineau-Desvoidy, 1830, *Rhyncomya* Robineau-Desvoidy, 1830, *Isomyia* Walker, 1859, *Stomorhina* Rondani, 1861, and *Fainia* Zumpt, 1958, while Cosminini include *Cosmina* Robineau-Desvoidy, 1830, *Stegosoma* Loew, 1863, *Thoracites* Brauer & Bergenstamm, 1891, *Eurhyncomyia* Malloch, 1926, *Trichoberia* Townsend, 1933, *Zumba* Peris, 1951 and *Pseudorhyncomyia* Peris, 1952 (Zumpt 1958, Pont 1980, Kurahashi and Kirk-Spriggs 2006, Lehrer 2011, Thomas-Cabianca et al. 2021).

The latest reviews of the taxonomy and diversity of South African Rhiniinae were carried out between the 1950s and 1980s by Dr. S.V. Peris and Dr. F.K.E. Zumpt, who made excellent contributions for the entire African continent. They generated keys for most Afrotropical genera and species, discussed known species and described 13 new species from South Africa (Peris 1951, Peris 1952a, Peris 1952b, Peris 1956, Zumpt 1957, Zumpt 1958, Peris 1960, Zumpt and Stimie 1965, Zumpt 1967, Zumpt and Argo 1978, Zumpt 1981). After a significant lull in the taxonomic study of the group, Kurahashi and Kirk-Spriggs (2006) reported new records for South Africa and Lehrer described three new species (Lehrer 2007a, Lehrer 2009, Lehrer 2010). Species descriptions and synonyms from the 1960s onwards, except for *Thoracites* (Kurahashi 2001) and *Fainia* genera (Thomas-Cabianca et al. 2021) and an Afrotropical genera identification key (Rognes, in press), have not been incorporated into identification keys and no formal revision of all of the taxonomic opinions has been conducted.

The biology (e.g. immature stages and habits) of most species of Rhiniinae remain poorly known (Cuthbertson 1933, Cuthbertson 1934, Peris 1952a, Zumpt 1958, Kurahashi and Kirk-Spriggs 2006, Arce et al. 2019). In general, it is known that most Rhiniines are found in non-disturbed environments. Adults of several species of *Isomyia*, *Rhyncomya*, *Thoracites* and *Cosmina* are flower visitors and some species of *Stegosoma*, *Rhinia* and *Stomorhina* show exclusive associations with termites and ants, frequently being found around their nests. Females have been observed laying eggs in recently-disturbed soil that is rich in humus, in elephant dung, around cow dung, in aardvark (African antbear—*Orycteropusafer* (Pallas)) faeces and burrows and in grasshopper oothecas (Cuthbertson 1933, Cuthbertson 1934, Peris 1952a, Zumpt 1958, Kurahashi and Kirk-Spriggs 2006). This information remains to be reviewed.

In order to update and augment the knowledge of the Rhiniinae of South Africa, we assembled the first checklist of species for the country. We also provide information on the current taxonomic and nomenclatural status of the species, occurrence maps, a thorough compilation of the known and novel biological information and high definition habitus photographs for each species.

## Materials and methods

This study is based on the examination of about 3,000 specimens from South Africa and 700 specimens from other countries in the Afrotropical Region housed in sixteen entomological collections in ten countries. Acronyms used in the text for the museums and institutions are as follows: **AMGS** (Department of Entomology, Albany Museum, Grahamstown, South Africa); **BMSA** (Department of Entomology, National Museum, Bloemfontein, South Africa); **CEUA** (Entomological Collection, University of Alicante, Alicante, Spain); **DMSA** (Durban Natural Science Museum, Durban, South Africa); **MNHN** (Muséum national d’Histoire Naturelle, Paris, France); **MZSUR** (Zoology Museum, La Sapienza University of Rome, Rome, Italy); **NHMUK** (Natural History Museum, London, United Kingdom); **NMSA** (KwaZulu-Natal Museum, Pietermaritzburg, South Africa); **RMCA** (Musée Royal de l’Afrique Centrale, Tervuren, Belgium); **SAMC** (Iziko South African Museum, Cape Town, South Africa); **SANC** (South African National Collection of Insects, Agricultural Research Council, Pretoria, South Africa); **SMNHTAU** (Steinhardt Museum of Natural History, Tel Aviv University, Tel Aviv, Israel); **UCME** (Entomological Museum, Biology School, Complutense University of Madrid, Madrid, Spain); **USNM-SM** (United States National Museum, Smithsonian Institution, Washington D.C., United States of America); **ZMHB** (Museum für Naturkunde, Leibniz-Institut für Evolutions- und Biodiversitätsforschung, Berlin, Germany); **ZMUC** (Natural History Museum of Denmark, Copenhagen, Denmark).

Pinned specimens or specimens preserved in ethanol were examined using stereoscopic microscopes with ocular micrometres (Leica M80 or Leica MZ95). Identifications were based on the works of Peris (1952a), Zumpt (1962a), Zumpt (1956), Zumpt (1958), Zumpt (1962b), Zumpt (1965), Zumpt (1972a), Zumpt (1972b), Zumpt (1974), Zumpt (1981), Peris (1992), Kurahashi (2001), Kurahashi and Kirk-Spriggs (2006), Lehrer (2007b), Rognes (2012), Rognes (2013) and Thomas-Cabianca et al. (2021). Terminalia of males were prepared following the dissection methods of Rognes (2009) and Cerretti and Pape (2012), stored in small plastic microvials filled with glycerine and pinned together with the specimen. Comparisons with original descriptions of species and examinations of type specimens (when possible) and reference collections were also conducted. Zumpt’s redescriptions, illustrations of male terminalia and identified specimens were used for corroboration in cases where an original description did not provide sufficient information for identification and the type material was unavailable.

For each genus and species studied, the following sections are provided: generic: synonyms and type species; species: specific synonyms (considered from Pont 1980 and the Systema Dipterorum (Evenhuis and Pape 2022)), type locality (only for type-series specimens examined the repository information is provided in a remarks section), distribution (new records indicated by an asterisk "*"), occurrence map, notes (divided into preferred environment, recorded elevations, seasonality, behaviour and ecology, collecting methods, life cycle and developmental stages, illustrations and photographs of adults), taxonomic notes (when needed), material examined (including type material when applicable). Type specimen label data is given verbatim, with information for each line separated by a slash "/" and labels separated by a double slash "//"). The notes section was compiled from literature and from the labels of the examined specimens, representing a major part of the novel information presented in this work. We also provide high definition habitus photographs of most species (methodology is described in Thomas-Cabianca et al. 2021).

The material examined for South Africa is included in Suppl. material 1 (divided by species). Specimens from other Afrotropical countries are listed in Suppl. material 2 (Data also available at https://doi.org/10.15468/wmpu5c). The supplementary material files include information obtained and adjusted from the specimen labels, here presented following the Darwin Core: acceptedNameUsage, adjustedName, basisOfRecord, catalogNumber, country, dateIdentified, decimalLatitude, decimalLongitude, eventDate, eventRemarks, family, genus, habitat, identifiedBy, individualCount, institutionCode, lifeStage, locality, locationRemarks, maximumElevationInMeteres minimumElevationInMeteres, occurrenceID, order, originalName, otherCatalogNumbers, preparations, previousIdentifications, province, recordedBy, samplingProtocol, specificEpithet, scientificName, scientificNameAuthorship, sex, state, taxonRemarks, taxonomicStatus, typeStatus, verbatimCoordinates, verbatimEventDate, verbatimLocality. The bionomical section refers to South African data unless indicated otherwise. Abbreviations used include: BECE = Boyekoli Ebale Congo Expedition, KR = Knut Rognes identification database number, AT = allotype, HT = holotype, LT = lectotype, PLT = paralectotype, PT = paratype, ST = syntype.

Distribution data were obtained from Malloch 1926, Séguy 1949, Peris 1952a, Peris 1956, Séguy 1958, Zumpt 1958, Rickenbach et al. 1962, Zumpt 1962a, Zumpt 1972a, Zumpt 1972b, Zumpt 1974, Dear 1977, Pont 1980, Hardy 1981, Zumpt 1981, Kurahashi 1986, Soós and Papp 1986, Rognes 1991, Woodley and Hilburn 1994, Deeming 1996, van Aartsen 1997, Drees 1998, Szpila 2000, Martínez-Sánchez et al. 2002, Nandi 2002, Rognes 2002, Verves 2003, Ta Huy 2004, Verves 2005, Kurahashi and Kirk-Spriggs 2006, Lehrer 2007b, Verves 2007, Deeming 2008, Prado e Castro et al. 2010, Bharti 2011, Lehrer 2011, Rognes 2013, Setyaningrum and Al Dhafer 2014, Šuláková et al. 2014, Yang et al. 2014, Hansen et al. 2015, Hassan et al. 2018, Lutovinovas and Kinduris 2018, Dawah et al. 2019, El-Hawagry and El-Azab 2019 and Verves and Khrokalo 2020. When a record was ambiguous or doubtful, the corresponding country is marked with a "?". The geographic names (countries and South African provinces) are based on Kirk-Spriggs (2017) and Phillips (2017).

A distribution gazetteer (Suppl. material 3) for South African localities was created from the label information of the material examined. The gazetteer includes the original name of each collecting locality, the corrected or current locality name (when applicable), geographical coordinates and source of georeference method. For instance, "Capland" (old German name predating "Cape Province") and "Cape Province" were changed to Eastern, Northern or Western Cape Provinces, as apropriate. DMSA, SAMC and AMGS provided the geographic coordinate data of the material studied. When geographic coordinates were not provided, they were generated based on the reported collecting locality using Jason Londt's NMSA database (Londt, unpubl.) and Google Earth Pro Software (version 7.3.2.5776) with an integrated QRGS layer for South Africa. When the locality was insufficiently specific (e.g. only including the name of a settlement or province), the geographic reference used was the centroid middle point of the locality or the data provided by DMSA, SAMC or AMGS for the region. When it was too vague (e.g. “in Cape”, “Natal”, “Transvaal” etc.), it was omitted. Species distribution maps, including records for South Africa, Lesotho and Eswatini, were created.

## Checklists

### Annotated checklist of the Rhiniinae of South Africa

#### Rhiniini

9C1326CF-CDEB-5BBB-999B-2D5A3A3BAEDA

#### Fainia

Zumpt, 1958

E45B5CC4-311C-536C-8DCD-69F4B3F81558

= Fainia Zumpt, 1958: 83. **Type species**: *Idiaalbitarsis* Macquart, 1846, by original designation.

#### Fainia albitarsis

(Macquart, 1846)

82B68A32-90F2-5855-AA84-2554FE703BA4

= Idia albitarsis Macquart, 1846: 321. **Type locality**: South Africa, Cafreria.
= Idia eupoda Loew, 1852: 660 [1862: 24]. **Type locality**: Mozambique, Inhambane.
= Idia extensa Walker, 1858: 211. **Type locality**: South Africa, Port Natal [= Durban].
= Fainia sambura Lehrer, 2008: 16. **Type locality**: Kenya, Taita Hills, 1000–2000 m, Wyundani Rd., 3º24'S 38º23'E.

##### Distribution

**Figure 1.**
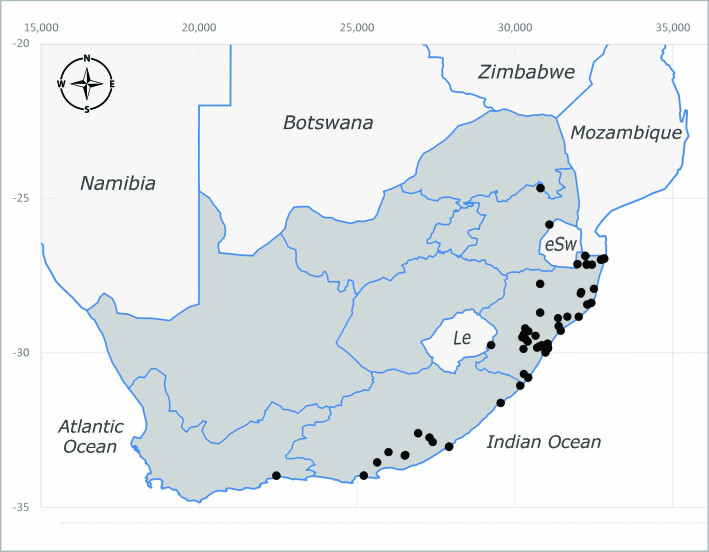
*Fainiaalbitarsis* occurrence map in South Africa, including eSwatini (eSw) and Lesotho (Le); horizontal axis longitude east and vertical axis latitude south.

**Afrotropical**: Central African Republic, Democratic Republic of Congo, ?Ghana, Kenya, Malawi, Mozambique, Namibia, Sierra Leone, South Africa (Fig. 1), Sudan, Tanzania, Uganda and Zimbabwe.

##### Notes

**Figure 2.**
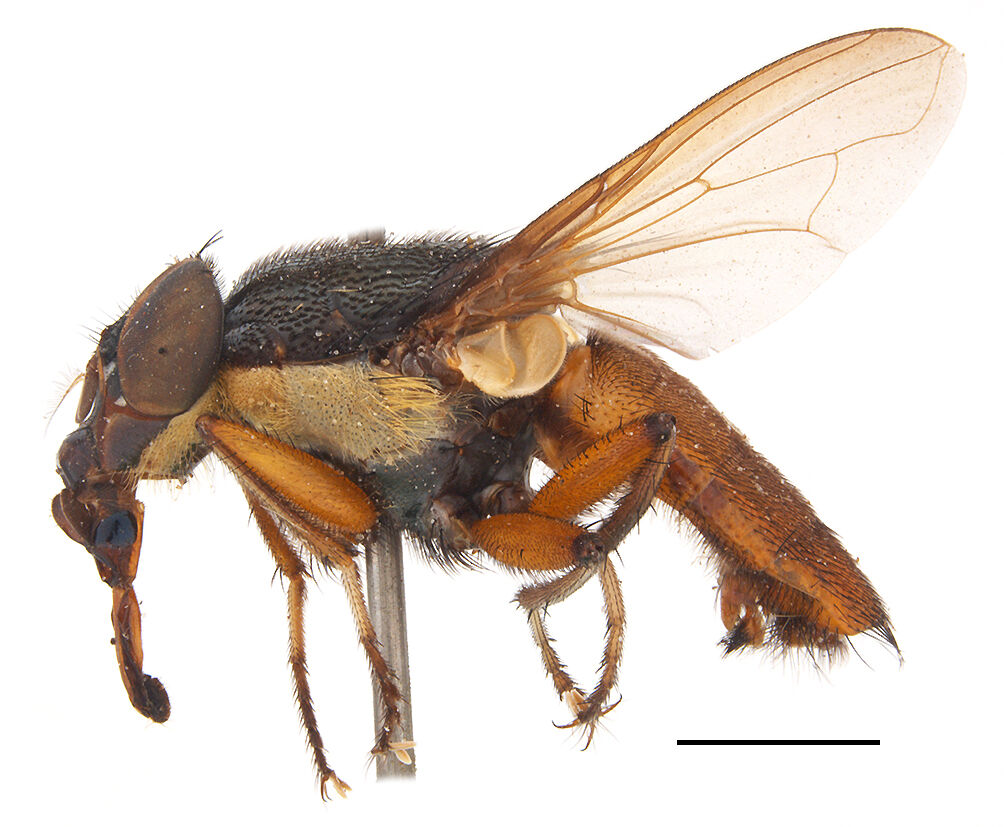
Habitus, left lateral view of *Fainiaalbitarsis* male SAMC DIP A015190 from South Africa. Scale bar = 2 mm.

**Preferred environment**: Afromontane forests, sand forests, dry scrub forest, open savannah/grassland, coastal bush and *Ficus* L. forest. In Namibia, a single specimen was reported from the Arid Savannah Biome (Kurahashi and Kirk-Spriggs 2006). Additionally collected in lowland rain forest in Tanzania, dry forest in Kenya and lowland evergreen secondary forest in the Democratic Republic of Congo. **Recorded elevations**: 10–1750 m a.s.l. **Seasonality**: a common and abundant species, collected year-round, most abundant during the warmer months, peaking in December and less abundant between March and October. In Namibia, a single specimen was caught in December (Kurahashi and Kirk-Spriggs 2006). **Behaviour and ecology**: associated with flowers in wild and rural environments. One male collected on *Cassine* L. flowers in the Drakensberg area (KwaZulu-Natal). Attracted to freshly-turned soil. Many males were observed and caught hovering in groups at the Amatigulu Nature Reserve (KwaZulu-Natal). Reported also in Zimbabwe as a flower-frequenting fly (as *Stomatorhinaalbitarsis* (Macquart) and as *Stomorhinaextensa* (Walker)) (Cuthbertson 1933, Cuthbertson 1934). **Life cycle and developmental stages**: unknown. **Collection methods**: more often with Malaise traps, followed by sweeping. Some specimens labelled as reared/ex Malaise trap, but without other details. Tanzanian and Kenyan specimens were collected by sweeping and Malaise and pitfall traps. **Illustrations and photographs**: male habitus as in Fig. 2 and figs. 5A–L in Thomas-Cabianca et al. (2021). Male terminalia as in fig. 8 in Peris (1952b), fig. 27 in Zumpt (1958)and figs. 6A–H in Thomas-Cabianca et al. (2021).

**Material examined**: Suppl. materials 1, 2.

#### Fainia elongata

(Bezzi, 1908)

3D62F807-4837-5B7E-9D61-569A706EA78C

= Stomatorrhina elongata Bezzi, 1908: 38. **Type locality**: Bas-Congo [= Democratic Republic of Congo].
= Idiella major Malloch, 1926: 510. **Type locality**: Sierra Leone, Masimera to Yonnibanna.

##### Distribution

**Figure 3.**
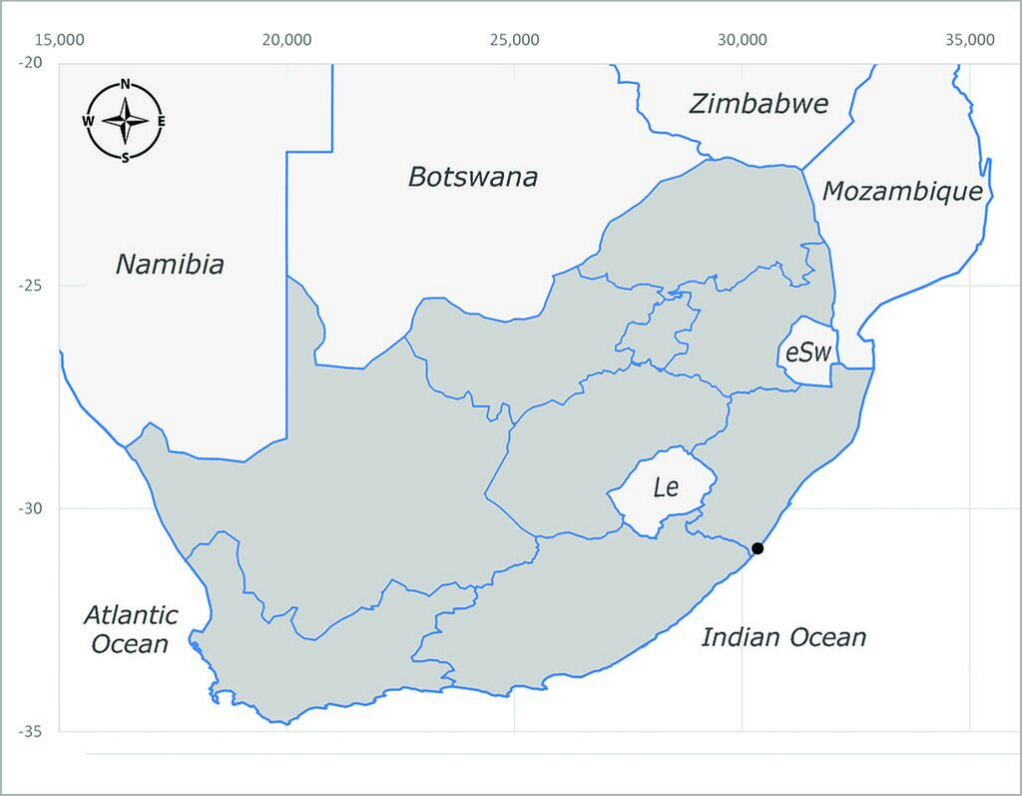
*Fainiaelongata* occurrence map in South Africa, including eSwatini (eSw) and Lesotho (Le); horizontal axis longitude east and vertical axis latitude south.

**Afrotropical**: Cameroon, Central African Republic, Democratic Republic of Congo, Equatorial Guinea, Côte d'Ivoire, Kenya, Madagascar, Malawi, Mozambique, Namibia, ?Nigeria, Rwanda, Sierra Leone, South Africa (Fig. 3), Sudan (reported as Sudan Anglo-Egyptian), Tanzania, Togo, Uganda and Zimbabwe.

##### Notes

**Figure 4.**
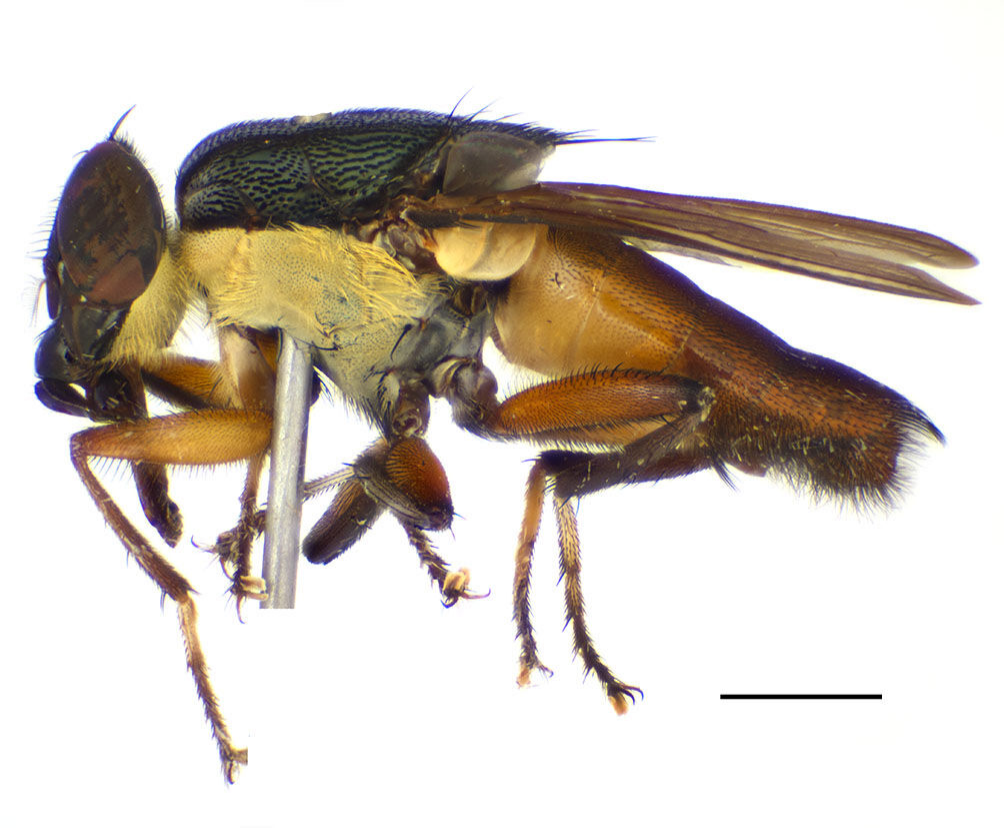
Habitus, left lateral view of *Fainiaelongata* male BMSA DIP (BECE) 03371 from Democratic Republic of Congo, scale bar = 2 mm.

**Preferred environment**: in Namibia, apparently restricted to the Arid and Mesic Savannah Biomes (Kurahashi and Kirk-Spriggs 2006). **Recorded elevations**: no data. **Seasonality**: A single specimen was collected in April. In Namibia, recorded in low numbers in December, February and March (Kurahashi and Kirk-Spriggs 2006). **Behaviour and ecology**: unknown. **Life cycle and developmental stages**: unknown. **Collection methods**: light traps. In Namibia, it was attracted in low numbers to rotten fish in baited traps (Kurahashi and Kirk-Spriggs 2006). Most often collected with Malaise traps in Central African Republic and Democratic Republic of Congo. **Illustrations and photographs**: male habitus as in Fig. 4 and figs. 8A–K in Thomas-Cabianca et al. (2021). Male terminalia as in fig. 7 in Peris (1952b), fig. 28 in Zumpt (1958) and figs. 9A–G in Thomas-Cabianca et al. (2021).

**Material examined**: Suppl. materials 1, 2.

#### Rhinia

Robineau-Desvoidy, 1830

607C2BC4-52FA-5849-B49A-0A043CE7CA49

= Rhinia Robineau-Desvoidy 1830: 422. **Type species**: *Rhiniatestacea* Robineau-Desvoidy, 1830 (= *Rhiniaapicalis* (Wiedemann, 1830)), by monotypy.
= Beccarimyia Rondani 1873: 287. **Type species**: *Beccarimyiaglossina* Rondani, 1873 (= *Rhiniaapicalis* (Wiedemann, 1830)), by monotypy.

#### Rhinia apicalis

(Wiedemann, 1830)

42EFF2E1-71ED-5A6A-A593-AAC575DB7660

= Idia apicalis Wiedemann, 1830: 354. **Type locality**: Canary Island, Tenerife.
= Rhinia testacea Robineau-Desvoidy, 1830: 423. **Type locality**: France, I'lle de France [= Mauritius].
= Idia flavipennis Macquart, 1844: 125. **Type locality**: Indonesia, Java.
= Idia simulatrix Loew, 1852: 660. **Type locality**: Olifant-River, South Africa.
= Idia punctata Bigot, 1858: 369. **Type locality**: Gabon.
= Idia bigoti Coquere, 1862: 96. **Type locality**: Senegal.
= Idia pleuralis Thomson, 1869: 542. **Type locality**: Australia, Keeling [= Cocos (Keeling)] Islands.
= Beccarimyia glossina Rondani, 1873: 287. **Type locality**: Abyssinia [= Ethiopia].
= Rhinia fulvipes Bigot, 1874: 239. **Type locality**: Ceylon [= Sri Lanka].
= Idiella trineuriformis Speiser, 1910: 153. **Type locality**: Tanzania, Kilimandjaro.

##### Distribution

**Figure 5.**
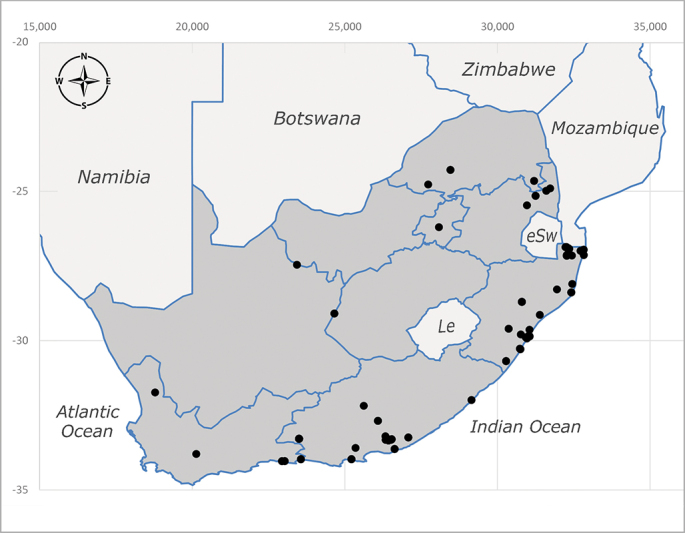
*Rhiniaapicalis* occurrence map in South Africa, including eSwatini (eSw) and Lesotho (Le); horizontal axis longitude east and vertical axis latitude south.

**Afrotropical**: Aldabra Island (Seychelles), Amirante Island (Seychelles), Angola, Benin*, Botswana, Burundi, Cameroon, Cosmoledo Island (Seychelles), Democratic Republic of Congo, Ethiopia, Gabon, Gambia, Ghana, Réunion Island (France)*, Madagascar, Malawi, Mauritus Island (Mauritius), Mozambique, Namibia, Nigeria, Oman, Rodriguez Island (Mauritius), Rwanda, Senegal, Sierra Leone, Socotra Island (Yemen), South Africa (Fig. 5), Tanzania, Togo*, Uganda, United Arab Emirates, Yemen, Zanzibar Island (Tanzania) and Zimbabwe. **Australasian**: Australia, French Polynesia, Fiji, Hawaiian Islands, Micronesia Islands, Papua New Guinea, Solomon Islands and Vanatu. **Palaearctic**: Azores Islands (Portugal), Canary Islands (Spain), China, Egypt, Iran, Israel, Jordan, Morocco, Palestine, Saudi Arabia, Syria and Turkey. **Oriental**: China, Hong-Kong, India, Indian Ocean Islands, Indonesia, Malaysia, Pakistan, Philippines, Sri Lanka, Taiwan, Thailand and Vietnam.

##### Notes

**Figure 6.**
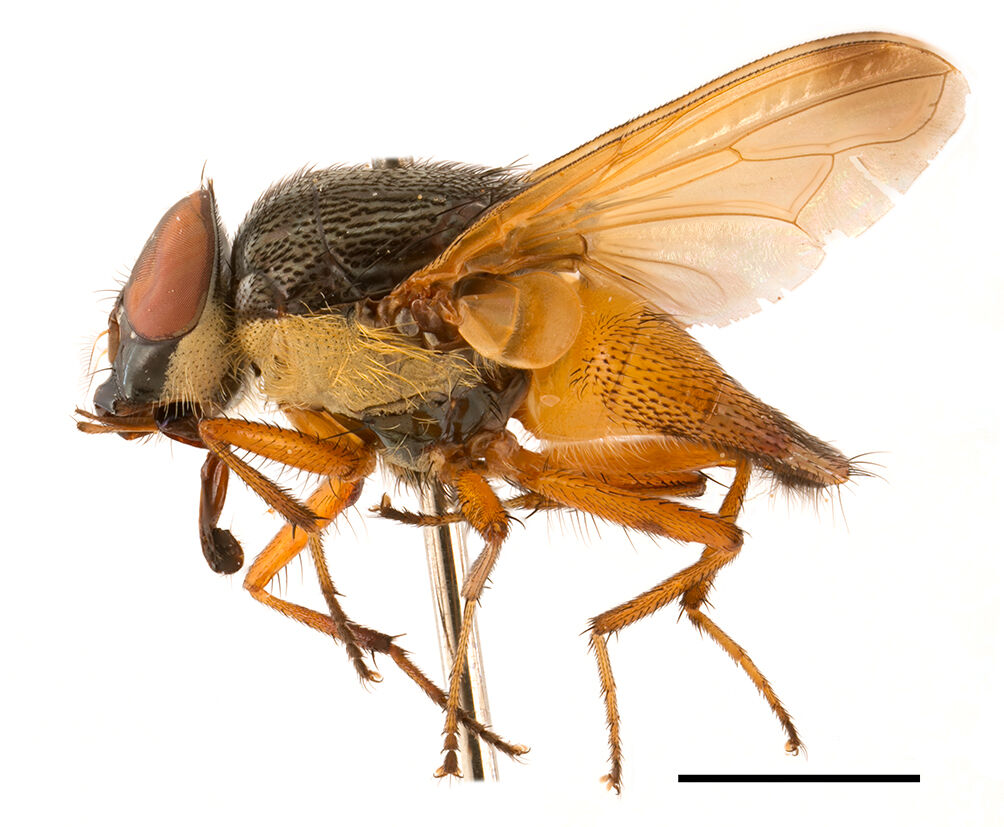
Habitus, left lateral view of *Rhiniaapicalis* male MZSUR from Kenya; scale bar = 2 mm.

**Preferred environment**: associated with a variety of anthropogenic and natural environments including poultry farms and gardens, dune and sand forests, dry scrub forests, *Ficus* forest, grassy floodplain, woodland savannah, broad-leaved deciduous woodland and Succulent Karoo. In Namibia, it occurs in all biomes, being especially abundant in the Mesic Savannah Biome (Kurahashi and Kirk-Spriggs 2006). In Burundi, the species was collected on the shore of Lake Tanganyika and in Benin. It was associated with mature secondary forest, remnant forest, agricultural plots, lowland gallery forest and streambeds. In Cameroon, the species was associated with cultivated plots, degraded savannah forest, grasses and other vegetation environments and in Réunion Island, with lowland tropical rainforest. **Recorded elevation**: 15–1000 m a.s.l. **Seasonality**: common species collected year-round, being most abundant in November, December and April and less abundant in August. In Namibia, abundance peaked in January and February (Kurahashi and Kirk-Spriggs 2006). **Behaviour and ecology**: considered a common flower visitor. Males and females have been observed feeding on pollen at flowers (Peris 1952a, Dear 1977) in Harare and Mutare, Zimbabwe (formerly Salisbury and Umtali, Rhodesia) during the late dry season (Cuthbertson 1933). A female was reported attending the nests of sphecoid and Pompilidae wasps (KwaZulu-Natal) and another one on the beach around a barbeque fire (Eastern Cape). Hulley (1983) reared adults from accumulated chicken manure in poultry houses from several locations in Eastern Cape. Additionally, adults have been observed at nests of *Bembix* Fabricius and *Cercerisyngvei* Cameron (as *Cercerisvumbui* Arn) (Hymenoptera) and larvae were obtained from nests of *Bembixmelanopa* Handlirsh (Cuthbertson 1938). Adults were attracted to freshly-removed soil during gardening in Grahamstown (Eastern Cape) (Martin Villet, personal observation 2016). A male was caught hovering in a group of males of *F.albitarsis* in the Amatigulu Nature Reserve, north of Tugela Mouth, KwaZulu-Natal. In Namibia, specimens have been collected from fresh elephant dung in the Caprivi Strip (Kurahashi and Kirk-Spriggs 2006). Cuthbertson (1933) reported females laying eggs in the soil at the bottom of aardvark burrows in Mbalabala (as Balla Balla, Zimbabwe) and in rich humus soil in thickets (Umzingwane, Zimbabwe). Cuthbertson (1938) also reports that females oviposit in soft earth excavated by driver ants (*Dorylus* Fabricius (Hymenoptera). **Life cycle and developmental stages**: oviparous species. Eggs, 3^rd^-instar larvae and puparia described and illustrated (Cuthbertson 1938). **Collection methods**: usually not reported, but Malaise traps seem to be the most common. Collected by sweeping in Burundi, Malaise traps in Benin and Réunion Island, Malaise traps and sweeping in Cameroon and UV-light, Malaise, yellow pans and pitfall traps in Namibia (Kurahashi and Kirk-Spriggs 2006). **Illustrations and photographs**: male habitus as in Fig. 6. Wing fig. 7 in Dear (1977). Male terminalia as in fig. 35 in Zumpt (1958), figs. IV. a, b, f in Baez and Santos-Pinto 1975, figs. 9, 21, 29, 37 in Dear (1977), slide 4 in González-Mora and Peris (1988) and figs. 93–101 in Rognes (2002). Female terminalia as in figs. 102, 103 in Rognes (2002).

**Material examined**: Suppl. materials 1, 2.

#### Rhinia coxendix

(Villeneuve, 1915)

58F3AFC3-B6E6-5C8C-A77A-0EDA795DFB23

= Idia coxendix Villeneuve, 1915: 204. **Type locality**: South Africa, Cap [= Cape Province].
= Rhinia pallidula Curran, 1927: 1. **Type locality**: Belgian Congo, Haut-Congo [Democratic Republic of Congo], Stanleyville [Kisangani].

##### Distribution

**Figure 7.**
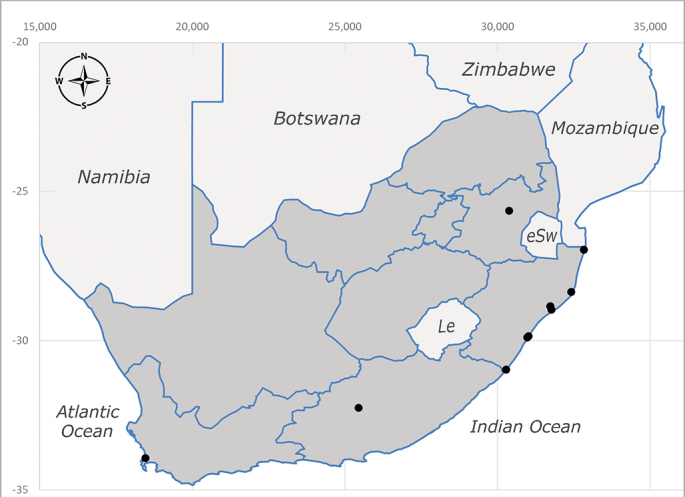
*Rhiniacoxendix* occurrence map in South Africa, including eSwatini (eSw) and Lesotho (Le); horizontal axis longitude east and vertical axis latitude south.

**Afrotropical**: Angola*, Burundi*, Cameroon, ?Cosmoledo Island (Seychelles), Democratic Republic of Congo, Kenya, South Africa (Fig. 7), Tanzania, Uganda and Zimbabwe*.

##### Notes

**Figure 8.**
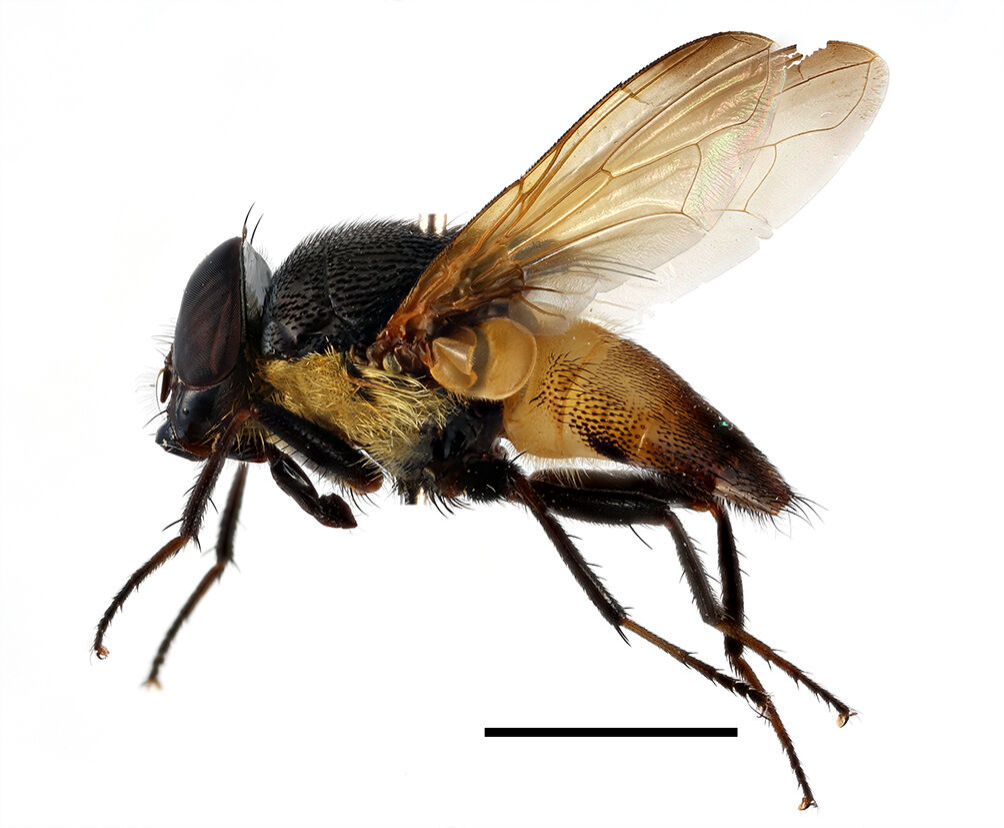
Habitus, left lateral view of *Rhiniacoxendix* male NMSA from South Africa; scale bar = 2 mm.

**Preferred environment**: associated with dune forest, coast scarp forest and coastal bush. In Democratic Republic of Congo, it was reported in lowland evergreen swamp forest. **Recorded elevations**: 50–1400 m a.s.l. **Seasonality**: uncommon species with highest numbers observed between January and February. No records in June, August and November; the rest of the year only one or two specimens per month. **Behaviour and ecology**: one specimen was collected inside the nest a *Sphextormentosus* Fabricius (Hymenoptera). **Collection methods**: Malaise traps. In Democratic Republic of Congo, collected with Malaise traps and in Tanzania, with hand net. **Life cycle and developmental stages**: oviparous; immature stages and life history unknown. **Illustrations and photographs**: male habitus as in Fig. 8. Male terminalia as in fig. 37 in Zumpt (1958).

**Material examined**: Suppl. materials 1, 2.

#### Rhinia nigricornis

(Macquart, 1843)

AB102E44-80F8-5EDA-849E-BF3199D25209

= Idia nigricornis Macquart, 1843: 281. **Type locality**: Senegal.
= Rhinia winthemi Villeneuve, 1915: 203. **Type locality**: Guinea.

##### Distribution

**Figure 9.**
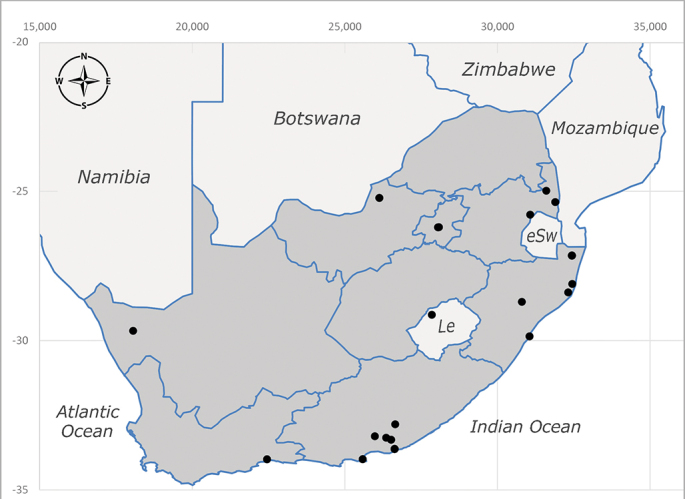
*Rhinianigricornis* occurrence map in South Africa, including eSwatini (eSw) and Lesotho (Le); horizontal axis longitude east and vertical axis latitude south.

**Afrotropical**: Botswana*, Cameroon, Democratic Republic of Congo, Equatorial Guinea*, Gambia, Ghana, Côte d'Ivoire, Lesotho*, Liberia, Madagascar, Malawi, Mozambique, Namibia, Nigeria, Senegal, Sierra Leone, South Africa (Fig. 9), Uganda, Yemen and Zimbabwe. **Palaearctic**: Saudi Arabia.

##### Notes

**Figure 10.**
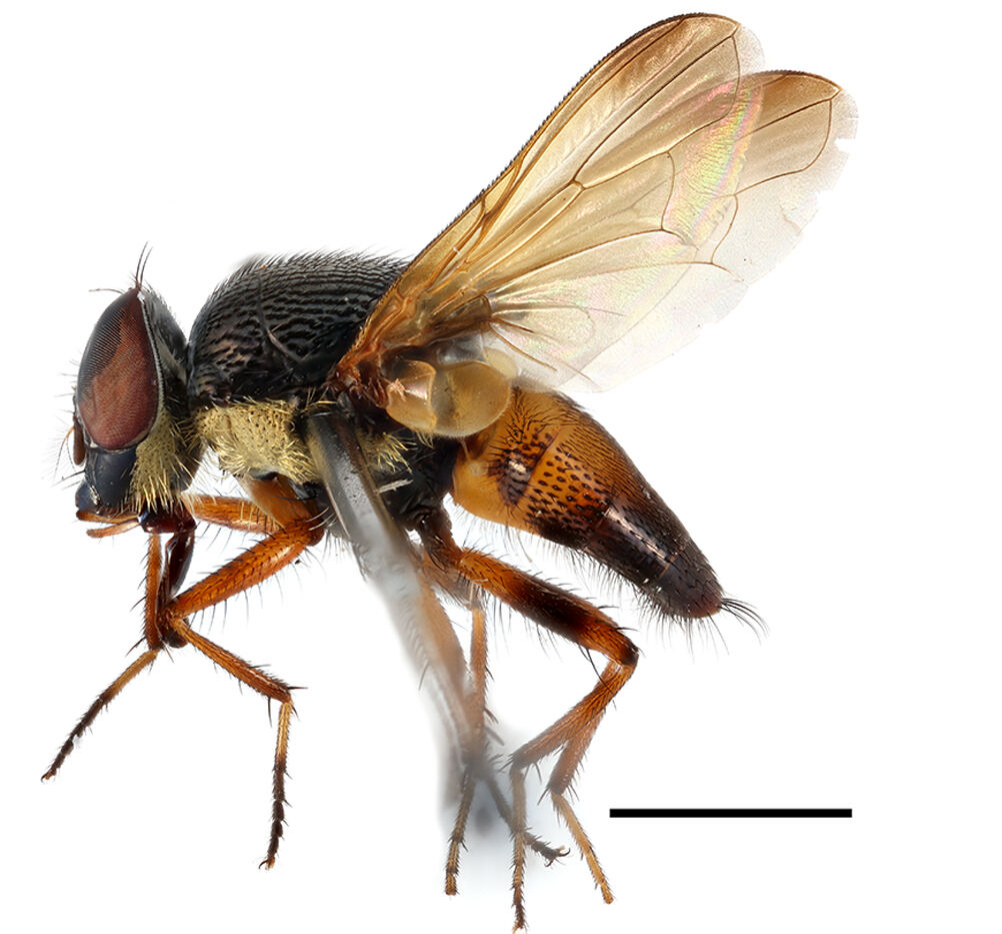
Habitus, left lateral view of *Rhinianigricornis* male NMSA from South Africa; scale bar = 2 mm.

**Preferred environment**: dry scrub forest and on the edge of a coastal forest in KwaZulu-Natal. In Namibia, apparently restricted to the Arid and Mesic Savannah Biomes (Kurahashi and Kirk-Spriggs 2006). **Recorded elevations**: 95 m a.s.l. **Seasonality**: more common in March, April and December, while absent or scarce for the rest of the year (1–3 specimens). In Namibia, most abundant in February (Kurahashi and Kirk-Spriggs 2006). **Behaviour and ecology**: collected on flowers of *Gymnosporialinearis* (L.f.) Loes. (as *Maytenuslinearis* (L.)) and “*Acacia*” (*Senegalia* Raf. or *Vachellia* Wight & Arn.) thickets. Females were observed attending nests of *Bembecinushaemorrhoidalis* (Handlirsch), Pompilidae and Sphecidae (Hymenoptera). **Life cycle and developmental stages**: unknown. **Collection methods**: most often collected with Malaise traps. In Namibia, it was reported as attracted to rotten fish and fermenting fruit in baited traps (Kurahashi and Kirk-Spriggs 2006). **Illustrations and photographs**: male habitus as in Fig. 10. Male terminalia as in fig. 36 in Zumpt (1958).

**Material examined**: Suppl. materials 1, 2.

#### Stomorhina

Rondani, 1861

18A5EB2F-6EE2-53EB-9852-FCE04493EC1F

= Idia Wiedemann, 1820: 21. **Type species**: *Muscalunata* Fabricius, 1805 by subsequent designation of Townsend (1916: 7).
= Stomorhina Rondani, 1861: 9 (replacement name for *Idia* Meigen by Wiedemann, 1820 (*nec* Hübner 1813)).
= Stomorhyna (misspelling of *Stomorhina* in *Stomorhynamaculata* Rondani 1865: 228)
= Stomathorrhina Bezzi, 1906: 53 (replacement name for *Idia* Wiedemann).
= Stomatorrhina Bezzi, 1906: 144 (unjustified emendation of *Stomathorrhina* Bezzi).
= Stomatorhina Speiser, 1910: 153 (misspelling of *Stomorhina*).
= Lomwerhina Lehrer, 2007: 12. **Type species**: *Lomwerhinamalobana* Lehrer, 2007, by original designation.

#### Stomorhina apta

Curran, 1931

C94943A8-1C75-55CF-B61E-F0C604B7CC1F

= Stomorhina apta Curran, 1931: 17. **Type locality**: Kenya Colony [Kenya], S. Masai Reserve [Maasai Mara National Reserve].

##### Distribution

**Figure 11.**
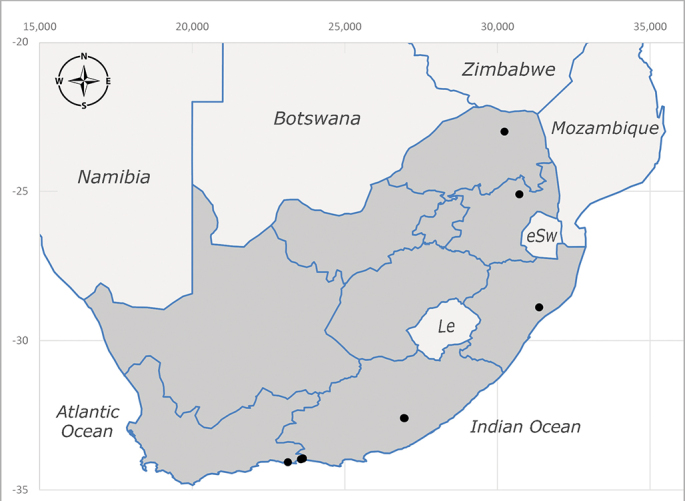
*Stomorhinaapta* occurrence map in South Africa, including eSwatini (eSw) and Lesotho (Le); horizontal axis longitude east and vertical axis latitude south.

**Afrotropical**: Burundi*, Democratic Republic of Congo, Kenya, South Africa* (Fig. 11) and Uganda.

##### Notes

**Figure 12.**
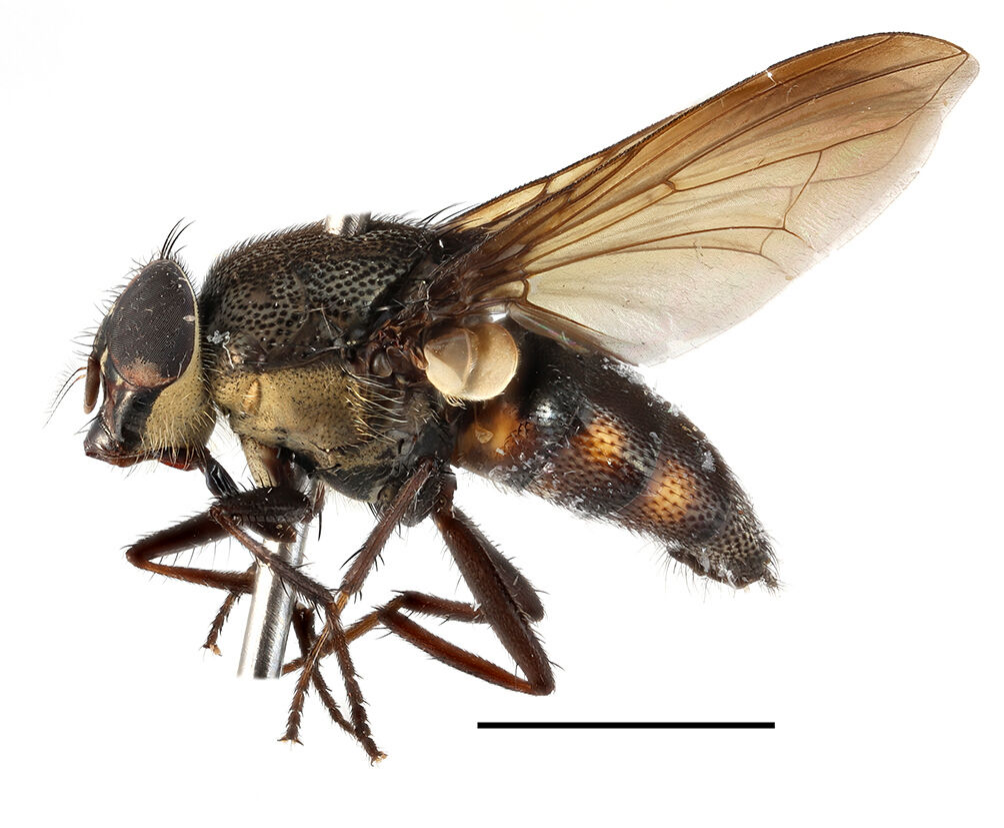
Habitus, left lateral view of *Stomorhinaapta* female BMSA DIP 24761 from Burundi; scale bar = 2 mm.

**Preferred environment**: associated with different environments, including: indigenous afromontane forest, indigenous mixed afromontane forest, indigenous forest, forest at the stream edge and Fynbos. In Burundi, it was associated with indigenous Afromontane forest and cloud forest. **Recorded elevations**: 30–1186 m a.s.l. **Seasonality**: uncommon species, most abundant in January with four specimens and present in September, November, December and April. The rest of the year it was absent. **Behaviour and ecology**: unknown. **Life cycle and developmental stages**: unknown. **Collection methods**: unknown. **Illustrations and photographs**: female habitus as in Fig. 12. Male terminalia as in fig. 29 in Zumpt (1958).

**Material examined**: Suppl. materials 2, 1.

#### Stomorhina armatipes

(Malloch, 1926)

AB3C4964-A1A5-5A76-A5A8-9226EB198DDE

= Stomatorrhina armatipes Malloch, 1926: 500. **Type locality**: South Africa, Natal [KwaZulu-Natal].
= Stomorhina fasciculata Curran, 1927: 528. **Type locality**: South Africa, Natal [KwaZulu-Natal], Willow Grange.

##### Distribution

**Figure 13.**
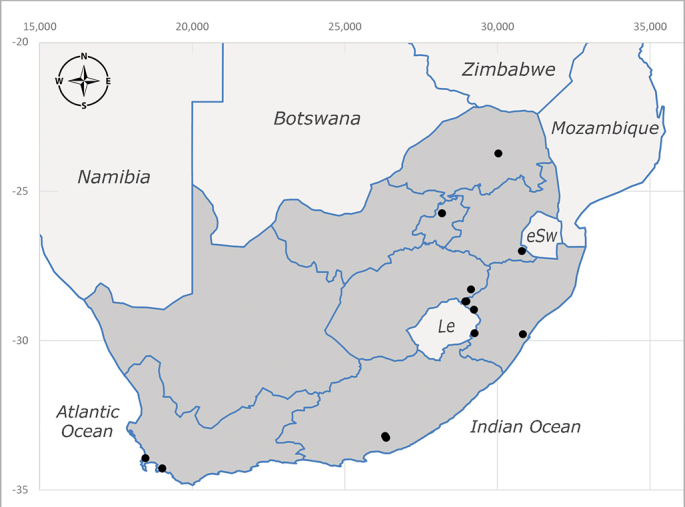
*Stomorhinaarmatipes* occurrence map in South Africa, including eSwatini (eSw) and Lesotho (Le); horizontal axis longitude east and vertical axis latitude south.

**Afrotropical**: Kenya and South Africa (Fig. 13).

##### Notes

**Figure 14.**
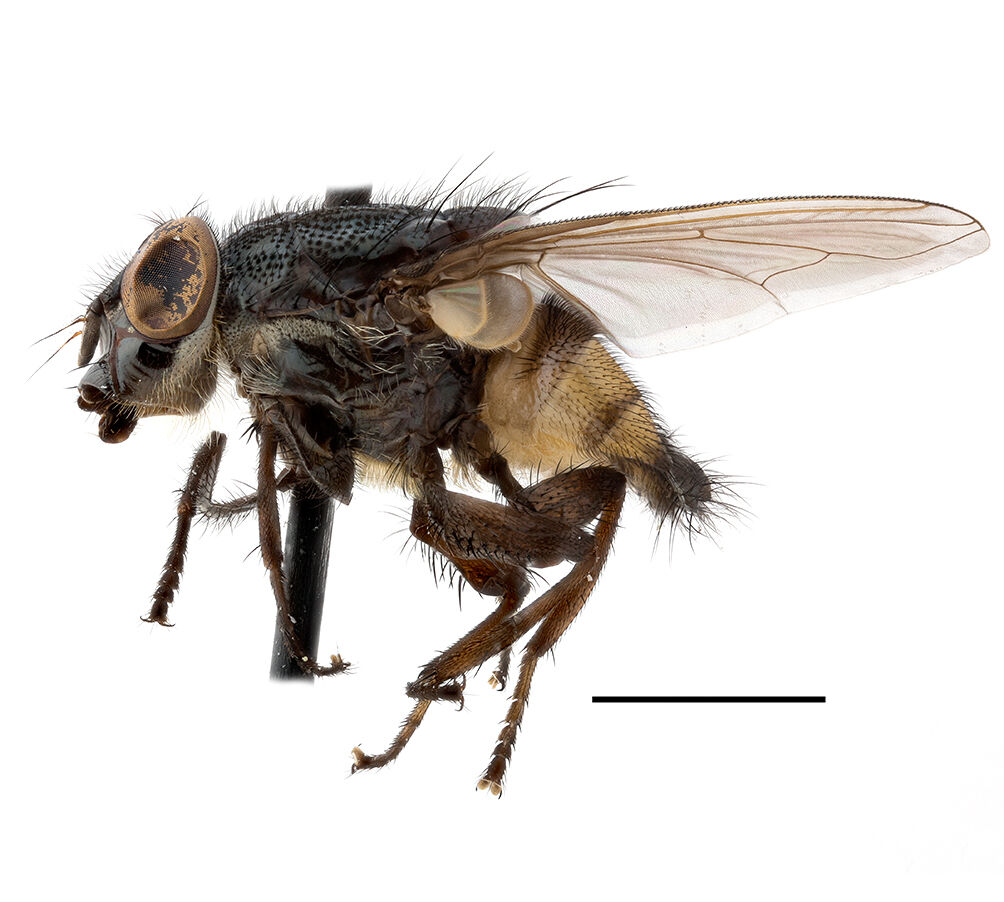
Habitus, left lateral view of *Stomorhinaarmatipes* male CEUA from South Africa; scale bar = 2 mm.

**Preferred environment**: no data. **Recorded elevations**: 1440–1750 m a.s.l. **Seasonality**: most abundant in September; absent or scarce for the rest of the year. **Behaviour and ecology**: adults were observed visiting flowers and collected on *Cassine* sp., *Proteacaffra* Meisn. and *Cussonia* Thunb. sp. Additionally, two males were found as prey of *Oxybeluslingula* Gerstaecker (Hymenoptera) in Grahamstown (Eastern Cape). During field work in September 2016 at the Kogelberg Nature Reserve, females were observed ovipositing on soil surrounding a termite nest. Gravid females and termites were collected and oviposition was achieved using a mixture of soil and live termites. Larvae were reared to adulthood and were observed preying on termites. **Life cycle and developmental stages**: oviparous. Immature stages (egg, larva and pupa) and adult females will be described (Thomas-Cabianca et al., unpublished). **Collection methods**: Malaise traps and sweeping. **Illustrations and photographs**: male habitus as in Fig. 14. Male terminalia unknown.

**Material examined**: Suppl. material 1.

#### Stomorhina chapini

Curran, 1931

3C0A8735-F9CB-5835-93F6-37EE2F0BBF83

= Stomorhina chapini Curran, 1931: 16. **Type locality**: Zaire [Democratic Republic of Congo], Lukulela.
= Rhinia patrizii Peris, 1952: 29. **Type locality**: Kenya, Ngong. **Remarks**: Zumpt (1962) indicated that *S.patrizzi* is identical to *S.chapini* after he examined the type series at NHMUK, but did not suggest it as Syn. nov. Subsequently, Pont (1980) considered this as a synomyn of *S.chapini*.

##### Distribution

**Figure 15.**
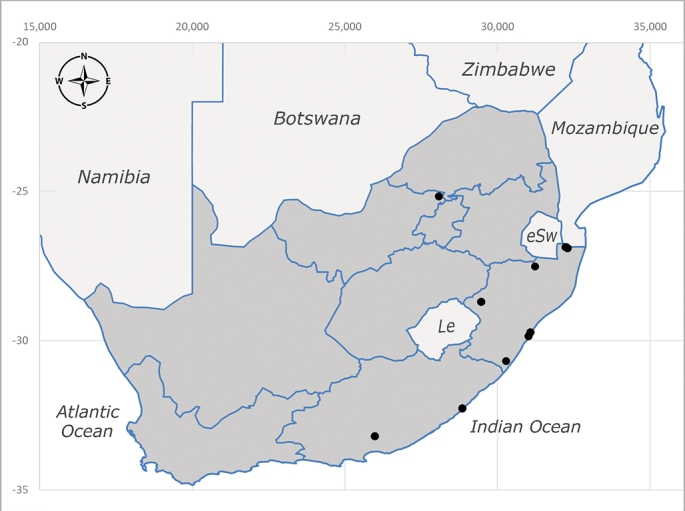
*Stomorhinachapini* occurrence map in South Africa, including eSwatini (eSw) and Lesotho (Le); horizontal axis longitude east and vertical axis latitude south.

**Afrotropical**: Cameroon, Democratic Republic of Congo, Kenya, Liberia, Namibia, South Africa (Fig. 15), Tanzania, Uganda. **Palaearctic**: Saudi Arabia.

##### Notes

**Figure 16.**
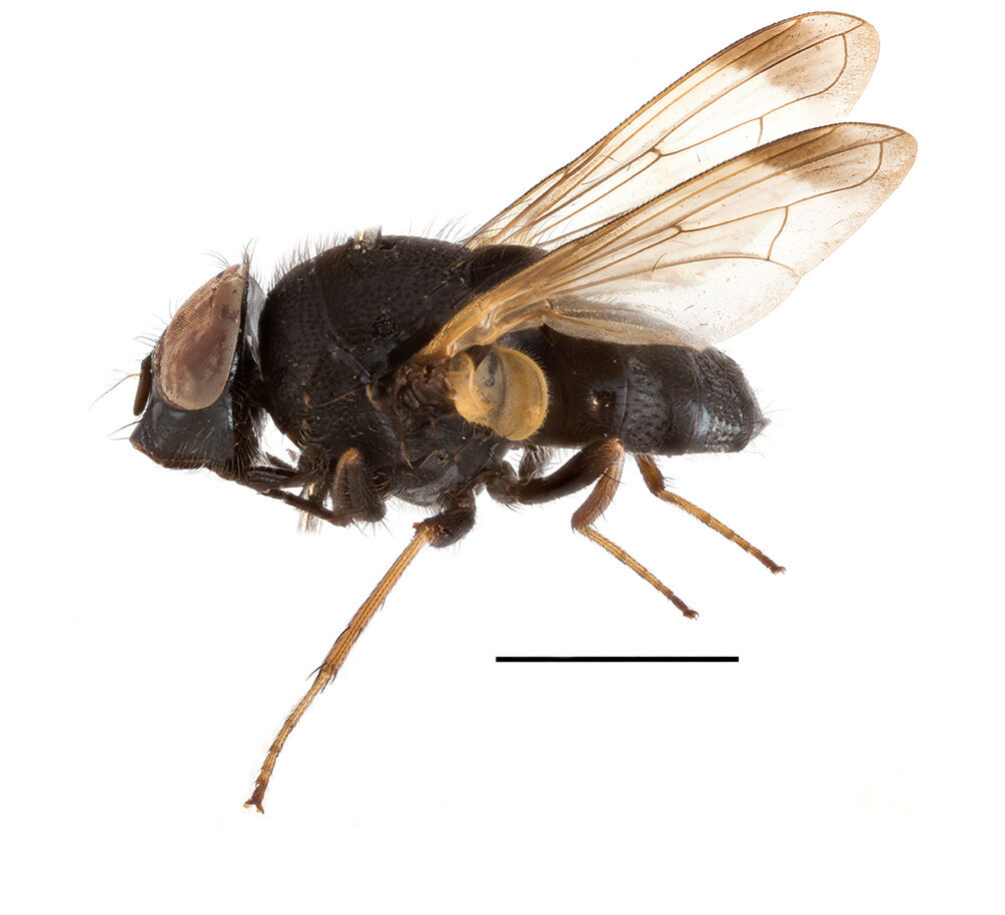
Habitus, left lateral view of *Stomorhinachapini* male AMGS 100664 from South Africa; scale bar = 2 mm.

**Preferred environment**: associated with *Ficus* forest, *Acacia* thornveld, indigenous forest and margin, sand forest and broad-leafed deciduous forest and long grass and woodland areas. In the Democratic Republic of Congo, the species was associated with lowland evergreen primary forest (disturbed). In Namibia, a single record was reported from the Mesic Savannah Biome (Kurahashi and Kirk-Spriggs 2006). **Recorded elevations**: 50-800 m a.s.l. **Seasonality**: highest abundance in April and from November to January; absent in February, September and October. Uncommon the rest of the year (1-2 specimens per month). **Behaviour and ecology**: unknown. **Life cycle and developmental stages**: unknown. **Collection methods**: Malaise traps in Namibia (Kurahashi and Kirk-Spriggs 2006). **Illustrations and photographs**: male habitus as in Fig. 16. Male terminalia as in fig. 30 in Zumpt (1958).

**Material examined**: Suppl. materials 1, 2.

#### Stomorhina cribrata

(Bigot, 1874)

C39266FF-7C31-5189-A90C-630AF7E473EA

= Rhinia cribrata Bigot, 1874: 239. **Type locality**: Sierra Leone, Yiraia; Sierra Leone, Dilijuli.
= Rhinia vertebrata Bigot, 1891: 378. **Type locality**: Ivory Coast [Côte d'Ivoire], Assinie.
= Rhinia tricincta Bigot, 1891: 379. **Type locality**: Ivory Coast [Côte d'Ivoire], Assinie.
= Rhinia striata Becker, 1912: 626. **Type locality**: Iran, Pers-Beludshistan [Sistan and Baluchestan Province].

##### Distribution

**Figure 17.**
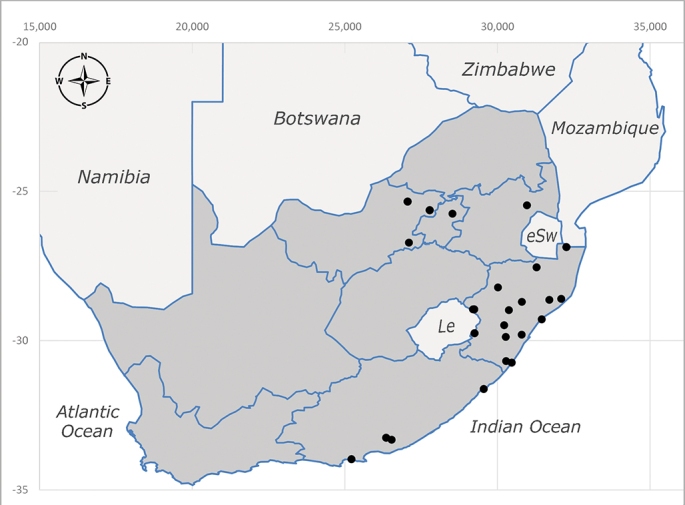
*Stomorhinacribrata* occurrence map in South Africa, including eSwatini (eSw) and Lesotho (Le); horizontal axis longitude east and vertical axis latitude south.

**Afrotropical**: Botswana, ?Burundi (plain Ruzizi), Cameroon, Democratic Republic of Congo, Gambia*, Ghana, Côte d'Ivoire, Kenya, Madagascar, Malawi, Mali, Namibia, Nigeria, Oman, Rwanda, Sierra Leone, South Africa (Fig. 17), Sudan (reported as Sudan Anglo-Egyptian), Tanzania, Zambia, Zimbabwe, Uganda and United Arab Emirates. **Palaearctic**: Cisjordan, Egypt, Iran, Israel, Saudi Arabia and Syria.

##### Notes

**Figure 18.**
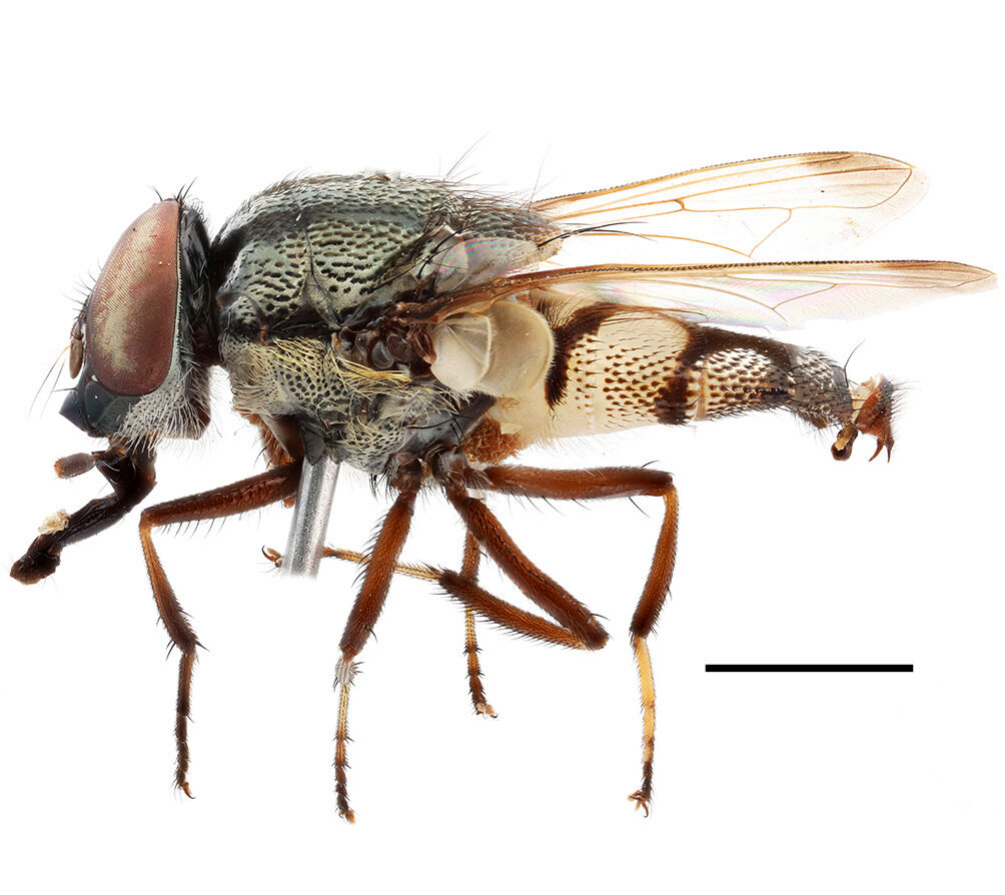
Habitus, left lateral view of *Stomorhinacribrata* male ZMUC from Tanzania; scale bar = 2 mm.

**Preferred environment**: Indigenous forests (mixed woodland, margin of a dune forest), in grassveld near a stream and thornveld camp grounds. In Namibia, it is apparently restricted to the Arid and Mesic Savannah Biome (Kurahashi and Kirk-Spriggs 2006). **Recorded elevations**: 10–1800 m a.s.l. **Seasonality**: most abundant in December, less abundant in January, March and September and absent or scarce the rest of the year. In Namibia, it was most abundant in October (Kurahashi and Kirk-Spriggs 2006). In Zimbabwe (as *Rhiniatricincta*), it was recorded as abundant from March to May (Cuthbertson 1933). **Behaviour and ecology**: a female was collected on avocado (*Perseaamericana* Mill) (Mpumalanga) and *Cassine* sp. flowers (KwaZulu-Natal). It was also collected together with *Oxybeluslingula* (Hymenoptera). Kurahashi and Kirk-Spriggs (2006) indicate on labels that females and males were observed hovering syrphid-like, usually at dusk, often around the margins of isolated trees. Swarm-hovering also recorded by (Cuthbertson 1938). **Life cycle and developmental stages**: females were observed ovipositing in soil rich in humus at the edge of cattle dung patch under tree shades and surrounded by long grass (Cuthbertson 1933). Cuthbertson (1933) also noted that the larvae live in the soil at the bottom of aardvark burrows and amongst the dead termites. Erzinçlioglu (1984) reared larvae and described the eggs, larva and pupa from a single specimen. In Zimbabwe, eggs hatch immediately after being laid and the larvae burrow into the soil (Cuthbertson 1933). Pupation occurs 6–7 days from hatching and the adults emerge in 7–9 days. **Collection methods**: Malaise traps in Namibia (Kurahashi and Kirk-Spriggs 2006). **Illustrations and photographs**: male habitus as in Fig. 18. Male terminalia as in fig. 34 in Zumpt (1958), figs. 289, 297 in Rognes (2002) and figs. 7A–G in Lehrer (2007b). Female terminalia as in figs. 298, 299 in Rognes (2002).

**Material examined**: Suppl. materials 1, 2.

#### Stomorhina guttata

(Villeneuve, 1914)

B01141B7-C077-5754-965A-237EA6DA7781

= Rhinia guttata Villeneuve, 1914: 384. **Type locality**: South Africa, Natal [KwaZulu-Natal], Willow Grange, Mooi River.

##### Distribution

**Figure 19.**
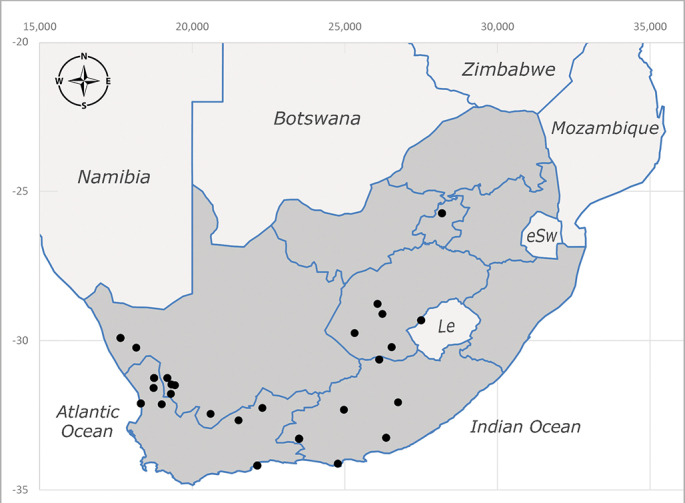
*Stomorhinaguttata* occurrence map in South Africa, including eSwatini (eSw) and Lesotho (Le); horizontal axis longitude east and vertical axis latitude south.

**Afrotropical**: Lesotho, Namibia and South Africa (Fig. 19).

##### Notes

**Figure 20.**
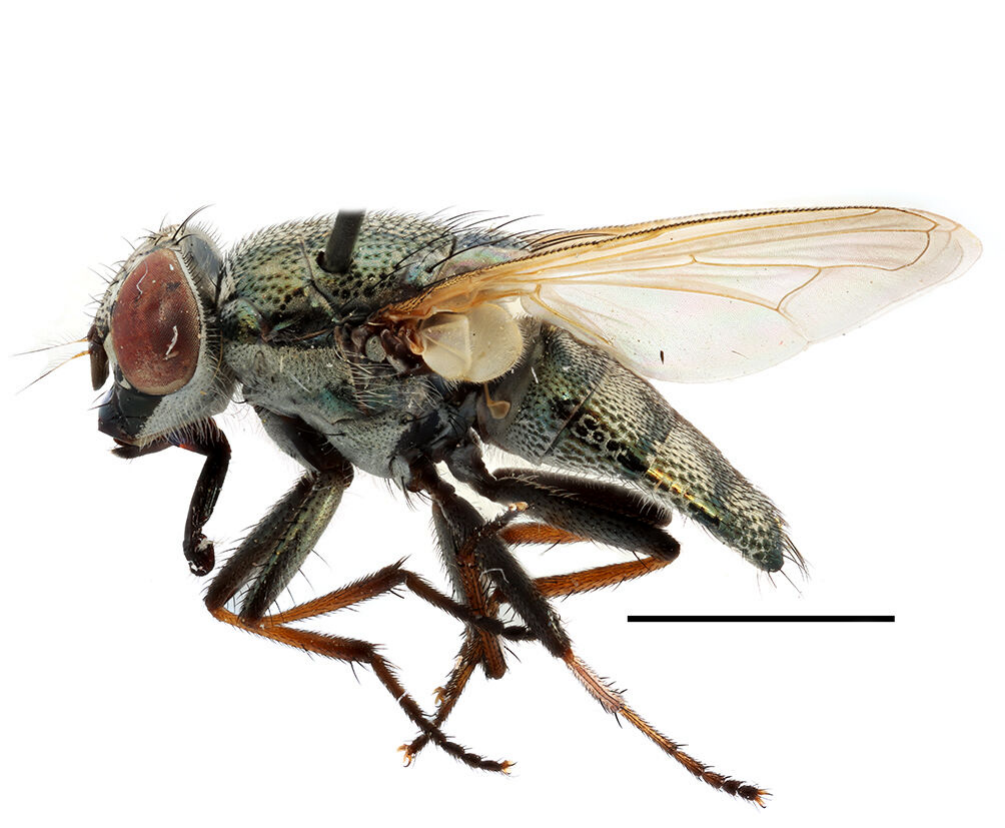
Habitus, left lateral view of *Stomorhinaguttata* female BMSA DIP 01831 from South Africa; scale bar = 2 mm.

**Preferred environments**: *Acacia* savannah, Succulent Karoo Scrub, white dune and coastal sand dunes biomes and to river banks, plains and slopes. Recorded in all Namibian biomes (Kurahashi and Kirk-Spriggs 2006). **Recorded elevations**: 120–1650 m a.s.l. **Seasonality**: high abundance between September-October and April. Present the rest of the year in low numbers, except in May and July. Recorded in low numbers in Namibia (Kurahashi and Kirk-Spriggs 2006). **Behaviour and ecology**: recorded as flower visitor; collected on *Lasiospermumbipinnatum* Druce and Knersvlakte flowers. Additionally, the larva was found in a termite fungus-garden (Peris 1952a). **Life cycle and developmental stages**: unknown. **Collection methods**: Malaise traps, yellow pan traps and by sweeping. In Namibia, yellow pans and pitfall traps (Kurahashi and Kirk-Spriggs 2006). **Illustrations and photographs**: female habitus as in Fig. 20. Male terminalia as in fig. 32 in Zumpt (1958).

**Material examined**: Suppl. materials 1, 2.

#### Stomorhina lunata

(Fabricius, 1805)

19125F96-ED26-5F05-8AB1-124478355115

= Musca lunata Fabricius, 1805: 292. **Type locality**: Portugal, Madeira Island. **Remarks**: type series specimen in ZMUC.
= Idia rostrata Wiedemann, 1820: 22. **Type locality**: South Africa, Cape of Good Hope - Promontorio bonae spei [Western Cape]. **Remarks**: type series specimen in ZMUC.
= Idia fasciata Meigen, 1826: 9. **Type locality**: France, Marseilles.
= Idia syrphoidea Robineau-Desvoidy, 1830: 421. **Type locality**: Mauritius.
= Idia cinerea Robineau-Desvoidy, 1830: 422. **Type locality**: Isles de la mer d'Africa [Indian Ocean d'Africa].
= Stomorhyna maculata Rondani, 1865: 228. **Type locality**: Italy, Parma.
= Stomorhina melanorhina Bigot, 1888: 592. **Type locality**: South Africa, Cape of Good Hope [Western Cape].
= Stomorhina muscoidea Brauer, 1899: 516. **Type locality**: Madagascar.
= Stomorhina selgae Lehrer, 1979: 89. **Type locality**: Bermuda.

##### Distribution

**Figure 21.**
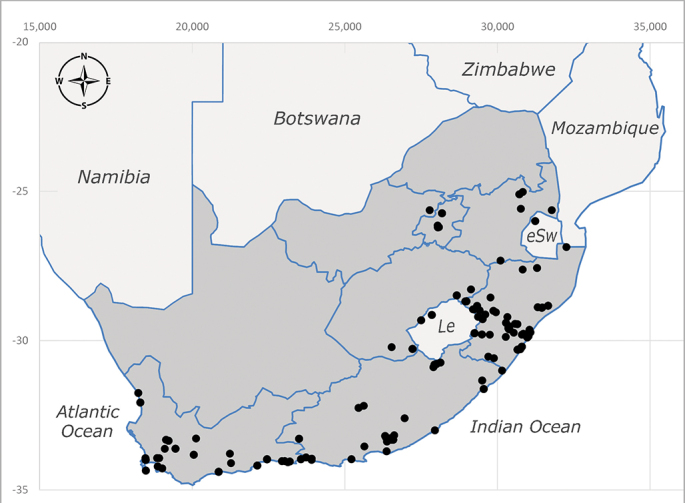
*Stomorhinalunata* occurrence map in South Africa, including eSwatini (eSw) and Lesotho (Le); horizontal axis longitude east and vertical axis latitude south.

**Afrotropical**: Angola, Burundi, Democratic Republic of Congo, Eritrea, Ethiopia, Kenya, Lesotho, Madagascar, Malawi, Mauricio Island (Mauritius), Oman, Namibia, Réunion Island (France), Rodriguez Island (Mauritius), South Africa (Fig. 21), Tanzania, Uganda, Yemen, Zambia and Zimbabwe. **Nearctic**: Bermuda. **Oriental**: China, India, Malaysia, Nepal, Pakistan, Taiwan. **Palaearctic**: Algeria, Armenia, Azerbaijan, China, Cyprus, Czech Republic, Denmark, Egypt, Finland, France, Georgia, Germany, Great Britain, Hungary, Iran, Iraq, Israel, Italy, Jordan, Kyrgyzstan, Lebanon, Lithuania, Morocco, Netherlands, Poland, Portugal (including Azores Islands), Russia, Saudi Arabia, Slovakia, Spain (including Canary Islands), Sweden, Syria, Tajikistan, Turkey, Turkmenistan, Ukraine and Uzbekistan.

##### Notes

**Figure 22.**
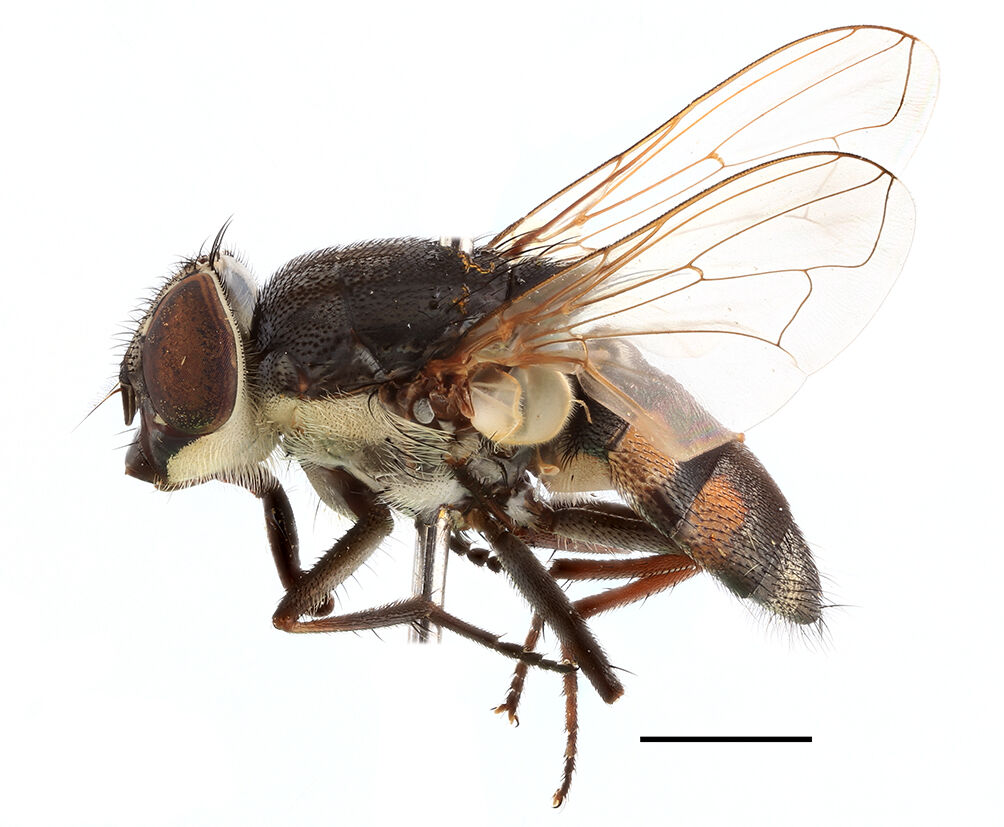
Habitus, left lateral view of *Stomorhinalunata* female AMGS 100730 from South Africa; scale bar = 2 mm.

**Preferred environment**: montane grass and woodlands, montane meadows, grasslands, rocky hillside, indigenous montane forest and forest margins, slopes, ravines, streams and cascade areas. Different kind of biomes such as: Macchia vegetation and old lands, mesic mountain Fynbos, false Macchia slopes and coastal Macchia. Associated with human environments such as houses, a university campus, caravan parks and main tracks through forest. Recorded in low numbers in Namibia, where it is virtually restricted to the Brandberg Massif and occurs at high elevations on the edge of Nama-Karoo (Kurahashi and Kirk-Spriggs 2006). In Mauritius, it was collected in a montane forest. **Recorded elevations**: 10–2080 m a.s.l. **Seasonality**: this is the most common, abundant and well-known species of Rhiniinae, present year-round. Highest abundance in September and January to February; lowest between May and July. Only four specimens recorded in Nambia (Kurahashi and Kirk-Spriggs 2006). **Behaviour and ecology**: collected on/and visiting flowers of *Foeniculumvulgare* Mill, *Gymnosporiaheterophylla* (Eckl. and Zeyh.) Loes., *Cassine* sp., *Buddleja* sp. L., *Cussonia* sp. and *Searsiacrenata* (Thunb.) Moffet. Additionally associated with: *Searsia* F. A. Barkley sp. (previously local *Rhus*), *Diospyros* L. sp. and *Celtis* L. sp.. In Mauritius, one specimen was collected on a flowering tree. The species seems to have a close relationship with Orthoptera. Adults lay eggs on oothecas, where the larvae developed (Bezzi 1911). Larvae are predators of locusts’ oothecas in Zimbabwe (Cuthbertson 1933, Cuthbertson 1934). Peris (1952a) recorded *S.lunata* on *Nomadacrisseptemfasciata* (Serville) oothecas and that it was obtained from locusts’ eggs ("Ex eggs") in Malawi (then Nyasaland) and Kenya. Additionally reported that larvae destroy oothecas of *Locustamigratorioides* (Reiche, L.J. and Fairmaire) in Kenya. In the material examined, *S.lunata* was reported as being attracted to open termite mounds (Pretoria, Gauteng) and found outside the nest of *Trinervitermes* Holmgren (Blattaria) (Johannesburg, Gauteng). Cuthbertson (1935) found larvae of *S.lunata* in the fungus beds of a termite nest and reared them on dead and dying termite workers and soldiers. Lewis (1955) reported the presence of *S.lunata* in carrion. **Life cycle and developmental stages**: Peris (1956) studied some drawings of *S.lunata* male terminalia made by S. Patrizi accompanied by biological notes from observations in Kenya. Patrizi indicated in his notes that females of *S.lunata* were observed throwing masses of eggs over ?*Anomma* Shuckard ants (Formicidae). Some eggs were collected and stuck strongly to the glass walls of the container. Later, the eggs were placed in an ant farm, larvae emerged after a few hours and entered into the interior of *Anomma* larvae completely eating them (predation). Finally, larvae migrated to the soil for pupation. Patrizi suggests that the sticky eggs are a mechanism for entering ants’ nests (Peris 1956). Cuthbertson (1934) studied the life history of *S.lunata*. Eggs (length: 1–1.25 mm) were deposited around soft soil close to a red locust ootheca, hatching in few minutes. First instar larvae were active (length: 1.5–1.75 mm) and quickly attacked the ootheca. In 3–4 days, larvae were fully fed (length: 2–14 mm), left the ootheca and migrated to soil to pupate (length pupae: 7.5–8 mm) for one-two days. Adults emerged in 7–10 days, but in cold weather, sometimes the pupal stage can last 14–15 days or longer. Adults' copulation occurred 4–5 days after they emerged and eggs started to be laid 1–2 days after copulation. Adults were fed with sugar solution, flower nectar and liquids from fresh cow-dung. Larvae stages fed on the yellow yolk of freshly-laid locust ootheca. Hall (1947) described eggs, 1st, 2nd and 3rd instar larvae and puparia from Cuthbertson's material and indicated that the life cycle seems to last 30 days in optimal conditions, where egg incubation was 18–24 hours, 1st instar was 14–24 hours, 2nd instar was 36–48 hours, 3rd instar was 2–3 days and pupation lasted 10–16 days in summer conditions. Greathead (1963) illustrated the 3rd instar larva (anal area, posterior spiracles and mouth parts) from a specimen collected in a locust's ootheca. Hall (1947) described the posterior spiracle, mouthparts and anal area of the 3rd instar larva as well and mouth parts of the 2nd instar larva. Immature stages are also illustrated in Cuthbertson (1935). **Collection methods**: sweeping with hand net and Malaise, yellow pan, black light and pitfall traps. Malaise and yellow pan traps in Namibia and hand net in Mauritius (Kurahashi and Kirk-Spriggs 2006). **Illustrations and photographs**: female habitus as in Fig. 22, fig. 10 in Greathead (1963) and fig. 3H in Prado e Castro et al. 2016. Male habitus as in fig. 1 in Lutovinovas and Kinduris (2018). Male terminalia as in fig. 32 (inaccurate) in Zumpt (1958), slide 3 in González-Mora and Peris (1988) and figs. 675–683 in Rognes (1991). Female terminalia as in figs. 684–685 in Rognes (1991).

**Type material examined**: *I.rostrata*: 1 ? / Mus. / Westerm. // Type // *I.rostrata* / Weid. / Cape of Good Hope / Jan: 1817 // [ZMUC 00025098]. *M.lunata*: 1 ? // [ZMUC 00027332].

**Material examined**: Suppl. materials 1, 2.

#### Stomorhina malobana

(Lehrer, 2007)

6A3DDB32-0E4E-5C86-AD35-70E301A95130

= Lomwerhina malobana Lehrer, 2007: 12. **Type locality**: Malawi, Mulanje Mt. near Likabula. **Remarks**: HT in SMNHTAU (TAUI) .

##### Distribution

**Figure 23.**
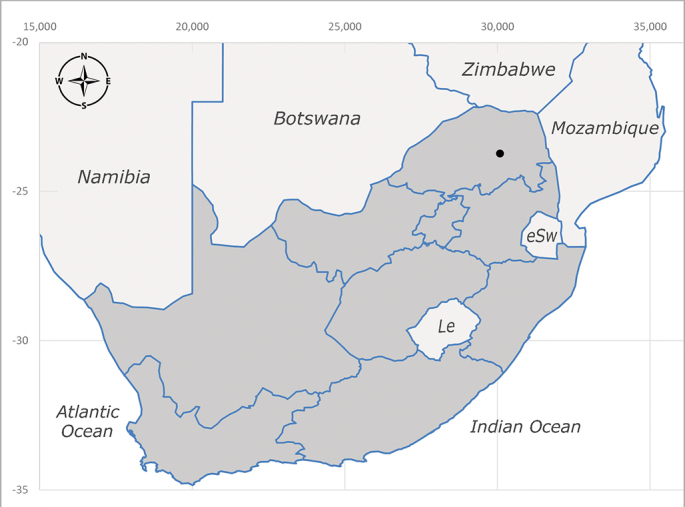
*Stomorhinamalobana* occurrence map in South Africa, including eSwatini (eSw) and Lesotho (Le); horizontal axis longitude east and vertical axis latitude south.

**Afrotropical**: Malawi, South Africa* (Fig. 23) and Tanzania*.

##### Notes

**Figure 24.**
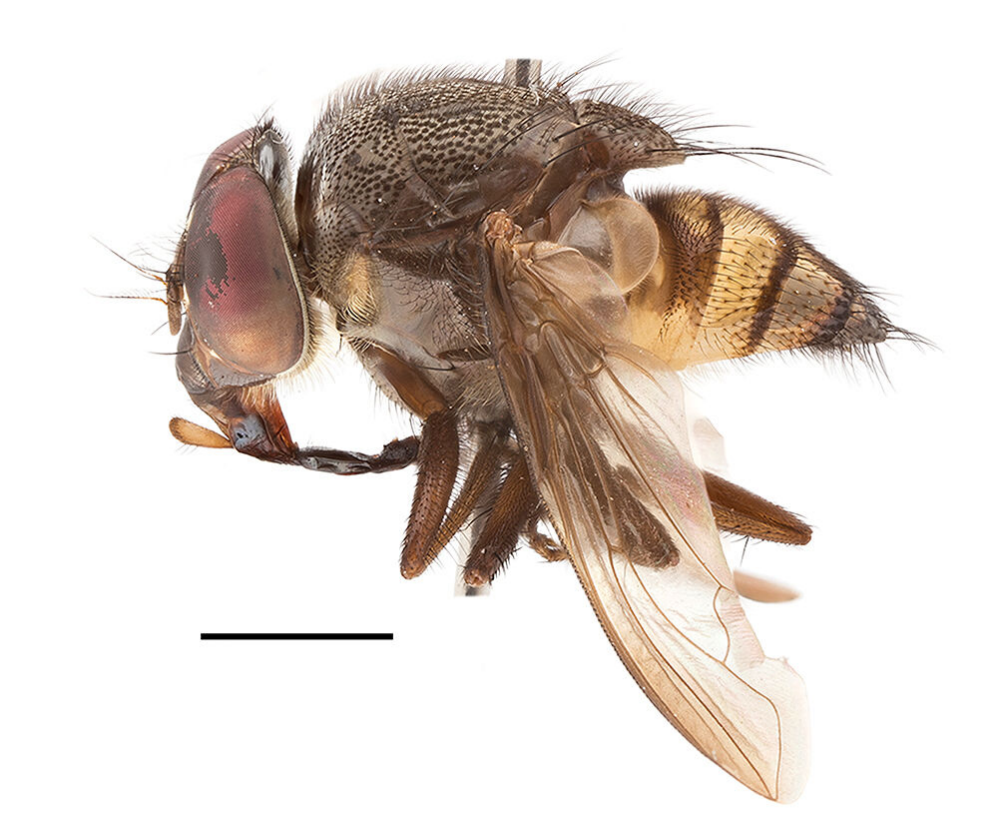
Habitus, left lateral view of *Stomorhinamalobana* male SANC from South Africa; scale bar = 2 mm.

**Preferred environment**: no data. **Recorded elevations**: 1050–1880 m a.s.l. **Seasonality**: the single specimen was collected in November. **Behaviour and ecology**: collected on avocado flowers (Gauteng). **Life cycle and developmental stages**: females collected in Tanzania contained a completely developed 3rd instar larva that occupied all of the abdomen (four females were dissected). This suggests an unilarviparous biology, an exclusive and new trait for the Afrotropical *Stomorhina* (Thomas-Cabianca et al., unpublished). **Collection methods**: in Tanzania, it was collected with Malaise and pitfall traps and a Malaise trap in Malawi. **Illustrations and photographs**: male habitus as in Fig. 24. Male terminalia as in figs. 9A-E in Lehrer (2007b).

**Type material examined**: *L.malobana*: 1 ? Malawi, 1500 m a.s.l., Mulanje Mt. nr. Likabula, 26-27.x.83, A. Freidberg // Holotype // *Lomwerhinamalobana* Det. Dr. A. Z. Lehrer, 2006 // SMNHTAU (TAUI) 318991.

**Material examined**: Suppl. materials 1, 2.

#### Stomorhina rugosa

(Bigot, 1888)

507C1454-0812-58C2-8272-15DD628942AD

= Rhinia rugosa Bigot, 1888: 591. **Type locality**: Sierra Leone.
= Stomorhina mitis Curran, 1931: 18. **Type locality**: South Africa, Natal [KwaZulu-Natal].
= Rhinia hyphena Séguy, 1958: 188. **Type locality**: Guinea.

##### Distribution

**Figure 25.**
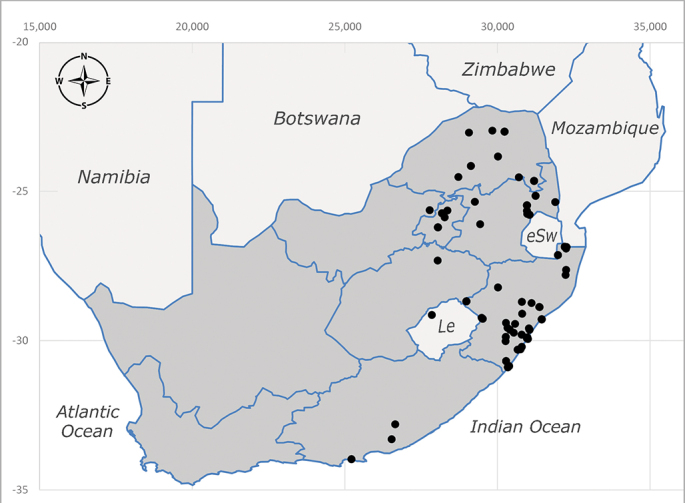
*Stomorhinarugosa* occurrence map in South Africa, including eSwatini (eSw) and Lesotho (Le); horizontal axis longitude east and vertical axis latitude south.

**Afrotropical**: Democratic Republic of Congo, Ghana, Guinea (today could be Guinea, Guinea-Bissau or Equatorial Guinea), Kenya, Lesotho*, Malawi, Mozambique, Namibia, Nigeria, Sierra Leone, Sudan (reported as Anglo-Egyptian Sudan), South Africa (Fig. 25), eSwatini*, Tanzania, Uganda, Zambia and Zimbabwe. **Palaearctic**: Saudi Arabia.

##### Notes

**Figure 26.**
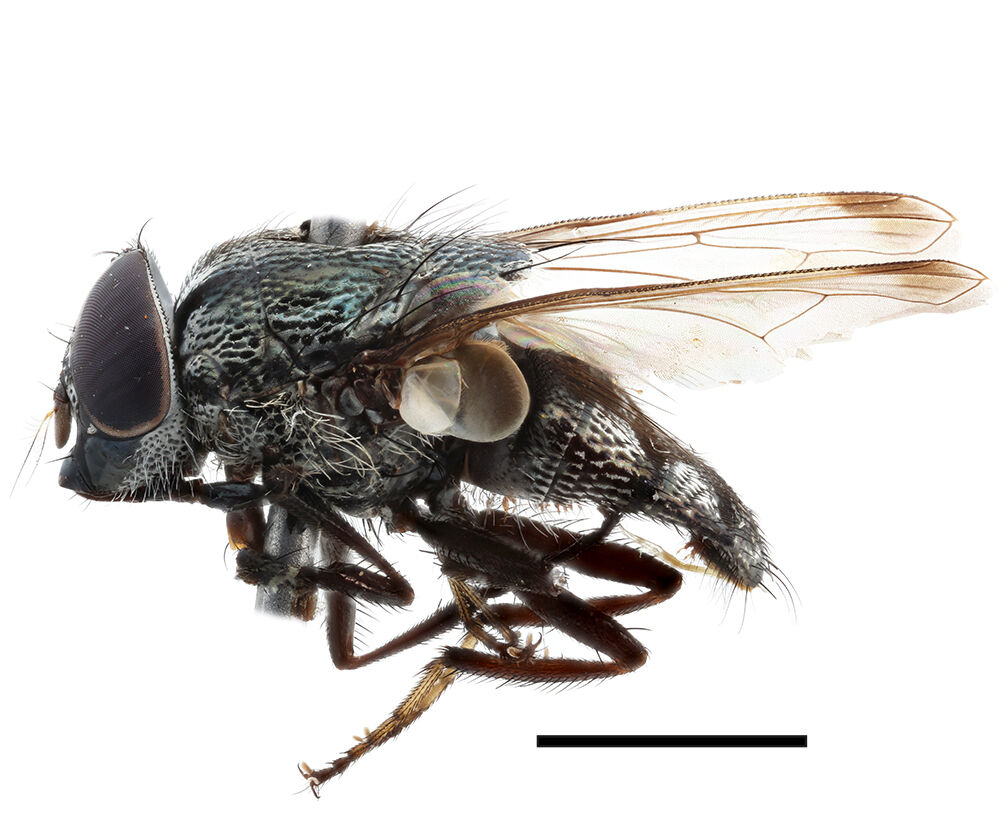
Habitus, left lateral view of *Stomorhinarugosa* male BMSA DIP 92522 from Malawi; scale bar = 2 mm.

**Preferred environment**: coast scarp forest, grassland, scrub and wooded grassland area, near streams, on the margin of dune forest, sewage-seepage area, bushveld and in picnic area. In Zimbabwe, the species was collected indoors and in Namibia, it was collected at a damaged termite mound and is apparently restricted to the Mesic Savannah Biome (Kurahashi and Kirk-Spriggs 2006). **Recorded elevations**: 10–1480 m a.s.l. **Seasonality**: present year-round with greatest abundance from November to February and least in May, August and September in South Africa. In Namibia, it was recorded in January and December in low numbers (Kurahashi and Kirk-Spriggs 2006). **Behaviour and ecology**: females were observed ovipositing in newly-excavated termite mounds. Peris (1952a) indicated that *S.rugosa* was caught on dung in Nigeria and seen buzzing over a termite nest in South Africa. In Ethiopia, a female of *S.rugosa* was seen emerging from a caterpillar of Fall Army Worm *Spodopterafrugiperda* (Smith) (Lepidoptera, Noctuidae) and the pupa was found inside the caterpillar (Tadele Tefera and Robert Copeland, personal communication). Specimens were collected on flowers of *Cussonia* sp. and *Poinsettia* Graham in South Africa. Cuthbertson (1934) (as *S.mitis*) found adults on daisy flowers in Eastern Victoria (Zimbabwe) in June 1932. Peris (1952a) reported specimens collected on honey-bearing wild flowers and on wild flowers in Zambia. **Life cycle and developmental stages**: unknown, but Cuthbertson (1934) indicated that eggs are large (1.5 mm) and hatch immediately after being deposited in soil. **Collection methods**: Malaise, blue pan and light traps. In Namibia, it was collected with Malaise traps (Kurahashi and Kirk-Spriggs 2006). **Illustrations and photographs**: male habitus as in Fig. 26. Male terminalia as in fig. 33 in Zumpt (1958).

**Material examined**: Suppl. materials 1, 2.

#### Cosminini

5F65D716-12C9-588A-9968-A400C67D71A5

#### Cosmina

Robineau-Desvoidy, 1830

4B0CD184-7970-51A3-BD99-4841C961106C

= Cosmina Robineau-Desvoidy, 1830: 423. **Type species**: *Cosminafuscipennis* Robineau-Desvoidy, 1830, by subsequent designation of Townsend (1916: 6).
= Seseromya Rondani, 1863: 32. **Type species**: *Muscapunctulata* Wiedemann, 1819 (= *Cosminafuscipennis* Robineau-Desvoidy, 1830), by original designation.
= Synamphoneura Bigot, 1887: xiv. **Type species**: *Synamphoneuracuprina* Bigot, 1887 (= *Idialimbipennis* Macquart, 1848), by original designation.
= Idiopsis Brauer and Bergenstamm, 1889:153. **Type species**: *Idiopsisprasina* Brauer and Bergenstamm, 1889, by monotypy.
= Eusynamphoneura Townsend, 1917: 189. **Type species**: *Idiaseriepunctata* Loew, 1852 (= *Dictyaaenea* Fabricius, 1805), by original designation.
= Synamphoneuropsis Townsend, 1917: 199. **Type species**: *Synamphoneuropsisviridis* Townsend, 1917, by original designation.

#### Cosmina aenea

(Fabricius, 1805)

E3529E35-7BB3-57C8-8C86-835894CE5BC5

= Dictya aenea Fabricius, 1805: 328. **Type locality**: "Guinea" Krieger, [Ghana, Teshi. **Remarks**: type-serie in ZMUC.
= Idia seriepunctata Loew, 1852: 660 [1864:32]. **Type locality**: Mozambique, Inhambane.
= Cosmina despressa Karsch, 1888: 377. **Type locality**: Tanganyika [Tanzania], Usambara.
= Cosmina punctulata var. microps Malloch, 1926: 518. **Type locality**: Gold Coast [Ghana], N. territories [North East Region], Yapi.

##### Distribution

**Figure 27.**
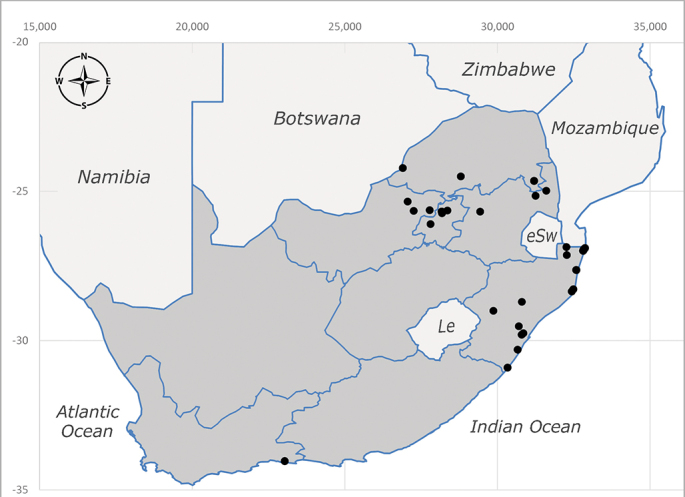
*Cosminaaenea* occurrence map in South Africa, including eSwatini (eSw) and Lesotho (Le); horizontal axis longitude east and vertical axis latitude south.

**Afrotropical**: Burkina Faso, Cameroon, Democratic Republic of Congo, Côte d'Ivoire*, Kenya*, Liberia, Malawi, Mali, Mozambique, Namibia*, Nigeria, South Africa (Fig. 27), Tanzania and Zimbabwe, “Ghana or Togo”*. **Palaearctic**: Saudi Arabia.

##### Notes

**Figure 28.**
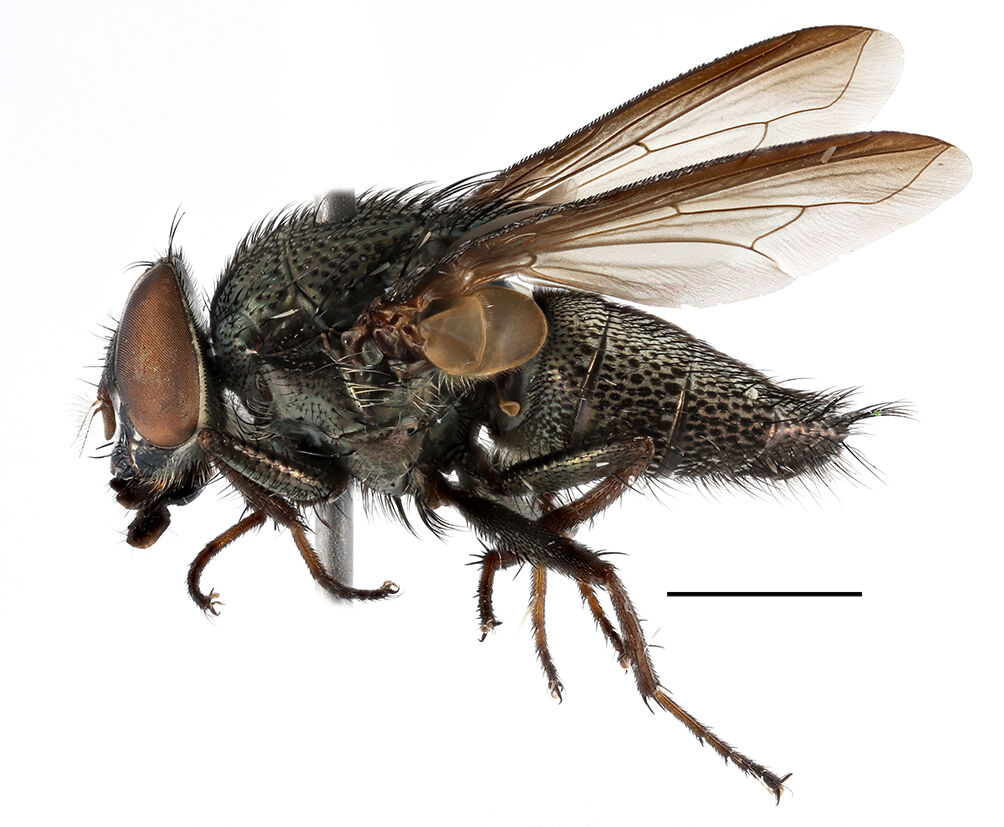
Habitus, left lateral view of *Cosminaaenea* male BMSA DIP 31129 from South Africa; scale bar = 2 mm.

**Preferred environment**: forest, open woodlands areas, woodland savannah, grasslands, coastal grassland, open mixed grassland, mixed dune woodland and bushveld near to a river (close to Nylstroom in Limpopo Province). In Namibia, the species was associated with the Kwanso River floodplain, in Kenya to Ngorowa Gorge/Stream and Lukenya cliffs/bushveld and in Malawi, to the *Acacia* woodland. **Recorded elevations**: 24–1219 m a.s.l. **Seasonality**: highest abundance in November and March and lowest between April and October (absent in July). **Behaviour and ecology**: unknown. **Life cycle and developmental stages**: unknown. **Collection methods**: sweeping net and Malaise trap. Hand net in Mozambique and Malaise trap in Namibia. **Illustrations and photographs**: male habitus as in Fig. 28. Male terminalia as in fig. 20 in Zumpt (1958).

**Material examined**: Suppl. materials 1, 2.

#### Cosmina fuscipennis

Robineau-Desvoidy, 1830

63C97C22-17C9-53C8-9D4E-EA6D7C110601

= Cosmina fuscipennis Robineau-Desvoide, 1830: 423. **Type locality**: Cap de le bonne-Espérance [Western Cape, South Africa]. **Remarks**: type-series in MNHN, destroyed, not in remnants of Robineau-Desvoidy's collection.
= Musca punctulata Wiedemann, 1819: 21. **Type locality**: Cape of Good Hope [Western Cape, South Africa]. **Remarks**: type-series in ZMUC.
= Cosmina cuprina Bigot, 1860: 539. **Type locality**: Madagascar.
= Cosmina aethiopissa Séguy, 1958: 176. **Type locality**: Kenya.

##### Distribution

**Figure 29.**
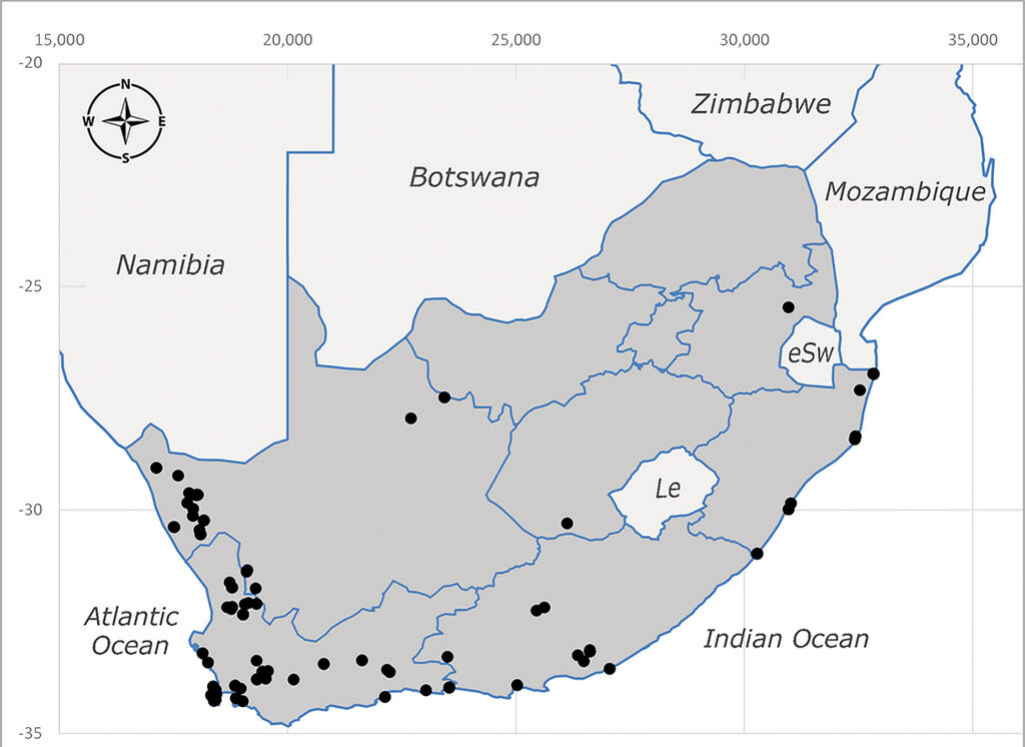
*Cosminafuscipennis* occurrence map in South Africa, including eSwatini (eSw) and Lesotho (Le); horizontal axis longitude east and vertical axis latitude south.

**Afrotropical**: Botswana, ?Kenya, Madagascar, Mozambique, Namibia, Oman, Seychelles, South Africa (Fig. 29), Tanzania and Zimbabwe. **Palaearctic**: Saudi Arabia.

##### Notes

**Figure 30.**
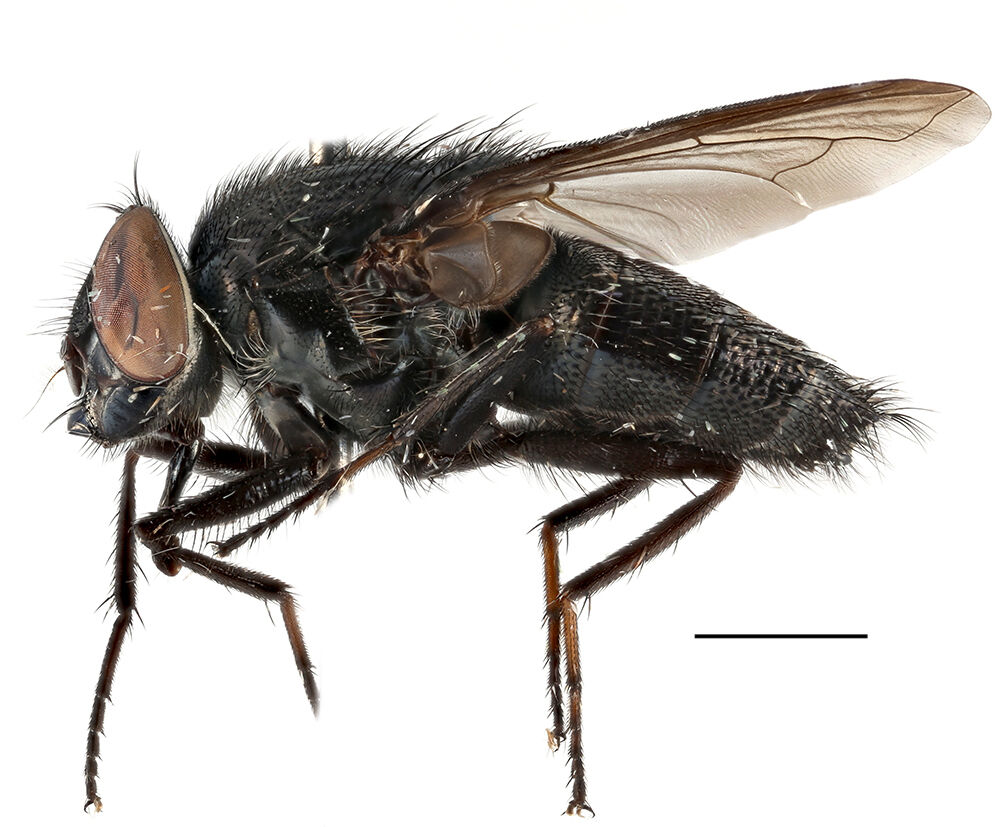
Habitus, left lateral view of *Cosminafuscipennis* male BMSA DIP 05023 from South Africa; scale bar = 2 mm.

**Preferred environment**: Indigenous dune forest; bushveld and dune vegetation forest; open woodland areas, sandy woodland savannah areas; Karoo and valley *Acacia* woodland, succulent Karoo garden on hot N slope, *Acacia* and *Ziziphus* Mill. veld; Worcester Macchia, coastal Macchia and sandy area; Fynbos, mesic mountain Fynbos on sandstone; rocky slopes at road cutting, stream edge or bushes; rocky hillside areas with vegetation or sandy areas below; rocky outcrops; open mixed grassland, riverine bush and grass, plains; mountain slope overlooking sea on vegetated cliff; and Caravan Park and surrounding area, dry stream bed near staff houses, orchards and grasslands. This species also was reported for Arid-Savannah and Succulent Karoo Biomes in Namibia (Kurahashi and Kirk-Spriggs 2006). **Recorded elevations**: 10–1350 m a.s.l. **Seasonality**: common species with highest abundance in September and October, low numbers the rest of the year and absent in April and June. In Namibia, most abundant in September (Kurahashi and Kirk-Spriggs 2006). **Behaviour and ecology**: many specimens were collected on yellow flowers of *Leucodendron* Brown sp. and *Ferrariacrispa* Burm. A group of specimens were collected in a mango orchard, with their bodies and proboscises covered with pollen (Western Cape). A female was collected on pink flowers of Mesembryanthemaceae (Northern Cape). Additionally, the species was associated with vegetation and flowers near a riverbed and a rocky gentle N slope scrub with wild flowers. A male was collected as prey of *Oxybeluslingula* (Hymenoptera). **Life cycle and developmental stages**: unknown. **Collection methods**: general sweeping and with Malaise, yellow pan and white pan traps. Hilltopping also with hand net. Eight females were collected with banana traps. In Namibia, it was collected with hand net and Malaise traps (Kurahashi and Kirk-Spriggs 2006) and in Madagascar with Malaise traps. **Illustrations and photographs**: male habitus as in Fig. 30. Male terminalia as in fig. 24 in Zumpt (1958).

**Type material examined**: *M.punctulata*: 1 ? // HT Cape Good Hope (South Africa) / June 1817 // Mus. Westerm // [ZMUC 00025139]; 1 ? // Type // Mus. Westerm // [ZMUC 00025140].

**Material examined**: Suppl. materials 1, 2.

#### Cosmina gracilis

Curran, 1927

8F7C51D6-0B80-5744-AB7D-174FB85A8E1D

= Cosmina gracilis Curran, 1927: 2. **Type locality**: South Africa, Barberton. **Remarks**: type-serie in SANC at ARC.

##### Distribution

**Figure 31.**
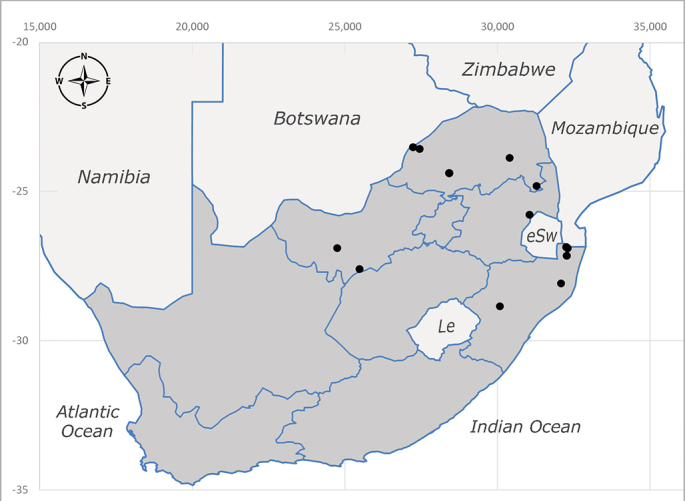
*Cosminagracilis* occurrence map in South Africa, including eSwatini (eSw) and Lesotho (Le); horizontal axis longitude east and vertical axis latitude south.

**Afrotropical**: Angola, Botswana, Mozambique, Namibia, South Africa (Fig. 31) and Zimbabwe.

##### Notes

**Figure 32.**
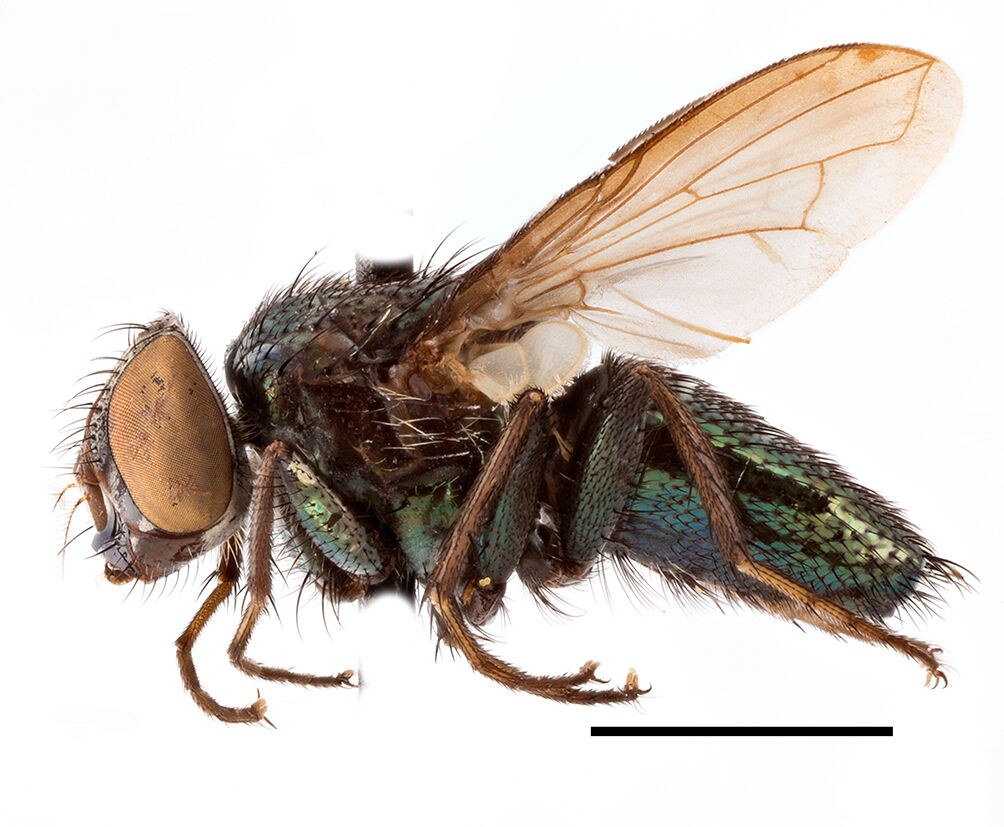
Habitus, left lateral view of *Cosminagracilis* male SANC from South Africa; scale bar = 2 mm.

**Preferred environment**: dry scrub forest, mixed bushveld-grass, sand and broad-leafed deciduous forest and *Rhus* and *Acacia* savannah. In Namibia, associated with the Mesic Savannah, Arid Savannah, Karoo and Desert Biomes (Kurahashi and Kirk-Spriggs 2006). We included new records in indigenous and degraded sand forest and cultivated plots for Namibia. **Recorded elevations**: 98–1240 m a.s.l. **Seasonality**: common species, highest abundance in September and November. Absent in February, June, July and August and low numbers in the remaining months. In Namibia, it was an abundant species, peaking from December to February (Kurahashi and Kirk-Spriggs 2006). **Behaviour and ecology**: in Namibia, was frequently observed feeding on flowers (Solanaceae and other families). The success of the pitfall-traps suggests ground-dwelling habit in adults (Kurahashi and Kirk-Spriggs 2006). **Life cycle and developmental stages**: unknown. **Collection methods**: Malaise traps and light trap. In Nambia, it was collected by sweeping or with UV-light, yellow and blue pan traps, hanging traps baited with fermenting fruit, Malaise and pitfall traps (Kurahashi and Kirk-Spriggs 2006). **Illustrations and photographs**: male habitus as in Fig. 32. Male terminalia as in fig. 26c in Zumpt (1958).

**Type material examined**: *C.gracilis*: 1 ? HT 1 ? AT // Barberton / May 17 1914 / (H. K. Munro).

**Material examined**: Suppl. materials 1, 2.

#### Cosmina margaritae

Peris, 1952

44335C1E-8313-5935-8588-B631D85E47C9

= Cosmina margaritae Peris, 1952a: 229. **Type locality**: Nyasaland [Malawi], Cholo. **Remarks**: type-series in NHMUK.

##### Distribution

**Figure 33.**
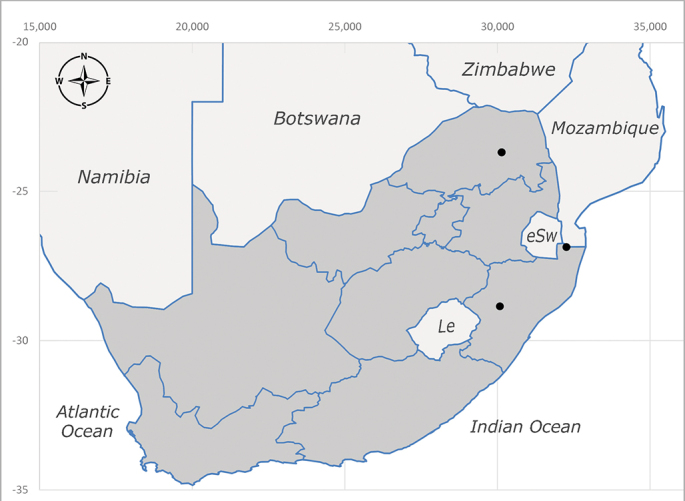
*Cosminamargaritae* occurrence map in South Africa, including eSwatini (eSw) and Lesotho (Le); horizontal axis longitude east and vertical axis latitude south.

**Afrotropical**: ?Botswana, Democratic Republic of Congo, ?Kenya, Malawi, Mozambique*, Namibia, ?Senegal, South Africa (Fig. 33), Tanzania and Zimbabwe.

##### Notes

**Figure 34.**
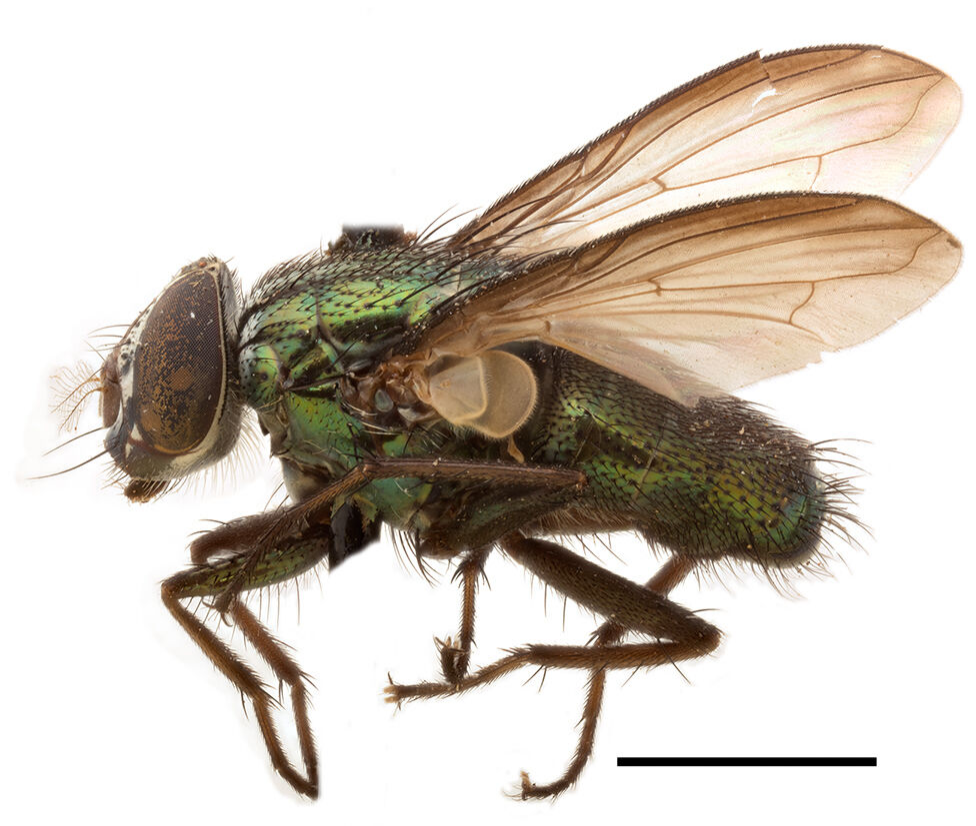
Habitus, left lateral view of *Cosminamargaritae* male NHMUK 010579920 (Copyright NHMUK); scale bar = 2 mm.

**Preferred environment**: Arid and Mesic Savannah Biome in Namibia (Kurahashi and Kirk-Spriggs 2006). **Recorded elevations**: no data. **Seasonality**: low abundance, only present in December. Abundant in Namibia with highest numbers in April (Kurahashi and Kirk-Spriggs 2006). **Behaviour and ecology**: unknown. **Life cycle and developmental stages**: unknown. **Collection methods**: yellow and blue pan traps, pitfall and Malaise traps in Namibia (Kurahashi and Kirk-Spriggs 2006) and in Mozambique, only with Malaise traps. **Illustrations and photographs**: male habitus as in Fig. 34. Male terminalia as in figs. 26a, b in Zumpt (1958).

**Type material examined**: *C.margaritae*: 1 ? Holotype // Nyasaland / Cholo. / R. C. Wood // Pres. by / Com. Inst. Ent. / BM. 1950-323 // Cosmina / margaritae / tipo n. sp. / Dr. S-V. Peris det. // [NHNUK 010579920]. 1 ? // Paratype // Nyasaland / Cholo. / R. C. Wood // Pres. by / Com. Inst. Ent. / BM. 1950-323 // Cosmina / margaritae / paratipo n. sp. / Dr. S-V. Peris det. // [NHNUK 010579921].

**Material examined**: Suppl. materials 1, 2.

#### Cosmina thabaniella

Lehrer, 2010

D952C5FA-14C5-58D7-92C0-B96F9F6BDD14

= Cosmina thabaniella Lehrer, 2010: 26. **Type locality**: South Africa, Natal [KwaZulu-Natal], Uvongo, South Coast.

##### Distribution

**Afrotropical**: South Africa.

##### Notes

No specimens examined for South Africa, based on Lehrer (2010). **Illustrations and photography**: male terminalia as in fig. 75 in Lehrer (2011).

#### Cosmina undulata

Malloch, 1926

5BA3B2EF-C46E-59D7-88EC-D2650990A97B

= Cosmina undulata : Malloch, 1926: 518. **Type locality**: S. Nigeria [Nigeria], Ibadan. **Remarks**: HT in NHMUK.

##### Distribution

**Figure 35.**
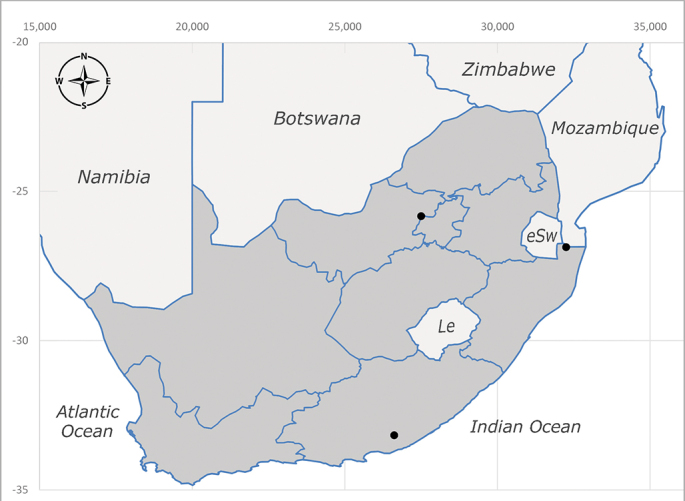
*Cosminaundulata* occurrence map in South Africa, including eSwatini (eSw) and Lesotho (Le); horizontal axis longitude east and vertical axis latitude south.

**Afrotropical**: ?Benin, Botswana, Burkina Faso, Cameroon, Côte d'Ivoire, Democratic Republic of Congo, Eritrea*, Ethiopia*, Malawi, Namibia, ?Niger, Nigeria, South Africa* (Fig. 35) and Togo.

##### Notes

**Figure 36.**
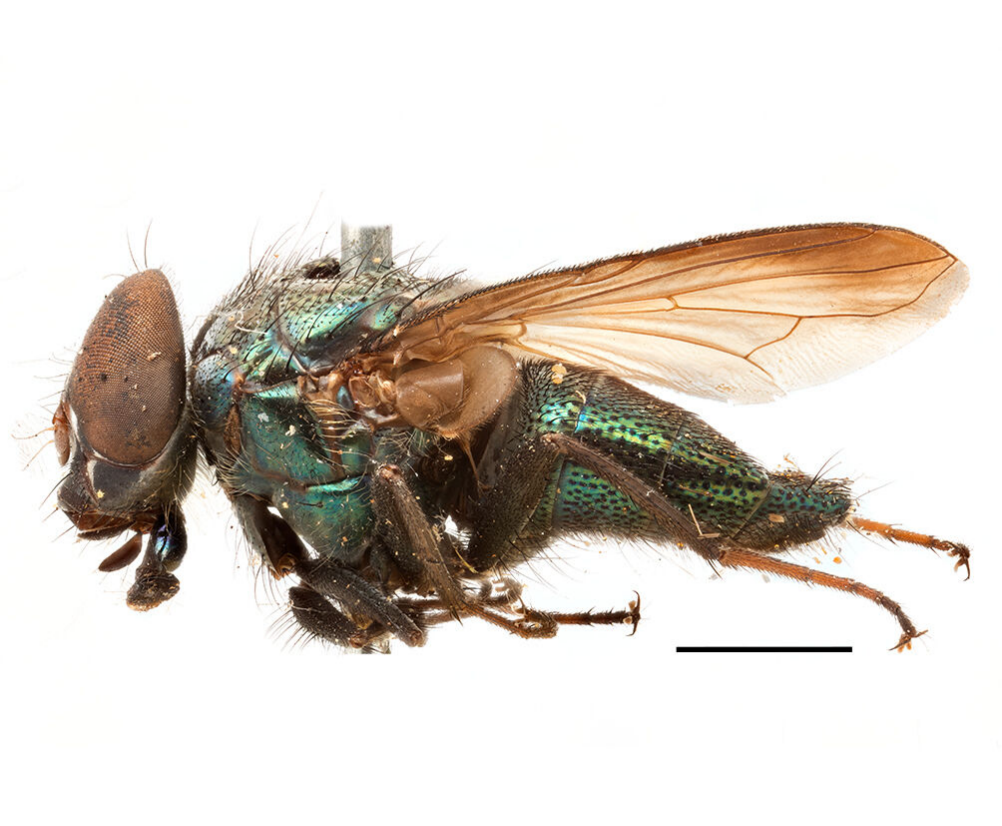
Habitus, left lateral view of *Cosminaundulata* male NHMUK 010579923 HT from Nigeria (Copyright NHMUK); scale bar = 2 mm.

**Preferred environment**: Mesic Savannah Biome in Nambia (Kurahashi and Kirk-Spriggs 2006). **Recorded elevations**: no data. **Seasonality**: uncommon species present in October, December and January (one specimen each). In Namibia, also uncommon, present in November and December (one specimen each) (Kurahashi and Kirk-Spriggs 2006). **Behaviour and ecology**: unknown. **Life cycle and developmental stages**: unknown. **Collection methods**: Malaise traps in Namibia (Kurahashi and Kirk-Spriggs 2006). **Illustrations and photographs**: male habitus as in Fig. 36. Male terminalia as in fig. 25 in Zumpt (1958).

**Type material examined**: *C.undulata*: 1 ? // Holo-type // Nigeria: / Ibadan. / 2.viii.1923. / A.W.J. Pomeroy // Pres. by / Imp. Bur. Ent. / Brit. Mus. / 192x-94 // *Cosmina* / *undulata* / Type / Det / J.R. Malloch // [NHMUK 010579923].

**Material examined**: Suppl. materials 1, 2.

#### Eurhyncomyia

Malloch, 1926

2AE0E02B-956C-52BE-A340-94577CD21989

= Eurhyncomyia Malloch, 1926: 513. **Type species**: *Xystaobtusa* Bigot, 1891 = *Eurhyncomyiadiversicolor* (Bigot, 1888) by original designation.

#### Eurhyncomyia diversicolor

(Bigot, 1888)

076590F4-867D-5EE2-A964-4E5338C1B4CE

= Rhyncomya diversicolor Bigot, 1888: 595. **Type locality**: Somalis [Somalia]. **Remarks**: LT in NHMUK, designated by Dear and Pont in the collection.
= Rhyncomyia bigoti Villeneuve, 1913: 155. **Type locality**: South Africa, Natal [KwaZulu-Natal], Port Natal [Durban].
= Eurhyncomyia thoracica Curran, 1931: 21. **Type locality**: South Africa, Natal [KwaZulu-Natal], Port Shepstone.

##### Distribution

**Figure 37.**
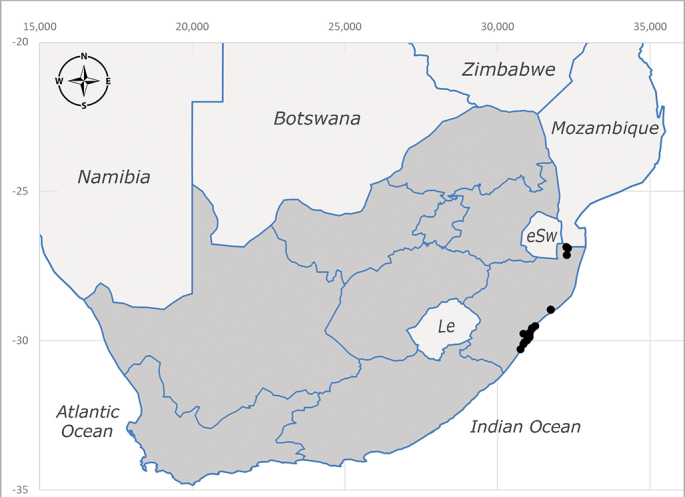
*Eurhyncomyiadiversicolor* occurrence map in South Africa, including eSwatini (eSw) and Lesotho (Le); horizontal axis longitude east and vertical axis latitude south.

**Afrotropical**: Mozambique, Namibia*, Somalia, South Africa (Fig. 37) and Tanzania (including Zanzibar Island).

##### Notes

**Figure 38.**
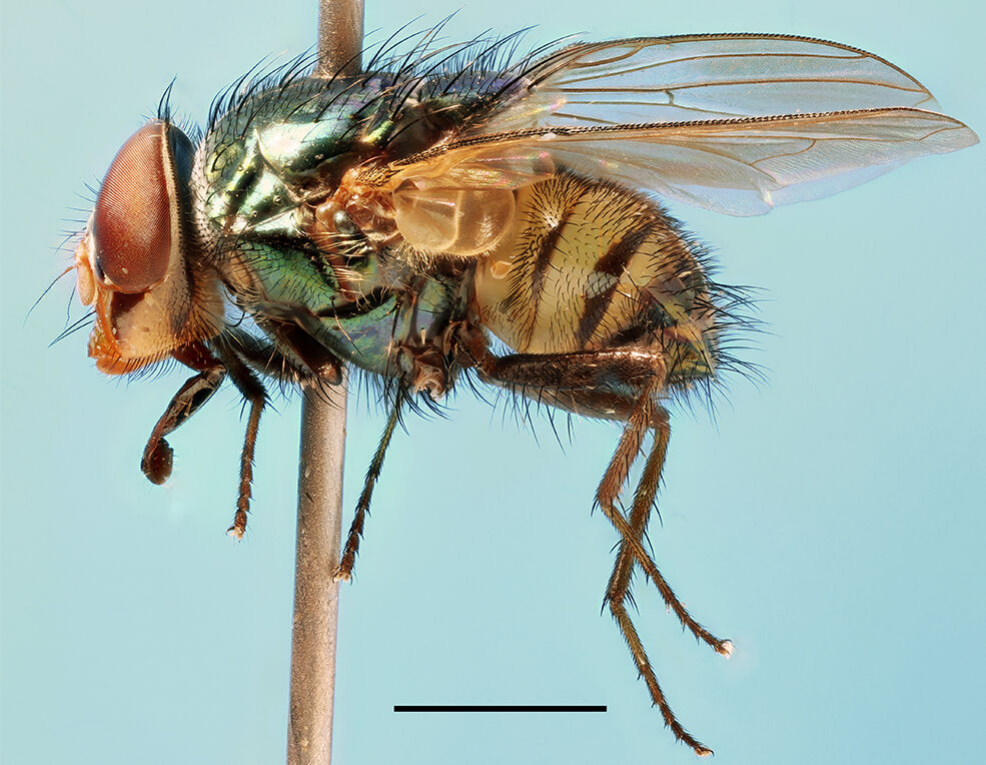
Habitus, left lateral view of *Eurhyncomyiadiversicolor* male BMSA DIP 16947 from South Africa; scale bar = 2 mm.

**Preferred environment**: broad-leafed, broad-leafed deciduous, coastal and sand forest. In Namibia, it is associated with a broken veld at the base of small hill. **Recorded elevations**: 77 m a.s.l. **Seasonality**: present almost all year-round, peaking in December, lower abundance in January, May, June and August and absent in September. **Behaviour and ecology**: unknown. **Life cycle and developmental stages**: unknown. **Collection methods**: Malaise trap. In Mozambique, Malaise trap. **Illustrations and photographs**: male habitus as in Fig. 38. Male terminalia as in fig. 39 in Zumpt (1958).

**Type material examined**: *R.diversicolor*: 1 ? // Lecto-type // Somalis // Brauer / Wien. CVIII / (No. 87) // Lectotype ? / *Rhyncomyia* / *diversicolor* / Bigot / Designated by / Dear and Pont. // BMNH (E) / #231121 // [NHMUK 010579922].

**Material examined**: Suppl. materials 1, 2.

#### Eurhyncomyia metzi

(Zumpt, 1981)

39A6728B-50F6-55AD-8AE7-3A054913158D

= Rhyncomyia metzi Zumpt, 1981: 487 (see taxonomic notes). **Type locality**: South Africa, Natal Zululand [KwaZulu-Natal], Umfalozi Game Park. **Remarks**: HT and PTs in NMSA.

##### Distribution

**Figure 39.**
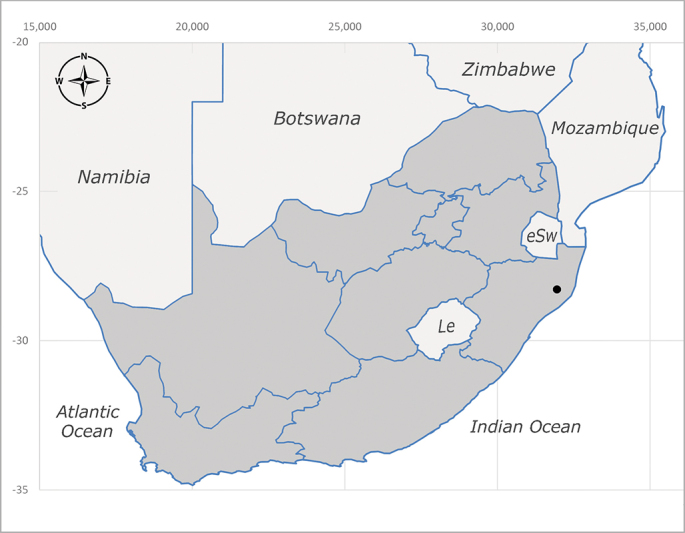
*Eurhyncomyiametzi* occurrence map in South Africa, including eSwatini (eSw) and Lesotho (Le); horizontal axis longitude east and vertical axis latitude south.

**Afrotropical**: South Africa (Fig. 39).

##### Notes

**Figure 40.**
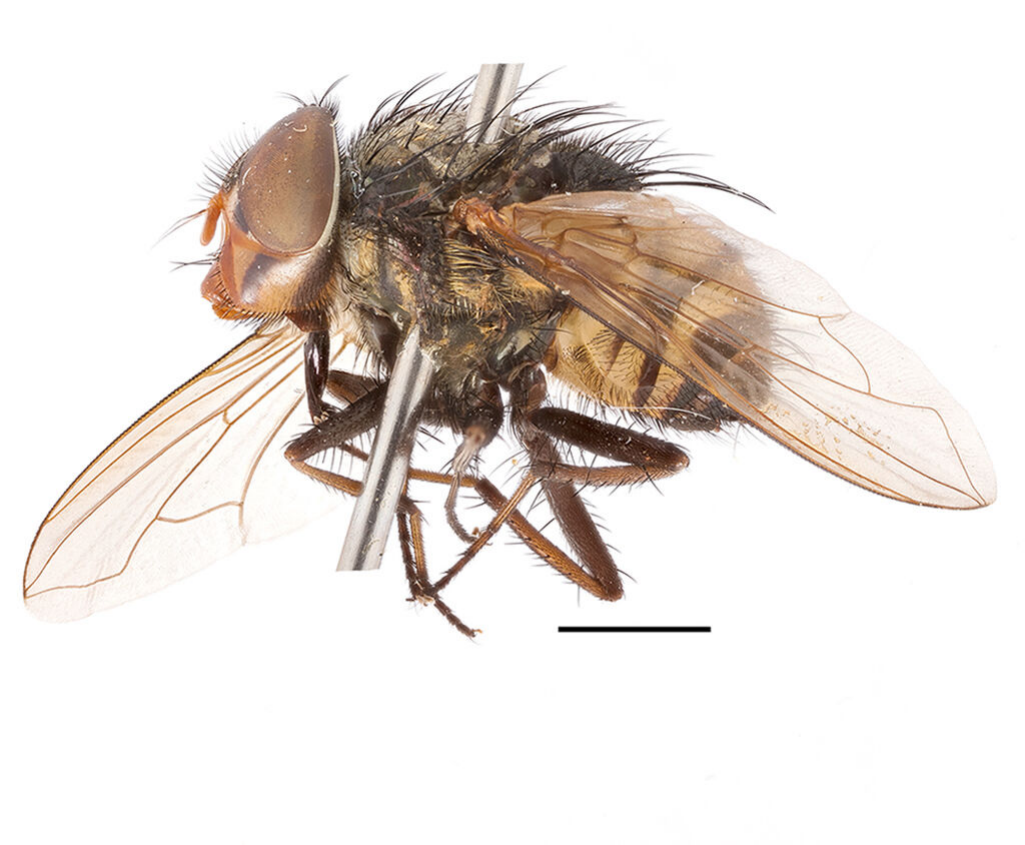
Habitus, left lateral view of *Eurhyncomyiametzi* male NMSA DIP 74954 HT from South Africa (without terminalia); scale bar = 2 mm.

**Preferred environment**: no data. **Recorded elevations**: no data. **Seasonality**: recorded only in September. **Behaviour and ecology**: unknown. **Life cycle and developmental stages**: unknown. **Collection methods**: unknown. **Illustrations and photographs**: male habitus as in Fig. 40. Male terminalia as in fig. 1 in Zumpt (1981).

**Taxonomic notes**: the HT and PTs of *R.metzi* were examined at the NMSA and their supra-squamal ridge is setulose in the posterior half, a diagnostic character that separates *Eurhyncomyia* (setulose) from *Rhyncomya* (bare). Another characteristic of *Eurhyncomyia* is that the aristal hairs are long and pubescent, the longest hairs slightly exceeding half the width of the post-pedicel, just as *R.metzi*, whereas in *Rhyncomya*, it is either bare or the hairs rarely exceed the width of the basal arista (Zumpt 1958, Kurahashi and Kirk-Spriggs 2006). Based on these morphological differences, we considered that this species belongs to *Eurhyncomyia* genus as *E.metzi* **comb**. **nov**.

**Type material examined**: *R.metzi*: 1 ? // HOLOTYPUS // SOUTH AFRICA, Natal / Zululand, Umfalozi / Game Park, 2831Bd / 21-VII-1973, ME Irwin // slide no. 30 // *Rhyncomya* ? / *metzi* Zumpt / det. Zumpt 80 // [NMSA-DIP 074954] // (N.M. Type No. 2437). 6 ?? // PARATYPE // SOUTH AFRICA, Natal / Zululand, Umfalozi / Game Park, 2831Bd / 21-VII-1973, ME Irwin // *Rhyncomya* ? / *metzi* Zumpt / det. Zumpt 80 // [NMSA-DIP [NMSA DIP 019991, 061680, 074915, 074918, 074952, 0749523] // (N.M. Type No. 2437).

**Material examined**: Suppl. material 1.

#### Isomyia

Walker, 1859

5EACCF31-9002-57CC-B0AC-81C6F2836794

= Isomyia Walker, 1859: 134. **Type species**: *Muscadelectans* Walker, 1859 by original designation and monotypy.
= Strongyloneura Bigot, 1886: xiv. **Type species**: *Strongyloneuraprasina* Bigot, 1887 (Oriental genus) by monotypy.
= Thelychaeta Brauer and Bergenstamm, 1891: 390. **Type species**: *Thelychaetachalybea* Brauer and Bergenstamm, 1891 = *Isomyiaviridaurea* (Wiedemann, 1819) by monotypy.
= Apollenia Bezzi, 1911: 79. **Type species**: *Pollenianudiuscula* Bigot, 1911 = *Phumosianudiuscula* (Bigot, 1888) [missidentification = *Curtonevratristis* Bigot, 1888] by original designation.
= Anna Malloch, 1926: 520. **Type species**: *Anna calliphoroides Malloch*, 1926 by original designation
= Gerschia Lehrer, 1970: 30. **Type species**: *Isomyiaeos* Zumpt, 1958 by original designation.

#### Isomyia cuthbertsoni

(Curran, 1938)

470D1D6C-6992-5896-9DE6-FCB68461674B

= Strongyloneura cuthbertsoni Curran, 1938: 2. **Type locality**: S. Rhodesia [Zimbabwe], Vumba Mts.

##### Distribution

**Figure 41.**
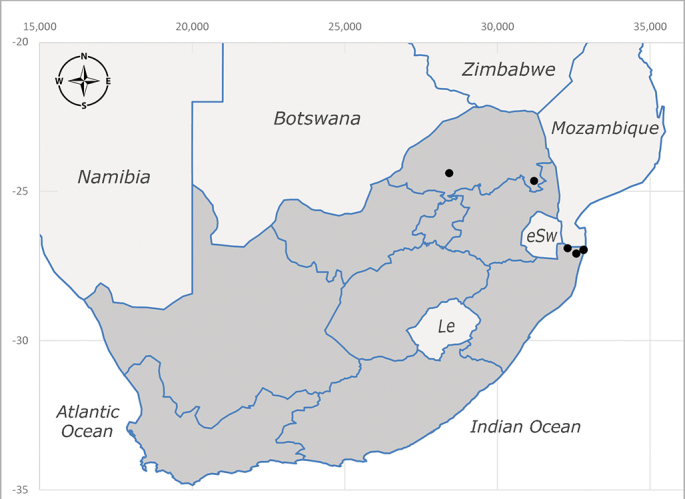
*Isomyiacuthbertsoni* occurrence map in South Africa, including eSwatini (eSw) and Lesotho (Le); horizontal axis longitude east and vertical axis latitude south.

**Afrotropical**: Zimbabwe and South Africa* (Fig. 41).

##### Notes

**Figure 42.**
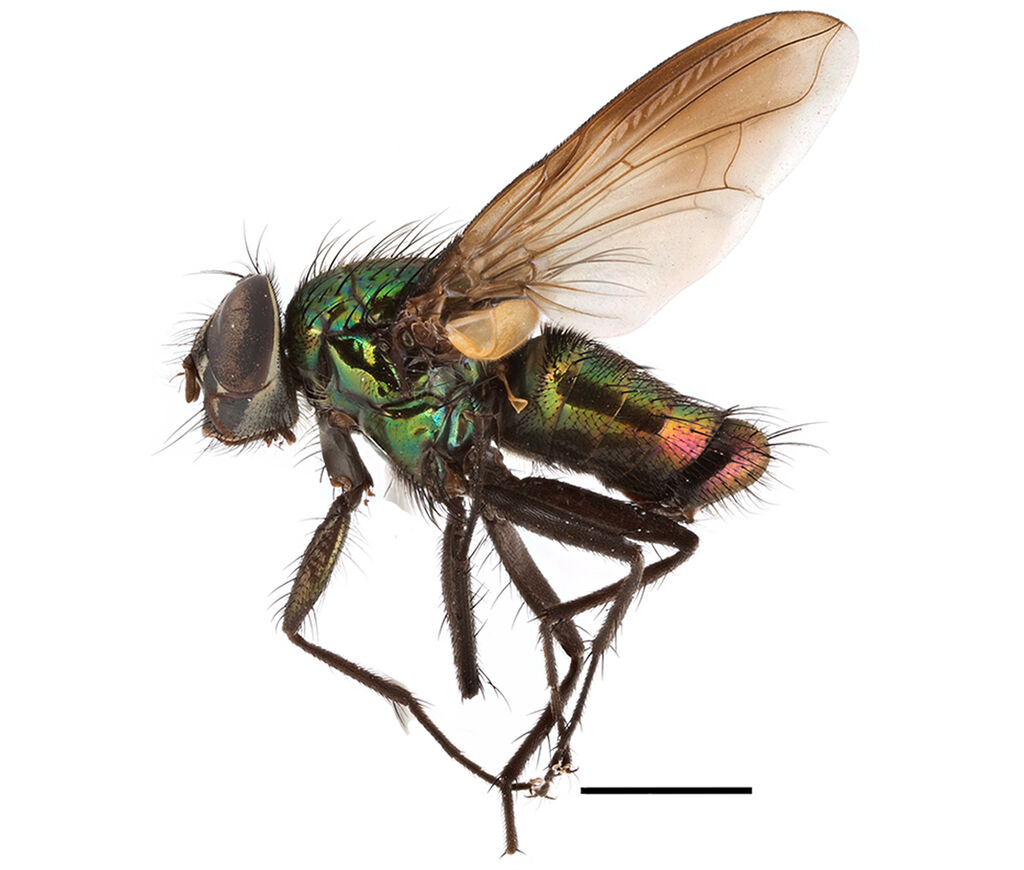
Habitus, left lateral view of *Isomyiacuthbertsoni* male SANC from South Africa; scale bar = 2 mm.

**Preferred environment**: woodland savannah, woodland, dune, sand and broad-leafed deciduous forest. **Recorded elevations**: 6–1095 m a.s.l. **Seasonality**: highest abundance in October and December, lower numbers or absent the rest of the year. **Behaviour and ecology**: specimens were swept from *Asparagus* L. sp. in Sileza Natural Reserve (KwaZulu-Natal). In Zimbabwe, Cuthbertson (1939) reported females (as *Strongyloneuracuthbertsoni*) attracted to pollen of Rosaceae. **Life cycle and developmental stages**: unknown. **Collection methods**: sweeping, hand nets and Malaise trap. **Illustrations and photographs**: male habitus as in Fig. 42. Male terminalia as in fig. 17–right in Zumpt (1958).

**Material exmained**: Suppl. material 1.

#### Isomyia darwini

(Curran, 1938)

449A2D03-CC59-566A-83B1-66634A3A444F

= Strongyloneura darwini Curran, 1938: 3. **Type locality**: S. Rhodesia [Zimbabwe].

##### Distribution

**Figure 43.**
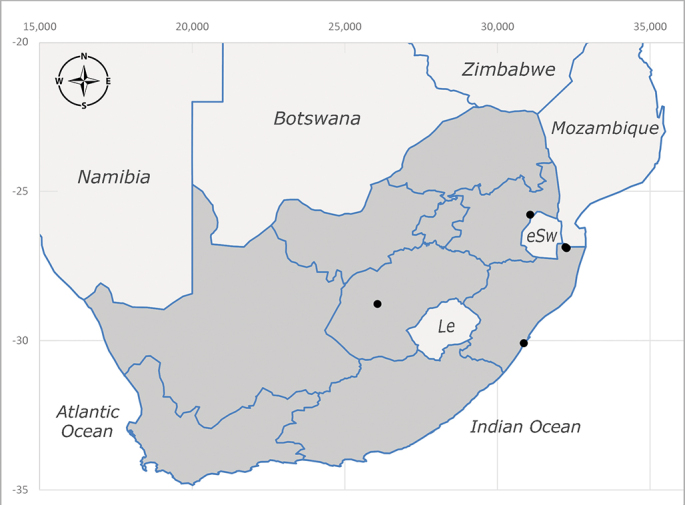
*Isomyiadarwini* occurrence map in South Africa, including eSwatini (eSw) and Lesotho (Le); horizontal axis longitude east and vertical axis latitude south.

**Afrotropical**: Botswana, ?Democratic Republic of Congo, Namibia, South Africa (Fig. 43) and Zimbabwe.

##### Notes

**Figure 44.**
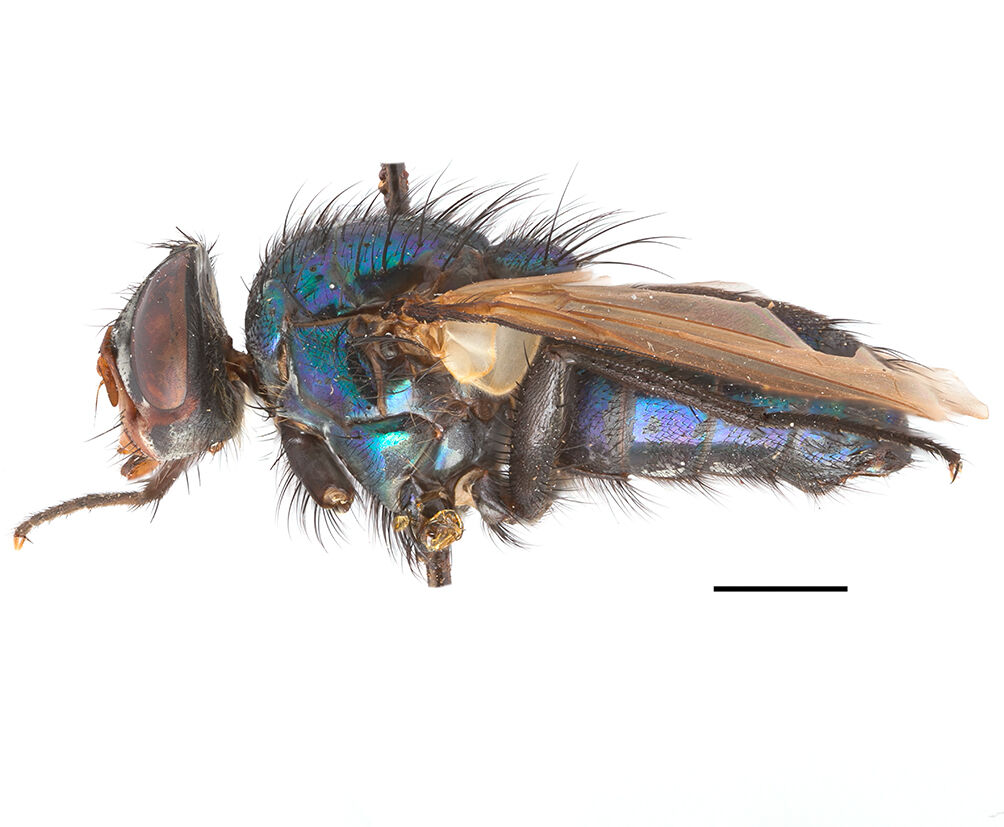
Habitus, left lateral view of *Isomyiadarwini* male NMSA DIP 19840 from South Africa (without male terminalia); scale bar = 2 mm.

**Preferred environment**: *Ficus* forest. Apparently restricted to the Arid Savannah Biome in Namibia (Kurahashi and Kirk-Spriggs 2006). **Recorded elevations**: 95 m a.s.l. **Seasonality**: maximum abundance in November (three specimens) and almost absent the rest of the year. In Namibia, it was present in low numbers (Kurahashi and Kirk-Spriggs 2006). **Behaviour and ecology**: observed at flowers in Zimbabwe by Cuthbertson (1939) (as *S.darwini*) and also collected at flowers near Darwin (Zimbabwe) in March 1933 (Curran 1938 in Kurahashi and Kirk-Spriggs 2006). **Life cycle and developmental stages**: unknown. **Collection methods**: Malaise trap. In Namibia, with UV light and with Malaise and pitfall traps (Kurahashi and Kirk-Spriggs 2006). **Illustrations and photographs**: female habitus as in Fig. 44. Male terminalia as in fig. 16 in Zumpt (1958).

**Material examined**: Suppl. materials 1, 2.

#### Isomyia deserti

(Karsch, 1888)

E9D0FDDD-193D-5E35-AE3E-BA1940B1CEFA

= Somomyia deserti Karsch, 1888: 378. **Type locality**: [Tanzania].
= Thelychaeta versipellis Villeneuve, 1917: 344. **Type locality**: Congo Belge [Democratic Republic of Congo], Kilimbi and Sankisia; Nyasaland [Malawi], Mt. Mlanje; Mozambique; South Africa. **Remarks**: PT in SAMC.

##### Distribution

**Figure 45.**
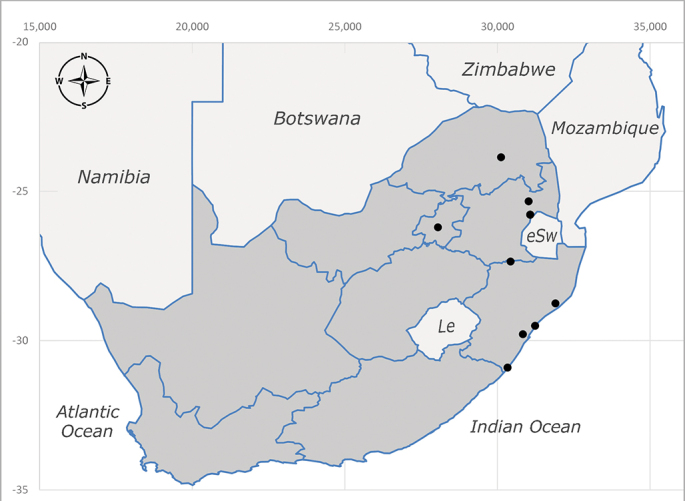
*Isomyiadeserti* occurrence map in South Africa, including eSwatini (eSw) and Lesotho (Le); horizontal axis longitude east and vertical axis latitude south.

**Afrotropical**: ?Botswana, Burundi, Democratic Republic of Congo, Malawi, Mozambique, Namibia, South Africa (Fig. 45), Tanzania and Zimbabwe.

##### Notes

**Figure 46.**
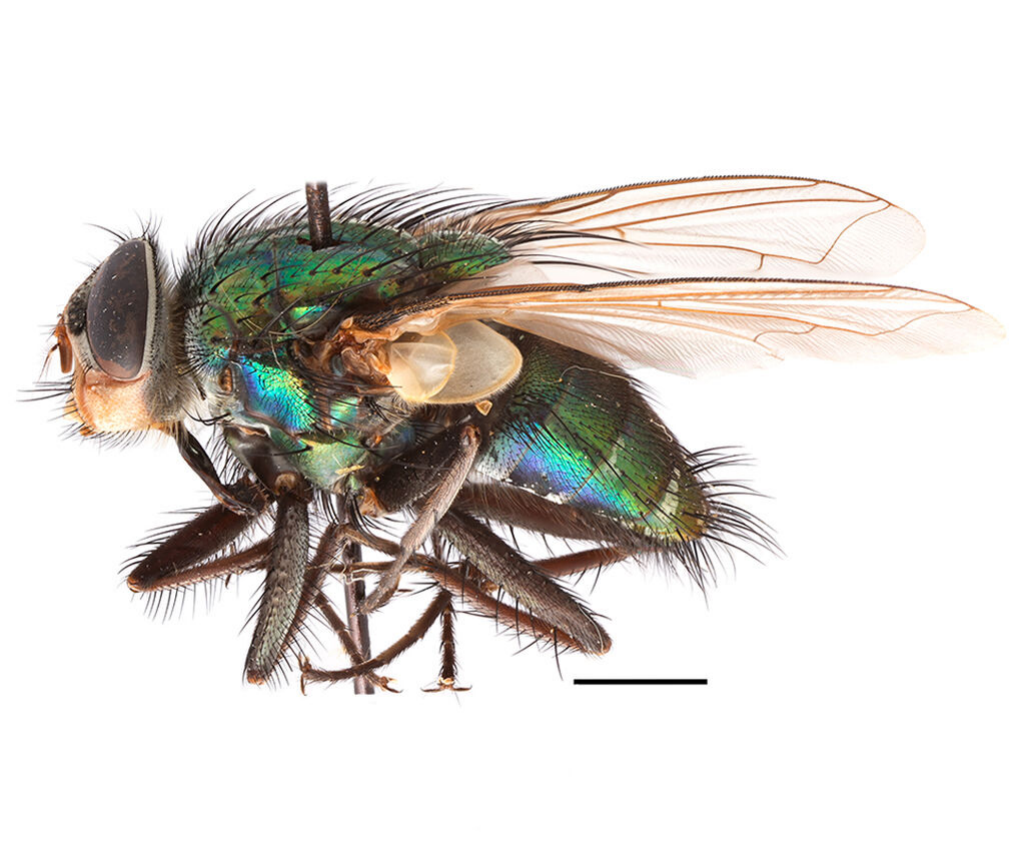
Habitus, left lateral view of *Isomyiadeserti* male NMSA DIP 61581 from South Africa; scale bar = 2 mm.

**Preferred environment**: dune vegetation and beach environment. Apparently restricted to the Arid and Mesic Savannah Biome in Namibia (Kurahashi and Kirk-Spriggs 2006). **Recorded elevations**: no data. **Seasonality**: present from December to March, May and June and absent in other months. **Behaviour and ecology**: collected on flowers in Zimbabwe. **Life cycle and developmental stages**: unknown. **Collection methods**: In Namibia, collected by hand net and Malaise and yellow pans traps (Kurahashi and Kirk-Spriggs 2006). **Illustrations and photographs**: male habitus as in Fig. 46. Male terminalia as in fig. 16 in Zumpt (1958).

**Type material examined**: *T.versipellis*: 1? / Marley / Kloof Natal/ 2-1915 // 359 // *Thelychaeta* / *versipellis* Ville. // Paratype // [SAMC DIP A011140]. **Remarks**: with head glued on a card.

**Material examined**: Suppl. materials 1, 2.

#### Isomyia distinguenda

(Villeneuve, 1917)

2249FFF7-3513-5945-8EB8-41274D22C16D

= Thelychaeta distinguenda Villeneuve, 1917: 352. **Type locality**: Congo Belge [Democratic Republic of Congo], Elisabethville [Lubumbashi] and Kundelungu; l'Afrique Orientale anglaise [Kenya], Nairobi.

##### Distribution

**Figure 47.**
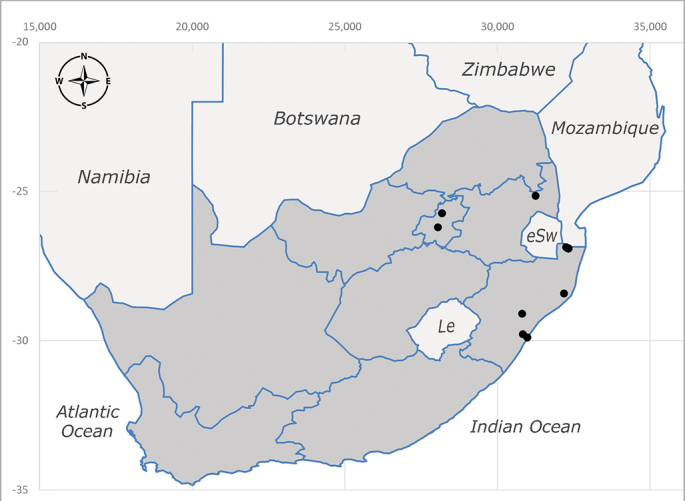
*Isomyiadistinguenda* occurrence map in South Africa, including eSwatini (eSw) and Lesotho (Le); horizontal axis longitude east and vertical axis latitude south.

**Afrotropical**: Burundi Democratic Republic of Congo, Kenya, Mozambique*, South Africa (Fig. 47), Togo*, Uganda and Zimbabwe.

##### Notes

**Figure 48.**
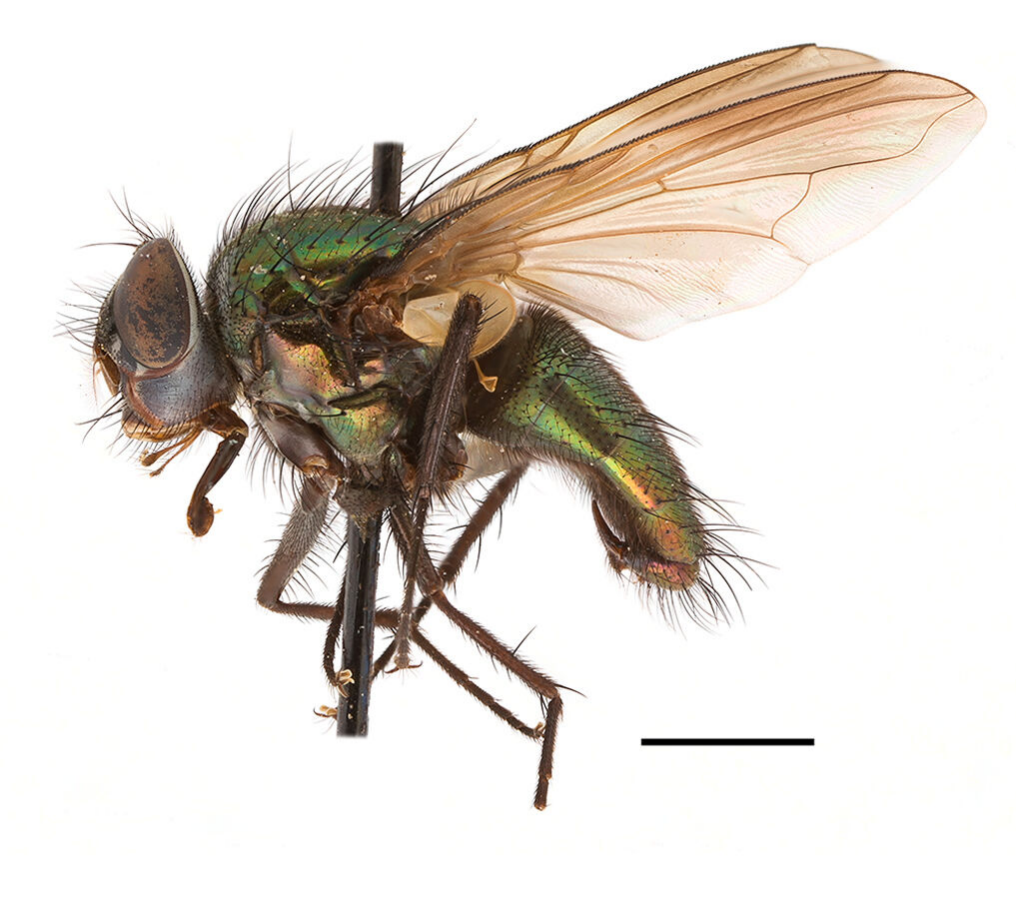
Habitus, left lateral view of *Isomyiadistinguenda* male BMSA DIP 13696 from South Africa; scale bar = 2 mm.

**Preferred environment**: sand, forest and broad-leafed deciduous forest and woodland. Vegetated stream-bed in Togo. **Recorded elevations**: no data. **Seasonality**: highest abundance between November and January, rest of the year generally absent or only one specimen per month. **Behaviour and ecology**: unknown. **Life cycle and developmental stages**: unknown. **Collection methods**: Malaise traps. In Togo, Malaise trap. **Illustrations and photographs**: male habitus as in Fig. 48. Male terminalia as in fig. 15 in Zumpt (1958).

**Material examined**: Suppl. materials 1, 2.

#### Isomyia dubiosa

(Villeneuve, 1917)

4521E7C9-20FA-545E-95DF-094EDD725A84

= Idiopsis buccata Bezzi, 1911: 73. **Type locality**: South Africa, Pretoria.
= Thelychaeta dubiosa Villeneuve, 1917: 350. **Type locality**: Belgian Congo [Democratic Republic of Congo], Urwald Moera; British East Africa [Kenya], Wa-Taita Boura District.
= Thelychaeta claripennis Villeneuve, 1917: 350. **Type locality**: Nyasaland [Malawi]; Southern Rhodesia [Zimbabwe], Salisbury [Harare].
= Strongyloneura sheppardi Curran, 1938: 3. **Type locality**: Southern Rhodesia [Zimbabwe], Balla-Balla.
= Apollenia nasica Séguy, 1949: 131. **Type locality**: Kenya, Nairobi.
= Apollenia promula Séguy, 1949: 133. **Type locality**: Guinea, Nimba, Keoulenta.

##### Distribution

**Figure 49.**
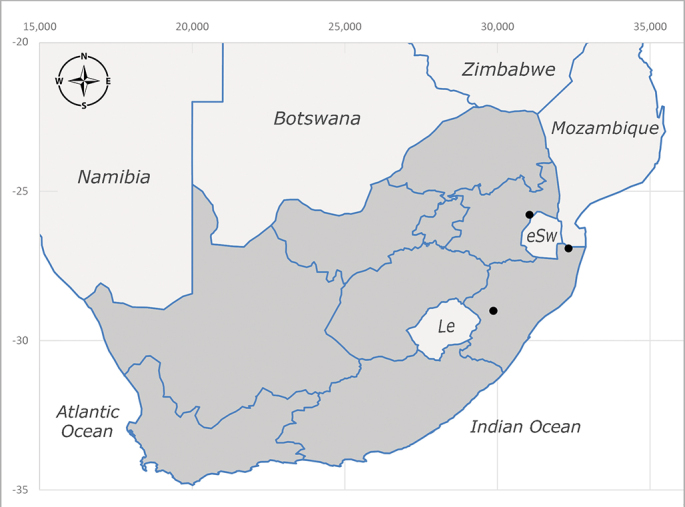
*Isomyiadubiosa* occurrence map in South Africa, including eSwatini (eSw) and Lesotho (Le); horizontal axis longitude east and vertical axis latitude south.

**Afrotropical**: Cameroon, Democratic Republic of Congo, Equatorial Guinea, Ghana, Guinea, Côte d’Ivoire, Kenya, Liberia, Malawi, Namibia, Nigeria, Rwanda, Senegal, Sierra Leone, South Africa (Fig. 49), Tanzania, Togo, Uganda and Zimbabwe.

##### Notes

**Figure 50.**
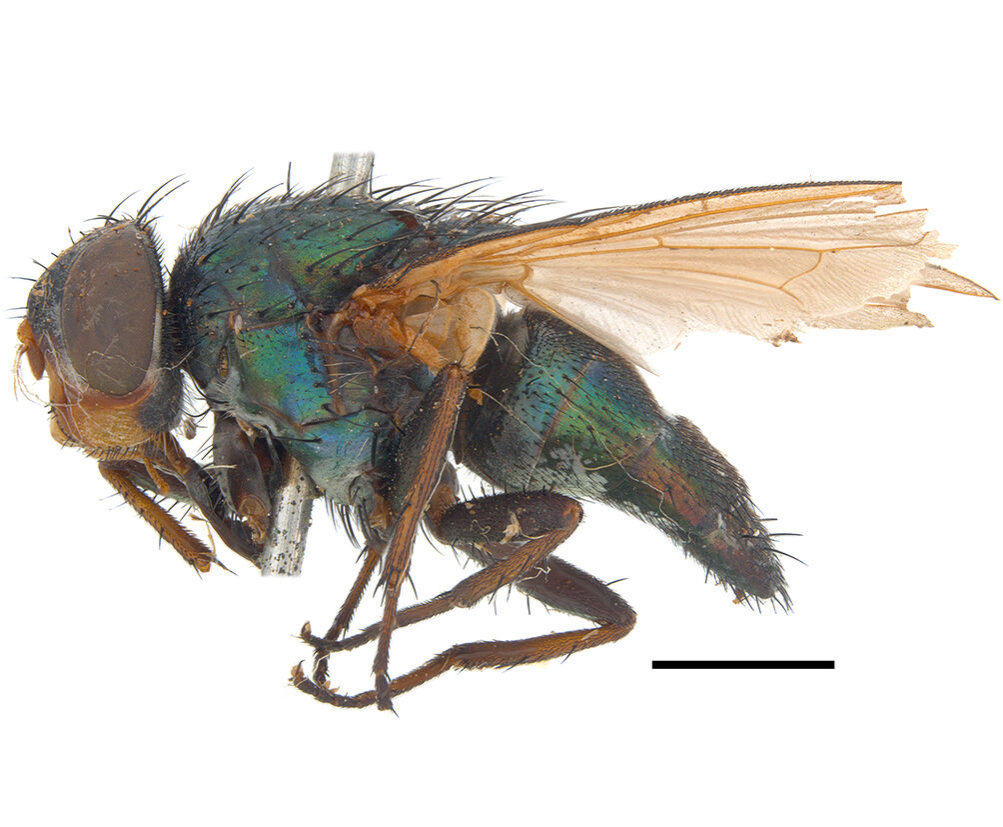
Habitus, left lateral view of *Isomyiadubiosa* female SAMC DIP A011143 from South Africa; scale bar = 2 mm.

**Preferred environment**: broad-leafed deciduous woodland. In Democratic Republic of Congo associated with bush paths and village environments; and in Togo, with remnant forest patches and vegetated stream-beds. Cuthbertson (1939) reported *I.dubiosa* (as *Strongyloneurasheppardi*) at the blossoms of a wild shrub during March and April near Balla Balla (now Mbalabala), Zimbabwe. **Recorded elevations**: no data. **Seasonality**: low numbers between October and December. In Zimbabwe, collected during March and April (Cuthbertson 1939). **Behaviour and ecology**: unknown. **Life cycle and developmental stages**: unknown. **Collection methods**: "MV" and black light traps. In Togo and the Democratic Republic of Congo, with Malaise traps. **Illustrations and photographs**: female habitus as in Fig. 50. Male terminalia as in fig. 7 in Zumpt (1958).

**Material examined**: Suppl. materials 1, 2.

#### Isomyia eos

Zumpt, 1958

468C5ED3-115C-5DB4-A5E4-31AA4AA4FB51

= Isomyia eos Zumpt, 1958: 45. **Type locality** Southern Rhodesia [Zimbabwe], Farfell Farm, Melsetter District. **Remarks**: PT in NMSA.

##### Distribution

**Figure 51.**
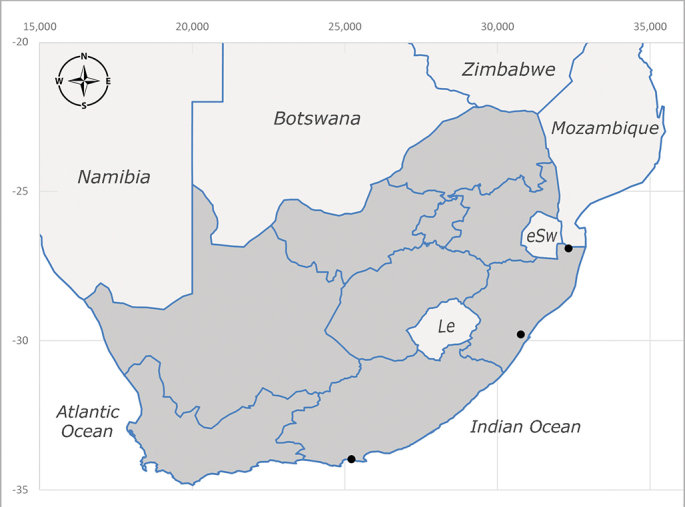
*Isomyiaeos* occurrence map in South Africa, including eSwatini (eSw) and Lesotho (Le); horizontal axis longitude east and vertical axis latitude south.

**Afrotropical**: Botswana, Burundi, Democratic Republic of Congo, Kenya, South Africa (Fig. 51) and Zimbabwe.

##### Notes

**Figure 52.**
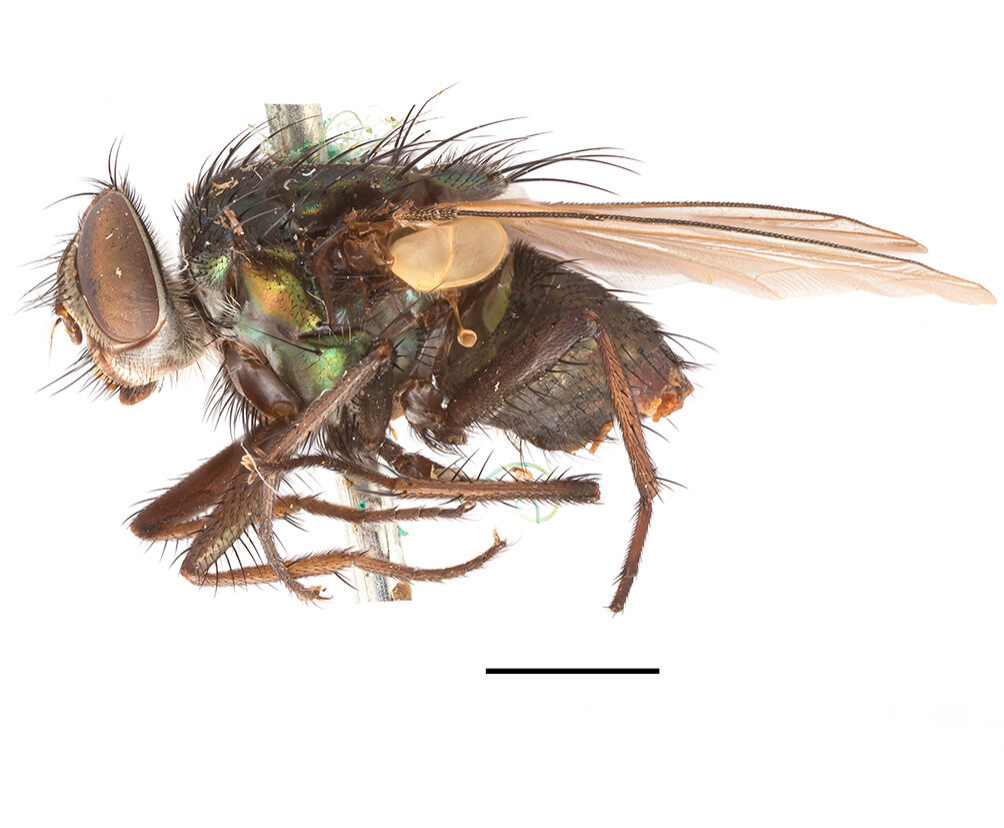
Habitus, left lateral view of *Isomyiaeos* male NMSA DIP 19909 PT from Zimbabwe (male without terminalia and partly broken tergite 5); scale bar = 2 mm.

**Preferred environment**: broad-leafed deciduous woodland. **Recorded elevations**: no data. **Seasonality**: low numbers during March, November and December. **Behaviour and ecology**: unknown. **Life cycle and developmental stages**: unknown. **Collection methods**: Malaise trap. **Illustrations and photographs**: male habitus as in Fig. 52. Male terminalia as in fig. 10 in Zumpt (1958).

**Type material examined**: *I.eos*: 1 ? // Grampirks / Inyamadzi River / Melsetter District / Dept. Agric. S. Rhodesia / 3/6/1939 // slide no 11 // Paratype // *Isomyia* / *eos* n.sp. / Zumpt 1956 // [NMSA DIP 019909].

**Material examined**: Suppl. materials 1, 2.

#### Isomyia innia

Lehrer, 2009

7B156A08-93B9-5017-85CD-A08FBE8CE308

= Isomyia innia Lehrer, 2009: 21. **Type locality**: South Africa, Inn on Robber's Pass, 1,400 m.

##### Distribution

**Afrotropical**: South Africa.

##### Notes

No specimens examined for South Africa, based on Lehrer (2009). **Illustrations and photographs**: male terminalia as in fig. 89 in Lehrer (2011).

#### Isomyia longicauda

(Villeneuve, 1917)

D590C67C-AD18-502A-B7C7-168E1522CA8F

= Thelychaeta longicauda Villeneuve, 1917: 350. **Type locality**: South Africa, East London [Eastern Cape]; N. W. Tanganika [Decomocratic Republic of Congo]. **Remarks**: PT in SAMC.

##### Distribution

**Figure 53.**
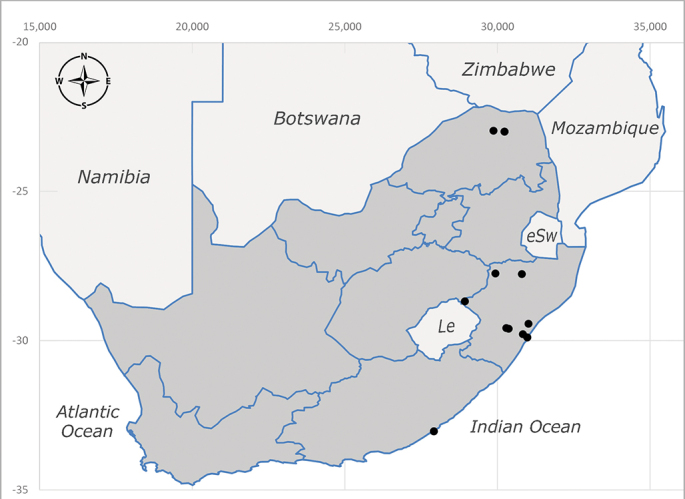
*Isomyialongicauda* occurrence map in South Africa, including eSwatini (eSw) and Lesotho (Le); horizontal axis longitude east and vertical axis latitude south.

**Afrotropical**: Burundi, Democratic Republic of Congo, Kenya, Malawi, South Africa (Fig. 53), ?Sudan (as Anglo-Egyptian Sudan), Tanzania, Uganda and Zimbabwe.

##### Notes

**Figure 54.**
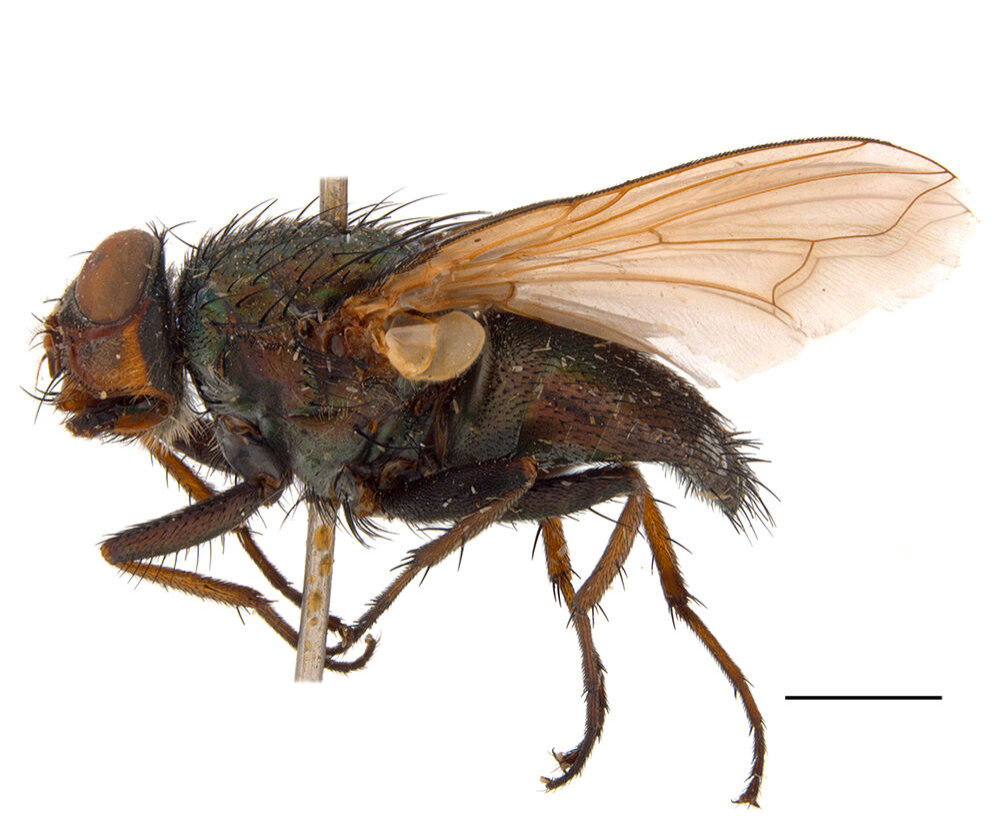
Habitus, left lateral view of *Isomyialongicauda* female SAMC DIP A011144 PT from South Africa; scale bar = 2 mm.

**Preferred environment**: savannah/grassland, mist-belt mixed forest, grass and forest edges and river stream. In Malawi, in grassland and forest edges. **Recorded elevations**: 1100–1350 m a.s.l. In Burundi, between 1800 and 2000 m a.s.l. (Peris 1952b). **Seasonality**: recorded in low numbers, with highest abundance (three specimens) in January and absent in February, April, May and October. **Behaviour and ecology**: unknown. **Life cycle and developmental stages**: unknown. **Collection methods**: Malaise and pan traps. **Illustrations and photographs**: female habitus as in Fig. 54. Male terminalia as in fig. 18 in Zumpt (1958).

**Type material examined**: *T.longicauda*: 1 ? // Paratype // E. London / Lightfoot / July 1914 // S.A.M // *Thelychaeta* / *longicauda* / Det. Villeneuve // [SAMC DIP A011144].

**Material examined**: Suppl. materials 1, 2.

#### Isomyia natalensis

(Villeneuve, 1917)

893FEC18-3FDF-58FA-8796-2C59E950D3E0

= Thelychaeta natalensis Villeneuve, 1917: 347. **Type locality**: South Africa, Natal [KwaZulu-Natal].

##### Distribution

**Figure 55.**
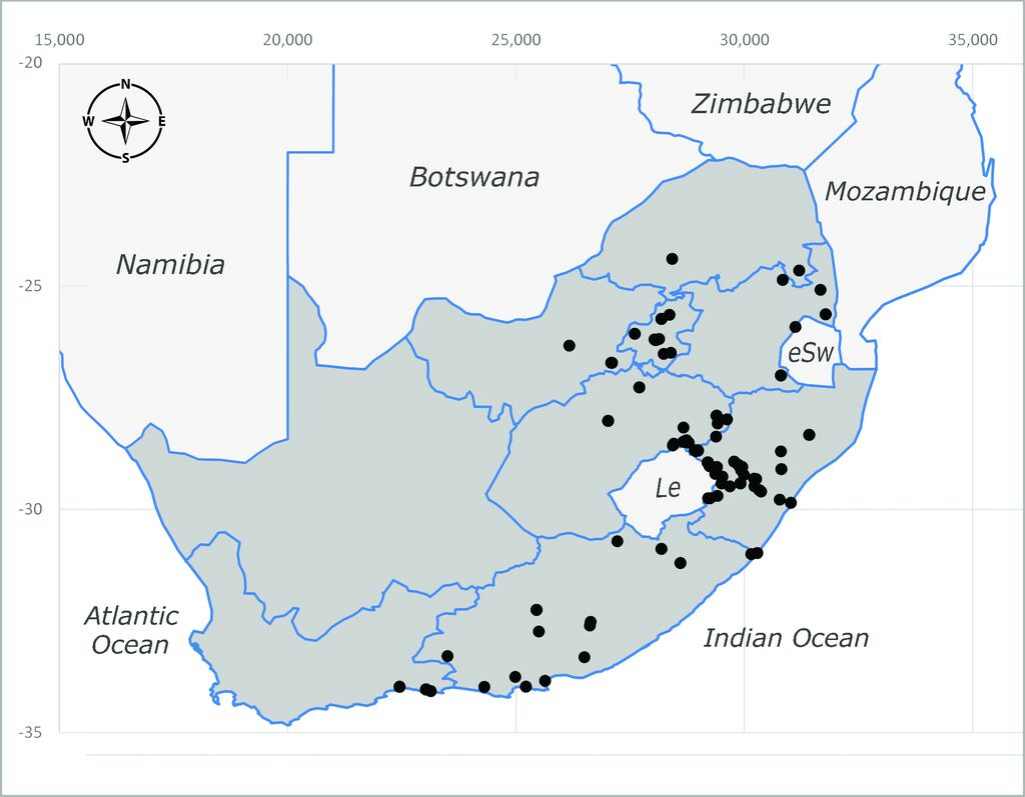
*Isomyianatalensis* occurrence map in South Africa, including eSwatini (eSw) and Lesotho (Le); horizontal axis longitude east and vertical axis latitude south.

**Afrotropical**: Lesotho, Namibia, South Africa (Fig. 55) and Zimbabwe.

##### Notes

**Figure 56.**
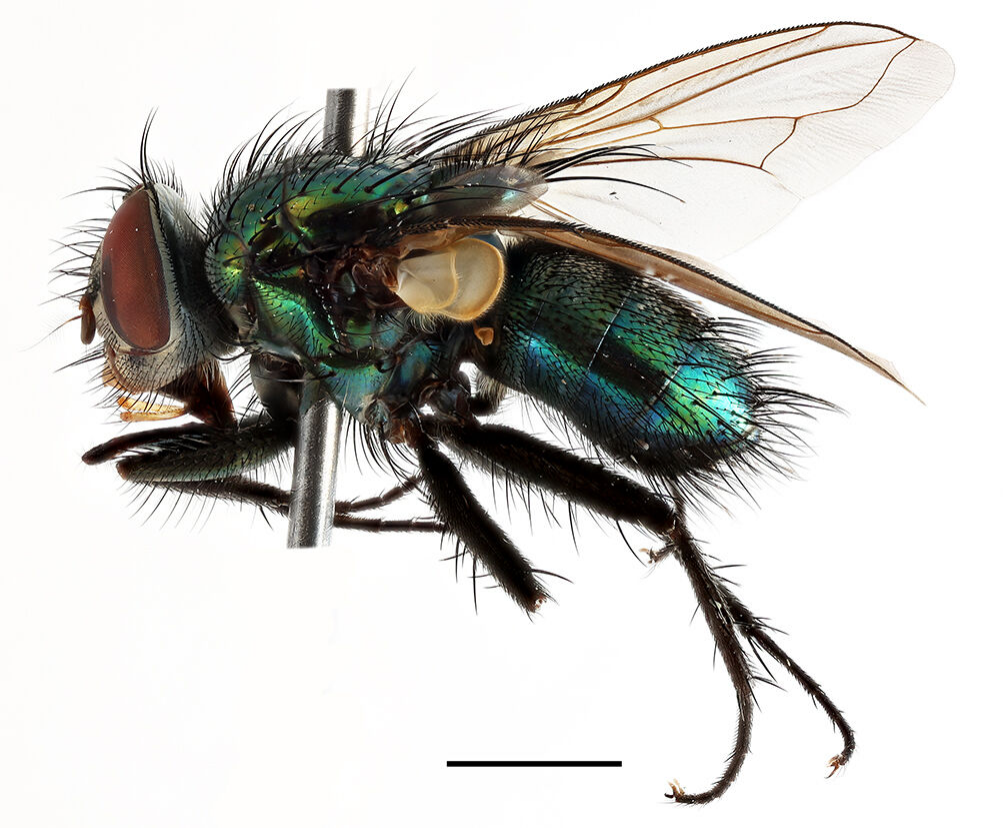
Habitus, left lateral view of *Isomyianatalensis* male BMSA DIP 20283 from South Africa; scale bar = 2 mm.

**Preferred environment**: forest, closed woodlands, montane forest, mist-belt forest in the margin, dune forest, forest margins, grass, grasslands, montane grasslands, Little Berg Summits *Themeda* grassland, riverine bush Montane slopes, riverine vegetation (Tugela River), straddling Mahai River, riverside in open road and environments with little anthropogenic intervention (Simes cottage, caravan park environments and garden areas). In Namibia, the Mesic Savannah Biome (Kurahashi and Kirk-Spriggs 2006). **Recorded elevations**: 30–1981 m a.s.l. **Seasonality**: common and abundant species present almost all year, peaking in February and March and absent from May to July. In Namibia, only one specimen was collected (Kurahashi and Kirk-Spriggs 2006). **Behaviour and ecology**: collected in sparse and dense *Leucosidea* Eckl. and Zeyh. sp. dominated scrub, also on *Protea* L. flowers, such as *Proteacaffra* woodland and *Protearoupelliae* Meisn. **Life cycle and developmental stages**: unknown. **Collection methods**: hand net, hilltopping and with yellow pans, Malaise and M/V light traps and ex a Malaise trap. In Namibia, hand net (Kurahashi and Kirk-Spriggs 2006). **Illustrations and photographs**: male habitus as in Fig. 56. Male terminalia as in fig. 11 in Zumpt (1958).

**Material examined**: Suppl. material 1.

#### Isomyia oculosa

(Villeneuve, 1917)

464ACE67-42CD-5E0A-97DF-9886AD0D8830

= Thelychaeta oculosa Villeneuve, 1917: 342.**Type locality**: South Africa and North-West Rhodesia [Zambia], Chilanga R. **Remarks**: STs in NHMUK and PT in SAMC.

##### Distribution

**Figure 57.**
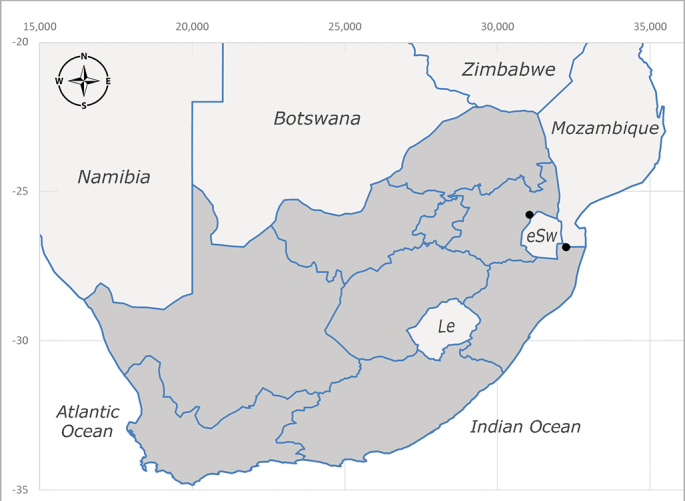
*Isomyiaoculosa* occurrence map in South Africa, including eSwatini (eSw) and Lesotho (Le); horizontal axis longitude east and vertical axis latitude south.

**Afrotropical**: Sierra Leone, South Africa (Fig. 57), Zambia and Zimbabwe.

##### Notes

**Figure 58.**
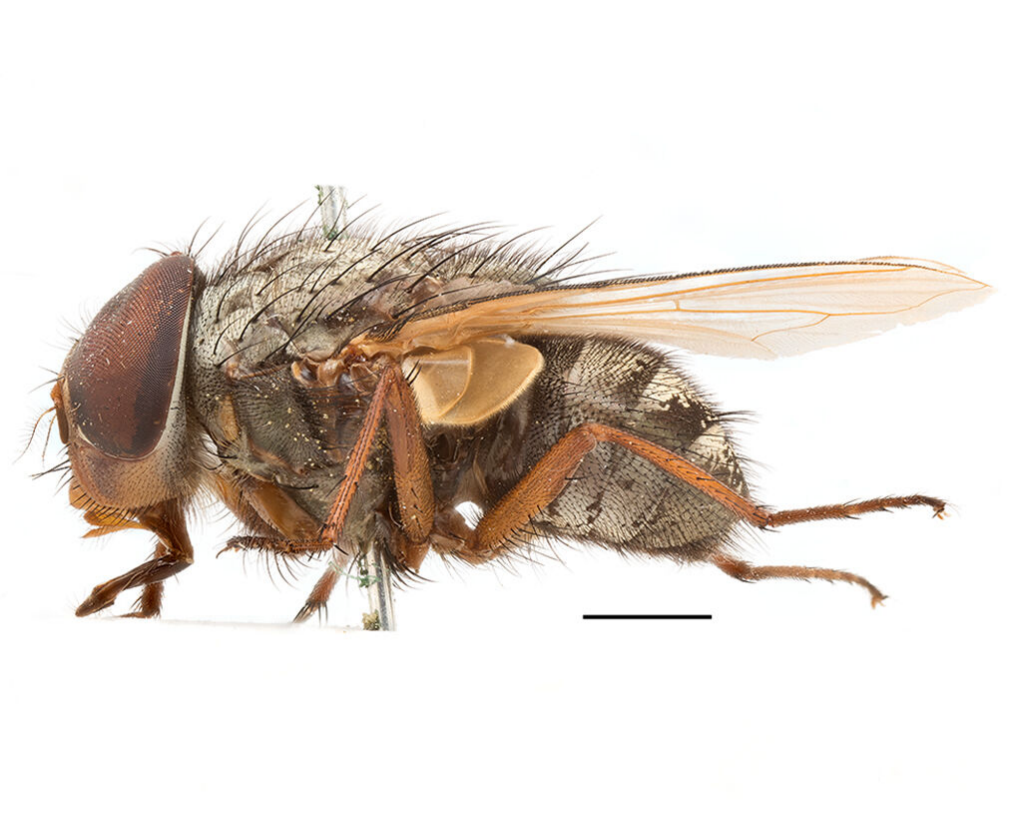
Habitus, left lateral view of *Isomyiaoculosa* male NHMUK 010580055 ST from Zambia (Copyright NHMUK); scale bar = 2 mm.

**Preferred environment**: in Zimbabwe, Cuthbertson (1933) reported *Isomyiaoculosa* (as *Strongyloneuraoculosa* Villeneuve) on twigs and trunks of trees in a dense forest at the Chirinda Forest, Vumba Mountains (in Cloudlands), Kadoma (as Gatooma) and Eastern Victoria. **Recorded elevations**: no data. **Seasonality**: uncommon species with only three specimens recorded in November. **Behaviour and ecology**: unknown. **Life cycle and** **developmental stages**: unknown. **Collection methods**: in Zambia and Zimbabwe, collected inside houses. **Illustrations and photographs**: male habitus as in Fig. 58. Male terminalia as in fig. 2 in Zumpt (1958).

**Type material examined**: *T.oculosa*: 1 ? SYN-TYPE // 24/9/13 / Chilanga R. / W. Lusaka; N.W.R. / (R.C.W.) / in house // R.C. Wood // Pres. by / Com. Inst. Ent. / B.M. 1956-102 // [NHMUK 010580057]. 1 ? SYN-TYPE // 25/09/13 M / Chilanga / N.W. Rhodesia / (R.C.W) / 4030 / in house // Pres. by / Com. Inst. Ent. / B.M. 1956-102 // *Thelychaeta* / *oculosa* Villen / Villeneuve det. // [NHMUK 010580055]. *T.oculosa*: 1 ? Para-Type // Bulawayo / 10.ix.1910 / E.C. Chubb / caught in house // S. Afri. Mus. // 119 // *Thelychaeta* / *oculosa* / Type Villeneuve // [SAMC DIP A011149].

**Material examined**: Suppl. materials 1, 2.

#### Isomyia pubera

(Villeneuve, 1917)

63E8DB1E-CED2-5BF9-8E8A-74FEDEBD9E40

= Thelychaeta pubera Villeneuve, 1917: 340. **Type locality**: British East Africa [Kenya], Eldoret; South Africa, Kloof [Durban]; North-West Tanganyka [Tanzania]; Uganda, Entebbe.
= Thelychaeta jactatrix Villeneuve, 1917: 343. **Type locality**: Belgian Congo [Democratic Republic of Congo], Stanleyville [Kisangani].
= Thelychaeta villeneuvei Curran, 1927: 3. **Type locality**: Democratic Republic of Congo [Belgian Congo], Stanleyville [Kisangani].
= Strongyloneura cupreithorax Curran, 1931: 1. **Type locality**: South Africa, Transvaal [Gauteng], Pretoria, Barberton.

##### Distribution

**Figure 59.**
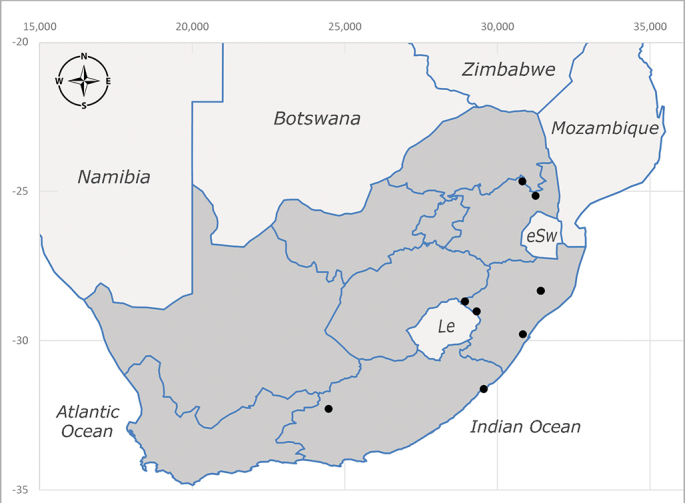
*Isomyiapubera* occurrence map in South Africa, including eSwatini (eSw) and Lesotho (Le); horizontal axis longitude east and vertical axis latitude south.

**Afrotropical**: Democratic Republic of Congo, Equatorial Guinea, Kenya, South Africa (Fig. 59), Tanzania, Uganda and Zimbabwe.

##### Notes

**Figure 60.**
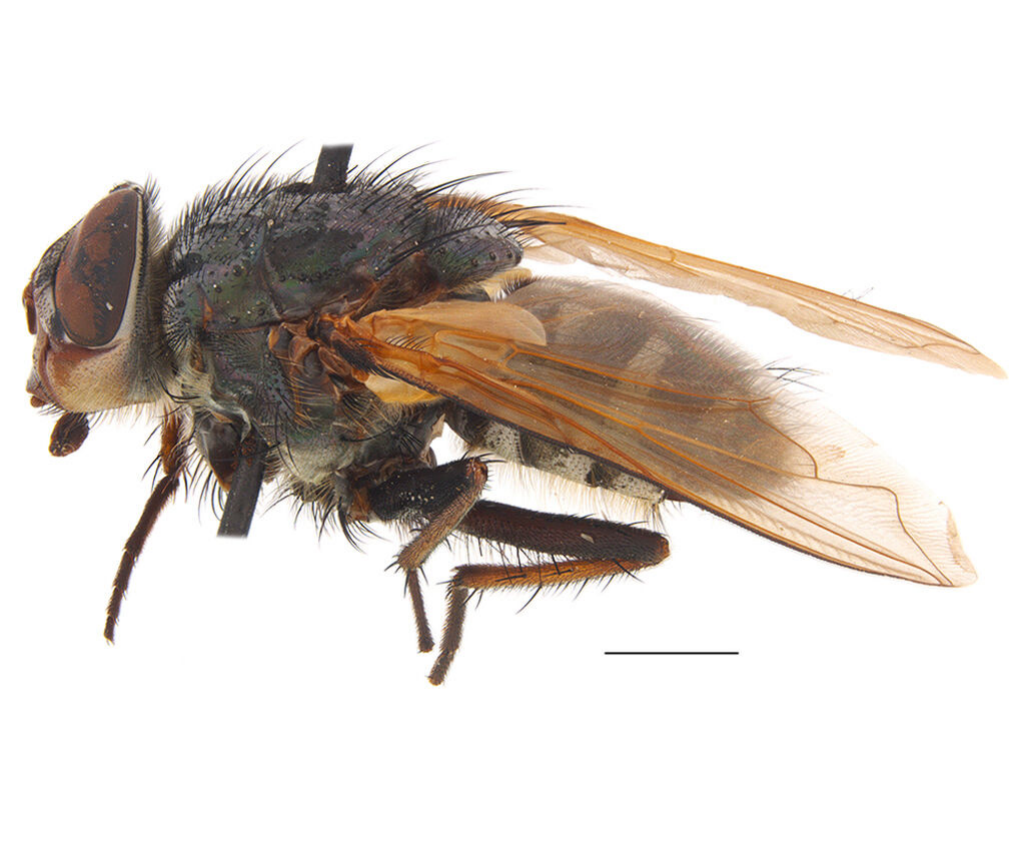
Habitus, left lateral view of *Isomyiapubera* male SAMC DIP A011150 LT from South Africa; scale bar = 2 mm.

**Preferred environment**: no data. **Recorded elevations**: 1450–1981 m a.s.l. **Seasonality**: present almost all year, peaking in September, absent in April, May, June and August with low numbers in other months. **Behaviour and ecology**: Villeneuve (1917) reported that the unique female used for the description of *I.jactatrix* was captured by a *Bembex* sp. at Stanleyville, Democratic Republic of Congo, collected by Bequaert, M.J. iii.1915. Another specimen of *I.pubera* was also recorded as "taken from *Bembex*" in the same region and date, but collected by Lang and Chapin. **Life cycle and developmental stages**: unknown. **Collection methods**: Malaise traps. **Illustrations and photographs**: male habitus as in Fig. 60. Male terminalia as in fig. 3 in Zumpt (1958).

**Type material examined**: *T.pubera*: 1 ? LT // Kloff / 16.xii.14 / H.W. Bell-Marley // S.A.M. // Type // Lectotype // *Thelychaeta* / *pubera* n. sp / Det. Villeneuve // *Isomyia* / *pubera* Ville. / Vid. Zumpt 56 // slide no 39 // Slide SAM: 33B10 // [SAMC DIP A011150].

**Material examined**: Suppl. material 1.

#### Isomyia transvaalensis

Zumpt and Stimie, 1965

6CC91A6F-6702-5A72-A66F-D3BFF497065E

= Isomyia transvaalensis Zumpt and Stimie, 1965: 8. **Type locality**: South Africa, Transvaal [North-West], Rustenburg. **Remarks**: HT in NMSA.

##### Distribution

**Figure 61.**
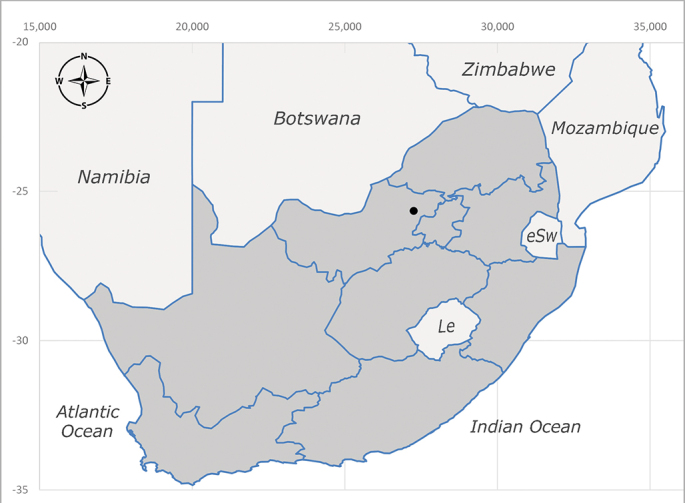
*Isomyiatransvaalensis* occurrence map in South Africa, including eSwatini (eSw) and Lesotho (Le); horizontal axis longitude east and vertical axis latitude south.

**Afrotropical**: South Africa (Fig. 61).

##### Notes

**Figure 62.**
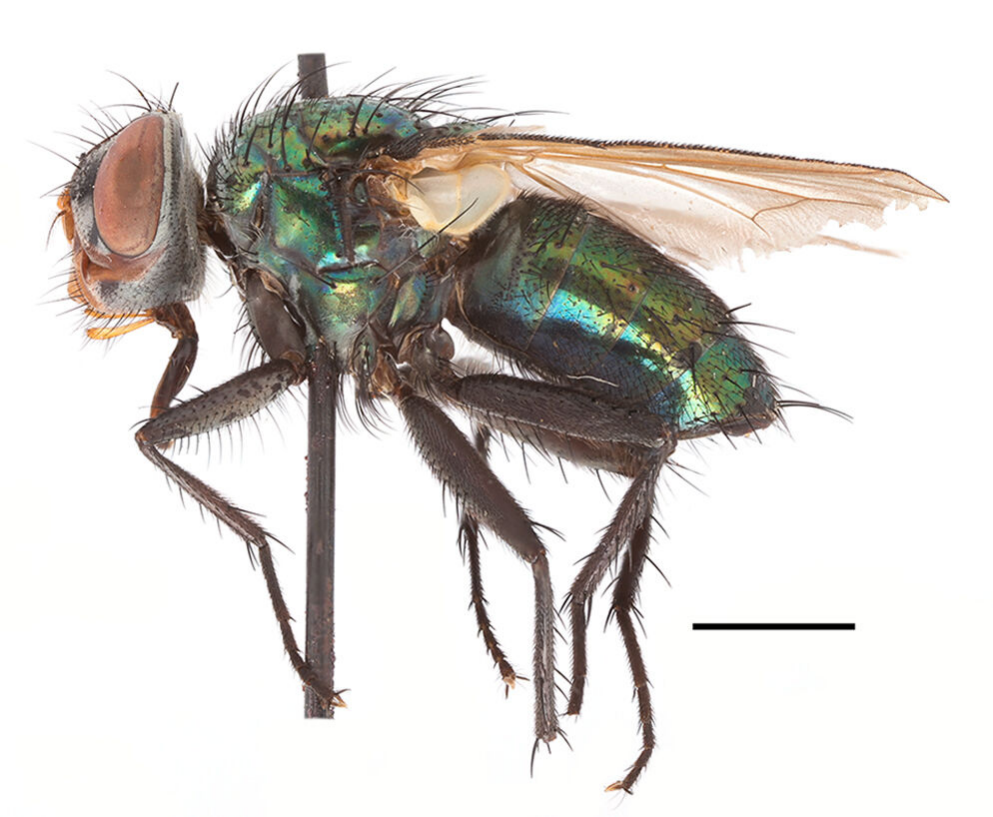
Habitus, left lateral view of *Isomyiatransvaalensis* male NMSA DIP 19760 HT from South Africa (male without terminalia); scale bar = 2 mm.

**Preferred environment**: no data. **Recorded elevations**: no data. **Seasonality**: only one specimen collected in December. **Behaviour and ecology**: unknown. **Life cycle and** **developmental stages**: unknown. **Collection methods**: unknown. **Illustrations and photographs**: male habitus as in Fig. 62. Male terminalia as in fig. 4 in Zumpt (1965).

**Type material examined**: *I.transvaalensis*: 1 ? Rustenburg / Transvaal / 3.xii.61. // HOLOTYPUS // *Isomyia* / *transvaalensis* / Z[um]pt & Stimie // [NMSA-DIP 019760].

**Material examined**: Suppl. material 1.

#### Isomyia tristis

(Bigot, 1888)

48CF351C-43C9-563A-81FC-2B8513FD4A81

= Curtonevra tristis Bigot, 1888: 613. **Type locality**: South Africa, Port-Natal [Durban]. **Remarks**: HT in NHMUK and LT in SAMC.
= Apollenia psophis Séguy, 1933: 74. **Type locality**: Mozambique, Macequece.

##### Distribution

**Figure 63.**
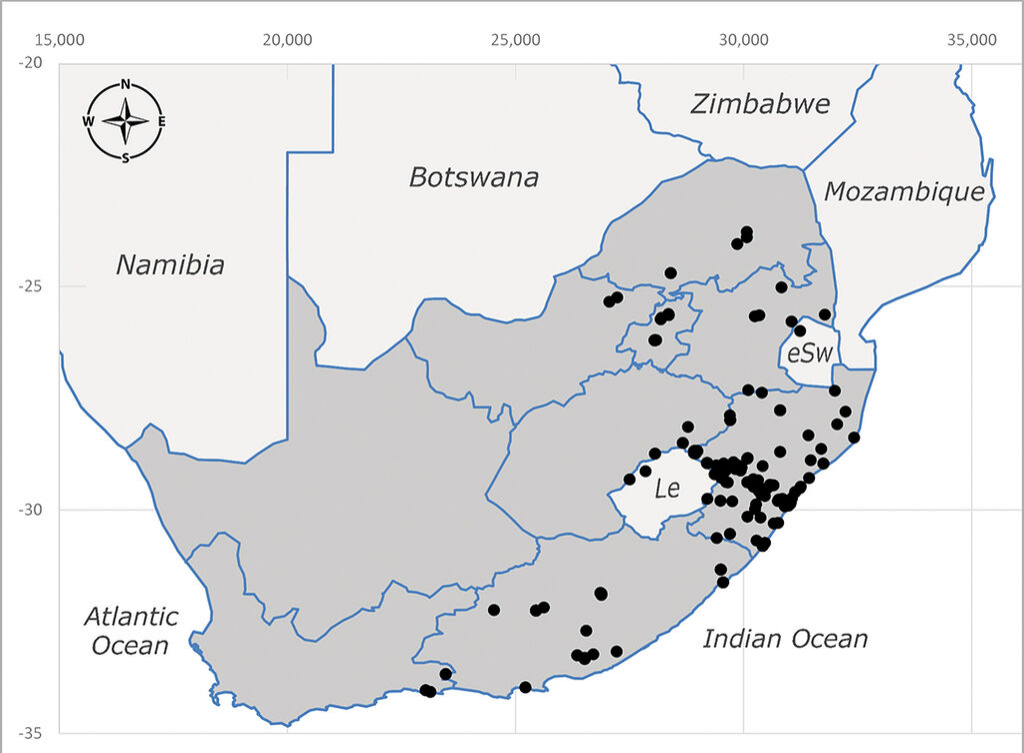
*Isomyiatristis* occurrence map in South Africa, including eSwatini (eSw) and Lesotho (Le); horizontal axis longitude east and vertical axis latitude south.

**Afrotropical**: Angola*, Botswana, Cameroon*, Democratic Republic of Congo, Ethiopia, ?Ghana, Kenya, Liberia, Lesotho, Mozambique, Namibia, Rwanda, South Africa (Fig. 63), Tanzania, Uganda, Zambia and Zimbabwe.

##### Notes

**Figure 64.**
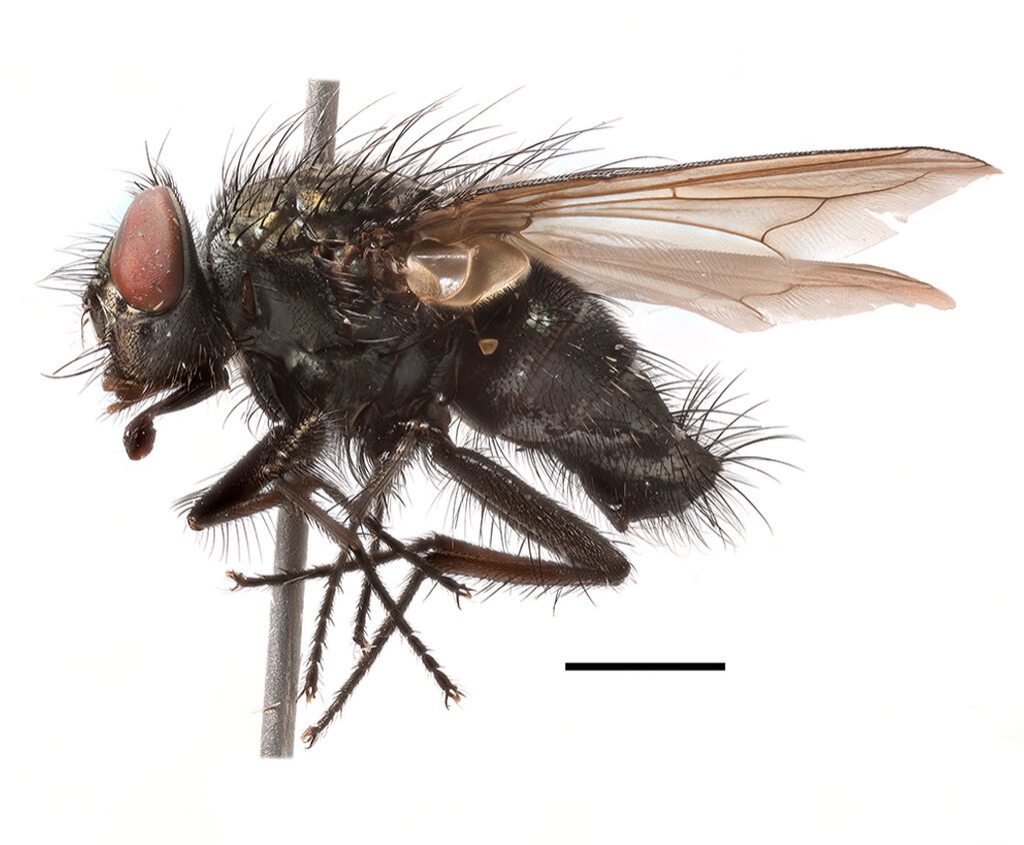
Habitus, left lateral view of *Isomyiatristis* male BMSA DIP 20287 from South Africa; scale bar = 2 mm.

**Preferred environment**: montane environments (grassland and bush, meadow, *Podocarpus* L'Hér ex Pers sp. forest margins and slopes), savannahs (*Acacia* and dry open), forests (Mbhome, pine and swamp), areas associated with forests (creeks, gorges, margins, stream, slopes, trail, roads and burnt-out forests), bush (bushveld, coastal, dry bushveld, in front beach, riverine; mixed *Acacia* woodland and mixed woodland), grasslands, amongst others. Additionally, caravan park, grotto near farm, thornveld camp ground. In Mozambique, it was associated with gallery forest. In Namibia, associated with the Mesic Savannah and the Succulent Karoo Biomes (Kurahashi and Kirk-Spriggs 2006). **Recorded elevations**: 30–1981–?3500 m a.s.l. **Seasonality**: present year-round, peaking in March and April; lower abundance in coldest months. Uncommon species in Namibia, present only in April and September (one specimen each) (Kurahashi and Kirk-Spriggs 2006). **Behaviour and ecology**: collected on flowers of *Cussonia* sp., *Proteacaffra* and general flowers and forests of Dassiekrantz (Makhanda, Eastern Cape), grassveld flowering daisies *Leucosidea* dominated scrub and euphorbias out of the forest in South Africa. In the Eastern Cape, one male was dropped at nest entrance by *Bembixalbofasciata* F. Smith and ex-nest of *Dasyproctusbraunsii* (Kohl) (as *D.ruficaudis* (Arnold)). Females were observed on fresh cattle dung in Zimbabwe by Cuthbertson (1934) (as *Strongyloneuratristis*). **Life cycle and developmental stages**: unknown. **Collection methods**: sweeping, at bait, M/V light trap, yellow and white pans and Malaise traps. In Namibia, using yellow pan and pitfall traps (Kurahashi and Kirk-Spriggs 2006). **Illustrations and photographs**: male habitus as in Fig. 64. Male terminalia as in fig. 4–right in Zumpt (1958).

**Type material examined**: *C.tristis*: 1 ? // Holo / type // *Curtoneura* / *tristis* Bigot / Port-Natal / ex. Bigot Coll: / B.M.1960-539. // Genus / *Apollenia* / Bezzi / det. Villen. // BMNH(E) # 231136 // [NHMUK 010832105]. 1 ? Durban / Natal // Pres. by / Com. Imp. Bur. Ent. // 1917-94 // *Thelychaeta* / Dr Villeneuve det. / tristis / sec type. Bigot // 861 // [NHMUK 010832122]. 1 ? // Marley / Jan. 1915 / P. Port Shepstone // *Apollenia* / *tristis* Bigot. / Lec Type // S.A. Museum. // [SAMC DIP A011159]. 1 ? / K/Kloof / Marley / 1-15 // S.A. Museum // *Apollenia* / *tristis* /sec.type Bigot / det. Villeneuve // [SAMC DIP A015196].

**Material examined**: Suppl. materials 1, 2.

#### Pseudorhyncomyia

Peris, 1952

7B0E45B7-ED90-59F2-A566-BD28FC6ED047

= Pseudorhyncomyia Peris, 1952: 58. **Type species**: *Rhyncomyiabraunsi* Villeneuve, 1920, by original designation.

#### Pseudorhyncomyia braunsi

(Villeneuve, 1920)

AEBAB651-7040-556F-A836-8F9F6C0EBA1A

= Rhyncomyia braunsi Villeneuve, 1920: 158. **Type locality**: South Africa, Cape Province [Western Cape], Willowmore. **Remarks**: LT in NMSA, designated by Zumpt (1958: 124).

##### Distribution

**Figure 65.**
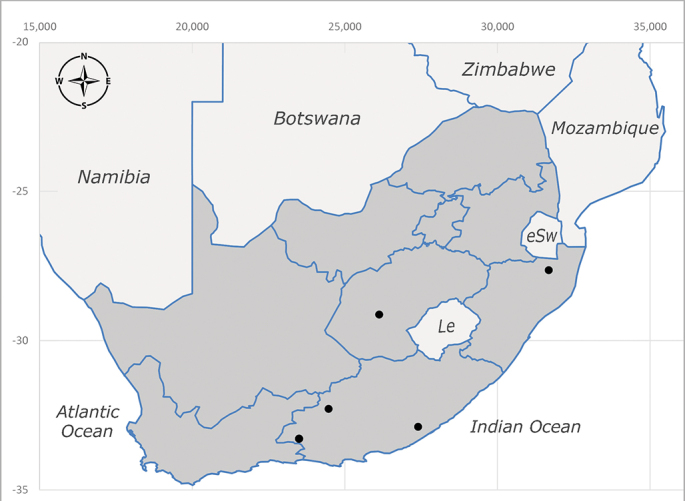
*Pseudorhyncomyiabraunsi* occurrence map in South Africa, including eSwatini (eSw) and Lesotho (Le); horizontal axis longitude east and vertical axis latitude south.

**Afrotropical**: Namibia, South Africa (Fig. 65) and Tanzania.

##### Notes

**Figure 66.**
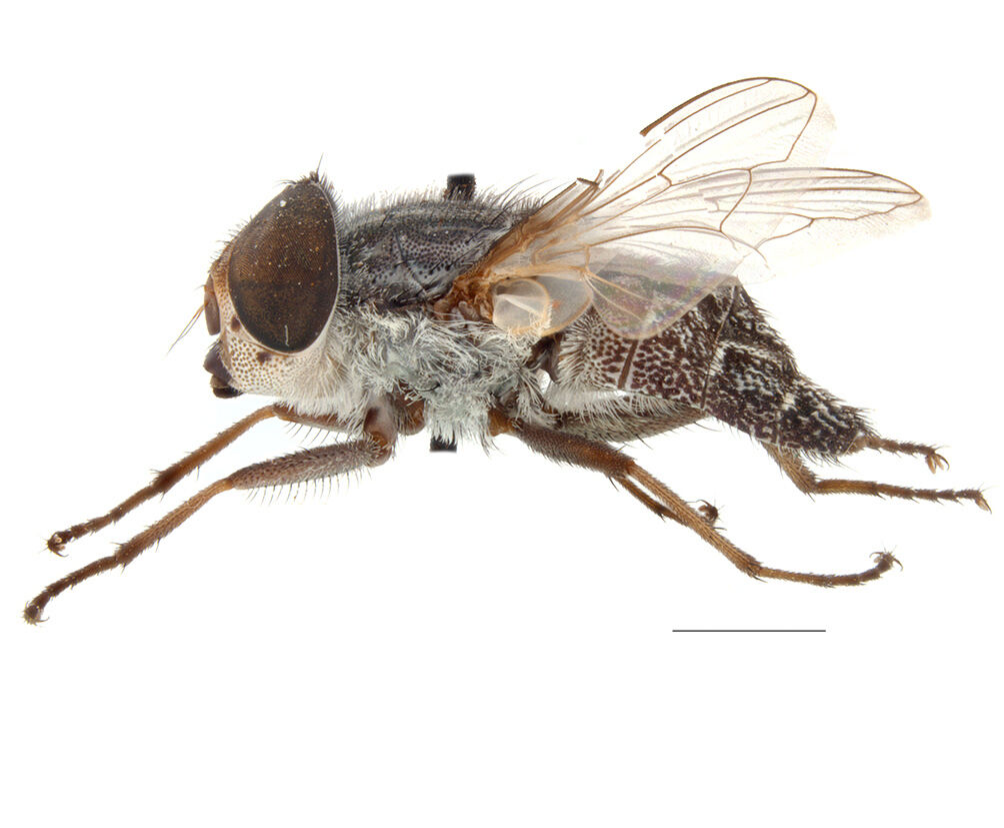
Habitus, left lateral view of *Pseudorhyncomyiabraunsi* male SAMC DIP A013585 from South Africa; scale bar = 2 mm.

**Preferred environment**: no data. **Recorded elevations**: no data. **Seasonality**: present in low numbers, more abundant in May and absent most of the year. In Namibia, low numbers in February (Kurahashi and Kirk-Spriggs 2006). **Behaviour and ecology**: recorded by Villeneuve (1920) and compiled by Ferrar (1987), *P.braunsi* larvae were found in South Africa in the rubbish heaps of harvester termites of the genus *Hodotermes* Hagen, which accumulate on the soil surface near the opening of subterranean nests. Larvae were noted to attack live termites that approach the heaps and were successfully reared on live termites. Pupation occurs under the heaps. The species was also recorded in wood with termites. **Life cycle and** **developmental stages**: unknown. **Collection methods**: in Namibia with UV-light (Kurahashi and Kirk-Spriggs 2006). Collected on dung beetle soil heap in Tanzania (as Tanganyika). **Illustrations and photographs**: female habitus as in figs. 14–16, 18, 20, 22, 24 in Rognes (2013). Male habitus as in Fig. 66 and figs. 17, 19, 21, 23, 25, 26 in Rognes (2013). Male terminalia as in fig. 40 in Zumpt (1958).

**Type material examined**: *P.braunsi*: 1 ? // Cape Province / Willowmore / 5.V.1920 / Brauns // Lectotype // [NMSA-DIP 19835].

**Material examined**: Suppl. materials 1, 2.

#### Rhyncomya

Robineau-Desvoidy 1830

96A60661-31B3-58C4-9809-8D9085BF57A7

= Beria Robineau-Desvoidy, 1830: 418. **Type species**: *Beriainflata* Robineau-Desvoidy, 1830, by monotypy.
= Rhyncomya Robineau-Desvoidy, 1830: 424. **Type species**: *Muscafelina* Fabricius, 1794, by monotypy.
= Trichometallea Townsend, 1917: 194. **Type species**: *Trichometalleapollinosa* Townsend, 1917, by original designation.
= Rhynchomyiopsis Townsend, 1917: 195. **Type species**: *Rhynchomyiopsisindica* Townsend, 1917 (= *Rhyncomyatownsendi* James, 1977), by original designation.
= Doljia Suster, 1953: 769. **Type species**: *Doljiaviridicauda* Šuster, 1953 (= *Rhyncomyaspeciosa* (Loew, 1844)), by monotypy.
= Sokotra Lehrer, 1970: 32. **Type species**: *Rhyncomyiavarifrons* Beckerm 1910, by original designation.

#### Rhyncomya bicolor

(Macquart, 1843)

4426160D-035A-5CB3-BD61-C9BEAA74689F

= Idia bicolor Macquart, 1844: 124. **Type locality**: South Africa, Cape of Good Hope [Western Cape]. **Remarks**: HT in NHMUK.
= Rhyncomya crinicauda Villeneuve, 1927: 22. **Type locality**: South Africa, Cape of Good Hope [Western Cape]. **Remarks**: HT in SAMC.

##### Distribution

**Figure 67.**
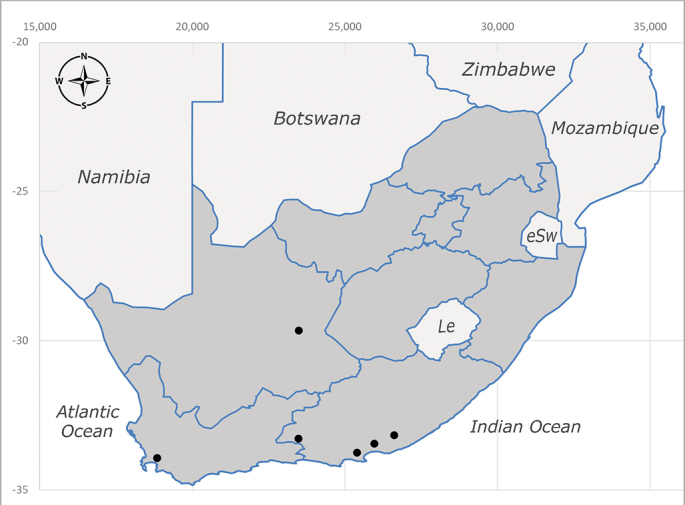
*Rhyncomyabicolor* occurrence map in South Africa, including eSwatini (eSw) and Lesotho (Le); horizontal axis longitude east and vertical axis latitude south.

**Afrotropical**: Namibia and South Africa (Fig. 67).

##### Notes

**Figure 68.**
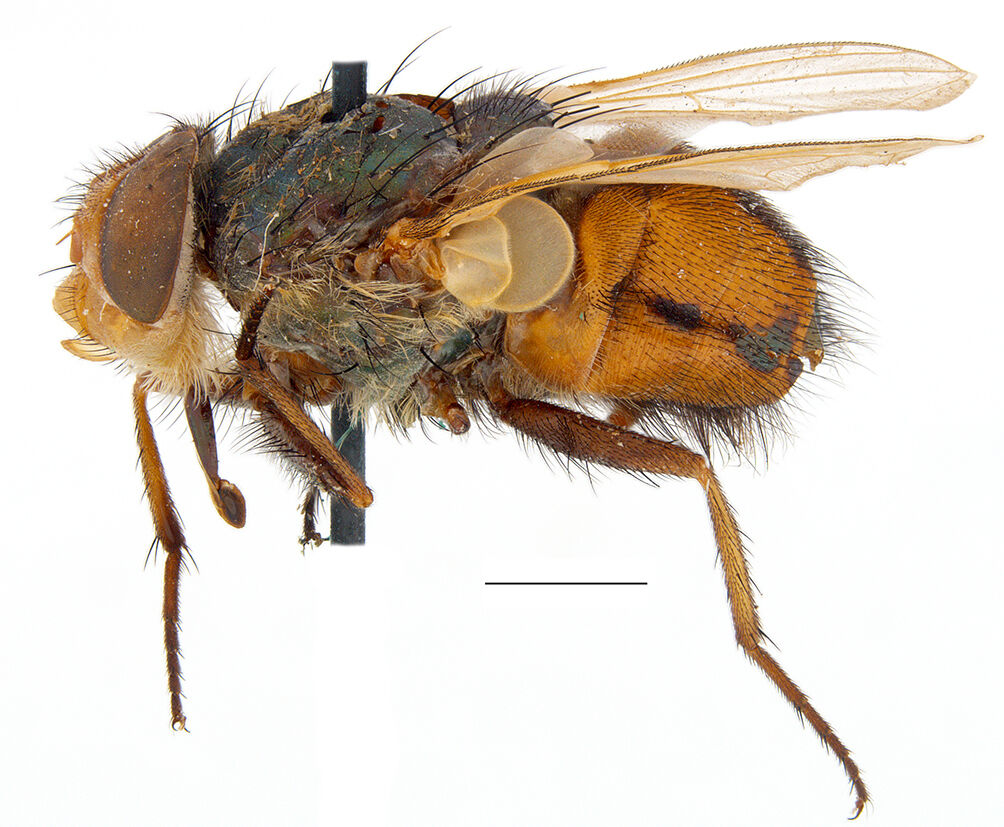
Habitus, left lateral view of *Rhyncomyabicolor* SAMC DIP A011180 (male without terminalia); scale bar = 2 mm.

**Preferred environment**: sand dunes. In Namibia, the Arid Savannah Biome (Kurahashi and Kirk-Spriggs 2006). **Recorded elevations**: 300 m a.s.l. **Seasonality**: low numbers between January and March and September and October, absent the rest of the year. In Namibia, a single specimen recorded between January and February (Kurahashi and Kirk-Spriggs 2006). **Behaviour and ecology**: unknown. **Life cycle and developmental stages**: unknown. **Collection methods**: pitfall trap in Namibia (Kurahashi and Kirk-Spriggs 2006). **Illustrations and photography**: male habitus as in Fig. 68. Male terminalia as in fig. 51 in Zumpt (1958).

**Type material examined**: *I.bicolor*: 1 ? // Holo-type // *Idia* / *bicolor* / n. sp. // *S. Bicolor* / Idia . iv. Macq / *C. B. Sp.* / J. Bigot // [NHMUK 010832205]. *B.crinicaudata*: 1 ? // Stellenbush / 9.86 // Holotypus // *Beria* / *crinicaudata* / n. sp. // *crinicaudata* / type Villen. // slide no. 6 // Slide / SAM: / 33B12 // [SAM-DIP A011181].

**Material examined**: Suppl. material 1.

#### Rhyncomya botswanae

Zumpt, 1974

D40CB980-CC93-5472-9FB7-2891272C6EF5

= Rhyncomya botswanae Zumpt, 1974: 129. **Type locality**: Botswana, Moremi Reserve. **Remarks**: HT and PT in NHMUK.

##### Distribution

**Figure 69.**
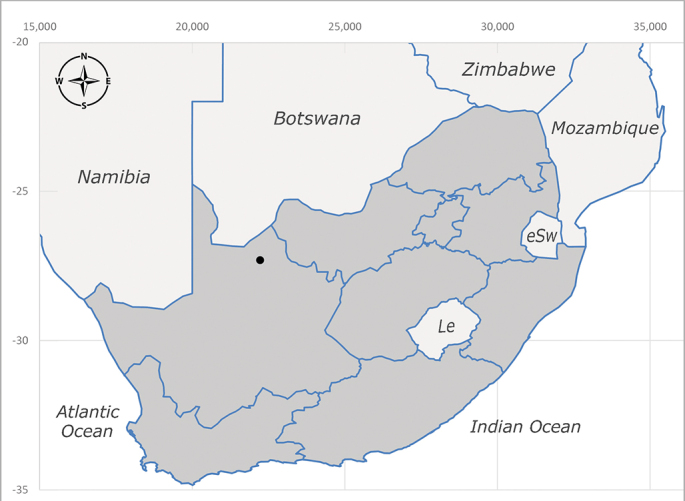
*Rhyncomyabotswanae* occurrence map in South Africa, including eSwatini (eSw) and Lesotho (Le); horizontal axis longitude east and vertical axis latitude south.

**Afrotropical**: Botswana and South Africa* (Fig. 69).

##### Notes

**Figure 70.**
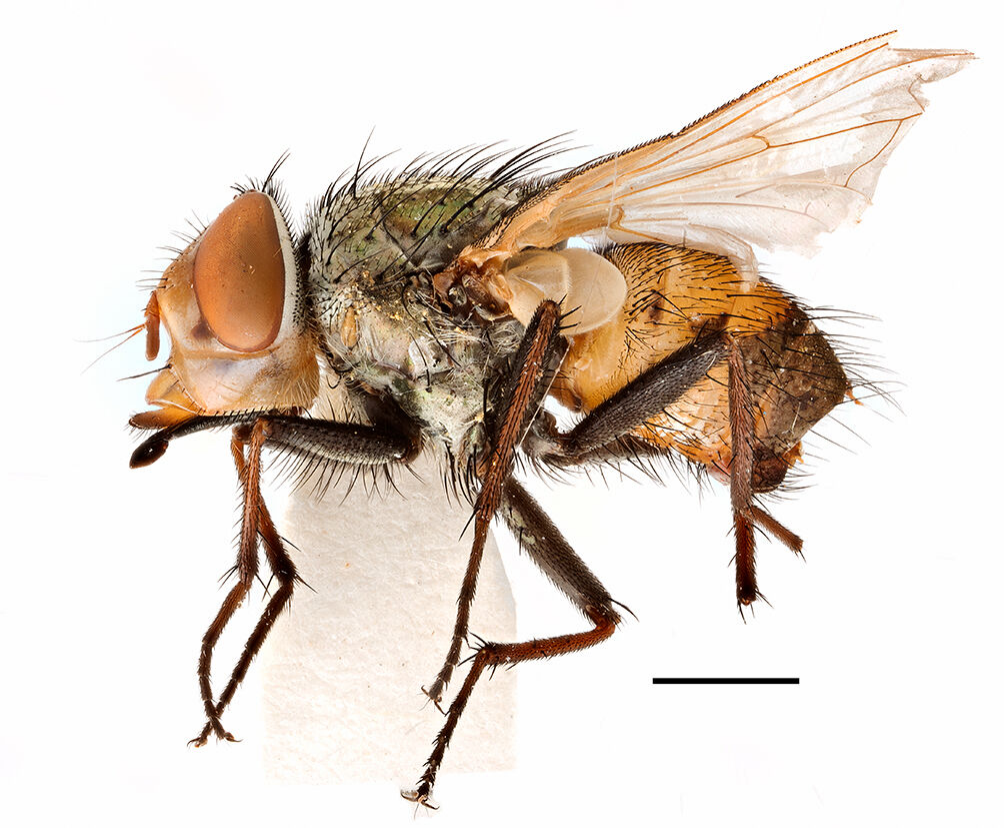
Habitus, left lateral view of *Rhyncomyabotswanae* male NHMUK 010832200 PT from Botswana (male without terminalia) (Copyright NHMUK); scale bar = 2 mm.

**Preferred environment**: Gordonia Plains Shrubland, *Senegaliamellifera* (M. Vahl) Seigler and Ebinger on red sand. **Recorded elevations**: 1035 m a.s.l. **Seasonality**: a single specimen collected between September and November. **Behaviour and ecology**: unknown. **Life cycle and developmental stages**: unknown. **Collection methods**: Malaise trap. **Illustrations and photography**: male habitus as in Fig. 70. Male terminalia as in fig. 1 in Zumpt (1974).

**Type material examined**: *R.botswanae*: 1 ? // Holo-type // Botswanhoa (B11) / Moremi Reserve, / 19º23'S, 23º33'E / 18-20.iv.1972 // Southern / African Exp. / B.M.1972-1 // slide no. 38 // *Rhyncomya* ? / *botswanae* n.sp / Zumpt 1973 // Slide prep.: / 010194475 // [NHMUK 010832201]. *R.botswanae*: 1 ? // Para-type // Botswana (B7) / Kuke Pan, / 20º56'S, 22º25'E / 14-15.iv.1972 // Southern / African Exp. / B.M.1972-1 // slide no. 82 // *Rhyncomya* ? / *botswanae* n. sp / Zumpt 1973 // Slide prep.: / 010194473 // [NHMUK 010832200]

**Material examined**: Suppl. materials 1, 2.

#### Rhyncomya buccalis

Villeneuve, 1927

A3EE2B8F-CAC1-584F-B061-C1CFA2D8CE55

= Rhyncomya buccalis Villeneuve, 1927: 24. **Type locality**: Congo Belge [Democratic Republic of Congo], Mufungwa; South Africa.

##### Distribution

**Afrotropical**: Democratic Republic of Congo, Kenya, Malawi, Mozambique, South Africa, Tanzania*, Uganda and Zambia*.

##### Notes

**Figure 71.**
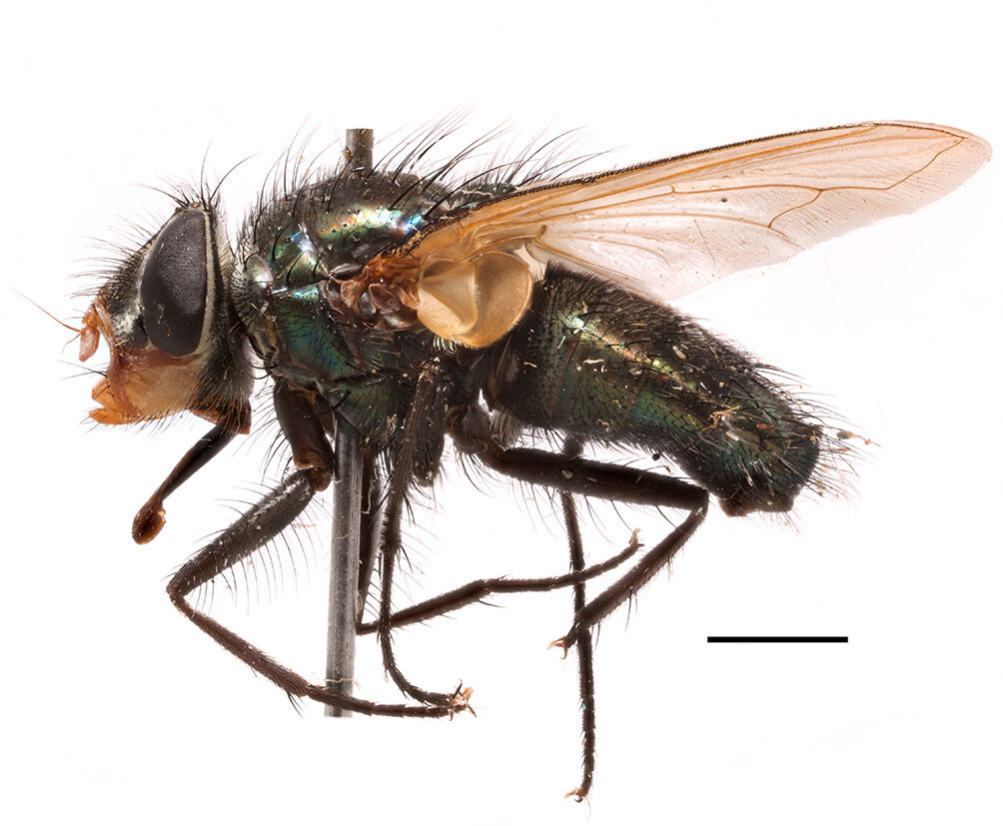
Habitus, left lateral view of *Rhyncomyabuccalis* male NMSA DIP 61658 from Tanzania; scale bar = 2 mm.

No specimens examined for South Africa, based on Pont (1980). **Illustrations and photographs**: male habitus as in Fig. 71. Male terminalia as in fig. 44 in Zumpt (1958).

**Material examined**: Suppl. material 2.

#### Rhyncomya cassotis

(Walker, 1849)

5877F280-DE75-5DBE-A7D6-D8027A9013A6

= Tachina cassotis Walker, 1849: 761. **Type locality**: Sierra Leone. **Remarks**: HT in NHMUK.

##### Distribution

**Figure 72.**
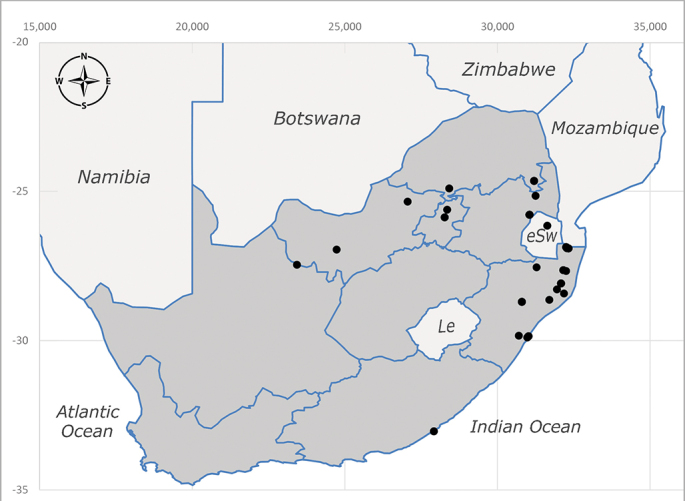
*Rhyncomyacassotis* occurrence map in South Africa, including eSwatini (eSw) and Lesotho (Le); horizontal axis longitude east and vertical axis latitude south.

**Afrotropical**: Angola*, Benin*, Cameroon, Democratic Republic of Congo, Ethiopia, Gambia, Kenya, Mali, Malawi, Mozambique, Namibia, Nigeria, Sierra Leone, South Africa (Fig. 72), eSwatini (Swaziland)*, Tanzania, Togo*, Uganda, Zambia and Zimbabwe. **Palaearctic**: Saudi Arabia.

##### Notes

**Figure 73.**
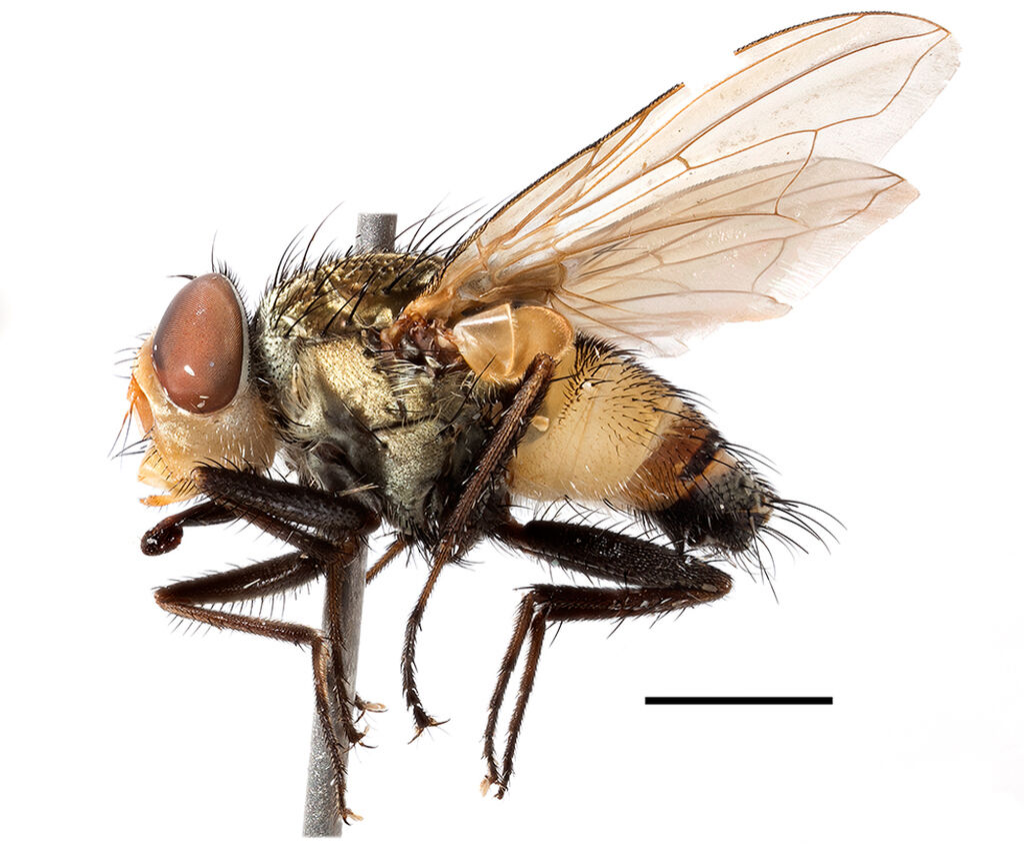
Habitus, left lateral view of *Rhyncomyacassotis* male BMSA DIP 17182 from South Africa; scale bar = 2 mm.

**Preferred environment**: *Acacia* savannah and *Acacia* veld, sand forest and broad-leafed deciduous forest, mixed woodland, broad-leafed deciduous woodland and woodland near stream and camp. In Benin, to lowland gallery forest and stream-bed, remnant forest and agricultural plots; Cameroon to degraded savannah forest; Democratic Republic of Congo to bush paths and village environs; Kenya to rocks and river margins; in Namibia, to degraded sand forest, cultivated plots and Kwando River floodplain; eSwatini to Panata Ranch and Bushveld; Togo to vegetated stream-bed; and Zambia to Central Zambezian Miombo Woodlands. In Namibia, also associated with the Arid and Mesic Savannah Biome (Kurahashi and Kirk-Spriggs 2006). **Recorded elevations**: 120–1480 m a.s.l. **Seasonality**: common species present year-round, most abundant in March and December and less in January and August. In Namibia, it was collected in low numbers (Kurahashi and Kirk-Spriggs 2006). **Behaviour and ecology**: Cuthbertson (1934) observed females and males visiting Umbelliferae flowers in Balla Balla (now Mbalabala), Gatooma and Salisbury (now Harare) in Zimbabwe. **Life cycle and developmental stages**: unknown. **Collection methods**: Malaise traps and MV light. In Benin, Cameroon, Togo, Zambia and Zimbabwe, Malaise trap; Democratic Republic of Congo and Mozambique by sweeping net. In Namibia, by hand net, yellow pans, pitfalls and Malaise traps (Kurahashi and Kirk-Spriggs 2006). **Illustrations and photographs**: male habitus as in Fig. 73. Male terminalia as in fig. 60 in Zumpt (1958) and figs. 107, 109 in Rognes (2002).

**Type material examined**: *T.cassotis*: 1? // Holo-type // Sierra Leone. / W. Africa. / Reud D.T. Morgan. // HOLOTYPE ? / *Tachina* / *cassotis* Walker / 1849, List Dipt. / Brit. Mus., 4:761 // *Tachinia* / Type / *cassotis* / Walk // ? *Nomia* sp / det. G.R. Else. 1974 (Hymenoptera) // [NHMUK 010832158].

**Material examined**: Suppl. materials 1, 2.

#### Rhyncomya currani

Zumpt, 1958

7C5A3225-3B07-506C-A681-F724B0751FCB

= Rhyncomya pollinosa Curran, 1931: 20. **Type locality**: South Africa, Transvaal [Gauteng], Pretoria. **Remarks**: nec. *R.pollinosa* Townsend 1917. **Remarks**: HT in SANC.
= Rhyncomya currani Zumpt, 1958: 144. **Type locality**: South Africa, Transvaal [Gauteng], Pretoria. **Remarks**: new replacement name for *R.pollinosa* Curran, 1931 by Zumpt in 1958; HT and AT in SANC at ARC.

##### Distribution

**Figure 74.**
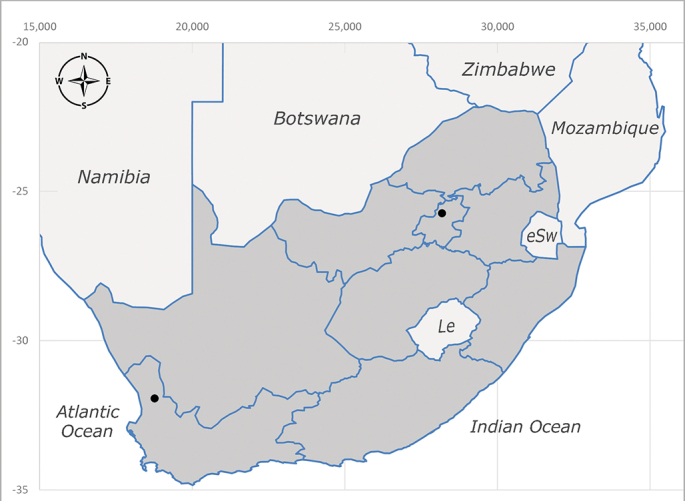
*Rhyncomyacurrani* occurrence map in South Africa, including eSwatini (eSw) and Lesotho (Le); horizontal axis longitude east and vertical axis latitude south.

**Afrotropica**: South Africa (Fig. 74).

##### Notes

**Figure 75.**
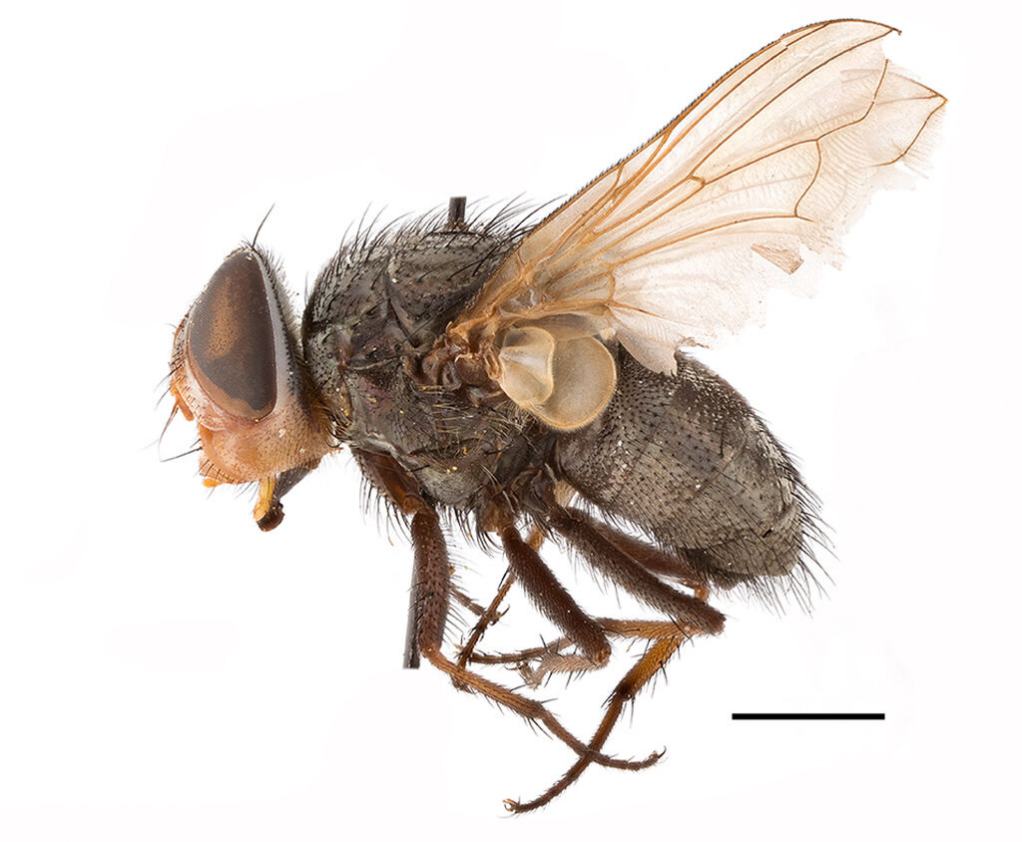
Habitus, left lateral view of *Rhyncomyacurrani* male SANC 01901 HT from South Africa; scale bar = 2 mm.

**Preferred environment**: no data. **Recorded elevations**: no data. **Seasonality**: uncommon, present only in September. **Behaviour and ecology**: unknown. **Life cycle and developmental stages**: unknown. **Collection methods**: unknown. **Illustrations and photographs**: male habitus as in Fig. 75. Male terminalia as in fig. 45 in Zumpt (1958).

**Type material examined**: *R.pollinosa*: 1 ? HT // Pretoria / 21.9.15 / H.K. Munro // *Rhyncomya* / Type / *pollinosa* ? / Curran. / No. // 1901 was ST258 // TYPH01901 // *Rhyncomyacurrani* Zumpt, 1958 / nom. nov.

**Material examined**: Suppl. material 1.

#### Rhyncomya dasyops

Bezzi, 1908

275855E2-C1A0-57D7-B1B7-BE6E28F2D893

= Rhyncomya dasyops Bezzi, 1908: 382. **Type locality**: Zaire [Democratic Republic of Congo], Haut-Congo [Oriental Province].
= Beria proxima Séguy, 1926: 12. **Type locality**: French Congo [Republic of Congo]. **Remarks**: Zumpt (1958) indicated that this species may be conspecific with *R.dasyops*, subsequently published as a synonym of *R.dasyops* by Pont (1980).
= ? Rhyncomya rugosa Séguy, 1926: 12. **Type locality**: Mozambique, Vallée du Pungoué. **Remarks**: Zumpt (1958) indicated that the original description of this species is inadequate and it needs to be considered as a synonym of *R.dasyops*; however, Pont (1980) considered the species as valid, but nowadays, the Systema dipterorum (Evenhuis and Pape 2022) considered it as a synonym of *R.dasyops*. Without a formal revision of the type specimen, we consider the species as a synonym, as was also suggested by Zumpt (1958).
= Rhyncomya nigropilosa Villeneuve, 1927: 19. **Type locality**: Rhodesia mérdionale [Zimbabwe], Salisbury [Harare]; Chilanga [Zambia]; Nigeria septetrionale [Nigeria]; Uganda Prot. [Uganda], Mt. Elgon, 3,700–3,900 ft.; Congo Belge [Democratic Republic of Congo], Elisabethville [Lubumbashi]; Abyssinie [Ethiopia]; région du Cap [South Africa, Western Cape].
= Metallea pseudoinflata Peris, 1951: 240. **Type locality**: Anglo-Egipsian Sudan [Sudan], W. Darfur, S. Jebel Murrs, Kallikitting, 4,4450 ft.

##### Distribution

**Figure 76.**
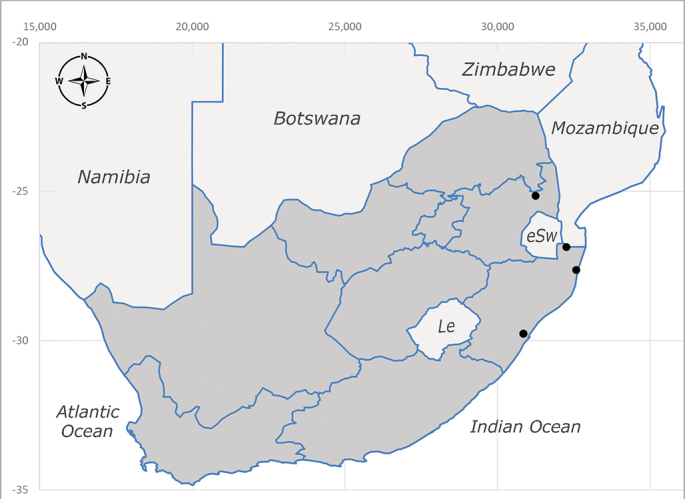
*Rhyncomyadasyops* occurrence map in South Africa, including eSwatini (eSw) and Lesotho (Le); horizontal axis longitude east and vertical axis latitude south.

**Afrotropical**: Angola, Benin, Botswana, Burundi*, Cameroon, Democratic Republic of Congo, Ethiopia, Gambia, Ghana, Kenya, Malawi, Mozambique, Namibia, Nigeria, South Africa (Fig. 76), Sudan, Uganda, Zambia and Zimbabwe.

##### Notes

**Figure 77.**
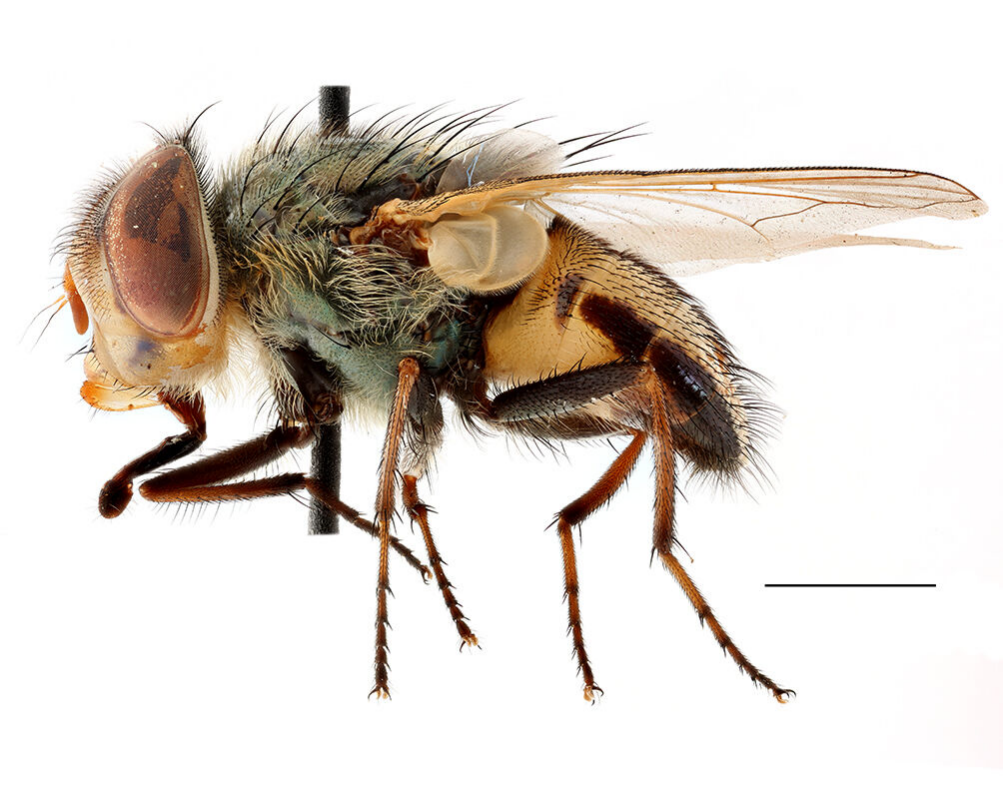
Habitus, left lateral view of *Rhyncomyadasyops* male NMSA DIP 84128 from South Africa; scale bar = 2 mm.

**Preferred environment**: in Namibia, restricted to the Arid and Mesic Savannah Biomes (Kurahashi and Kirk-Spriggs 2006). **Recorded elevations**: 556 m a.s.l. **Seasonality**: low numbers in November, December, January, April, May and July. In Namibia, low numbers too in October and December (Kurahashi and Kirk-Spriggs 2006). **Behaviour and ecology**: a female collected as prey of a robber fly (Asilidae) in August 1938 in the Gota Gota camp, Zimbabwe. Cuthbertson (1939) recorded specimens on flowers in Urungwe, Lomagundi District. **Life cycle and developmental stages**: unknown. **Collection methods**: in Namibia, with pitfall and Malaise traps (Kurahashi and Kirk-Spriggs 2006). **Illustrations and photographs**: male habitus as in Fig. 77. Male terminalia as in fig. 41–right in Zumpt (1958).

**Material examined**: Suppl. materials 1, 2.

#### Rhyncomya depressifrons

Villeneuve, 1927

6C9CEC83-E772-5AC8-831E-E80FD37BF92C

= Rhyncomya depressifrons Villeneuve, 1927: 24. **Type locality**: South Africa, Natal [KwaZulu-Natal], Estcourt. **Remarks**: STs in NHMUK.

##### Distribution

**Figure 78.**
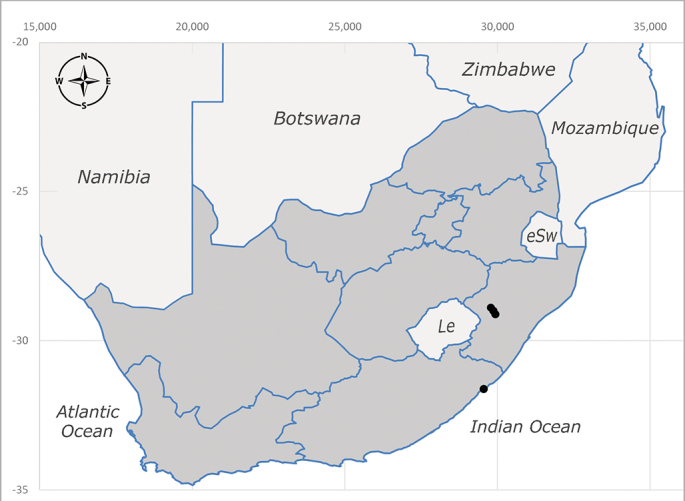
*Rhyncomyadepressifrons* occurrence map in South Africa, including eSwatini (eSw) and Lesotho (Le); horizontal axis longitude east and vertical axis latitude south.

**Afrotropical**: South Africa (Fig. 78).

##### Notes

**Figure 79.**
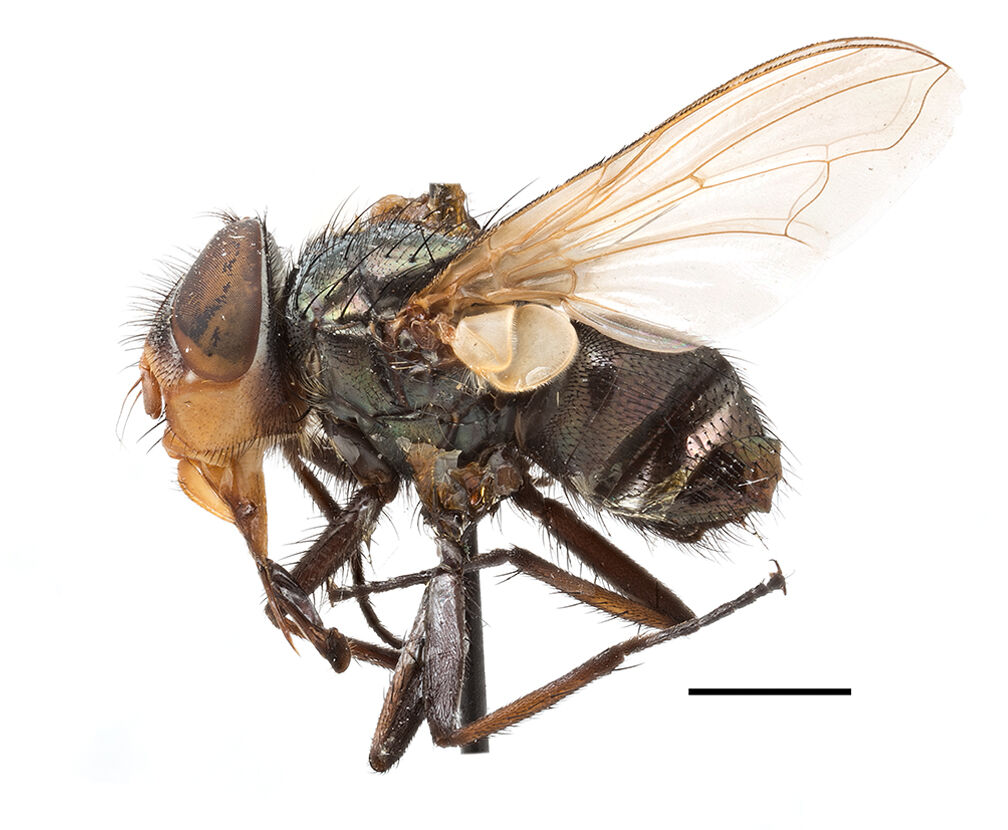
Habitus, left lateral view of *Rhyncomyadepressifrons* male NHMUK 010832207 ST (male without terminalia) (Copyright NHMUK); scale bar = 2 mm.

**Preferred environment**: no data. **Recorded elevations**: no data. **Seasonality**: only present from December to March. **Behaviour and ecology**: unknown. **Life cycle and developmental stages**: unknown. **Collection methods**: unknown. **Illustrations and photography**: male habitus as in Fig. 79. Male terminalia unknown.

**Type material examined**: *R.depressifrons*: 1 ? // SYN-TYPE // Natal / Estcourt / 5.ii.1913 / R.C. Wroughton // Slide no. 61 // *Rhyncomya* ? / *depressifrons* / Villeneuve // Slide prep.: / 010194477 // [NHMUK 010832207]. 1 ? // SYN-TYPE // Natal / Estcourt / 5.ii.1913 / R.C. Wroughton //Pres. by / Com. Inst. Ent. / BM. 1955-504 // [NHMUK 010832208]. *R.depressifrons*: 1 ? // SYN-TYPE // Natal / Estcourt / 26.i.1913 / R.C. Wroughton // Pres. by / Com. Inst. Ent. / B.M.1955-504 // *depressifrons* / typ. Villen. // [NHMUK 010832209].

**Material examined**: Suppl. material 1.

#### Rhyncomya disclusa

Villeneuve, 1927

2FD80948-2623-5C63-BD5E-BAB9E0C601F6

= Rhyncomya disclusa Villeneuve, 1927: 24. **Type locality**: South Africa, Natal [KwaZulu-Natal], Willow Grange. **Remarks**: STs in NHMUK.

##### Distribution

**Figure 80.**
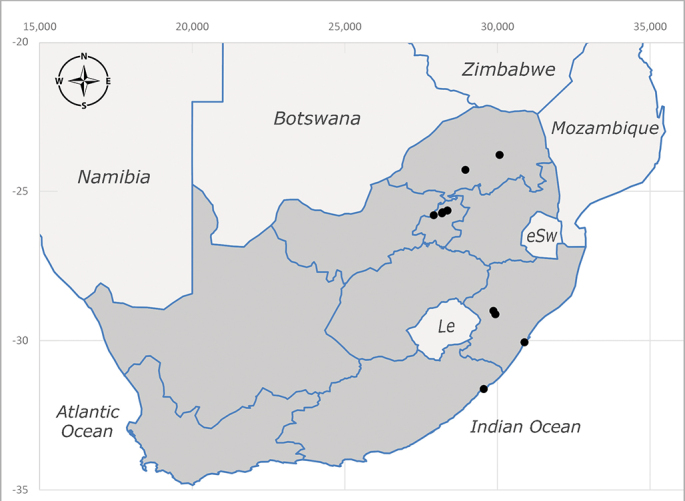
*Rhyncomyadisclusa* occurrence map in South Africa, including eSwatini (eSw) and Lesotho (Le); horizontal axis longitude east and vertical axis latitude south.

**Afrotropical**: ?Cameroon and South Africa (Fig. 80).

##### Notes

**Figure 81.**
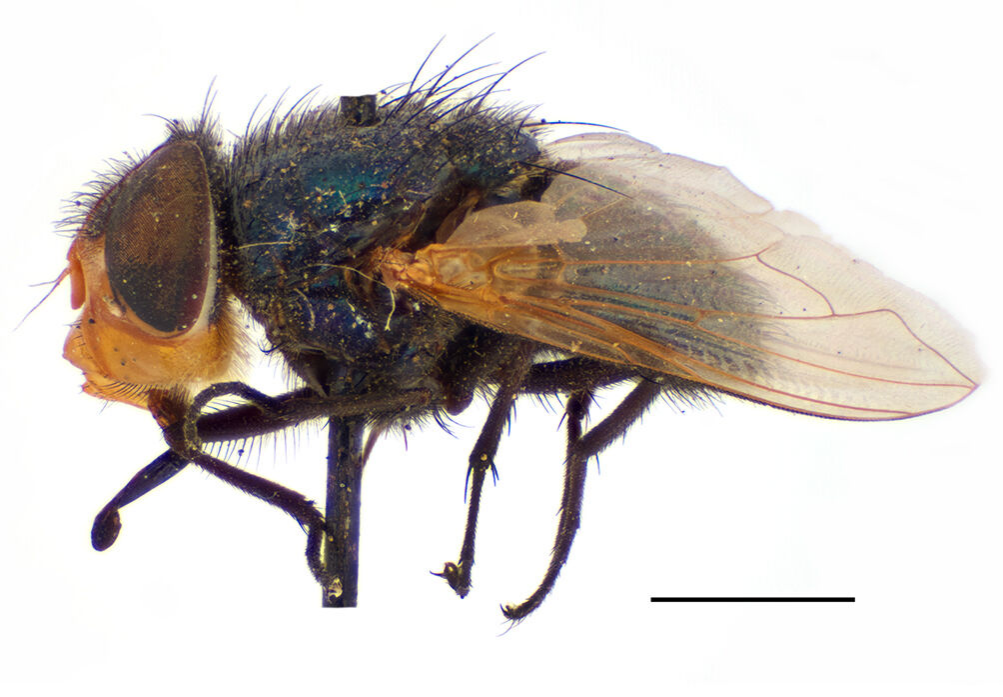
Habitus, left lateral view of *Rhyncomyadisclusa* male USNM-SM 01457916 from South Africa; scale bar = 2 mm.

**Preferred environment**: no data. **Recorded elevations**: no data. **Seasonality**: only present in March, September, October and December. **Behaviour and ecology**: reported as collected on flowers of *Acacia* Mill. and visiting flowers of onions. **Life cycle and developmental stages**: unknown. **Collection methods**: unknown. **Illustrations and photographs**: male habitus as in Fig. 81. Male terminalia unknown.

**Type material examined**: *R.disclusa*: 1 ? // SYN-TYPE // Natal / Willow Grange / Mooi River / R.C. Wroughton / 18.iii.1913 // Pres. by / Imp. Inst. Ent. / Brit. Mus. / 1933-14 // [NHMUK 010832216]. *R.disclusa*: 1 ? // SYN-TYPE // Natal / Willow Grange / Mooi River / R.C. Wroughton // slide no. 43 // [NHMUK 010832217]. *R.disclusa*: 1 ? // SYN-TYPE // Natal / Willow Grange / Mooi River / R.C. Wroughton // ?llBrook / 22.x.13 // Pres. by / Com. Inst. Ent. / B.M. 1955-504. // *Rhyncomyia* / Dr. Villeneuve det. / *disclusa* / Typ. Villen. // [NHMUK 010832218].

**Material examined**: Suppl. material 1.

#### Rhyncomya discrepans

Villeneuve, 1927

7A8D0BB6-DA32-5D0A-9CD8-79559496A7C3

= Rhyncomya discrepans Villeneuve, 1927: 22**. Type locality**: South Africa, Gt. Wint-hock, Tulbach, 3,600 ft.; and Cap [Eastern Cape], Algoa-bay. **Remarks**: LT in SAMC; the specimens are labelled as "lectotypus", apparently designated by Zumpt, with no data about the date.

##### Distribution

**Figure 82.**
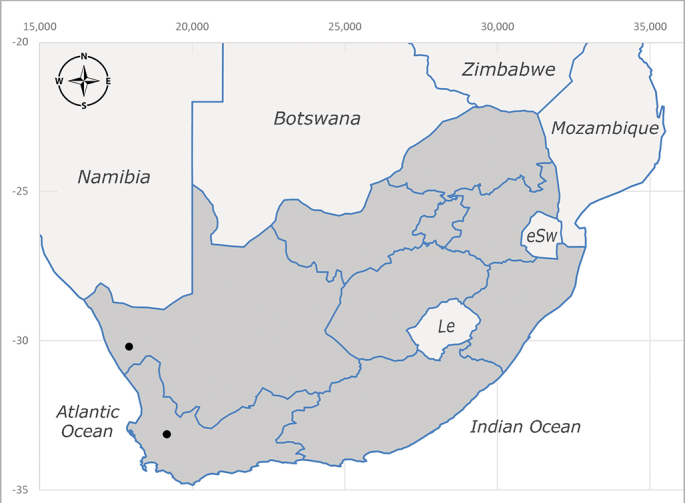
*Rhyncomyadiscrepans* occurrence map in South Africa, including eSwatini (eSw) and Lesotho (Le); horizontal axis longitude east and vertical axis latitude south.

**Afrotropical**: Namibia and South Africa (Fig. 82).

##### Notes

**Figure 83.**
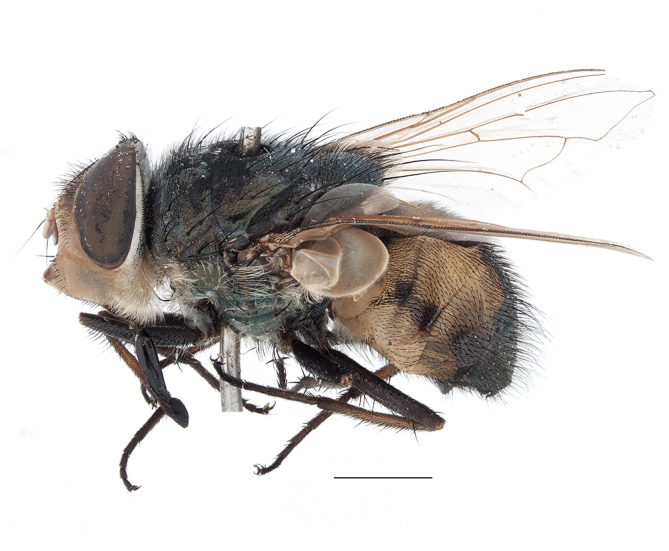
Habitus, left lateral view of *Rhyncomyadiscrepans* male SAMC DIP A011192 LT from Namibia; scale bar = 2 mm.

**Preferred environment**: in Namibia, the edge of the Nama-Karoo Biome (Kurahashi and Kirk-Spriggs 2006). **Recorded elevations**: 1097–1158 m a.s.l. **Seasonality**: peaking in April with six specimens, present in September and absent the rest of the year; in Nambia, only one specimen in November (Kurahashi and Kirk-Spriggs 2006). **Behaviour and ecology**: unknown. **Life cycle and developmental stages**: unknown. **Collection methods**: in Namibia, Malaise trap (Kurahashi and Kirk-Spriggs 2006). **Illustrations and photographs**: male habitus as in Fig. 83. Male terminalia as in fig. 50a in Zumpt (1958).

**Type material examined**: *R.discrepans*: 1 ? Gt.Wint-hoek Tulbagh 3600 ft // April1916 R.M.L // *discrepans* Typ. Villen. // *semihirta* Typ. Villen. // Lectotypus // slide no 52 // Slide SAM 33B13 [SAMC DIP A011192].

**Material examined**: Suppl. materials 1, 2.

#### Rhyncomya forcipata

Villeneuve, 1927

6492E038-5068-5BD9-8589-EDD321A0B832

= Rhyncomya forcipata Villeneuve, 1927: 17. **Type locality**: Northern Rhodesia [Zambia]; South Africa, Transvaal [Gauteng], Pretoria; and Zaire [Democratic Republic of Congo]. **Remarks**: ST in NHMUK.

##### Distribution

**Figure 84.**
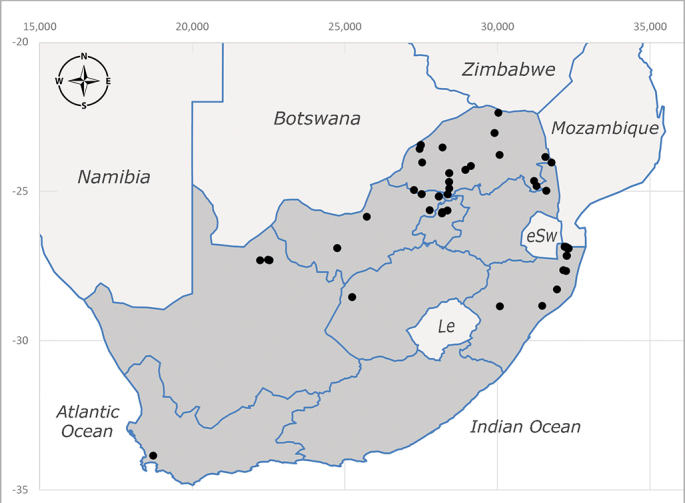
*Rhyncomyaforcipata* occurrence map in South Africa, including eSwatini (eSw) and Lesotho (Le); horizontal axis longitude east and vertical axis latitude south.

**Afrotropical**: Botswana, Democratic Republic of Congo, Gambia*, Kenya, Malawi, Mozambique, Namibia, Nigeria*, South Africa (Fig. 84), Tanzania, Uganda, Zambia and Zimbabwe.

##### Notes

**Figure 85.**
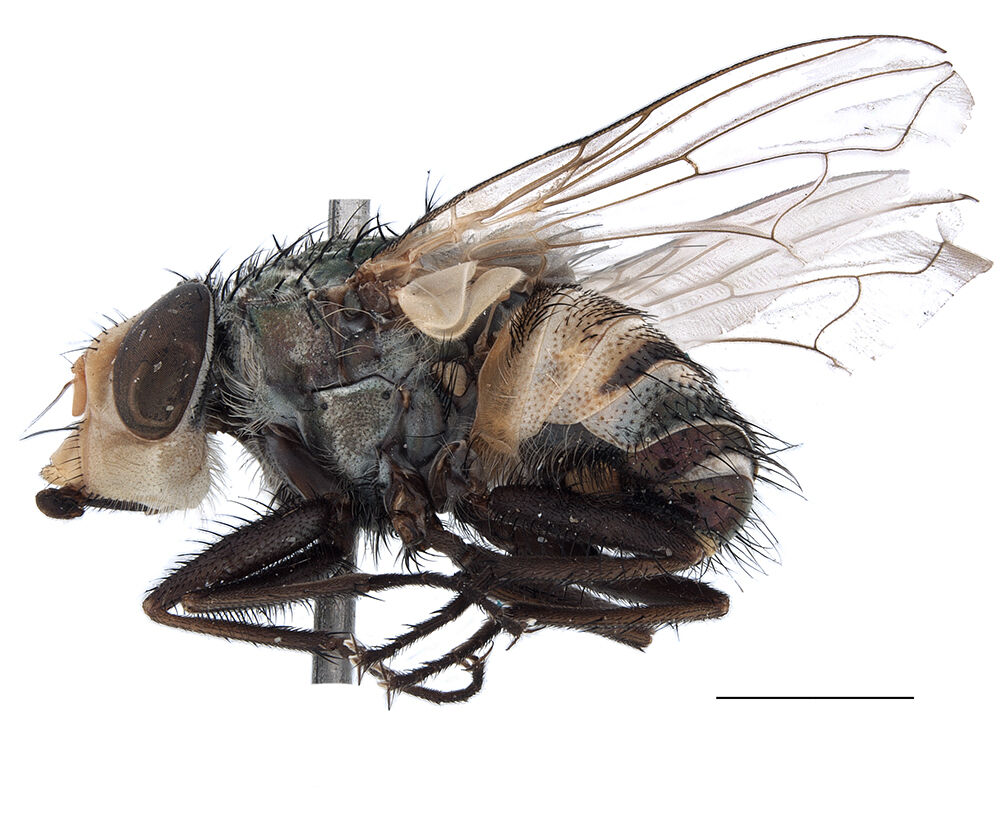
Habitus, left lateral view of *Rhyncomyaforcipata* male SAMC DIP A011194 from South Africa; scale bar = 2 mm.

**Preferred environment**: on red sand and directly associated with *Acacia* sp., *Boscia* Lam. ex J.St.-Hil. sp. and *Rhuslancea* L.fil savannah. Additionally, in broad-leafed deciduous woodland and forest, dry scrub (*Ficus*) riverine and sand forest, mixed Bushveld-grass and Kathu Bushveld to the Olifants River near Balule riparian woodland and Olifantshoek Plains Thornveld., Gordonia Plains Schrubland and Savanah Biome to Mesinda. In Kenya, the dry *Acacia* savannah, rocks and river margins. In Namibia, the indigenous and degraded sand forest, Miombo and Mopane Woodlands, Kwando River and open savannah floodplain and cultivated plots. Additionally, reported as restricted to Arid and Mesic Savannah Biome in Namibia (Kurahashi and Kirk-Spriggs 2006). **Recorded elevations**: 53–1320 m a.s.l. **Seasonality**: Abundant species, absent in January, July and August, highest abundance from October to December. Abundant in Namibia, peaking from December to March (Kurahashi and Kirk-Spriggs 2006). **Behaviour and ecology**: collected in *Senegaliamellifera*. Cuthbertson (1934) observed males at the blossom of trees and shrubs (*Gymnosporia* (Wight & Arn.) Hook.fil. (Celastraceae) and in the foliage of citrus trees infested with soft scales (Hemiptera, Coccidae) in Balla Balla (now Mbalabala), Zimbabwe. Females were observed depositing eggs around cattle-dung infested by termites in late afternoon (Kurahashi and Kirk-Spriggs 2006). **Life cycle and developmental stages**: unknown. **Collection methods**: sweep-net, Malaise and light traps. In Botswana and Kenya, with Malaise traps. In Zambia, ex-Malaise trap. In Namibia, using UV-light, by hand net, Malaise, yellow pans and pitfall traps (Kurahashi and Kirk-Spriggs 2006). **Illustrations and photographs**: male habitus as in Fig. 85. Male terminalia as in fig. 61 in Zumpt (1958).

**Type material examined**: *R.forcipata*: 1 ? // SYN-TYPE // Mid' / Luangara Koller / N.E. Rhodesia / Aug. 28.1910 / S.A. Neave // Pres. by / Com. Inst. Ent. / B.M. 1955-504 // *Rhyncomyia* / *forcipata* / Typ. Villen. // [NHMUK 010832190].

**Material examined**: Suppl. materials 1, 2.

#### Rhyncomya fovealis

Bezzi, 1908

C59C3644-64EC-53D0-8219-06C82CD417DB

= Rhyncomya fovealis Bezzi, 1908: 188**. Type locality**: South Africa, Port Nolloth, Klein Namaland.

##### Distribution

**Afrotropical**: South Africa.

##### Notes

No specimens examined for South Africa, based on Pont (1980). **Illustrations and photographs**: unavailable.

#### Rhyncomya hessei

Zumpt, 1958

1A669251-5B19-54D8-B205-16350D0EB30E

= Rhyncomya hessei Zumpt, 1958: 150. **Type locality**: South Africa, C. P. [Cape Province - Western Cape], Dikbome, Merweville Koup. **Remarks**: PTs and HT in SAMC and PTs in NMSA.

##### Distribution

**Figure 86.**
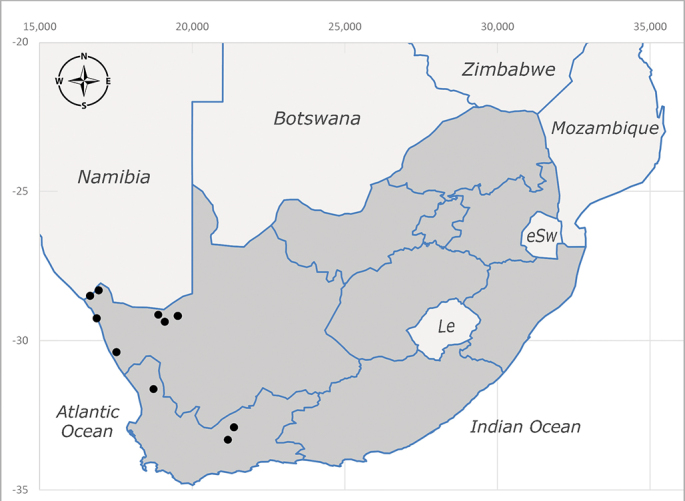
*Rhyncomyahessei* occurrence map in South Africa, including eSwatini (eSw) and Lesotho (Le); horizontal axis longitude east and vertical axis latitude south.

**Afrotropical**: Mozambique, Namibia and South Africa (Fig. 86).

##### Notes

**Figure 87.**
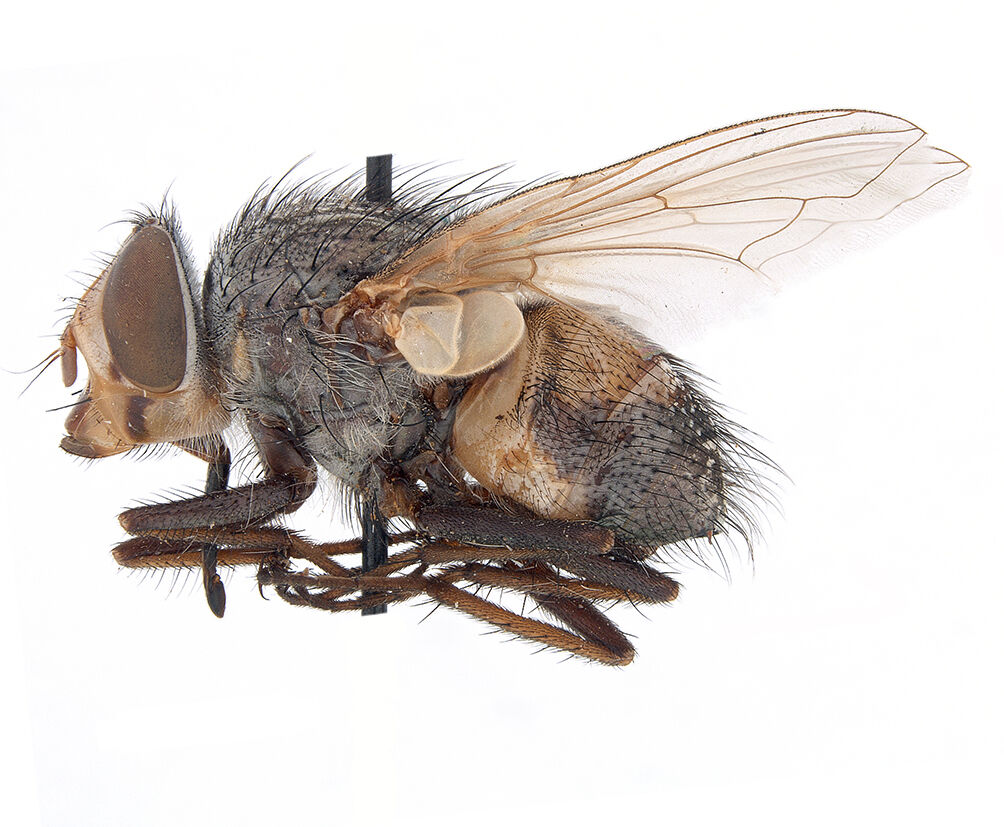
Habitus, left lateral view of *Rhyncomyahessei* male SAMC DIP A011196 HT from South Africa (without terminalia); scale bar = 2 mm.

**Preferred environment**: flowers at the roadsides, rocky hillsides and sandy and rocky ridges with succulents. In Namibia, virtually restricted to the semi-arid region, Nama-Karoo and Succulent Karoo Biomes (Kurahashi and Kirk-Spriggs 2006). **Recorded elevations**: 90–817 m a.s.l. **Seasonality**: present only in March, August, September and October, peaking in September. In Namibia, most abundant in August and September (Kurahashi and Kirk-Spriggs 2006). **Behaviour and ecology**: unknown. **Life cycle and developmental stages**: unknown. **Collection methods**: in Namibia, with Malaise, yellow pan and pitfall traps and at UV-light (Kurahashi and Kirk-Spriggs 2006). **Illustrations and photographs**: male habitus as in Fig. 87. Male terminalia as in fig. 47 in Zumpt (1958).

**Type material examined**: *R.hessei*: 1 ? // HOLOTYPUS // Dikbome / Merweville / Koup / C.P. // Mus. Expd. / Oct. 1952 // slide no. 94 // // *Rhyncomyia* ? / *hessei* n.sp. / Det. Zumpt '56 // Slide / SAM: / 33B9 // [SAMC DIP A011196]. 2 ?? // PARATYPE // Wallekraal / Namaqualand // Mus., Expd. / Oct. 1950. // *Rhyncomyia* ? / *hessei* n.sp. / Det. Zumpt 1956 // [SAMC DIP A015139, A015140]. 1 ? // PARATYPE // Wallekraal / Namaqualand // *Rhyncomyia* ? / *hessei* n.sp. / Det. Zumpt 1956 [NMASA-DIP 19954]. 1 ? // PARATYPE // Wallekraal / Namaqualand // Mus., Expd. / Oct. 1950. // slide no. 92 // *Rhyncomyia* ? / *hessei* n.sp. / Det. Zumpt 1956 [SAMC DIP A011197]. 1 ? // PARATYPE // Vogelfontein / P. Albert Div. // A. J. Hesse / Mar.-Apr. 1929 // ? term. 4 // *Rhyncomyia* / *hessei* n. sp. / Zumpt 1956 // [SAM-DIP A011198].

**Material examined**: Suppl. materials 1, 2.

#### Rhyncomya inflata

(Robineau-Desvoidy, 1830)

FB24A003-897F-5180-939A-4440A8F32F8F

= Beria inflata Robineau-Desvoidy, 1830: 418. **Type locality**: Cape [South Africa].
= Rhyncomya elegantula Villeneuve, 1927: 20. **Type locality**: Congo Belge [Democratic Republic of Congo], Kashiobwe.

##### Distribution

**Afrotropical**: Democratic Republic of Congo, Mozambique* and South Africa.

##### Notes

**Figure 88.**
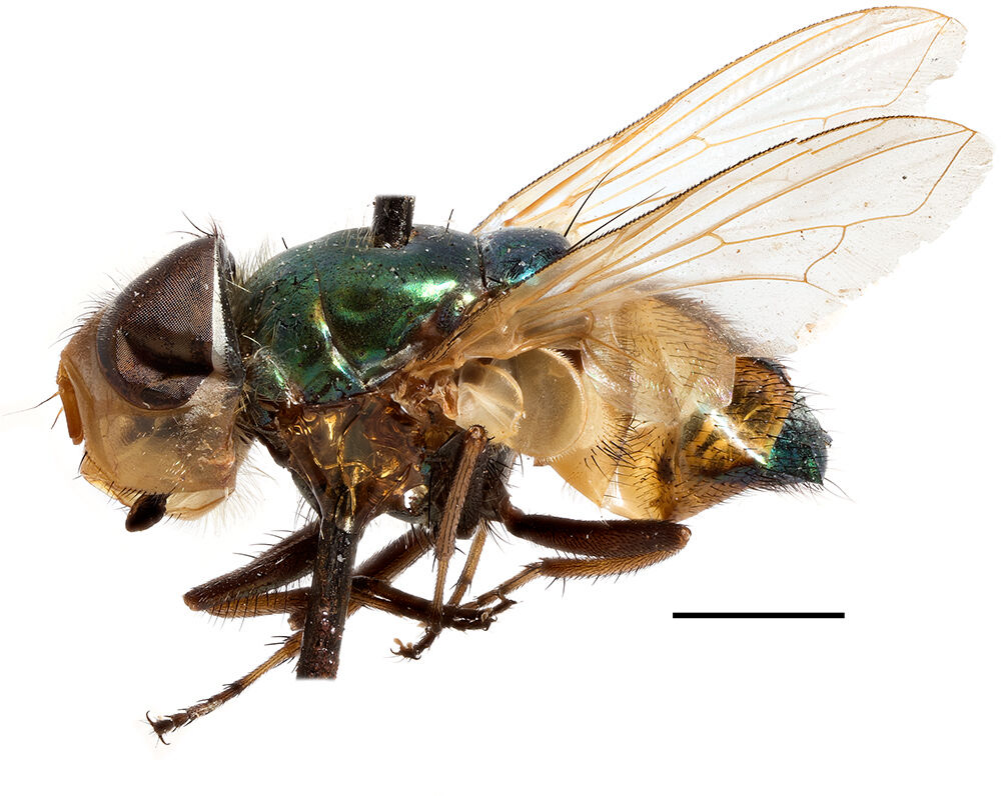
Habitus, left lateral view of *Rhyncomyainflata* male NMSA DIP 19959 from Mozambique (without terminalia); scale bar = 2 mm.

No specimens examined, based on *Pont (1980)*. **Illustrations and photographs**: male habitus as in Fig. 88. Male terminalia as in fig. 43 in Zumpt (1958).

**Material examined**: Suppl. material 2.

#### Rhyncomya interclusa

Villeneuve, 1920

EFCAC215-D510-5CEC-91F0-645363317060

= Rhyncomya interclusa Villeneuve, 1920: 160. **Type locality**: South Africa, Western Cape [Capland], Willowmore. **Remarks**: PT in NMSA and PLT (see Zumpt 1958) in NHMUK.

##### Distribution

**Figure 89.**
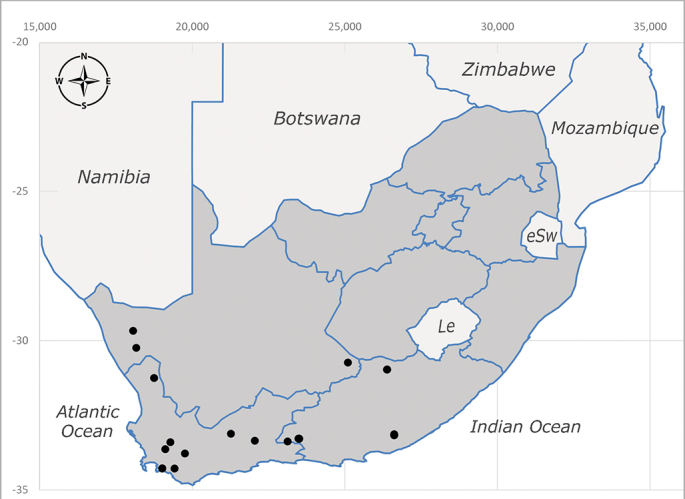
*Rhyncomyainterclusa* occurrence map in South Africa, including eSwatini (eSw) and Lesotho (Le); horizontal axis longitude east and vertical axis latitude south.

**Afrotropical**: Angola*, Botswana*, Namibia and South Africa (Fig. 89).

##### Notes

**Figure 90.**
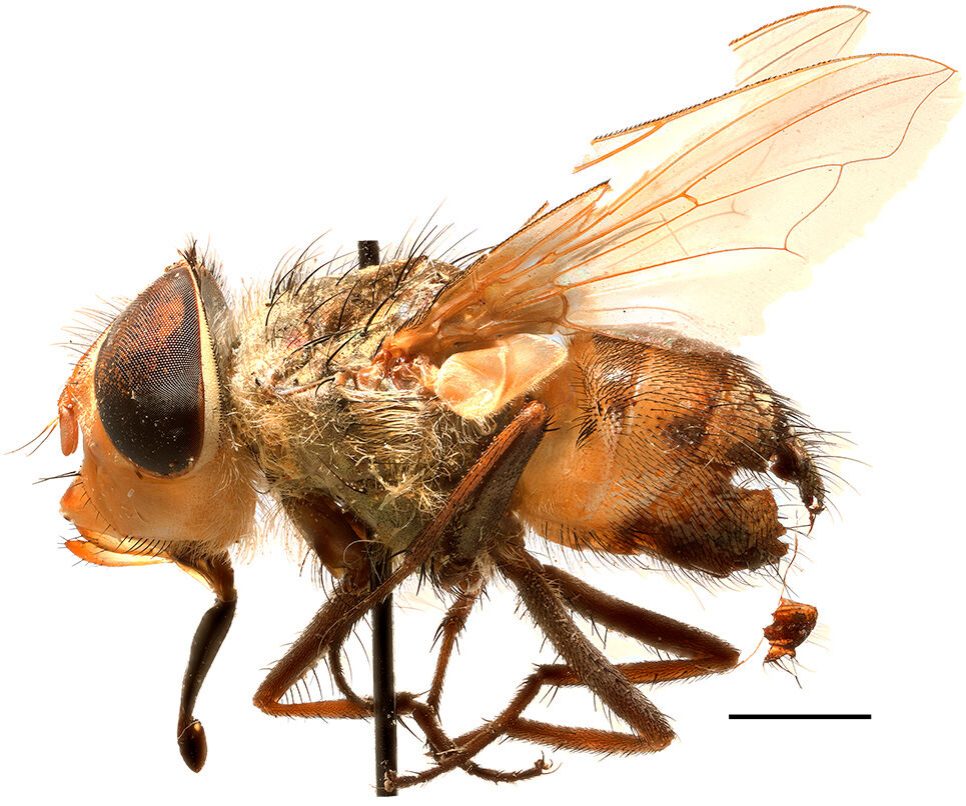
Habitus, left lateral view of *Rhyncomyainterclusa* male NMSA DIP 19960 LT from South Africa (without terminalia); scale bar = 2 mm.

**Preferred environment**: mesic Mountain Fynbos to plains. In Namibia, apparently restricted to the Desert Biome (Kurahashi and Kirk-Spriggs 2006). **Recorded elevations**: 250–1524 m a.s.l. **Seasonality**: peaking in October-November, absent during the coolest months (June, July and August). **Behaviour and ecology**: on *Ruschiarobusta* L. Bolus pink flowers. **Life cycle and developmental stages**: unknown. **Collection methods**: Malaise and yellow pan traps. **Illustrations and photographs**: male habitus as in Fig. 90. Male terminalia as in fig. 50b in Zumpt (1958).

**Type material examined**: *R.interclusa*: 1 ? // PARA- / LECTO- / TYPE / Capland / Willowmore, Mai 5 1920 / Dr. Brauns // Pres By / Com Inst Ent / B M 1953-354 // *Rhyncomyia* / *interclusa* / Villen. // Paralectotype ? / See Zumpt, 1958 / Explor Parq Nati. / Albert Miss. G.F. / de Witte, 92:156 // [NHMUK 010832194]. 1 ? // Capland / Willowmore / März 1926 / Dr. H. Brauns // PARATYPE // *Rhyncomyia* ? / *interclusa* Vill. / det. Zumpt // [NMSA-DIP 19961].

**Material examined**: Suppl. materials 1, 2.

#### Rhyncomya maculata

Macquart, 1846

B3833533-0D0D-5064-902F-F4F17392278E

= Rhyncomyia maculata Macquart, 1846: 322. **Type locality**: Cape [South Africa].

##### Distribution

**Figure 91.**
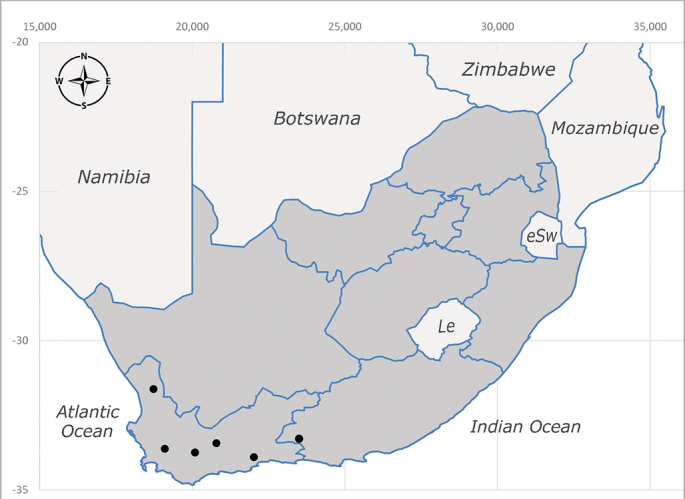
*Rhyncomyamaculata* occurrence map in South Africa, including eSwatini (eSw) and Lesotho (Le); horizontal axis longitude east and vertical axis latitude south.

**Afrotropical**: South Africa (Fig. 91).

##### Notes

**Figure 92.**
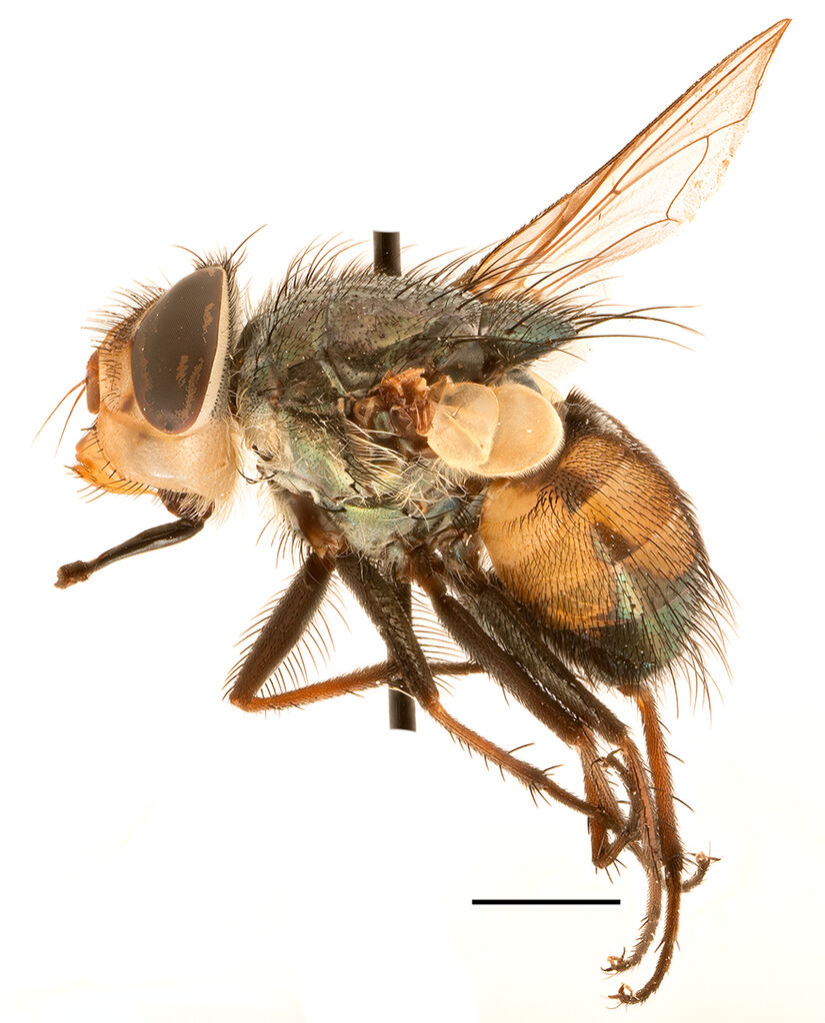
Habitus, left lateral view of *Rhyncomyamaculata* male MZSUR from South Africa; scale bar = 2 mm.

**Preferred environment**: no data. **Recorded elevations**: 479–747 m a.s.l. **Seasonality**: present August-October, December-January and May, rest of the year absent. **Behaviour and ecology**: unknown. **Life cycle and developmental stages**: unknown. **Collection methods**: hand net with hilltopping. **Illustrations and photographs**: male habitus as in Fig. 92. Male terminalia as in fig. 49 in Zumpt (1958).

**Material examined**: Suppl. material 1.

#### Rhyncomya messoria

Villeneuve, 1927

DD18FDFB-086B-59DB-8B50-C757734B5C5E

= Rhyncomya messoria Villeneuve, 1927: 25. **Type locality**: Congo Belge [Democratic Republic of Congo], Kilwa; South Africa, Cape of Good Hope [Western Cape]. **Remarks**: HT in RMCA.
= Beria erula Séguy, 1933: 68. **Type locality**: Oriental Portuguese East Africa [Mozambique], Moulima.

##### Distribution

**Figure 93.**
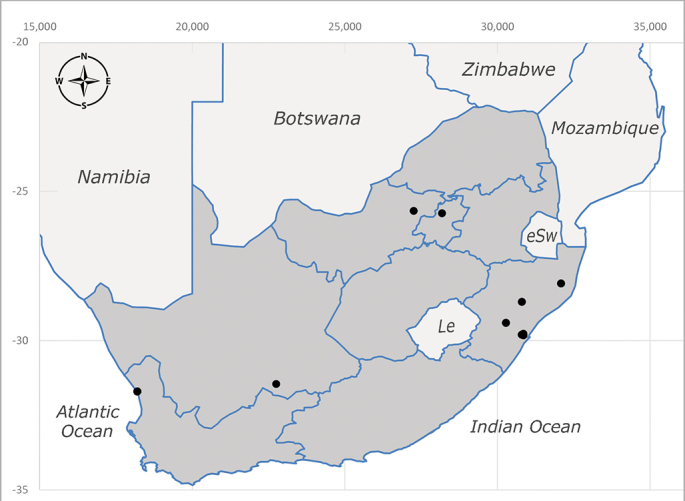
*Rhyncomyamessoria* occurrence map in South Africa, including eSwatini (eSw) and Lesotho (Le); horizontal axis longitude east and vertical axis latitude south.

**Afrotropical**: Botswana, Democratic Republic of Congo, Kenya, Lesotho, Mozambique, Namibia, South Africa (Fig. 93), Tanzania and Zimbabwe.

##### Notes

**Figure 94.**
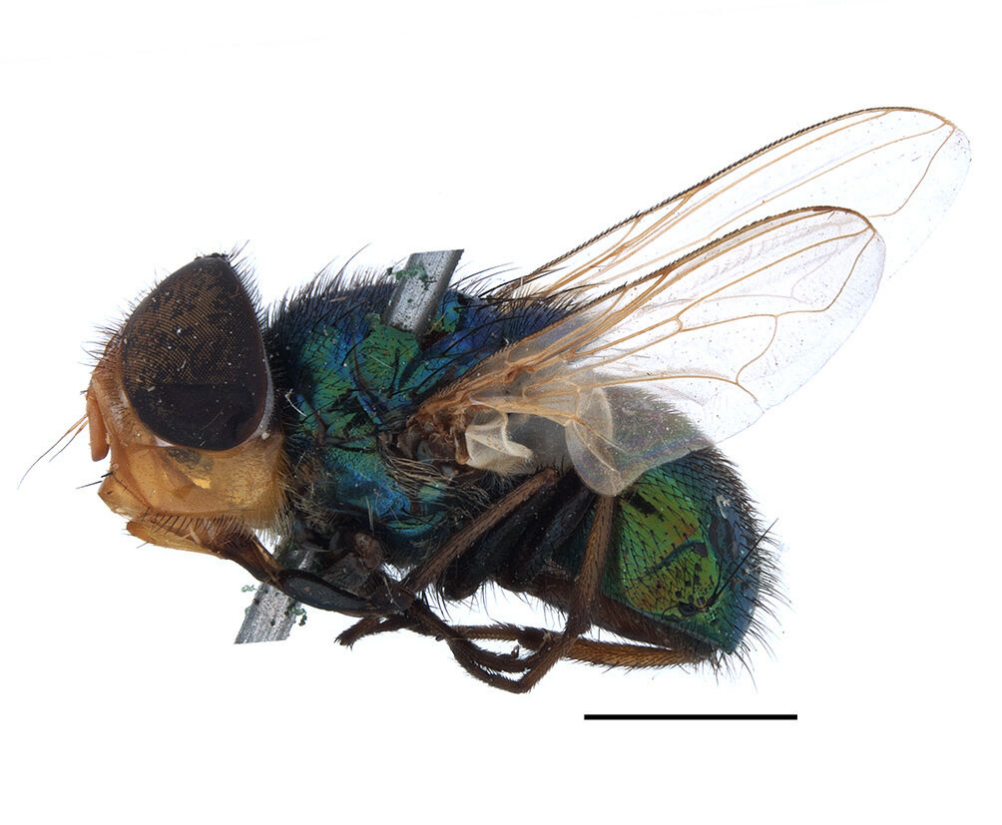
Habitus, left lateral view of *Rhyncomyamessoria* male SAMC DIP A011207 from South Africa; scale bar = 2 mm.

**Preferred environment**: in Namibia, the Arid and Mesic Savannah and Desert Biomes (Kurahashi and Kirk-Spriggs 2006). **Recorded elevations**: no data. **Seasonality**: present in January, February, April, October and peaking in December, rest of the year absent. In Namibia, low numbers from May to December (Kurahashi and Kirk-Spriggs 2006). **Behaviour and ecology**: unknown. **Life cycle and developmental stages**: unknown. **Collection methods**: in Namibia, hand net, pitfall, yellow pan and Malaise traps (Kurahashi and Kirk-Spriggs 2006). **Illustrations and photographs**: male habitus as in Fig. 94. Male terminalia as in fig. 46 in Zumpt (1958).

**Type material examined**: *R.messoria*: 1 ? // HOLOTYPUS // COLL. MUS. CONGO / Kilwa / (Dr.: J. Bequaert.) // *Rhyncomyia* / *messoria* / Typ. Villen. // Kilwa / 28- XII.11 // [RMCA ENT 000012156].

**Material examined**: Suppl. materials 1, 2.

#### Rhyncomya minutalis

Villeneuve, 1927

A38C4C3C-B9F9-5427-B01A-52E909A08785

= Rhyncomya minutalis Villeneuve, 1927: 22. **Type locality**: Cape [South Africa].

##### Distribution

**Figure 95.**
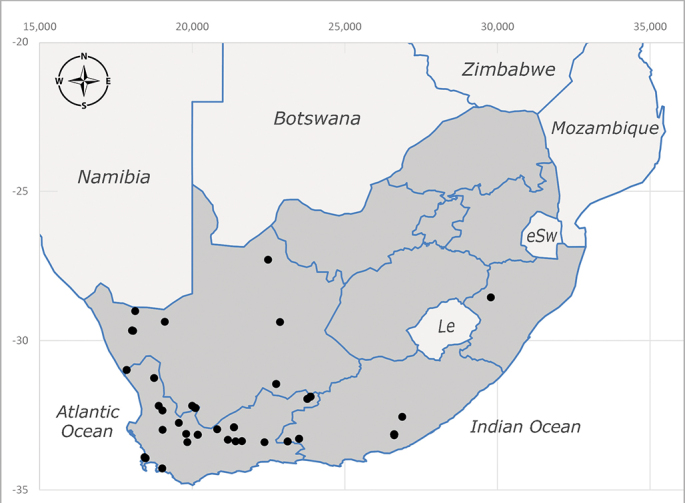
*Rhyncomyaminutalis* occurrence map in South Africa, including eSwatini (eSw) and Lesotho (Le); horizontal axis longitude east and vertical axis latitude south.

**Afrotropical**: Namibia and South Africa (Fig. 95).

##### Notes

**Figure 96.**
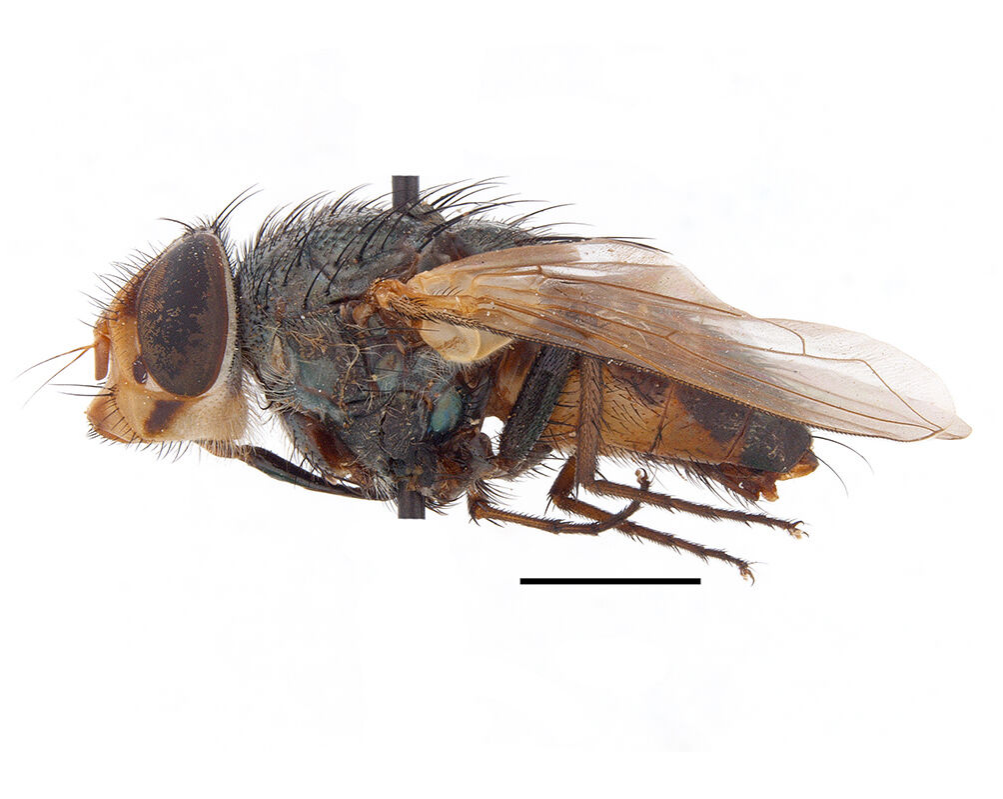
Habitus, left lateral view of *Rhyncomyaminutalis* male SAMC DIP A015144 from South Africa (without terminalia); scale bar = 2 mm.

**Preferred environment**: *Acacia* Karoo thicket, succulent Karoo, Karoo vegetation, Tanqua succulent Karoo; rocky sandy in Fynbos area, sandy bank and Olifantshoek Plains Thornveld; Savannah Biome and Macchia with Proteas. More or less restricted to the hyper-arid zone in Namibia (Kurahashi and Kirk-Spriggs 2006). **Recorded elevations**: 238–1245 m a.s.l. **Seasonality**: abundant throughout the year, peaking in October, but absent in July. In Namibia, most abundant in June and August (Kurahashi and Kirk-Spriggs 2006). **Behaviour and ecology**: unknown. **Life cycle and developmental stages**: unknown. **Collection methods**: hand net, Malaise and pitfall traps. In Namibia with Malaise, yellow pan and pitfall traps (Kurahashi and Kirk-Spriggs 2006). **Illustrations and photographs**: male habitus as in Fig. 96. Male terminalia as in fig. 48 in Zumpt (1958).

**Material examined**: Suppl. materials 1, 2.

#### Rhyncomya nana

Peris, 1951

EC9772EB-DAAC-5CEC-9A13-A17F02F2557D

= Rhyncomyia nana Peris, 1951: 240. **Type locality**: South Africa, Transvaal [?Gauteng], Plaas Kopernijn. **Remarks**: HT and PT in NHMUK.

##### Distribution

**Figure 97.**
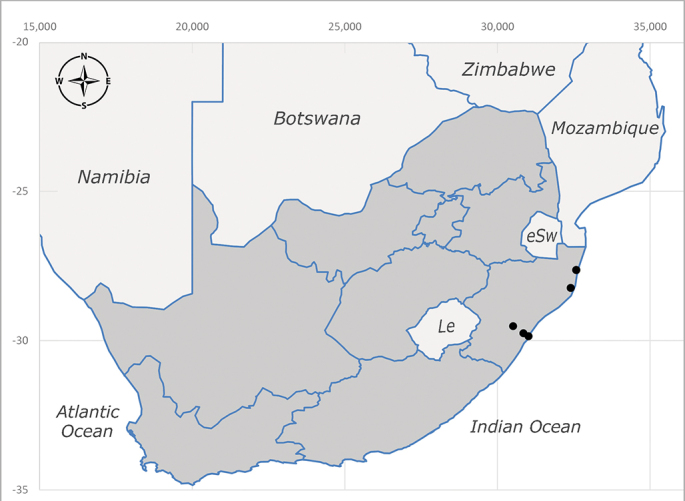
*Rhyncomyanana* occurrence map in South Africa, including eSwatini (eSw) and Lesotho (Le); horizontal axis longitude east and vertical axis latitude south.

**Afrotropical**: Mozambique and South Africa (Fig. 97).

##### Notes

**Figure 98.**
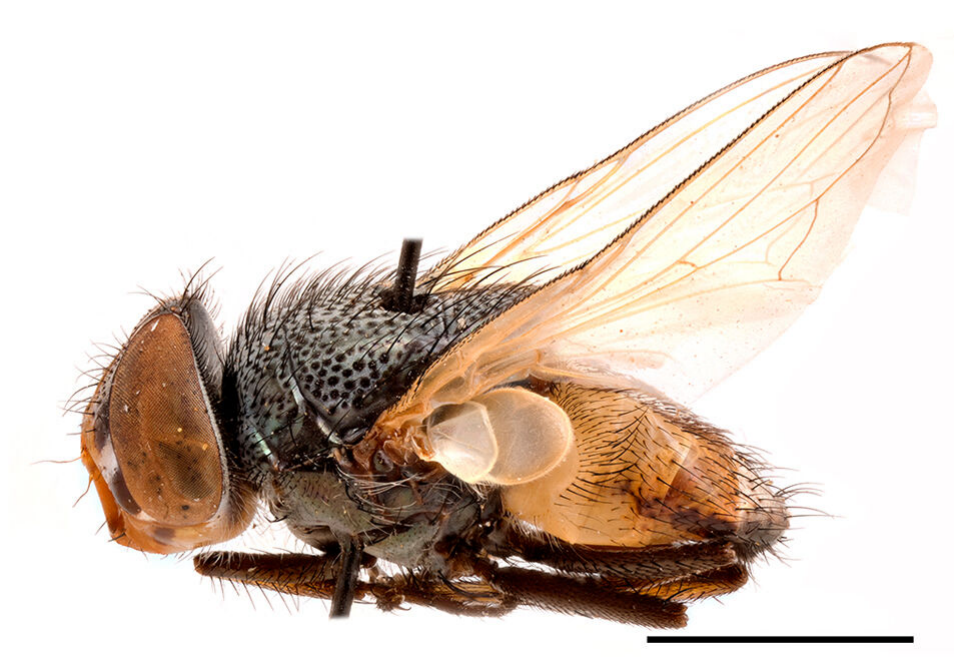
Habitus, left lateral view of *Rhyncomyanana* male NHMUK 010832203 HT from South Africa (Copyright NHMUK); scale bar = 2 mm.

**Preferred environment**: coastal grassland, open *Acacia* woodland with thick grass-cover, adjoining thicker valley bush and dry forest; subtropical grassland and open lala-palm woodland. **Recorded elevations**: 35–830 m a.s.l. **Seasonality**: low numbers in April, July, November and December, absent the rest of the year. **Behaviour and ecology**: unknown. **Life cycle and developmental stages**: unknown. **Collection methods**: sweeping hand net and with Malaise trap. In Mozambique, by sweeping hand net. **Illustrations and photographs**: male habitus as in Fig. 98. Male terminalia as in fig. 57 in Zumpt (1958).

**Type material examined**: *R.nana*: 1 ? // Holo- / type // Plaas Kopesrijn / J.J. Rust Tvl / 19.vii.1924 // Rhyncomyia/?nana n. sp S-V. Peris det. / Tipo // [NHMUK 010832203]. 1 ? // Para-type // Durban. / F. Muir. / 1905-313. // Sharp Coll. / 1905-313 // *Rhyncomyia* / *nana* n. sp / S-V. Peris det. / Paratipo // [NHMUK 010832204].

**Material examined**: Suppl. materials 1, 2.

#### Rhyncomya paradoxa

Zumpt, 1958

CBBBD705-A9DA-5496-B3B2-370998746DC5

= Rhyncomya paradoxa Zumpt, 1958: 159. **Type locality**: South Africa, Cape Province [Eastern Cape], Resolution, Albany District. **Remarks**: HT in NMSA, PTs in SAMC and SANC at ARC.

##### Distribution

**Figure 99.**
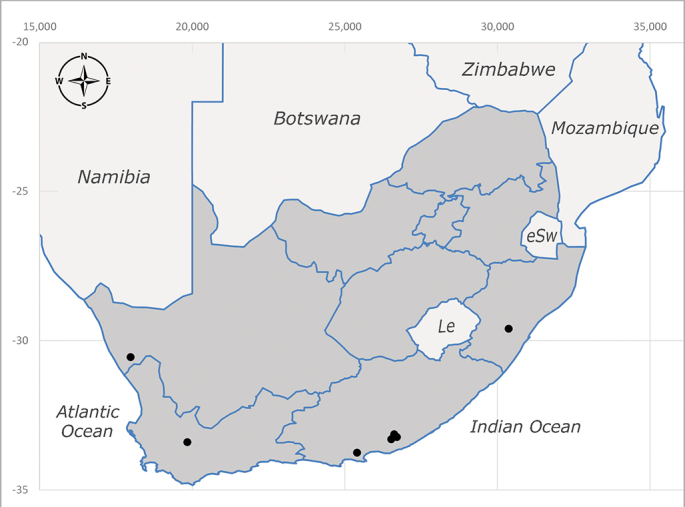
*Rhyncomyaparadoxa* occurrence map in South Africa, including eSwatini (eSw) and Lesotho (Le); horizontal axis longitude east and vertical axis latitude south.

**Afrotropical**: South Africa (Fig. 99).

##### Notes

**Figure 100.**
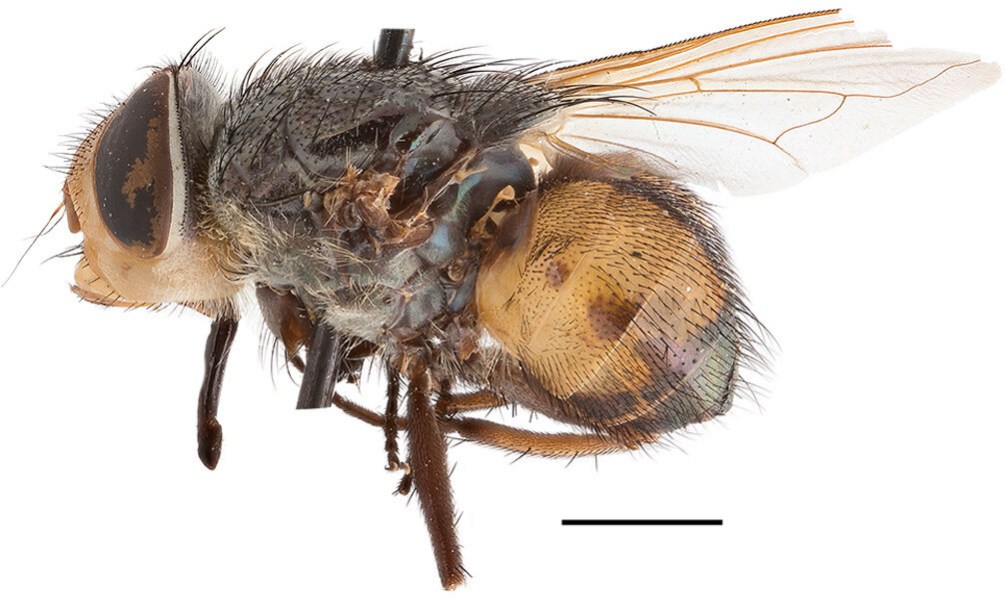
Habitus, left lateral view of *Rhyncomyaparadoxa* male SANC 00302 PT from South Africa (without terminalia); scale bar = 2 mm.

**Preferred environment**: no data. **Recorded elevations**: 958 m a.s.l. **Seasonality**: low numbers, present only in February, March, June and December. **Behaviour and ecology**: two males were collected from a nest of *Dasyproctusbraunsii* and *Dasyproctus ?nificanidis* (Hymenoptera). **Life cycle and developmental stages**: unknown. **Collection methods**: unknown. **Illustrations and photography**: male habitus as in Fig. 100. Male terminalia as in fig. 50c in Zumpt (1958).

**Type material examined**: *R.paradoxa*: 1 ? //Holotype // Resolution / Albany District / 21.ii.1928 / A. Walton // slide no. 88 // [NMSA-DIP 20008] - NMSA Type 2077. 1 ? // Garies / Namaqualand // Museum Staff /June 1930 // slide no. 89 // PARATYPE // *Rhyncomyia* ? / *paradoxa* n.sp. / Zumpt, 1956 // Slide number / SAM 4A9 // [SAM-DIP A011222]. 1 ? // Uitenhage / De Hoek / 1x.3.19 / H.K. Munro // PARATYPE // slide no.60 // *Rhyncomyia* ? / *paradoxa* n. sp / det. Zumpt 56 // SANC-Pretoria / Database No. / DIPT00302 // "Slide not available in the collection".

**Material examined**: Suppl. material 1.

#### Rhyncomya paratristis

Zumpt and Stimie, 1965

24833E90-876E-594A-AD0C-42468F422012

= Rhyncomya paratristis Zumpt and Stimie, 1965: 9. **Type locality**: South Africa, Zululand Natal [KwaZulu-Natal], Ndumu Reserve, Ingwavuma District. **Remarks**: HT and PTs in NMSA.

##### Distribution

**Figure 101.**
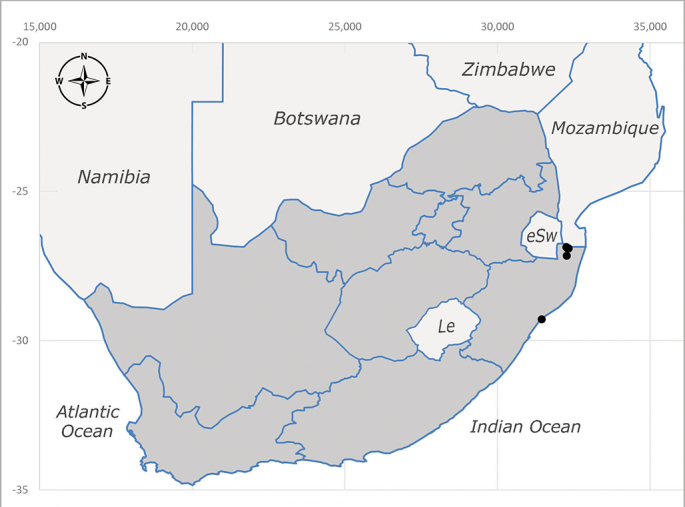
*Rhyncomyaparatristis* occurrence map in South Africa, including eSwatini (eSw) and Lesotho (Le); horizontal axis longitude east and vertical axis latitude south.

**Afrotropical**: South Africa (Fig. 101).

##### Notes

**Figure 102.**
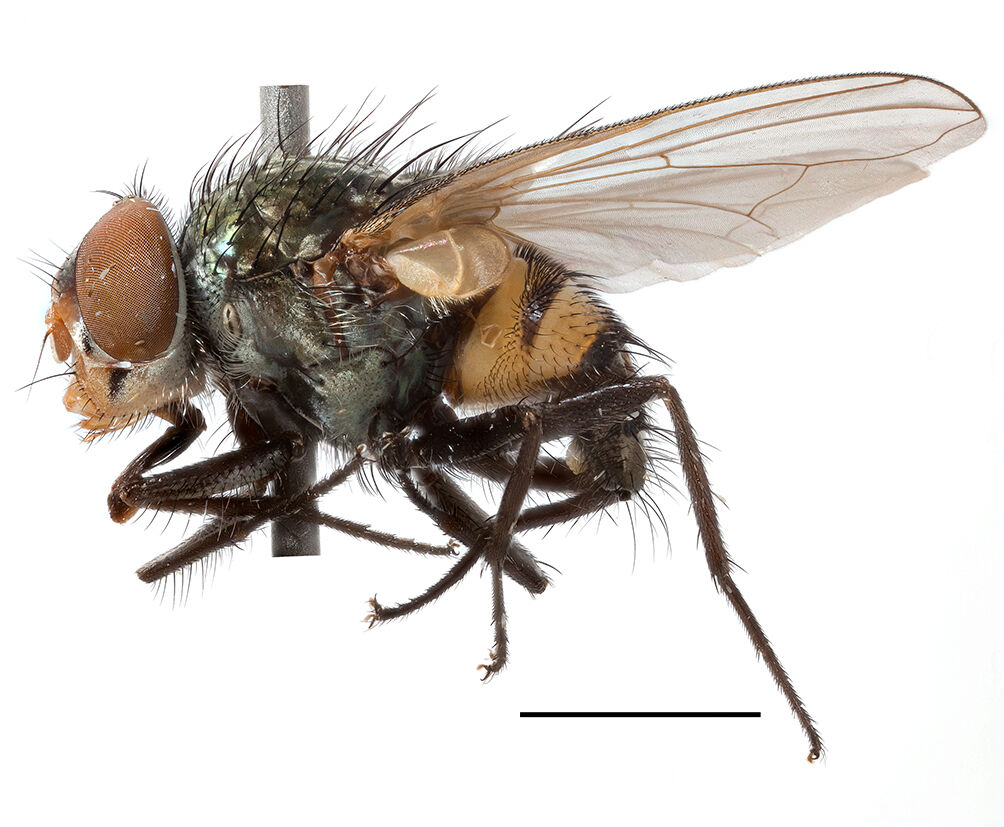
Habitus, left lateral view of *Rhyncomyaparatristis* male BMSA DIP 13688 from South Africa; scale bar = 2 mm.

**Preferred environment**: broad-leafed deciduous woodland, sand forest and broad-leafed deciduous forest, dry scrub forest, grassy floodplain and at the margin of dune forest. **Recorded elevations**: 10–98 m a.s.l. **Seasonality**: abundant, present in June and November, peaking in December. **Behaviour and ecology**: unknown. **Life cycle and developmental stages**: unknown. **Collection methods**: Malaise traps. **Illustrations and photographs**: male habitus as in Fig. 102. Male terminalia as in fig. 4 in Zumpt (1965).

**Type material examined**: *R.paratristis*: 2 ?? // HOLOTYPE // Ndumu Reserve / Ingwavuma dist. / Zululand, Natal / South Africa / 1-10.xii.63 // collectors / B. & P. Stuckenberg // *Rhyncomya* ? / *paratristis* / Zumpt & Stimie // [NMSA-DIP 074955, 074956]. 2 ?? // PARATYPE // Ndumu Reserve / Ingwavuma dist. / Zululand, Natal / South Africa / 1-10.xii.63 // collectors / B. & P. Stuckenberg // *Rhyncomya* ? / *paratristis* / Zumpt & Stimie // [NMSA-DIP 061694, 074957].

**Material examined**: Suppl. material 1.

#### Rhyncomya peraequa

Villeneuve, 1929

6B534842-C637-5593-858F-9354F711D5B2

= Rhyncomya peraequa Villeneuve, 1929: 186. **Type locality**: South Africa, Transvaal [Mpumalanga], Barberton. **Remarks**: HT in SAMC.

##### Distribution

**Figure 103.**
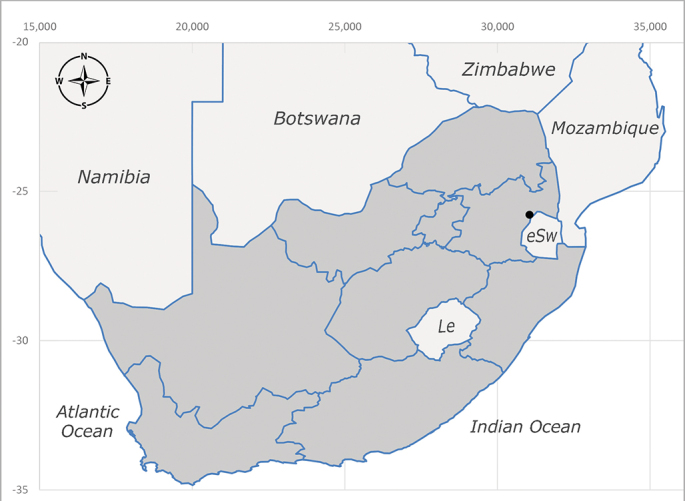
*Rhyncomyaperaequa* occurrence map in South Africa, including eSwatini (eSw) and Lesotho (Le); horizontal axis longitude east and vertical axis latitude south.

**Afrotropical**: Angola, Kenya, Namibia, South Africa (Fig. 103), Tanzania and Uganda.

##### Notes

**Figure 104.**
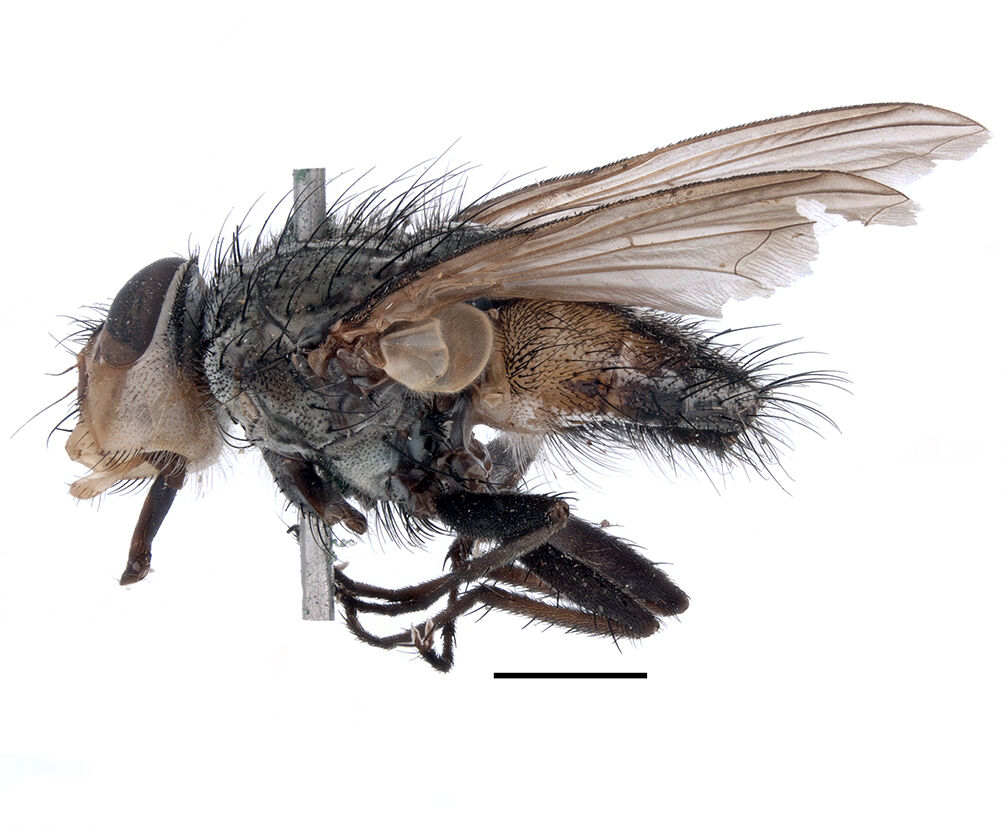
Habitus, left lateral view of *Rhyncomyaperaequa* male SAMC DIP A011223 HT from South Africa; scale bar = 2 mm.

**Preferred environment**: all Namibian biomes, except desert (Kurahashi and Kirk-Spriggs 2006). **Recorded elevations**: no data. **Seasonality**: only one specimen in December. In Namibia, most abundant in November and absent in February and March (Kurahashi and Kirk-Spriggs 2006). **Behaviour and ecology**: unknown. **Life cycle and developmental stages**: unknown. **Collection methods**: in Namibia with yellow pan, pitfall and Malaise traps and attracted to UV-light (Kurahashi and Kirk-Spriggs 2006). **Illustrations and photographs**: male habitus as in Fig. 104. Male terminalia as in fig. 2 in Zumpt (1974).

**Type material examined**: *R.peraequa*: 1 ? // Type // Barberton / Transvaal / H Edwards // Dec / 1911 // Type // *Rhyncomyia* / *peraequa* / Type Villen //[SAMC DIP A011223].

**Material examined**: Suppl. materials 1, 2.

#### Rhyncomya pruinosa

Villeneuve, 1922

6CC5AD93-75DB-5C21-B07B-AE4623B5A72E

= Rhyncomya pruinosa Villeneuve, 1922: 65. **Type locality**: Kenya, Zaire [Democratic Republic of Congo], Nyasaland [Malawi]; Anglo-Egipsian Sudan [Sudan].

##### Distribution

**Figure 105.**
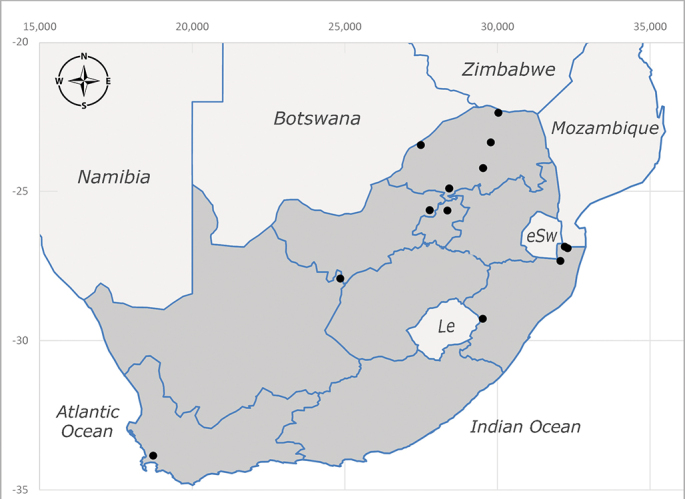
*Rhyncomyapruinosa* occurrence map in South Africa, including eSwatini (eSw) and Lesotho (Le); horizontal axis longitude east and vertical axis latitude south.

**Afrotropical**: Angola, Botswana, Cameroon*, Democratic Republic of Congo, Ethiopia*, Gambia, Kenya, Malawi, Mauritania*, Mozambique, Namibia, Nigeria, Senegal*, South Africa (Fig. 105), Sudan, Zambia and Zimbabwe.

##### Notes

**Figure 106.**
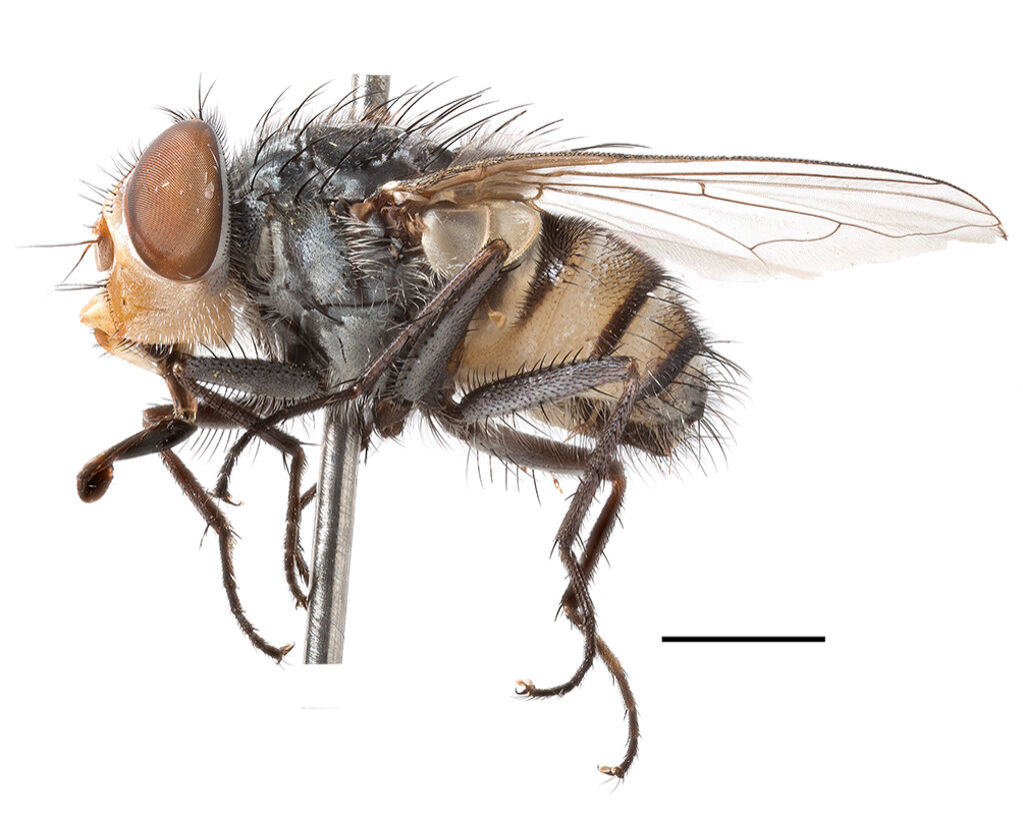
Habitus, left lateral view of *Rhyncomyapruinosa* male BMSA DIP 16807 from South Africa; scale bar = 2 mm.

**Preferred environment**: Dry, sand and broad-leafed deciduous forest and *Acacia* savannah. In Cameroon, in degraded savannah forest. In Namibia, in Miombo and Mopane Woodlands, Arid and Mesic Savannah and Nama-Karoo Biomes (Kurahashi and Kirk-Spriggs 2006). **Recorded elevations**: 53–1086 m a.s.l. **Seasonality**: absent from January to March, May and October, present in low numbers the rest of the months, peaking in December. In Namibia, most abundant in October and December (Kurahashi and Kirk-Spriggs 2006). **Behaviour and ecology**: Cuthbertson (1933) observed in Balla Balla (now Mbalabla), Zimbabwe, that males are scarce and found in flowers and females are very active in late afternoon around cattle kraals, newly-ploughed fields and places in Mopane forest under trees where soil had been dug up by the species in search of larvae. Females were observed depositing eggs into soft soil by thrusting their ovipositors inside and moving at the same time in Zimbabwe (Cuthbertson 1933). Peris (1952b), also in Zimbabwe, recorded females laying eggs in "svil at noots" and grass attacked by termites. **Life cycle and developmental stages**: oviparous. Eggs, larva and pupa known (Engel and Cuthbertson 1937). Engel and Cuthbertson (1937) indicate that: "The eggs are deposited singly in soft soil under the shade of trees, in sandy pathways in savannah forest or in the powdery dung and sand of cattle kraals. The eggs are about 1.75–2 mm. in length, elongate oval, the chorion faintly marked with microscopical reticulations. The female sometimes has a curious habit of filling in the hole made during egg-laying with soil by means of the hind legs, and sometimes by means of the tip of the ovipositor which she uses as a broom. An examination of the egg laying tube has revealed the presence of an armature of spines, which may be similar in function to the spines on the ovipositor of the Asilid. The eggs are usually fully incubated at the time of extrusion, and the newly hatched larvae are very active. They inhabit the top three or four inches of soil, and are often associated there with worker termites, and the pupae of coprophagous beetles and flies. The larvae usually are fully grown when about seven to ten days old. The puparia are found in the soil, usually about four inches beneath the surface. The pupal period is about 7–9 days in the warm weather of the wet season, but is much longer in the cold weather of June and July, about two weeks. The number of eggs laid by this species is not known. Dissections of the female reproductive organs have revealed, in sexually mature individuals, about ten large ovarioles (1.5 mm. in length) in each ovary". Cuthbertson (1933) notes that eggs are covered with a sticky secretion that camouflages them by the attachment of soil particles. Incubation period was around 18 hours. He also observed that larvae are active and live near the soil surface during cool hours of the morning and towards sundown, but retire to the greater depths during the heat of the day. The larvae were found in large numbers in soil under termite-infested dung patches in shade during March and April. The duration of the larval stages varies from 7–10 days. Mature larvae measure 12–15 mm. Their prey is presumably dipterous larvae and pupae or termites which live in their habitat. The pupal stage lasts 7 days in mid-December and 7–10 days in May. Engel and Cuthbertson (1937) illustrated the immature stages. **Collection methods**: sweep net, with light, yellow pan and Malaise traps. In Botswana, Cameroon and Zambia, it was collected with Malaise traps. In Namibia, it was collected with pitfall and Malaise traps and attracted by UV light (Kurahashi and Kirk-Spriggs 2006). **Illustrations and photographs**: male habitus as in Fig. 106. Male terminalia as in fig. 55 in Zumpt (1958).

**Material examined**: Suppl. materials 1, 2.

#### Rhyncomya soyauxi

Karsch, 1886

6595D870-8E1D-5C45-8278-078381CBE5E9

= Rhyncomya soyauxi Karsch, 1886: 262. **Type locality**: Pongo-Andongo [Angola]. **Remarks**: HT in ZMHB.
= Rhyncomya pictifacies Bigot, 1888: 595. **Type locality**: Cape [South Africa].
= Rhynchomyia isaea Séguy, 1933: 69. **Type locality**: Cameroon.
= Rhynchomyia proterva Séguy, 1938: 378. **Type locality**: Kenya, Mt. Elgon.

##### Distribution

**Figure 107.**
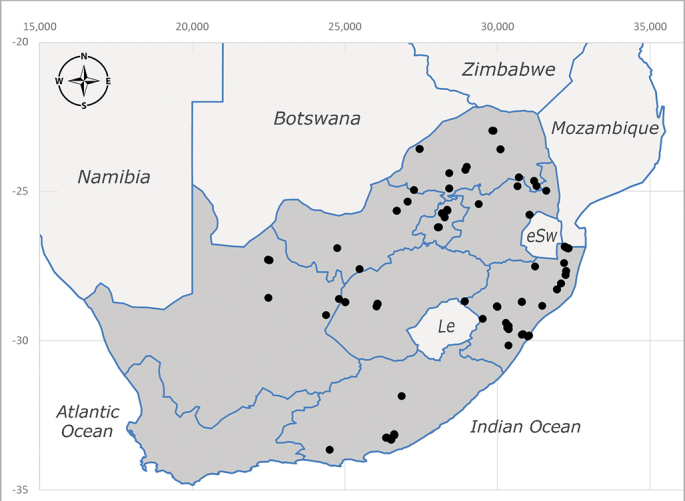
*Rhyncomyasoyauxi* occurrence map in South Africa, including eSwatini (eSw) and Lesotho (Le); horizontal axis longitude east and vertical axis latitude south.

**Afrotropical**: Angola, Botswana, Cameroon, Democratic Republic of Congo, Kenya, Malawi, Namibia, Rwanda*, South Africa (Fig. 107), Sudan, Tanzania, Zambia and Zimbabwe.

##### Notes

**Figure 108.**
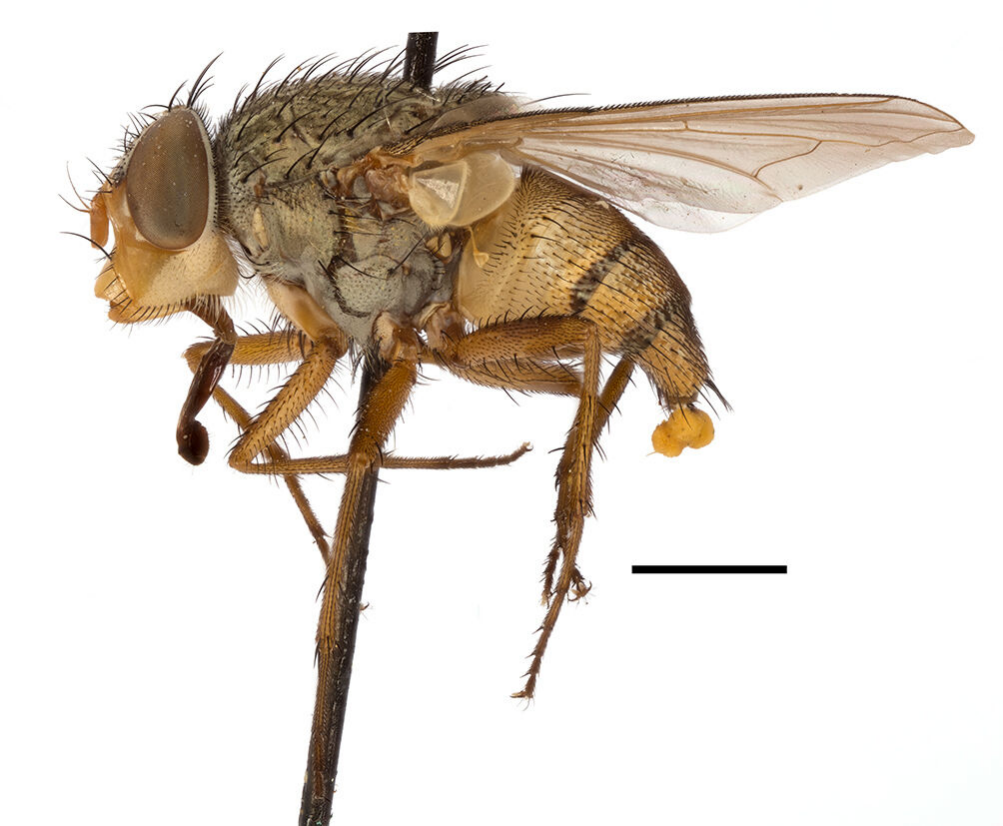
Habitus, left lateral view of *Rhyncomyasoyauxi* female ZMHB HT from Angola; scale bar = 2 mm.

**Preferred environment**: wild environments such as *Acacia* veld (dry mixed bush, savannah and woodland), forests (indigenous Afromontane, broad-leafed deciduous woodland, *Ficus* forest, sand and red sand), grasslands (grassy floodplain, mixed, grass and Kathu), savannah, Kalahari thornveld and rural and urban environments, such as the Albany Museum grounds, camp site areas and sewage-seepage areas. In Kenya, Kenyan dry forest; in Malawi, forest edge, margins and grasslands; in Namibia, the Kwando River floodplain, Miombo and mopane woodlands and open savannah floodplain. Almost all Namibian biomes, except the Hyper-Arid Desert and Succulent Karoo Biomes (Kurahashi and Kirk-Spriggs 2006). **Recorded elevations**: 17–1628 m a.s.l. **Seasonality**: abundant species recorded year-round, with highest abundance in warmer months and lower in colder. Abundant in Namibia, peaking in February and September (Kurahashi and Kirk-Spriggs 2006). **Behaviour and ecology**: flower-frequenting, both sexes feed on flowers, especially Asteraceae (as Compositae) in the savannah forest of Zimbabwe (as *R.pictifacies*) (Cuthbertson 1933). Peris (1952b) also recorded the species on flowers of wild Compositae and Cape Gooseberry in Zambia. In South Africa, some specimens were recorded to be associated with *Acacia* sp., *Boscia* sp., *Acacia*-*Rhigozum* Burch. scrub and *Searsia* sp. F.A.Barkley (as *Rhus*). Females were observed ovipositing in rich soil at the edge of fresh cow-dung, Eastern Victoria, Zimbabwe. Additionally, *R.soyauxi* was caught together with *Bembixalbofasciata* and *Bembixmelanopa* as their prey. **Life cycle and developmental stages**: unknown, but Cuthbertson (1933) indicated that their eggs are similar to *R.pruinosa*, cream-coloured, sausage-shaped and about 1.75 mm long. **Collection methods**: Malaise and light traps, MV and black light trap and sweeping. In Botswana, Malaise traps; in Kenya, general sweeping, Malaise and migration traps. In Namibia, with yellow, blue and white pans, pitfall and Malaise traps, hand net, sweeping, UV-light and McPhail traps baited with Nu-Lure (Kurahashi and Kirk-Spriggs 2006). **Illustrations and photographs**: female habitus as in Fig. 108. Male terminalia as in fig. 53 in Zumpt (1958).

**Type material examined**: *R.soyauxi*: 1 ? // Typus // Pungo-Andongo / Leg. V. Homeyer // 11013 // *Rhynch*. / ?*Soyeauxi* / K.* // *Rhyncomyia* ? / *soyauxi* Karsch / C = *pictifacies* Bigot) // [ZMHB].

**Material examined**: Suppl. materials 1, 2.

#### Rhyncomya stannocuprea

Speiser, 1910

60467187-EE05-547B-A0F3-C2C89B9905BD

= Rhyncomya stannocuprea Speiser, 1910: 150. **Type locality**: Tanganyika [Tanzania], Meru Kilimandjaro.
= Rhyncomyia stannocuprea spp. *abyssinica* Peris, 1951: 244. **Type locality**: Abyssinia [Ethiopia], Gatelo Amaizu.

##### Distribution

**Figure 109.**
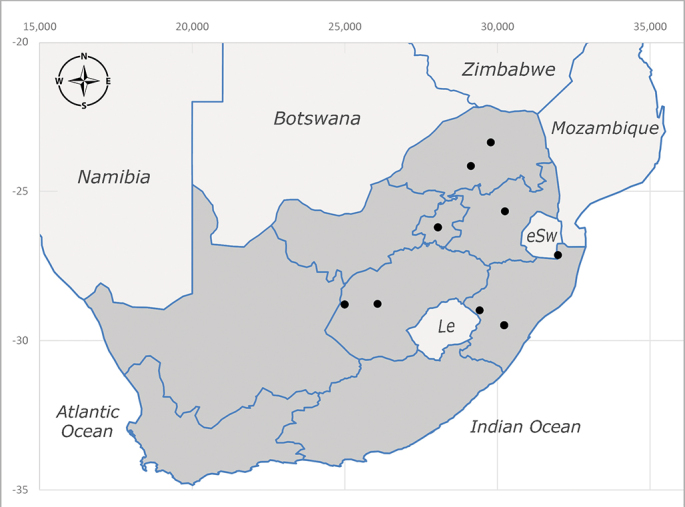
*Rhyncomyastannocuprea* occurrence map in South Africa, including eSwatini (eSw) and Lesotho (Le); horizontal axis longitude east and vertical axis latitude south.

**Afrotropical**: Kenya, Ethiopia, Malawi*, Namibia*, South Africa (Fig. 109), Tanzania and Zimbabwe.

##### Notes

**Figure 110.**
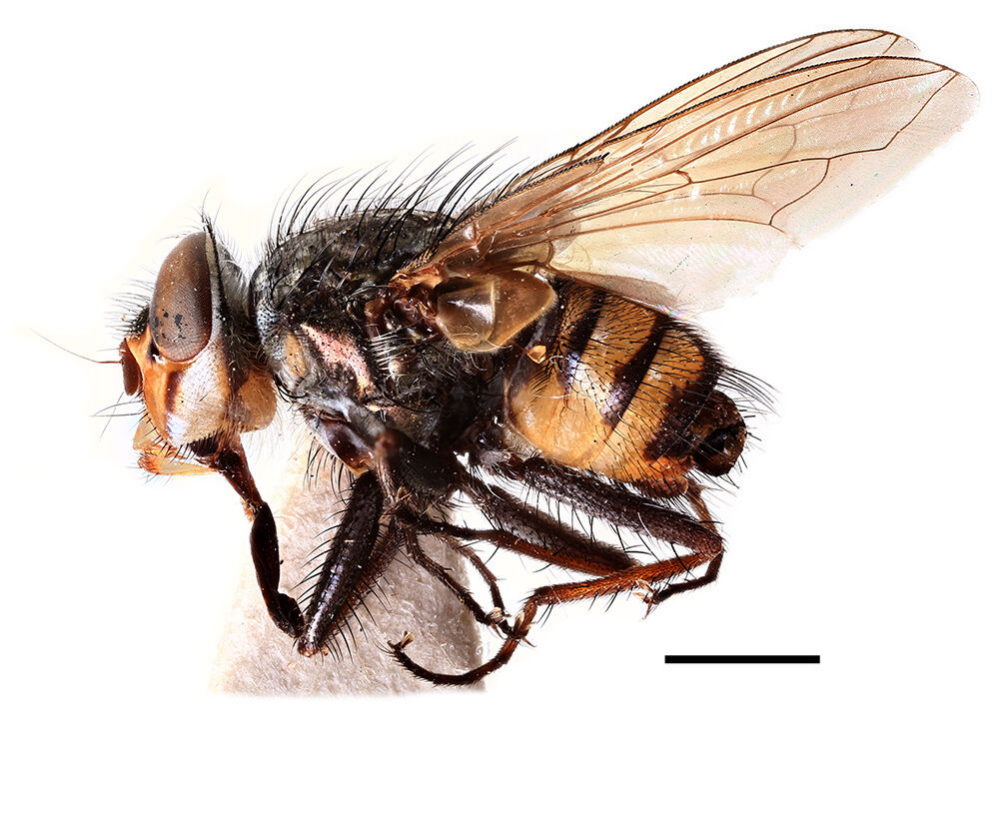
Habitus, left lateral view of *Rhyncomyastannocuprea* male NMSA DIP 019535 from South Africa; scale bar = 2 mm.

**Preferred environment**: *Acacia* savannah, rocky hills with *Acacia* veld and grassland and forest edges. In Malawi, forest edges and grasslands; in Namibia the Kwando River floodplain. **Recorded elevations**: 1200–1500 m a.s.l. **Seasonality**: low numbers year-round, peaking in September, absent in March and May to July. **Behaviour and ecology**: unknown. **Life cycle and developmental stages**: unknown. **Collection methods**: Malaise trap. In Nambia, Malaise trap. **Illustrations and photographs**: male habitus as in Fig. 110. Male terminalia as in fig. 54 in Zumpt (1958).

**Material examined**: Suppl. materials 1, 2.

#### Rhyncomya trispina

Villeneuve, 1929

EA0B41AD-E660-59E7-BE65-46B7DB1B1C89

= Rhyncomya trispina Villeneuve, 1929: 62. **Type locality**: Southern Rhodesia [Zimbabwe], Bulawayo. **Remarks**: HT in NHMUK.

##### Distribution

**Figure 111.**
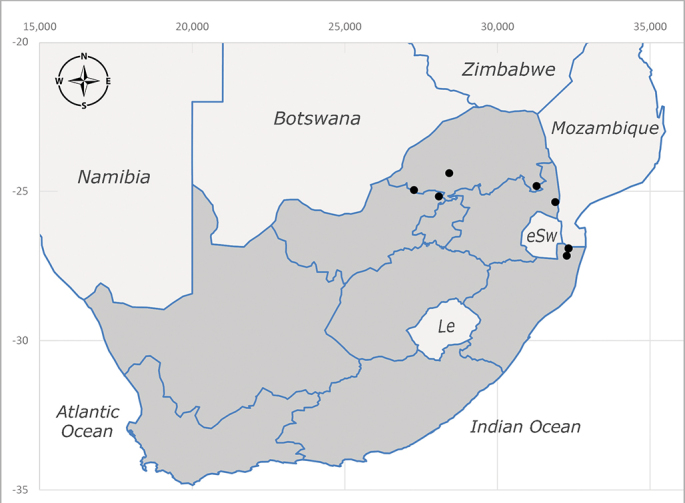
*Rhyncomyatrispina* occurrence map in South Africa, including eSwatini (eSw) and Lesotho (Le); horizontal axis longitude east and vertical axis latitude south.

**Afrotropical**: Botswana, Democratic Republic of Congo*, Kenya, ?Mali, Mozambique, Namibia, South Africa (Fig. 111) and Zimbabwe.

##### Notes

**Figure 112.**
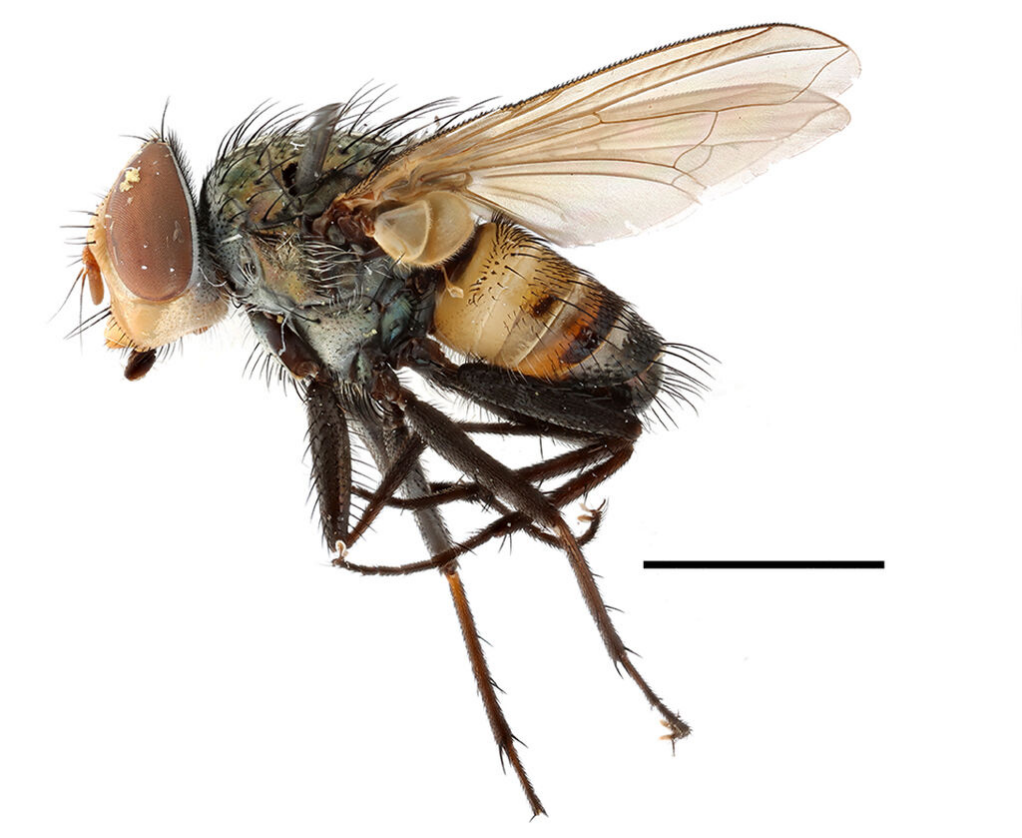
Habitus, left lateral view of *Rhyncomyatrispina* male BMSA DIP 33393 from Namibia; scale bar = 2 mm.

**Preferred environment**: broad-leafed deciduous woodland and dry scrub forest and to *Acacia* thornveld, Kwando river floodplain and the Miombo and mopane woodlands. In Namibia, degraded sand forest and cultivated plots. Kurahashi and Kirk-Spriggs (2006) reported it as restricted to the Arid and Mesic Savannah Biomes. **Recorded elevations**: 98–1095 m a.s.l. **Seasonality**: only present in April, May, July and peaking in November. In Namibia, most abundant in December and April (Kurahashi and Kirk-Spriggs 2006). **Behaviour and ecology**: in Namibia, collected feeding on fresh African elephant (*Loxodontaafricana* (Blumenbach)) dung (Kurahashi and Kirk-Spriggs 2006). **Life cycle and developmental stages**: unknown. **Collection methods**: Malaise traps. In Zimbabwe, Malaise traps. In Namibia, yellow and blue pans, pitfall and Malaise traps, UV-light, hanging traps baited with fermented fruit and also sweeping (Kurahashi and Kirk-Spriggs 2006). **Illustrations and photographs**: male habitus as in Fig. 112. Male terminalia as in fig. 59 in Zumpt (1958).

**Type material examined**: *R.trispina*: 1 ? // Holo- / type // Bulawayo / S. Rhodesia / 25.ix.1923 / Coll. R. Stevenson // Pres. by / Com. Inst. Ent. / B.M. 1955-504 // *Rhyncomyia* / *trispina* / type Villen. // [NHMUK 010832199].

**Material examined**: Suppl. materials 1, 2.

#### Rhyncomya tristis

Séguy, 1933

2A62478F-19B2-559F-989D-79F3FACA1302

= Rhynchomyia tristis Séguy, 1933: 67. **Type locality**: Mozambique, Zambèze [Zambezi], N. Chupanga [Nova-Chupanga]. **Remarks**: HT and PT in MNHN.

##### Distribution

**Figure 113.**
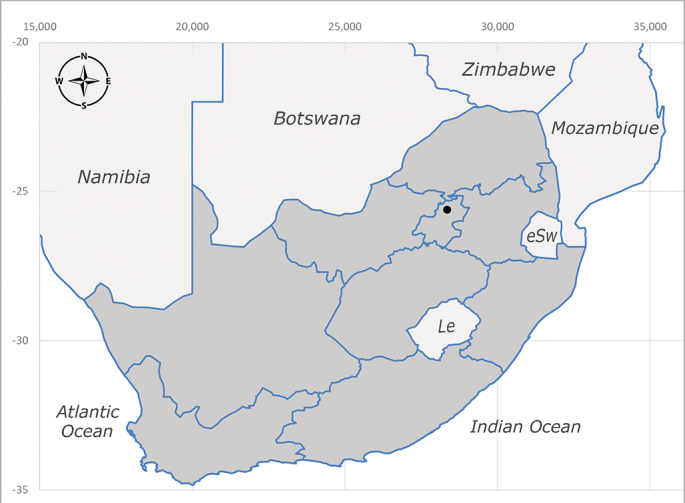
*Rhyncomyatristis* occurrence map in South Africa, including eSwatini (eSw) and Lesotho (Le); horizontal axis longitude east and vertical axis latitude south.

**Afrotropical**: Botswana, Chad, Namibia, Nigeria, Mozambique, South Africa* (Fig. 113), Tanzania*, Yemen, Zambia* and Zimbabwe. **Palaearctic**: Saudi Arabia.

##### Notes

**Figure 114.**
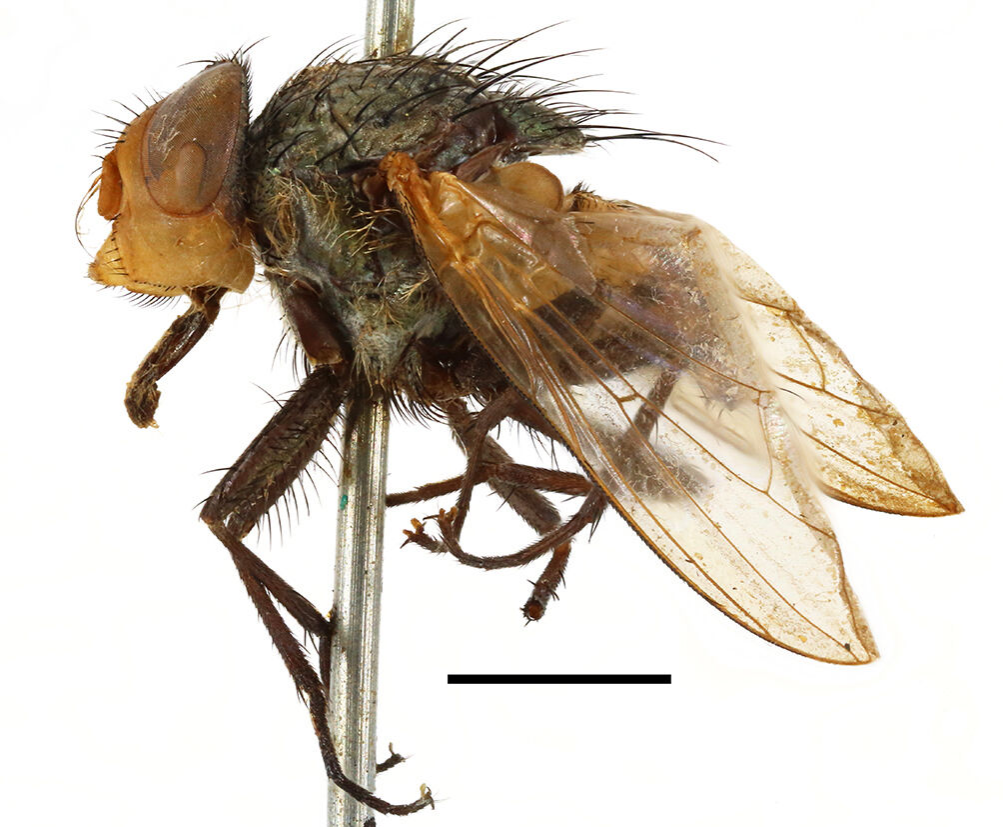
Habitus, left lateral view of *Rhyncomyatristis* male MNHN HT from Mozambique; scale bar = 2 mm.

**Preferred environment**: in Tanzania, riverine in dry forest. In Namibia, the Arid Savannah, Mesic Savannah and Nama-Karoo Biomes (Kurahashi and Kirk-Spriggs 2006). **Recorded elevations**: 1200 m a.s.l. **Seasonality**: one specimen in February. Recorded year-round in Namibia, except in August, being most abundant in March (Kurahashi and Kirk-Spriggs 2006). **Behaviour and ecology**: unknown. **Life cycle and developmental stages**: unknown. **Collection methods**: Malaise traps. In Mozambique and Tanzania, Malaise traps. In Namibia, collected by hand, in yellow pans, pitfall and Malaise traps and at UV-light (Kurahashi and Kirk-Spriggs 2006). **Illustrations and photographs**: male habitus as in Fig. 114. Male terminalia unknown.

**Type material examined**: *R.tristis*: 1 ? // TYPE // MUSEUM PARIS / Zambèze / N. Chupanga / J. SURCOUF 1926 // Octobre 27 // *Rhyncomyia* / *tristis* Séguy / TYPE / E. Séguy det. 1925 // slice no. 16 // Prépar. microsc. / nº 3344. 1 ? // PARATYPE // MUSEUM PARIS / Zambèze / N. Chupanga / J. SURCOUF 1926 // Janvier 28 // *Rhyncomyia* ? / *tristis* Séguy / vide Zumpt 75.

**Material examined**: Suppl. materials 1, 2.

#### Rhyncomya viduella

Villeneuve, 1927

EFE03BD1-5991-5235-B8FA-D8345E33BF81

= Rhyncomya viduella Villeneuve, 1927: 18 (see taxonomic notes). **Type locality**: South Africa, Transvaal [Mpumalanga], Barberton. **Remarks**: HT in SAMC; nec *Rhynchomyacassotis* (Walker, 1849) sensu *Zumpt (1958)* (see discussion).

##### Distribution

**Figure 115.**
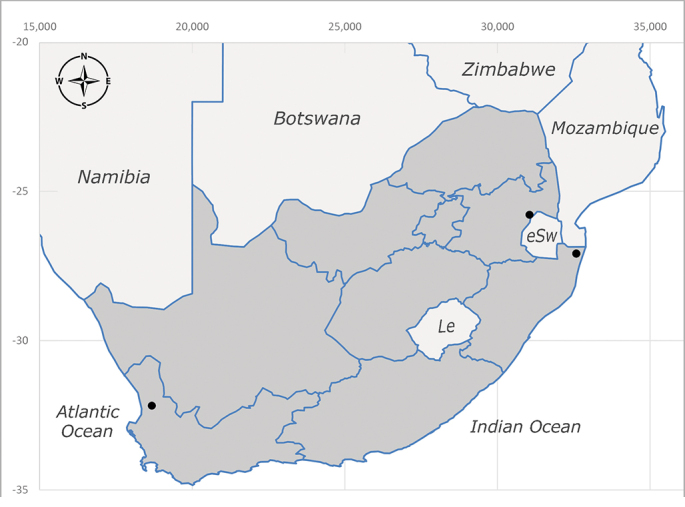
*Rhyncomyaviduella* occurrence map in South Africa, including eSwatini (eSw) and Lesotho (Le); horizontal axis longitude east and vertical axis latitude south.

**Afrotropical**: South Africa (Fig. 115).

##### Notes

**Figure 116.**
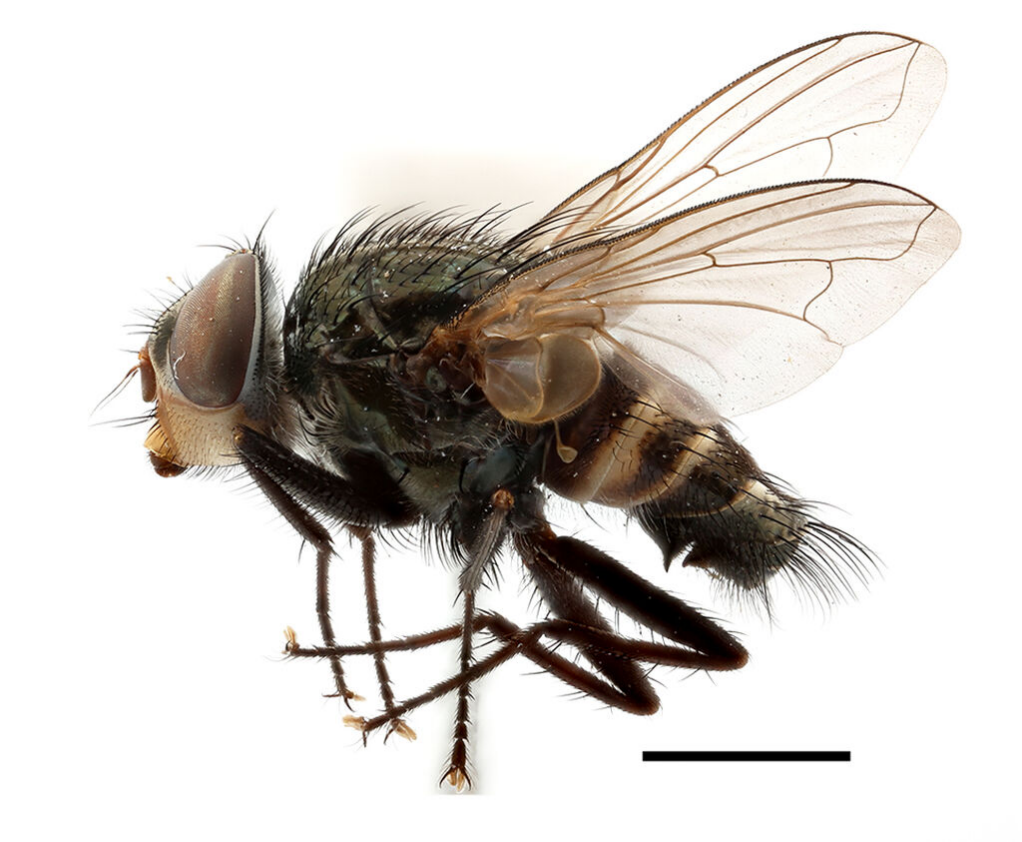
Habitus, left lateral view of *Rhyncomyaviduella* male SANC from South Africa; scale bar = 2 mm.

**Preferred environment**: hillside with flowers. **Recorded elevations**: 495 m a.s.l. **Seasonality**: low numbers in September, October and December. **Behaviour and ecology**: unknown. **Life cycle and developmental stages**: unknown. **Collection methods**: sweeping from *Asparagus* sp. (Asparagaceae). **Illustrations and photographs**: male habitus as in Fig. 116. Male terminalia unknown.

**Taxonomic notes**: *Rhyncomyaviduella* **stat**. **rev**. is reinstated as a valid species. Previously listed as a synonym of *R.cassotis* (Fig. 37) by *Zumpt (1958)*, *R.viduella* **stat**. **rev**. is characterised by a dark fronto-orbital plate and parafacial, a parafacial and genal-dilatation covered by black setulae, an abdomen predominately dark with small testaceous fringe in the posterior tergites border and a body length of 7-9 mm. In contrast, the typical morphotype of *R.cassotis* is represented by having a yellow-golden fronto-orbital plate and parafacial, a bare parafacial, genal-dilatation covered with pale setulae, a predominately yellow abdomen with variable dark patterns on tergites 4 to 5 and a body length of 4-7 mm. Thus, the morphologies of *R.viduella* **stat**. **rev**. and *R.cassotis* indicate that they are distinct species, as Peris (1952a) indicated in his monographs. Despite these morphological differences, *Zumpt (1958)* considered that *R.viduella* **stat**. **rev**. was probably a dark morphotype of *R.cassotis* because he did not find differences between the male terminalia of the dark and light morphotypes and remarked that the taxonomic status of the forms is not quite clear, an opinion he also held regarding the readily distinguishable species *Chrysomyachloropyga* (Wiedemann, 1818) and *Chrysomyaputoria* (Wiedemann, 1830) (e.g. Zumpt, 1965; cf. Rognes and Paterson 2005). After dissecting and examining the HT of *R.viduella* **stat**. **rev**. at SAMC and comparing it with typical *R.cassotis* specimens, we found that their male terminalia are clearly different, confirming that they are different species (Thomas-Cabianca et al., unpublished).

**Type material examined**: *R.viduella*: 1 ? // Barberton / Transvaal /H Edwards // Dec 1911 // *Rhyncomyia* / *viduella* / ? Type // [SAMC DIP A011183].

**Material examined**: Suppl. material 1.

#### Stegosoma

Loew, 1863

9A0DE24C-5BB1-5ADB-A752-322C8C5B7FF6

= Stegosoma Loew, 1863: 15. **Type species**: *Stegosomavinculatum* Loew, 1863, by monotypy.

#### Stegosoma bowdeni

Peris, 1951

A6368ED3-51BD-57EA-932C-7B04657FBEAE

= Stegosoma bowdeni Peris, 1951: 239. **Type locality**: Golden Coast [Ghana], Mampong. **Remarks**: HT and PT in NHMUK and PTs in NMSA.

##### Distribution

**Figure 117.**
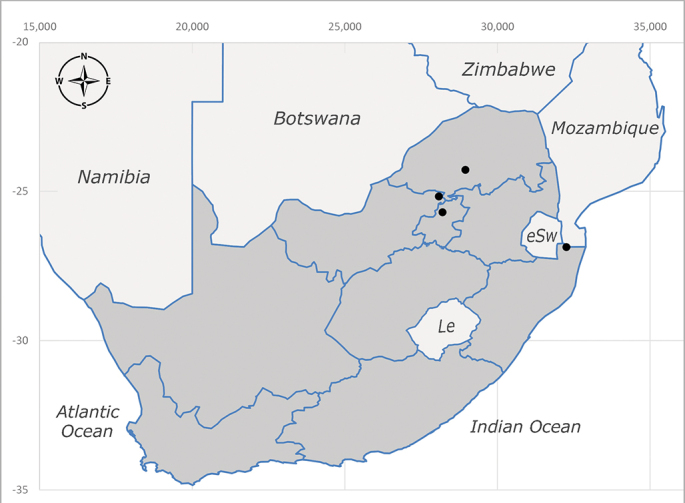
*Stegosomabowdeni* occurrence map in South Africa, including eSwatini (eSw) and Lesotho (Le); horizontal axis longitude east and vertical axis latitude south.

**Afrotropical**: Cameroon, Democratic Republic of Congo, Ghana, Kenya*, Nigeria, South Africa (Fig. 117), Tanzania and Togo*.

##### Notes

**Figure 118.**
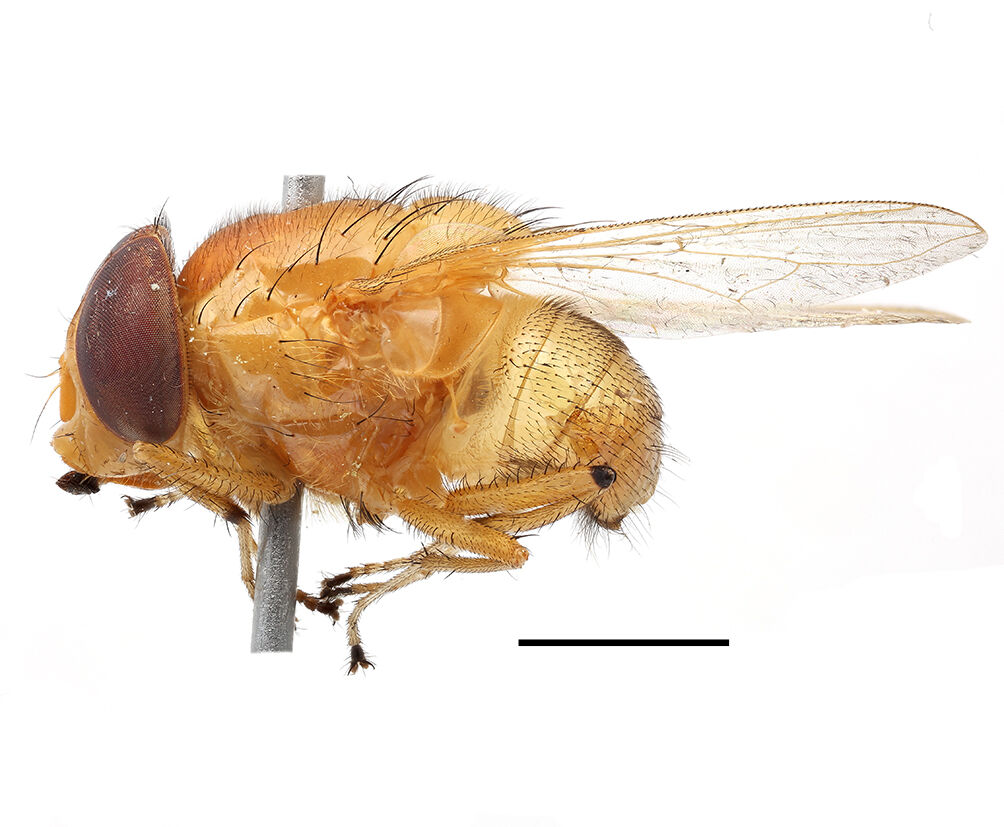
Habitus, left lateral view of *Stegosomabowdeni* male BMSA DIP 84384 from Togo; scale bar = 2 mm.

**Preferred environment**: in Togo, vegetated stream-bed and lowland evergreen secondary forest. **Recorded elevations**: no data. **Seasonality**: recorded in February, October and December. **Behaviour and ecology**: Peris (1952a) reported *S.bowdeni* with *Nasutitermes* Banks in Nigeria and that it was attracted to open termite nests in South Africa. In Ghana, females were observed ovipositing in an open mount of *Macrotermes* Holmgren. **Life cycle and developmental stages**: unknown. **Collection methods**: in Togo, with Malaise traps. **Illustrations and photographs**: male habitus as in Fig. 118. Male terminalia unknown.

**Type material examined**: *S.bowdeni*: 1 ? // Holotype // GOLD COAST / Mampong / (Ashanti) / 12.iv.1947 / J. Bowden // *Stegosoma* / *bowdeni* / n.sp. / S-V. Peris 1948 // [NHMUK]. 5 ?? // GOLD COAST / Mampong / (Ashanti) / 12.iv.1947 / J. Bowden / 451/47 // Ovipositing in / opened mount /of macrotermes // NB Par-of / onqunalantes: / presumably rank as / Paratypes J.B. // *Stegosoma* / *bowdeni* / n.sp. / S-V. Peris 1948 // [NMSA DIP 019516].

**Material examined**: Suppl. materials 1, 2.

#### Stegosoma vinculatum

Loew, 1863

88FE79F2-261F-5367-A5E8-DC1FC884F553

= Stegosoma vinculatum Loew, 1863: 15. **Type locality**: South Africa, Orange Free State [Free State], Bloemfontein.

##### Distribution

**Figure 119.**
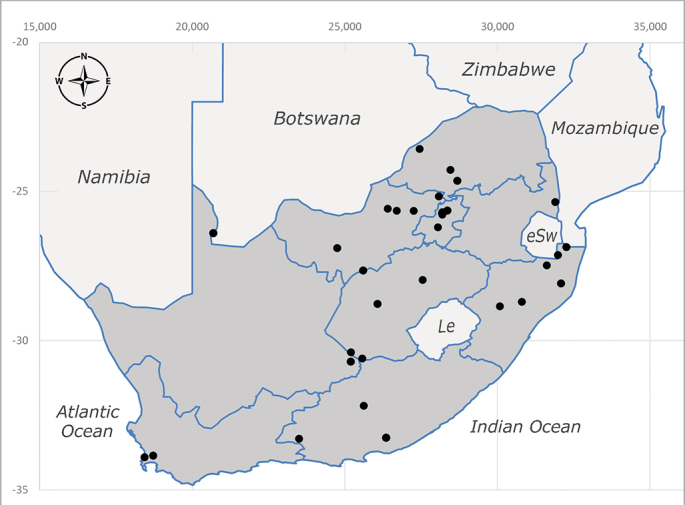
*Stegosomavinculatum* occurrence map in South Africa, including eSwatini (eSw) and Lesotho (Le); horizontal axis longitude east and vertical axis latitude south.

**Afrotropical**: Benin, Botswana, Democratic Republic of Congo, Ghana, Kenya, Malawi, Mali, Mozambique, Namibia, Nigeria, South Africa (Fig. 119), Zambia and Zimbabwe.

##### Notes

**Figure 120.**
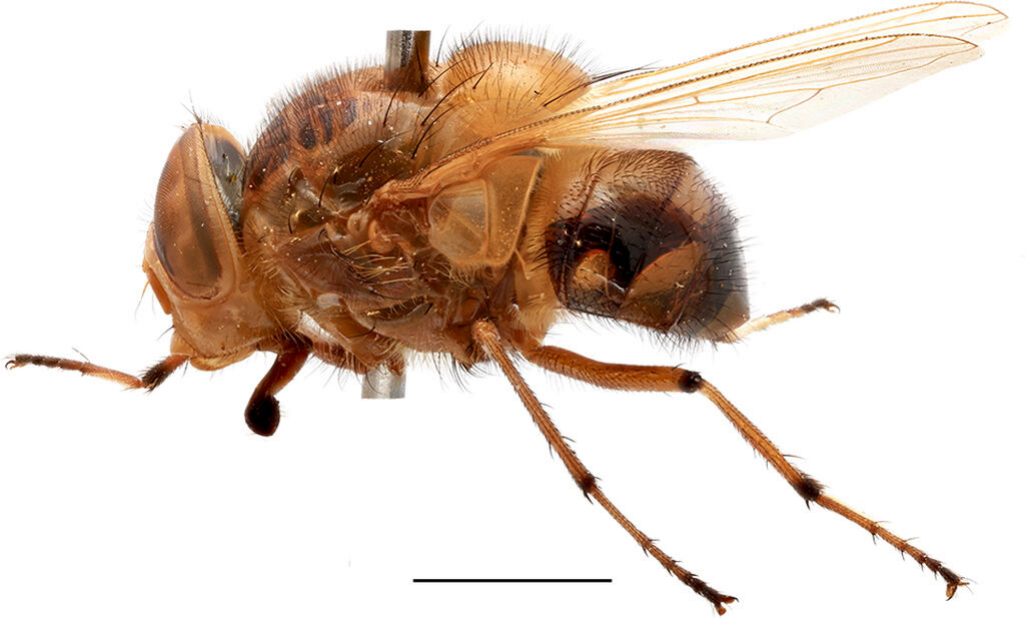
Habitus, left lateral view of *Stegosomavinculatum* male AMGS 100997 from South Africa; scale bar = 2 mm.

**Preferred environment**: *Acacia* savannah and mixed bushveld-grass. In Namibia, degraded sand forest and cultivated plots; apparently restricted to the Arid and Mesic Savannah Biomes (Kurahashi and Kirk-Spriggs 2006). **Recorded elevations**: 1000–1240 m a.s.l. **Seasonality**: present year-round except for coldest months, most abundant in March and December. In Namibia, most abundant in February and December (Kurahashi and Kirk-Spriggs 2006). **Behaviour and ecology**: females and males were observed on flowering *Gymnosporialinearis*, on flowers of *Gymnosporiaheterophylla* and on yellow flowers of *Deverraaphylla* (Cham. and Schlechtd.) DC. and *Heteromorphatrifoliate* (Wendl.) Eckl. and Zeyh. In Namibia, it was collected and observed on yellow flowers of *Zygophyllumsimples* I. Cuthbertson (1933) observed that males are uncommon in Harare and Victoria District in Zimbabwe (as Salisbury) and occur in flowers of *Gymnosporia* sp. (as *Gymosporia* [*sic*]). Attracted to open termite nest, ex termite nest of *Trinervitermes* (Isoptera), pinned with termite. Additionally in South Africa, *Zumpt (1958)* recorded that one male was reared from the nest of *Trinervitermeshavilandi* Fuller (= *T.trinervoides* (Sjöstedt, 1911)) in Johannesburg. In Namibia, females were exclusively attracted to broken termite nests (*Trinervitermes*: *T.* ?*T.rapulum* (Sjöstedt), *T.* ?*T.rhodesiensis* (Sjöstedt) and *Trinervitermes* sp. (Kurahashi and Kirk-Spriggs 2006). In Gobabis District, Namibia, males were observed swarming before dawn, approximately one metre above the ground and around a *Terminalia sericea* Burch tree. In Bulawayo, Zimbabwe, Cuthbertson (1933) observed adults swarming from a termite nest. Females were found in the vicinity of termite nests that had been cut through by a plough. Additionally seen in aardvark (*Oryctoropusafer* (Pallas, 1766)) burrows. Peris (1952a) reported the species in an ant hill in Ghana and in a pig hole (probably a warthog (*Phacochoerusafricanus* (Gmelin)) burrow) in Nigeria. Females were observed laying eggs in soil and detritus at the bottom of the burrows in termite nests made by aardvarks (Cuthbertson 1933). In Namibia, it was reported to have nocturnal and semi-nocturnal habits (Kurahashi and Kirk-Spriggs 2006). **Life cycle and developmental stages**: oviparous. Eggs, larvae and pupae described (Cuthbertson 1933, Cuthbertson 1935). Cuthbertson (1933) also reported that "*Larvae were very active, occurring among dead and dying worker termites, it suggesting larvae could be predators. Eggs are long and slender (2.25 mm.) and were fully incubated at the moment of deposition in the soil. Newly hatched larvae measure 2.5 mm, and are able to burrow quickly downwards into the soil and termite-debris, where they develop to the pupal stage in 4–5 days. The live cycle in Mbalabala was between 14–17 days in April, 1933*". In laboratory conditions, larvae were reared by supplying worker termites daily, but Cuthbertson (1933) also noted that the precise nature of their food is unknown. The eggs, 1st and 3rd instar larvae and puparium were described and illustrated by Cuthbertson (1935). **Collection methods**: Malaise trap. In Namibia, with Malaise and pitfall traps and sweeping (Kurahashi and Kirk-Spriggs 2006). **Illustrations and photographs**: male habitus as in Fig. 120. Male terminalia as in fig. 66 in Zumpt (1958).

**Material examined**: Suppl. materials 1, 2.

#### Stegosoma wellmani

(Lichtwardt, 1908)

4BB92558-38CC-5022-BA36-1E3A8A35D8E9

= Rhynchomyia wellmani Lichtwardt, 1908: 338. **Type locality**: Angola, Benguella.

##### Distribution

**Figure 121.**
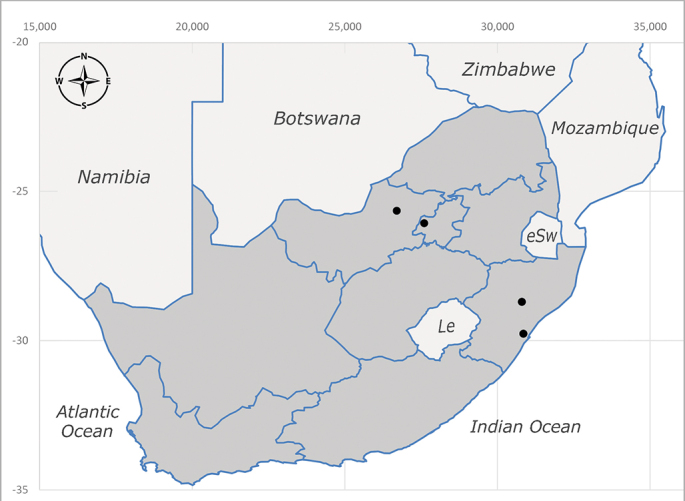
*Stegosomawellmani* occurrence map in South Africa, including eSwatini (eSw) and Lesotho (Le); horizontal axis longitude east and vertical axis latitude south.

**Afrotropical**: Angola, Cameroon, Central African Republic*, Democratic Republic of Congo, Equatorial Guinea, Ghana, Kenya, Liberia, Nigeria, Sierra Leone, South Africa (Fig. 121), Sudan, Tanzania, Uganda and Zimbabwe.

##### Notes

**Figure 122.**
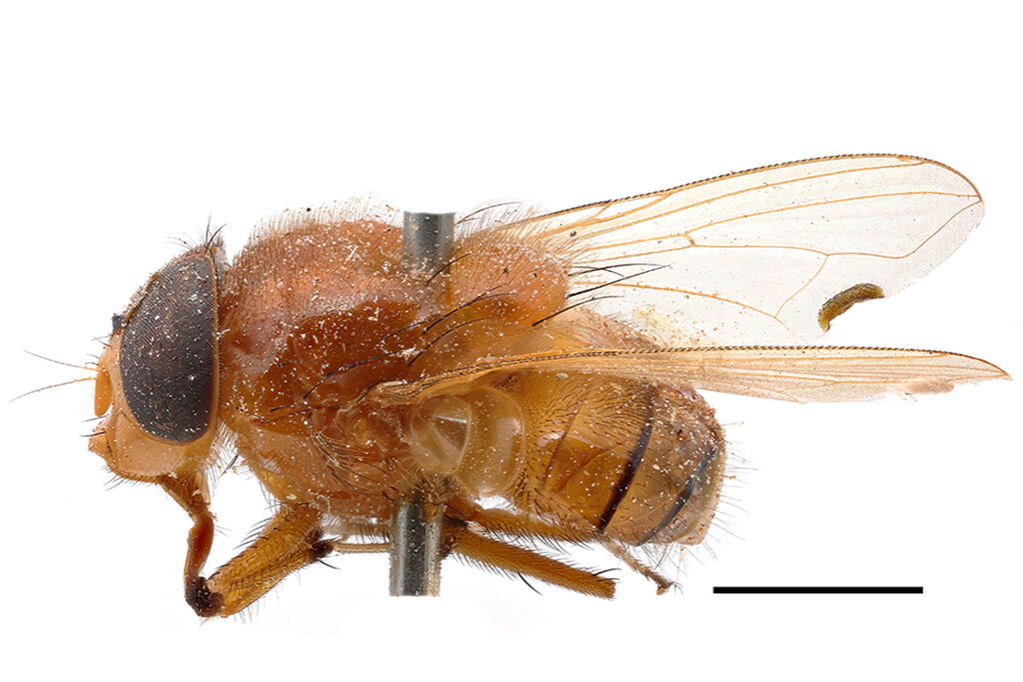
Habitus, left lateral view of *Stegosomawellmani* male BMSA DIP (BECE) 02553 from Kenya; scale bar = 2 mm.

**Preferred environment**: in Democratic Republic of Congo, in lowland evergreen swamp forest. **Recorded elevations**: no data. **Seasonality**: recorded in January, February and April, absent the rest of the year. **Behaviour and ecology**: collected at *Microtermes* Wasmann nest in Kenya and ovipositing in opened mound of *Macrotermes* Holmgren in Ghana. **Life cycle and developmental stages**: unknown. **Collection methods**: in Democratic Republic of Congo, with Malaise trap. **Illustrations and photographs**: male habitus as in Fig. 122. Male terminalia unknown.

**Material examined**: Suppl. materials 1, 2.

#### Thoracites

Brauer and Bergenstamm, 1891

8E2EA61C-8736-560D-88DA-AAEDCAE43673

= Thoracites Brauer and Bergenstamm, 1891: 363. **Type species**: *Muscaabdominalis* Fabricius, 1805, by original designation.

#### Thoracites cingulatus

Bezzi, 1914

2223DDEA-630D-5B94-9284-BB74F6EB707E

= Thoracites cingulatus Bezzi, 1914: 290. **Type locality**: Senegal, Theis.

##### Distribution

**Figure 123.**
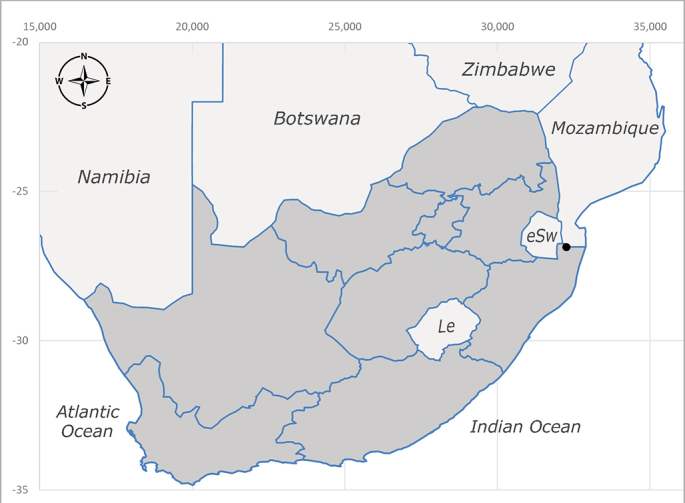
*Thoracitescingulatus* occurrence map in South Africa, including eSwatini (eSw) and Lesotho (Le); horizontal axis longitude east and vertical axis latitude south.

**Afrotropical**: Botswana, Mozambique, Nigeria, Senegal and South Africa (Fig. 123).

##### Notes

**Preferred environment**: no data. **Recorded elevations**: no data. **Seasonality**: three specimens recorded in December. **Behaviour and ecology**: unknown. **Life cycle and developmental stages**: unknown. **Collection methods**: unknown. **Illustrations and photographs**: female habitus as in fig. 29a in Kurahashi (2001). Male terminalia as in fig. 19 in Zumpt (1958) and fig. 4 in Zumpt (1972b).

**Material examined**: Suppl. material 1.

#### Thoracites kirkspriggsi

Kurahashi, 2001

2313CE29-75A3-5345-9160-D9F55D28A3F1

= Thoracites kirkspriggsi Kurahashi, 2001: 146. **Type locality**: Namibia, Rundo District.

##### Distribution

**Figure 124.**
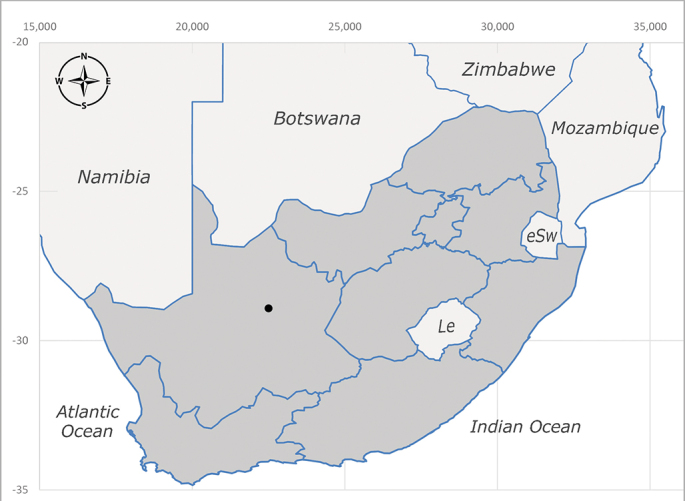
*Thoraciteskirkspriggsi* occurrence map in South Africa, including eSwatini (eSw) and Lesotho (Le); horizontal axis longitude east and vertical axis latitude south.

**Afrotropical**: Namibia and South Africa* (Fig. 124).

##### Notes

**Preferred environment**: in Namibia, apparently restricted to the Arid and Mesic Savannah Biomes (Kurahashi 2001; Kurahashi and Kirk-Spriggs 2006). **Recorded elevations**: no data. **Seasonality**: a single specimen was collected in September. In Namibia, abundant species, most abundant from December to March, absent in June (Kurahashi and Kirk-Spriggs 2006). **Behaviour and ecology**: unknown. **Life cycle and developmental stages**: unknown. **Collection methods**: preservative trap. In Namibia, swept and hand net, UV-Light, Malaise, preservative, pitfall, baited with dead millipede and yellow, blue and white pan traps (Kurahashi 2001, Kurahashi and Kirk-Spriggs 2006). **Illustrations and photographs**: male habitus as in fig. 30a in Kurahashi (2001). Male terminalia as in figs. 5-10 in Kurahashi (2001).

**Material examined**: Suppl. materials 1, 2.

#### Thoracites petersiana

(Loew, 1852)

E84C3C05-DE7C-5FD8-BABE-EF7F35A59C84

= Ochromyia petersiana Loew, 1852: 660. **Type locality**: South Africa, Zululand [KwaZulu-Natal], Mtubatuba.
= Thoracites neglectus Zumpt, 1972: 49. **Type locality**: South Africa, Zululand [KwaZulu-Natal], Mtubatuba. **Remarks**: HT in NMSA.

##### Distribution

**Figure 125.**
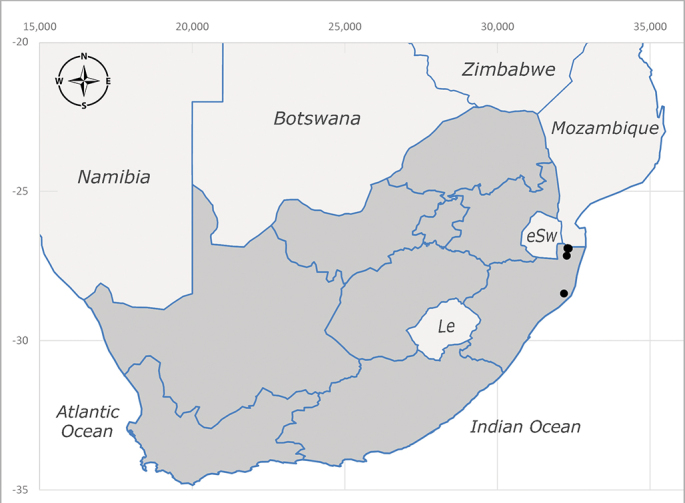
*Thoracitespetersiana* occurrence map in South Africa, including eSwatini (eSw) and Lesotho (Le); horizontal axis longitude east and vertical axis latitude south.

**Afrotropical**: South Africa (Fig. 125).

##### Notes

**Figure 126.**
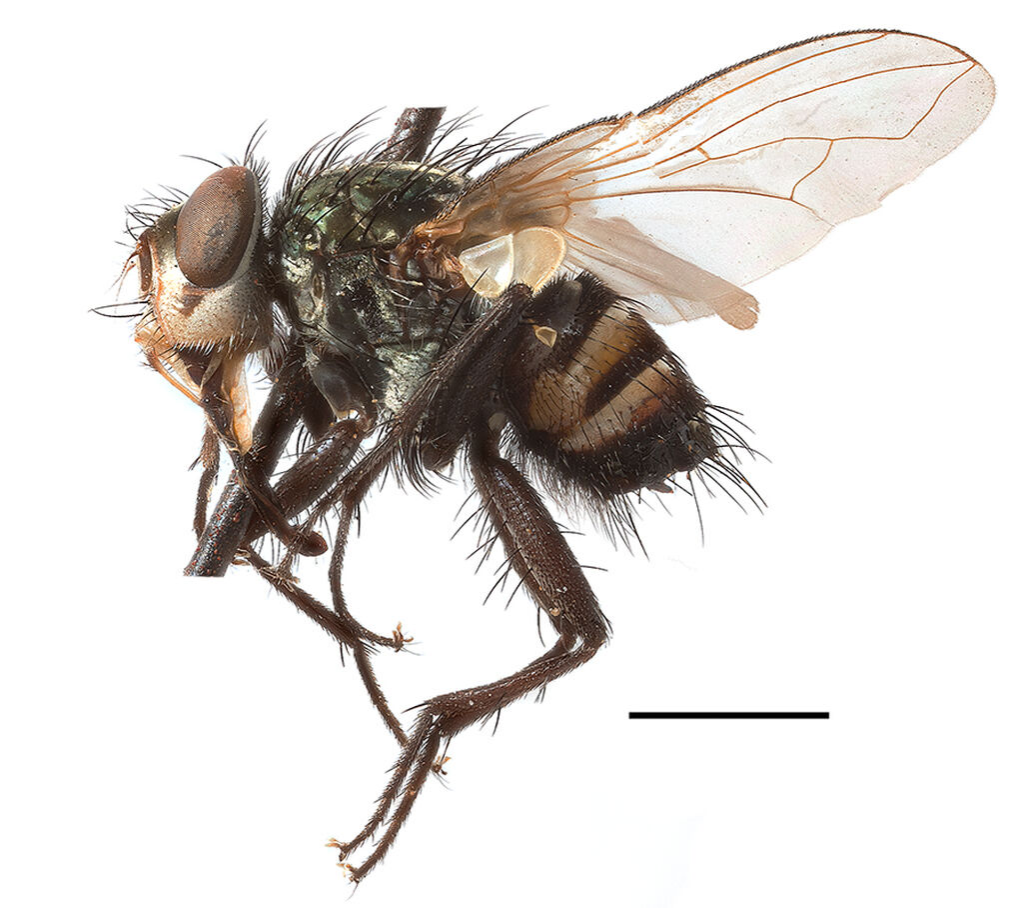
Habitus, left lateral view of *Thoracitespetersiana* male NMSA DIP 19669 HT of *T.neglectus* from South Africa (without terminalia); scale bar = 2 mm.

**Preferred environment**: broad-leafed deciduous woodland forest, grassy floodplain and sand and forest. **Recorded elevations**: 77–98 m a.s.l. **Seasonality**: abundant species present in May, November and December (peaking in December). **Behaviour and ecology**: unknown. **Life cycle and developmental stages**: unknown. **Collection methods**: Malaise trap. **Illustrations and photographs**: male habitus as in Fig. 126 and fig. 29d in Kurahashi (2001). Male terminalia as in fig. 2 in Zumpt (1972b).

**Type material examined**: *T.neglectus*: 1 ? // Holotypus // Mtubatuba / Zululand / May 1941 / H.K. Munro // slide no. 95 // *Thoracites* / *neglectus* n. sp. / Zumpt 1972 // [NMSA-DIP 019669].

**Material examined**: Suppl. material 1.

#### Thoracites sarcophagoides

Kurahashi, 2001

E55AD062-20BB-5E68-A6F6-719E3A2D3BD6

= Thoracites sarcophagoides Kurahashi, 2001: 155. **Type locality**: Namibia, West Caprivi PK, Kwando River.

##### Distribution

**Figure 127.**
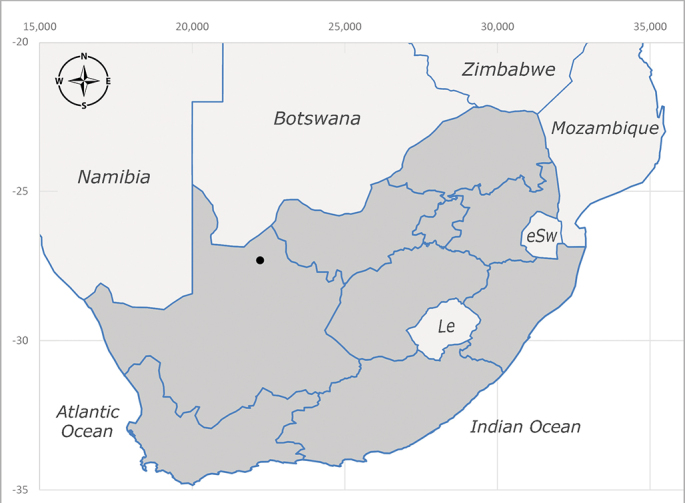
*Thoracitessarcophagoides* occurrence map in South Africa, including eSwatini (eSw) and Lesotho (Le); horizontal axis longitude east and vertical axis latitude south.

**Afrotropical**: Namibia and South Africa* (Fig. 127).

##### Notes

**Preferred environment**: Gordonia Plains Schrubland, *Senegaliamellifera* (as *Acacia)* on red sand in the Savannah Biome. **Recorded elevations**: 1035 m a.s.l. **Seasonality**: only one specimen between September and November. In Namibia, recorded year-round, most abundant from December to February and September (Kurahashi 2001, Kurahashi and Kirk-Spriggs 2006). **Behaviour and ecology**: unknown. **Life cycle and developmental stages**: unknown. **Collection methods**: Malaise traps. In Namibia, by hand net, pitfall, pan (yellow, white, blue, brown and orange) and Malaise traps and in fresh African elephant dung (Kurahashi 2001, Kurahashi and Kirk-Spriggs 2006). **Illustrations and photography**: male habitus as in fig. 30b in Kurahashi (2001). Male terminalia as in figs. 23–28 in Kurahashi (2001).

**Material examined**: Suppl. material 1.

#### Trichoberia

Townsend, 1933

58AAABDE-50D4-50A1-9235-E8319767A3F8

= Trichoberia Townsend, 1933: 439. **Type species**: *Trichoberiarufopilosa* Townsend, 1933 (= *Rhyncomyialanata* Villeneuve, 1920), by original designation.

#### Trichoberia kamita

Lehrer, 2007

1854855F-573B-53AA-BE9B-395398BD1FDC

= Trichoberia kamita Lehrer, 2007: 13. **Type Locality**: South Africa, Natal [KwaZulu-Natal], St. Lucia Park.

##### Distribution

**Afrotropical**: South Africa.

##### Notes

No specimens examined for South Africa, based on Lehrer 2007a. **Illustrations and photography**: male terminalia as in fig. 132 in Lehrer (2011).

#### Trichoberia lanata

(Villeneuve, 1920)

CFE4E541-3321-54EF-B0E1-518E9292CE82

= Rhyncomya lanata Villeneuve, 1920: 162. **Type locality**: Congo Belge [Democratic Republic of Congo].
= Trichoberia rufopilosa Townsend, 1933: 440. **Type locality**: Guinea.

##### Distribution

**Figure 128.**
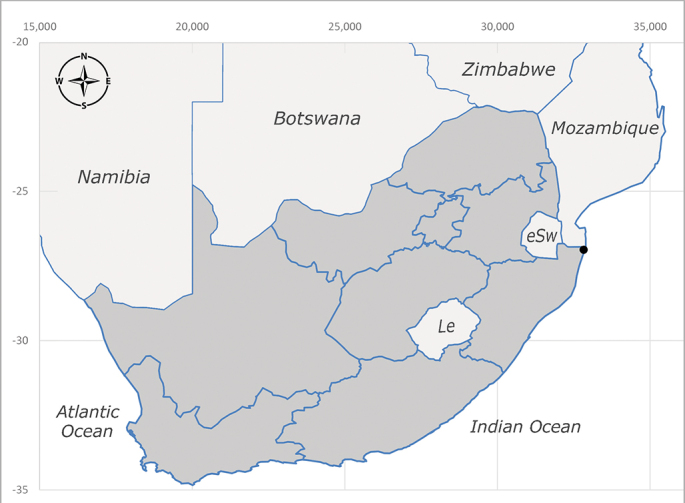
*Trichoberialanata* occurrence map in South Africa, including eSwatini (eSw) and Lesotho (Le); horizontal axis longitude east and vertical axis latitude south.

**Afrotropical**: Democratic Republic of Congo, Equatorial Guinea, Ethiopia, Malawi, South Africa* (Fig. 128), Uganda and Zimbabwe.

##### Notes

**Figure 129.**
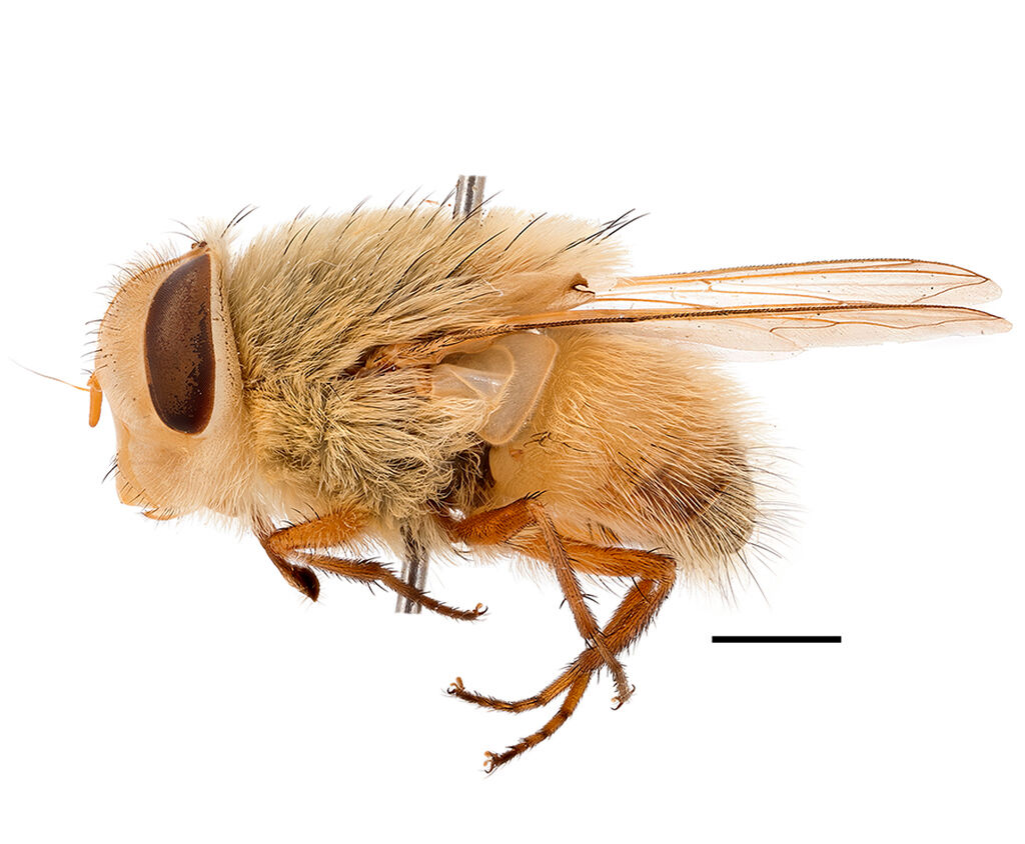
Habitus, left lateral view of *Trichoberialanata* male NMSA DIP 19721 from South Africa; scale bar = 2 mm.

**Preferred environment**: forest and open woodland areas. **Recorded elevations**: no data. **Seasonality**: three males between September and October. **Behaviour and ecology**: unknown. **Life cycle and developmental stages**: unknown. **Collection methods**: unknown. **Illustrations and photographs**: male habitus as in Fig. 129 and figs. 65a, b, d in Zumpt (1958). Male terminalia as in fig. 65c in Zumpt (1958).

**Material examined**: Suppl. material 1.

#### Zumba

Peris, 1951

D9E765BE-6549-5E0D-AAC6-5E3EFC49A471

= Zumba Peris, 1951: 239. T**ype species**: *Zumbarhinoidea* Peris, 1951, by original designation.

#### Zumba antennalis

(Villeneuve, 1929)

2B9CE393-2C1F-5280-83E4-308A27F21ED1

= Rhyncomyia antennalis Villeneuve, 1929: 185. **Type locality**: South West Africa [Namibia]. **Remarks**: LT in SAMC designated by Zumpt 1958.
= Pseudorhyncomyia deserticola Zumpt and Argo, 1978: 35. **Type locality**: South West Africa [Namibia], Gobabeb. **Remarks**: HT in NMSA.

##### Distribution

**Figure 130.**
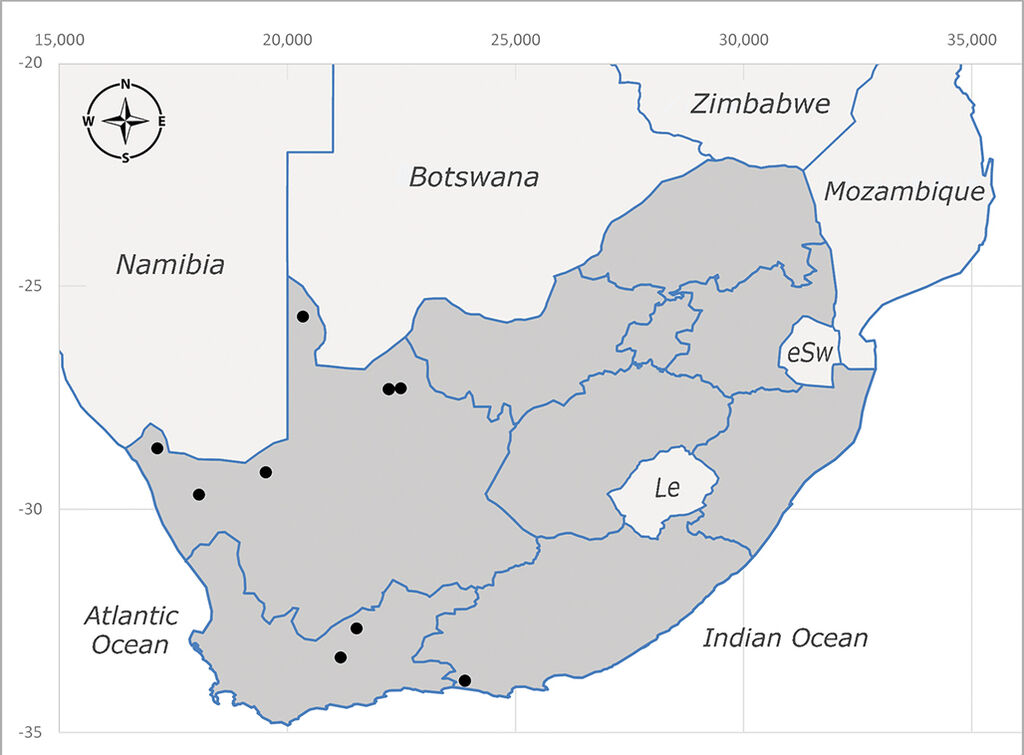
*Zumbaantennalis* occurrence map in South Africa, including eSwatini (eSw) and Lesotho (Le); horizontal axis longitude east and vertical axis latitude south.

**Afrotropical**: Namibia and South Africa (Fig. 130).

##### Notes

**Figure 131.**
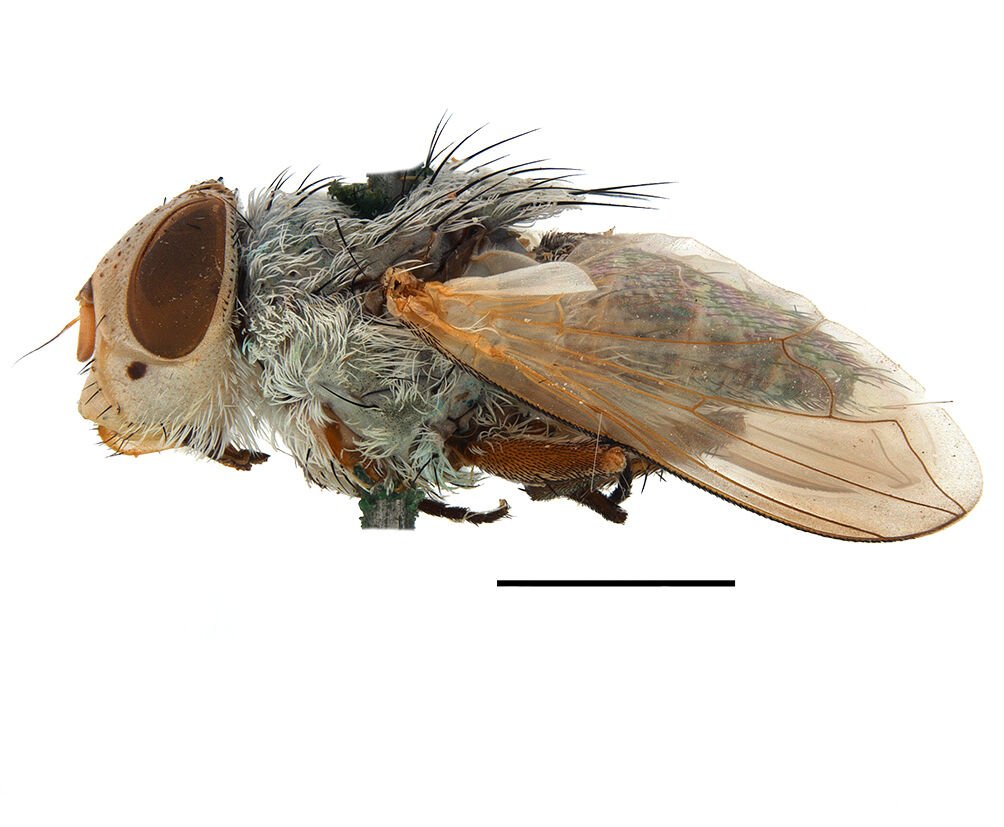
Habitus, left lateral view of *Zumbaantennalis* female SAMC DIP A015172 from Namibia; scale bar = 2 mm.

**Preferred environment**: Gordonia Plains Shrubland, Olifantshoek Thornveld plains, *Senegaliamellifera* on red sand and the Savannah Biome. All the Namibian Biomes, but mainly present in the Desert and Succulent Karoo Biomes showing preferences for the hyper-arid regions (Kurahashi and Kirk-Spriggs 2006). **Recorded elevations**: 762–1245 m a.s.l. **Seasonality**: with low numbers between January and March and September and November, otherwise absent. In Namibia, collected year-round (except June), most abundant from November to February (Kurahashi and Kirk-Spriggs 2006). **Behaviour and ecology**: flower visitor on white flowers of *Stoeberia* Dinter and Schwantes sp., *Deverra* DC sp. (as *Pituranthos* Viv.) and roadside flowers. In Namibia, observed visiting pink flowers of the dwarf shrub *Hermannia* sp. (Kurahashi and Kirk-Spriggs 2006). **Life cycle and developmental stages**: unilarviparous (Thomas-Cabianca et al., unpublished). **Collection methods**: Malaise traps. In Namibia, by hand net and sweeping on flowering bush, UV-light, pitfall, pans (yellow, brown, white and blue) and Malaise traps (Kurahashi and Kirk-Spriggs 2006). **Illustrations and photographs**: female habitus as in Fig. 131. Male habitus as in figs. 29–36 in Rognes (2013). Male terminalia as in fig. 64 in Zumpt (1958) and figs. 37–43 in Rognes (2013).

**Type material examined**: *R.antennalis*: 1 ? // Mafa / Feb. 1923 // S.W. Africa / Mus. Exped. // *Rhyncomyia* / *antennalis* / Typ. Villen. // *Zumba* ? / *antennalis* Vill. / det. Zumpt 56 // LECTO-TYPE / designated / Zumpt 1958 // [SAMC-DIP A011283]; 1 ? // Mafa / Feb. 1923 // S.W. Africa / Mus. Exped. // Type series // [SAMC-DIP A015172]. *P.deserticola*: 1 ? // S.W. Africa: Namib / Desert, Welwitschia / “Forest” nr Gobabeb / 3-X-1967 E. S. Ross / A. R. Stephen // slide no. 6 // *Pseudorhyncomyia* ? / *deserticola* n. sp. / Zumpt & Argo 1976 // HOLOTYPUS // [NMSA-DIP 19837].

**Material examined**: Suppl. materials 1, 2.

#### Zumba rhinoidea

Peris, 1951

4BBA081E-8AD9-5F20-9AE9-800E05A36A04

= Zumba rhinoidea Peris, 1951: 239. **Type locality**: N. Rhodesia [Zambia], Mozabuka.

##### Distribution

**Afrotropical**: South Africa and Zambia.

##### Notes

No specimens examined for South Africa, based on Pont (1980). **Illustrations and photographs**: unavailable.

## Discussion

### Bionomics

This review showed that the life cycles of the Rhiniinae are more diverse than previously thought (Meier et al. 1999). Initially, Rognes (1997) suggested that the group is oviparous. Later, it was considered oviparous and larviparous (Pape and Arnaud 2001). Now, dissections have revealed that females of some species bear fully-developed third instar larvae, suggesting an unilarviparous biology (Table 2) (Thomas-Cabianca et al., unpublished). This uncommon trait, previously reported in the Rhiniinae only once, in the Indian *Stomorhinaprocula* (Walker, 1849) (Arce et al. 2019), increases the range of biological diversity of the family and calls for further studies on life cycles and larval morphology. Previous data on the immature stages of Afrotropical Rhiniinae fauna were available for only five species: *R.apicalis* (Cuthbertson 1938), *R.pruinosa* (Engel and Cuthbertson 1937), *S.vinculatum* (Cuthbertson 1933, Cuthbertson 1935), *S.cribrata* (Erzinçlioglu 1984) and *S.lunata* (Cuthbertson 1933, Cuthbertson 1935, Hall 1947, Greathead 1963).

Data on the ecology and natural history of several rhiniid species also improved. All species of *Pseudorhyncomyia* and *Stegosoma*, several species of *Stomorhina* and a few species of *Rhyncomya* show some level of association with termites (Table 2) and species of *Rhinia* and *Villeneuviella* Austen, 1914 have also been reported in termite nests (Ferrar 1987). This generally scarce evidence suggests that rhiniines behave like parasitoids, predators or scavengers inside the nests (Ferrar 1987), which resembles the biology of some Bengaliidae, the sister group to Rhiniinae (Buenaventura et al. 2020, Yan et al. 2021).

Similarly, three *Rhinia* species (*R.apicalis*, *R.coxendix* and *R.nigricornis*) showed different associations with wasps (specifically *Bembixmelanopa* in Cuthbertson 1938 and *Cercerisyngvei* on material examined), as both adults and larvae were collected inside nests and females were observed attending nests. This suggests an ecological relationship between *Rhinia* and these wasps, the nature of which remains unknown. Four other species (*F.albitarsis*, *R.apicalis*, *R.pruinosa* and *S.cribrata*) showed a relationship with soft, turned or humus-rich soil, as several females were reported ovipositing in soil (Table 2), even introducing their entire bodies into the soil while burying the eggs (Peris 1952a), suggesting that the larvae of these species develop in this substrate. This could explain, in part, why the immature stages of many Rhiniinae species are dificult to find.

The abundance of adult Rhiniinae varies seasonally (Table 1), generally with higher numbers in warmer months (October-April), peaking in December and scarcer in colder months (May-August). Rhiniinae adults are frequently reported as flower visitors. Thirty-one of the species recorded in South Africa have been reported doing so, including species from eight of the country’s 12 genera (Table 2). *Rhyncomya* was the most often reported (10 species out of 23), followed by *Isomyia* (seven species out of 12). They presented associations with indigenous plant species and different crops, suggesting that they are potentially important pollinators in different environments. To our knowledge, no studies on the role of Rhiniinae as pollinators have been conducted.

### Diversity

Seventy-three species of Rhiniinae belonging to twelve genera are now known for South Africa, nine of which are new records (*Cosminaundulata*, *Isomyiacuthbertsoni*, *Rhyncomyabotswanae*, *R.trisis*, *Stomorhinaapta*, *S.malobana*, *Thoraciteskirkspriggsi*, *Th.sarcophagoides* and *Trichoberialanata*). Of the 73 species, 66 were examined (identifications, re-identifications and corroborations) and the other seven were assessed exclusively on literature records.

The 73 species represent almost 50% of the Rhiniinae fauna known for the Afrotropical Region (approx. 150 species sensu Pont 1980). Fourteen species (19.18%) are endemic to South Africa, 50 species (68.49%) are also reported in other Afrotropical countries and nine species (12.33%) also occur in other biogeographical regions. Seven of the latter nine species (*Cosminaaenea*, *C.fuscipennis*, *Rhinianigricornis*, *Rhyncomyacassotis*, *R.tristis*, *Stomorhinachapini* and *S.cribrata*) extend to the southern Palaearctic Region (Rognes 2002, Dawah et al. 2019). The other two, *Rhiniaapicalis* and *S.lunata*, are also present in the Oriental and Palaearctic Regions, with *R.apicalis* even reaching the Pacific islands (including Hawaii) and the Australasian Region (Verves 2003, Hassan et al. 2018). A single record of *S.lunata* was reported for the Nearctic Region in the Bermuda Islands, where it is considered an exotic species (Woodley and Hilburn 1994).

Our review confirms that South Africa has the highest species richness of Rhiniinae, followed by India with 57 species (Nandi 2004, Bharti 2011, Bharti 2014, Bharti and Bunchu 2016), Thailand with 43 (Bunchu et al. 2012) and Vietnam with 41 (Kurahashi and Chowanadisai 2001). In the Afrotropics, Namibia is the only other country with an updated Rhiniinae checklist, with roughly half the richness (39 species) of South Africa (Kurahashi and Kirk-Spriggs 2006). Considering that several specimens remain to be determined and/or corroborated within the collections that we examined,and that around 15 new South African morphospecies need to be described (Thomas-Cabianca et al., unpublished), it is very likely that the diversity of Rhiniinae in South Africa is significantly higher.

At the tribe level, Cosminini is the most diverse in South Africa with 60 species in nine genera, with *Rhyncomya* the largest genus of the subfamily with 27 species that represent 54% of the 50 known Afrotropical *Rhyncomya* species sensu Pont (1980). This is consistent with Namibia, where it is also the most diverse genus (Kurahashi and Kirk-Spriggs 2006). *Isomyia* is the second most diverse genus with 13 species, representing 31.71% of the 41 known *Isomyia* species for the Afrotropical Region (Pont 1980). The other tribe, Rhiniini, is represented by 13 species in three genera, where *Stomorhina* is the most diverse with eight species that represent 61.54% of the 13 species recorded in the Afrotropical Region (including *S.malobana*, but excluding *S.tristriata* (Becker, 1909)) (Pont 1980).

### Sampling

Specimens here studied have been sampled using a variety of techniques, each of them offering different advantages and targets. The most common technique is Malaise trap, followed by hand netting, pan trapping (23 yellow, eight blue, six white, two brown and one orange) and pitfall trapping (Table 3). The Malaise trap is an efficient method for capturing rare and uncommon species (e.g. *Pseudorhyncomyia* spp. and *Trichoberia* spp.). Attraction traps, such as pan traps, are useful for collecting flower-visiting species, behaviour commonly reported for many Rhiniinae, whereas pitfall traps are useful for capturing species with ground-dwelling habits, such as *Cosminagracilis* (Kurahashi and Kirk-Spriggs 2006).

Compared with other African countries, South Africa has had a broad sampling effort as indicated by the almost 3,000 collected specimens. However, sampling has been uneven. KwaZulu-Natal has been extensively sampled and showed the largest number of collected individuals and diversity of species, while the Free State, Northern Cape and North West Provinces have the lowest, showing that more collection effort is necessary in the central areas of the country. This is clearly evident in most of the distribution maps for each species and was very well illustrated in a trapping and mapping study of the common and economically significant calliphorids *Luciliasericata* (Meigen, 1826) and *Luciliacuprina* (Wiedemann, 1830) (Williams et al. 2014). In relation to the South African biomes, the savannah (East, South and West) and grasslands contain the highest abundance and diversity of insects and the Nama-Karoo the lowest (Mucina and Rutherford 2006, Kirk-Spriggs and Stuckenberg 2010). The Nama-Karoo and Savannah Biomes in the Northern-Central area (Northern Cape and North West Provinces) remain poorly explored. Recent expeditions to the area by the SAMC, which resulted in three new records (*R.botswanae*, *Th.kirkspriggsi* and *Th.sarcophagoides*) for South Africa, are clear evidence of this.

### Conclusion

Overall, although knowledge on the Diptera of South Africa is vast, collection expeditions and research have never focused specifically on Rhiniinae. In this context, thorough revisions, based on specimens housed in entomological collections, such as the one presented here, are very useful for gathering information that would otherwise be scattered and lost across different institutions. Our main findings and contributions include nine new records for South Africa, one new combination, one reinstated species and significant information on the life habits and ecology of the group, all of which form a base for productive future expeditions and studies focused on Rhiniinae. In particular, future studies should focus on the taxonomic value of the immature stages, exploring the ecological association of some species of Rhiniinae with termites, ants, wasps and soils rich in organic matter and the phylogeny of the family. Promoting and training local taxonomists on Diptera would be important to increase our knowledge of this complex group.

## Supplementary Material

XML Treatment for Rhiniini

XML Treatment for Fainia

XML Treatment for Fainia albitarsis

XML Treatment for Fainia elongata

XML Treatment for Rhinia

XML Treatment for Rhinia apicalis

XML Treatment for Rhinia coxendix

XML Treatment for Rhinia nigricornis

XML Treatment for Stomorhina

XML Treatment for Stomorhina apta

XML Treatment for Stomorhina armatipes

XML Treatment for Stomorhina chapini

XML Treatment for Stomorhina cribrata

XML Treatment for Stomorhina guttata

XML Treatment for Stomorhina lunata

XML Treatment for Stomorhina malobana

XML Treatment for Stomorhina rugosa

XML Treatment for Cosminini

XML Treatment for Cosmina

XML Treatment for Cosmina aenea

XML Treatment for Cosmina fuscipennis

XML Treatment for Cosmina gracilis

XML Treatment for Cosmina margaritae

XML Treatment for Cosmina thabaniella

XML Treatment for Cosmina undulata

XML Treatment for Eurhyncomyia

XML Treatment for Eurhyncomyia diversicolor

XML Treatment for Eurhyncomyia metzi

XML Treatment for Isomyia

XML Treatment for Isomyia cuthbertsoni

XML Treatment for Isomyia darwini

XML Treatment for Isomyia deserti

XML Treatment for Isomyia distinguenda

XML Treatment for Isomyia dubiosa

XML Treatment for Isomyia eos

XML Treatment for Isomyia innia

XML Treatment for Isomyia longicauda

XML Treatment for Isomyia natalensis

XML Treatment for Isomyia oculosa

XML Treatment for Isomyia pubera

XML Treatment for Isomyia transvaalensis

XML Treatment for Isomyia tristis

XML Treatment for Pseudorhyncomyia

XML Treatment for Pseudorhyncomyia braunsi

XML Treatment for Rhyncomya

XML Treatment for Rhyncomya bicolor

XML Treatment for Rhyncomya botswanae

XML Treatment for Rhyncomya buccalis

XML Treatment for Rhyncomya cassotis

XML Treatment for Rhyncomya currani

XML Treatment for Rhyncomya dasyops

XML Treatment for Rhyncomya depressifrons

XML Treatment for Rhyncomya disclusa

XML Treatment for Rhyncomya discrepans

XML Treatment for Rhyncomya forcipata

XML Treatment for Rhyncomya fovealis

XML Treatment for Rhyncomya hessei

XML Treatment for Rhyncomya inflata

XML Treatment for Rhyncomya interclusa

XML Treatment for Rhyncomya maculata

XML Treatment for Rhyncomya messoria

XML Treatment for Rhyncomya minutalis

XML Treatment for Rhyncomya nana

XML Treatment for Rhyncomya paradoxa

XML Treatment for Rhyncomya paratristis

XML Treatment for Rhyncomya peraequa

XML Treatment for Rhyncomya pruinosa

XML Treatment for Rhyncomya soyauxi

XML Treatment for Rhyncomya stannocuprea

XML Treatment for Rhyncomya trispina

XML Treatment for Rhyncomya tristis

XML Treatment for Rhyncomya viduella

XML Treatment for Stegosoma

XML Treatment for Stegosoma bowdeni

XML Treatment for Stegosoma vinculatum

XML Treatment for Stegosoma wellmani

XML Treatment for Thoracites

XML Treatment for Thoracites cingulatus

XML Treatment for Thoracites kirkspriggsi

XML Treatment for Thoracites petersiana

XML Treatment for Thoracites sarcophagoides

XML Treatment for Trichoberia

XML Treatment for Trichoberia kamita

XML Treatment for Trichoberia lanata

XML Treatment for Zumba

XML Treatment for Zumba antennalis

XML Treatment for Zumba rhinoidea

E1D44406-0B75-54FF-89F3-DCD3E6218941 10.3897/BDJ.10.e72764.suppl1 7538293 Supplementary material 1 Material examined of Rhiniinae (Diptera, Oestroidea) from South Africa Data type georeferences, occurrences and information from specimen labels. File: oo_737008.tsv https://binary.pensoft.net/file/737008 Thomas-Cabianca A, Villet MH, Matínez-Sánchez A, Rojo S

52CE0A35-A2F7-5ECC-B6E2-D84728026D50 10.3897/BDJ.10.e72764.suppl2 Supplementary material 2 Material examined of Rhiniinae (Diptera, Oestroidea) from the Afrotropical Region (excluding South Africa) Data type data from specimen labels. File: oo_737009.tsv https://binary.pensoft.net/file/737009 Thomas-Cabianca A, Villet MH, Martínez-Sánchez A, Rojo S

19FBD76E-FF0E-54CC-AD75-19732E75A852 10.3897/BDJ.10.e72764.suppl3 Supplementary material 3 Georeferences of the localities of South Africa Data type georeferences. Brief description Georeferences of the localities of South Africa, with modification and accurate spelling of South African places recorded in the labels data of the material examined (cited in Appendix I). Abbreviations used in the tables: AFMD: added from museum data; AFOL: added from other label; DL: data provided by the labels; GE: data georeferenced with Google Earth; JLDB: data provided from Jason Londt database; ML: data provided by the museum; N/D: no data; QDGS: quarter degree grid square for South Africa. File: oo_575732.tsv https://binary.pensoft.net/file/575732 Thomas-Cabianca A, Villet MH, Martínez-Sánchez A, Rojo S

## Figures and Tables

**Table 1.** Seasonal abundance of South African Rhiniinae species, based on the material reviewed for this study and with available data. Abbreviations used in the table: J: January; F: February; M: March; A: April; M: May; J: June; J: July; A: August; S: September; O: October; N: November; D: December; T: Total.

**Species**	**J**	**F**	**M**	**A**	**M**	**J**	**J**	**A**	**S**	**O**	**N**	**D**	**T**
*Cosminaaenea*	5	2	14	1	1	3	0	1	5	1	14	5	52
*C.fuscipennis*	7	1	9	0	1	0	2	8	41	64	12	7	152
*C.gracilis*	2	0	2	2	1	0	0	0	18	6	9	3	43
*C.margaritae*	0	0	0	0	0	0	0	0	0	0	1	3	4
*C.undulata*	1	0	0	0	0	0	0	0	0	1	0	1	3
*Eurhyncomyiadiversicolor*	1	4	5	5	1	1	6	2	0	5	3	24	57
*E.metzi*	0	0	0	0	0	0	7	0	1	0	0	0	8
*Fainiaalbitarsis*	13	14	1	2	7	5	7	6	1	4	16	22	98
*F.elongata*	0	0	0	1	0	0	0	0	0	0	0	0	1
*Isomyiacuthbertsoni*	0	0	4	0	0	0	0	0	0	11	2	15	32
*I.darwini*	1	1	0	0	0	0	0	0	0	0	4	1	7
*I.deserti*	2	2	2	0	2	1	0	0	0	0	0	2	11
*I.distinguenda*	3	1	0	1	1	0	0	0	1	0	8	8	23
*I.dubiosa*	0	0	0	0	0	0	0	0	0	3	1	0	4
*I.eos*	0	0	1	0	0	0	0	0	0	0	1	1	3
*I.longicauda*	3	0	1	0	0	1	3	2	1	0	1	1	13
*I.natalensis*	17	48	42	23	0	0	0	5	25	17	35	26	238
*I.oculosa*	0	0	0	0	0	0	0	0	0	0	3	0	3
*I.pubera*	2	1	1	0	0	0	1	0	5	1	1	1	13
*I.transvaalensis*	0	0	0	0	0	0	0	0	0	0	0	1	1
*I.tristis*	39	13	62	43	11	3	2	5	34	21	26	35	294
*Pseudorhyncomyiabraunsi*	0	1	0	2	5	0	0	0	0	1	0	0	9
*Rhinia.apicalis*	5	10	10	32	5	6	8	4	7	9	20	43	159
*Rh.coxendix*	5	2	2	0	1	0	1	0	0	0	0	1	12
*Rh.nigricornis*	3	1	6	6	1	0	0	1	0	0	2	10	30
*Rhyncomyabicolor*	1	1	2	0	0	0	0	0	1	2	0	0	7
*R.botswanae*	0	0	0	0	0	0	0	0	1	0	0	0	1
*R.cassotis*	2	3	10	5	4	7	1	4	3	4	6	12	61
*R.currani*	0	0	0	0	0	0	0	0	4	0	0	0	4
*R.dasyops*	1	0	0	1	1	0	1	0	0	0	1	1	6
*R.depressifrons*	2	2	2	0	0	0	0	0	0	0	2	0	8
*R.disclusa*	0	0	1	0	0	0	0	0	5	4	0	4	14
*R.discrepans*	0	0	0	6	0	0	0	0	1	0	0	0	7
*R.forcipata*	0	10	6	4	3	1	0	0	18	9	32	54	137
*R.hessei*	0	0	1	0	0	0	0	6	14	7	0	0	28
*R.interclusa*	3	1	2	1	2	0	0	0	3	9	6	3	30
*R.maculata*	1	0	0	0	3	0	0	1	3	4	0	3	15
*R.messoria*	2	1	0	1	0	0	0	0	0	2	0	8	14
*R.minutalis*	4	16	6	6	2	0	0	1	9	23	6	5	78
*R.nana*	0	0	4	0	0	1	1	0	0	0	1	3	10
*R.paradoxa*	0	3	1	0	0	1	0	0	0	0	0	1	6
*R.paratristis*	0	0	0	0	0	1	0	0	0	0	10	111	122
*R.peraequa*	0	0	0	0	0	0	0	0	0	0	0	1	1
*R.pruinosa*	0	0	0	1	0	1	2	1	1	0	3	6	15
*R.soyauxi*	14	16	11	26	8	2	7	4	73	8	31	54	254
*R.stannocuprea*	3	1	0	3	0	0	0	2	7	1	1	2	20
*R.trispina*	0	0	0	2	1	0	1	0	0	0	5	0	9
*R.tristis*	0	1	0	0	0	0	0	0	0	0	0	0	1
*R.viduella*	0	0	0	0	0	0	0	0	1	5	0	1	7
*Stegosomabowdeni*	0	1	0	0	0	0	0	0	0	2	0	1	4
*St.vinculatum*	5	7	18	2	3	6	0	0	0	7	6	15	69
*St.wellmani*	2	1	1	2	0	0	0	0	0	0	0	0	6
*Stomorhinaapta*	4	0	0	2	0	0	0	0	2	0	2	2	12
*S.armatipes*	1	1	2	1	0	1	0	0	3	1	2	0	12
*S.chapini*	6	0	1	6	2	1	1	1	0	0	5	4	27
*S.cribrata*	5	0	4	3	0	1	0	1	7	1	3	15	40
*S.guttata*	2	1	3	11	0	3	0	3	17	16	2	6	64
*S.lunata*	40	50	24	11	7	4	2	27	34	26	28	18	271
*S.malobana*	0	0	0	0	0	0	0	0	0	0	1	0	1
*S.rugosa*	13	31	12	10	7	10	8	3	2	8	19	28	151
*Thoracitescingulatus*	0	0	0	0	0	0	0	0	0	0	0	3	3
*Th.kirkspriggsi*	0	0	0	0	0	0	0	0	1	0	0	0	1
*Th.petersiana*	0	0	0	0	1	0	0	0	0	0	20	57	78
*Th.sarcophagoides*	0	0	0	0	0	0	0	0	1	0	0	0	1
*Trichoberialanata*	0	0	0	0	0	0	0	0	0	0	3	0	3
*Zumbaantennalis*	2	3	2	0	0	0	0	0	4	2	2	0	15
Total	222	251	275	222	81	60	61	88	354	285	356	628	2883

**Table 2.** Biological information compiled for the Afrotropical Rhiniinae from the material reviewed (Appendices I and II) and literature. Abbreviations used in the table: **W**: wasp association; **T**: Termite association; **A**: Ant association; **F**: collected or observed on flowers; **S**: females observed laying eggs on soil or immature stages associated with soil; **AB**: collected in animal burrows; **H**: collected or observed hovering; **S**: collected or observed swarming; **FD**: collected on fresh dung of different animals; **O**: Association with Orthoptera oothecae. **Notes**: †oviparous, †† Unilarviparous.

**Species**	**W**	**T**	**A**	**F**	**S**	**AB**	**H**	**Sw**	**FD**	**O**
*Cosminaaenea*										
*C.fuscipennis*	x			x						
*C.gracilis*†				x						
*C.margaritae*†										
*C.undulata*										
*C.thabaniella*										
*Eurhyncomyiadiversicolor*										
*E.metzi*										
*Fainiaalbitarsis*†				x	x		x			
*F.elongata*										
*Isomyiacuthbertsoni*				x						
*I.darwini*				x						
*I.deserti*				x						
*I.distinguenda*										
*I.dubiosa*				x						
*I.eos*										
*I.innnia*										
*I.longicauda*										
*I.natalensis*				x						
*I.oculosa*				x						
*I.pubera*	x									
*I.transvaalensis*										
*I.tristis*	x			x					x	
*Pseudorhyncomyiabraunsi*		x							x	
*Rhiniaapicalis*†	x			x	x	x	x		x	
*Rh.coxendix*†	x									
*Rh.nigricornis*	x			x						
*Rhyncomyabicolor*										
*R.botswana*				x						
*R.buccalis*										
*R.cassotis*				x						
*R.currani*										
*R.dasyops*				x						
*R.depressifrons*										
*R.disclusa*				x						
*R.discrepans*										
*R.forcipata*		x		x						
*R.fovealis*										
*R.hessei*										
*R.inflata*										
*R.interclusa*				x						
*R.maculata*										
*R.messoria*										
*R.minutalis*				x						
*R.nana*				x						
*R.paradoxa*	x									
*R.paratristis*										
*R.peraequa*										
*R.pruinosa*†		x		x	x					
*R.soyauxi*	x	x		x						
*R.stannocuprea*										
*R.trispina*									x	
*R.tristis*										
*R.viduella*				x						
*Stegosomabowdeni*		x								
*St.vinculatum*†		x	x	x		x		x		
*St.wellmani*		x								
*Stomorhinaapta*										
*S.armatipes*†	x	x		x						
*S.chapini*										
*S.cribrata*†	x	x		x	x		x			
*S.guttata*		x		x						
*S.lunata*†		x	x	x						x
*S.rugosa*		x		x					x	
*S.malobana*††				x						
*Thoraciteskirkspriggsi*										
*Th.petersina*										
*Th.cingulatus*										
*Th.sarcophagoides*										
*Trichoberiakamita*										
*T.lanata*										
*Zumbaantennalis*††										
*Z.rhinoides*				x						

**Table 3.** Collection methods reported for the Afrotropical Rhiniinae examined (Appendices I and II). Abbreviations used in the table: sweeping with hand net (**Sb**: Sweeping on bait); Pan traps (**Br**: Brown pan; **Bu**: Blue pan; **Y**: Yellow pan; **W**: White pan; **Mc**: McPhail trap with Nu-Lure); Light trap (**B**: black light; **MV-B**: Mercury Vapour and black light; **UV**: ultraviolet light); Traps baited with organic matter (**R**: Rotten fish; **Ba**: Banana; **Ff**: Hanging traps with fermenting fruit; **Dm**: dead millipedes).

**Species**	**Malaise trap**	**Hand net**	**Pan traps**	**Pitfall trap**	**Light trap**	**Organic baits**
*Cosminaaenea*	x	x				
*C.fuscipennis*	x	x	x (Y, W)			x (Ba)
*C.gracilis*	x	x	x (Y, Bu)	x	x, x (UV)	x (Ff)
*C.margaritae*	x		x (Y, Bu)	x		
*C.undulata*	x					
*C.thabaniella*						
*Eurhyncomyiadiversicolor*	x					
*E.metzi*						
*Fainiaalbitarsis*	x	x		x		
*F.elongata*	x				x	x (R)
*Isomyiacuthbertsoni*	x	x				
*I.darwini*	x			x	x (UV)	
*I.deserti*	x	x	x (Y)			
*I.distinguenda*	X					
*I.dubiosa*	x				x (MV-B)	
*I.eos*	x					
*I.innnia*						
*I.longicauda*	x		x			
*I.natalensis*	x	x	x (Y)		x (MV)	
*I.oculosa*						
*I.pubera*	x					
*I.transvaalensis*						
*I.tristis*	x	x, (Sb)	x (Y, W)	x	x (MV)	
*Pseudorhyncomyiabraunsi*					x (UV)	
*Rhiniaapicalis*†	X, x (UV)	x	x (Y)	x		
*Rh.coxendix*†	x	x				
*Rh.nigricornis*	x					x (R)
*Rhyncomyabicolor*				x		
*R.botswana*	x					
*R.buccalis*						
*R.cassotis*	x	x	x (Y)	x	x (MV)	
*R.currani*						
*R.dasyops*	x			x		
*R.depressifrons*						
*R.disclusa*						
*R.discrepans*	X					
*R.forcipata*	x, x (MV)	X		x	x, x (UV, MV-B)	
*R.fovealis*						
*R.hessei*	x		x (Y)	x	x (UV)	
*R.inflata*						
*R.interclusa*	x		x (Y)			
*R.maculata*						
*R.messoria*	x	X	x (Y)	x		
*R.minutalis*	x	X	x (Y)	x		
*R.nana*	x	X				
*R.paradoxa*						
*R.paratristis*	x					
*R.peraequa*	x		x (Y)	x	x (UV)	
*R.pruinosa*	x	X	x (Y)	x	x, x (UV)	
*R.soyauxi*	x, x (Ex)	X	x (Bu, Y, W, Mc)	x	x, x (UV, MV-B)	
*R.stannocuprea*	x					
*R.trispina*	x	x	x (Bu, Y)	x	x (UV)	x (Ff)
*R.tristis*	x	x	x (Y)	x	x (UV)	
*R.viduella*		x				
*Stegosomabowdeni*						
*St.vinculatum*	x	x		x		
*St.wellmani*	x					
*Stomorhinaapta*	x					
*S.armatipes*	x	x				
*S.chapini*	x					
*S.cribrata*	x					
*S.guttata*	x	x	x (Y)	x		
*S.lunata*	x	x	x (Y)	x	x (B)	
*S.rugosa*	x		x (Bu)		x	
*S.malobana*	x			x		
*Thoraciteskirkspriggsi*	x	x	x (Bu, Y, W)	x (Dm)		
*Th.petersina*	x					
*Th.cingulatus*						
*Th.sarcophagoides*						
*Trichoberiakamita*						
*T.lanata*						
*Zumbaantennalis*	x	x	x (Br, Bu, Y, W)	x	x (UV)	
*Z.rhinoides*						

## References

[B7073260] Arce Bernal J., Clout S., Pat Desirée L., Bharti Meenakshi, Pape Thomas, Marshall Stephen A. (2019). Viviparity and oviparity in termitophilous Rhiniidae (Diptera: Oestroidea) in the Western Ghats, India. Oriental Insects.

[B7073103] Baez M, Santos-Pinto E (1975). Dipteros de Canarias. 1: Calliphoridae. Vieraea.

[B7073199] Bezzi M (1911). Miodarii superiori nell'Africa australe orientale. Bolletino del Laboratorio di Zoologia Generale e Agraria.

[B7073311] Bharti Meenakshi (2011). An updated checklist of blowflies (Diptera: Calliphoridae) from India. Halteres.

[B7073251] Bharti Meenakshi (2014). New record of *Stomorhina Siamensis* Kurahashi et Tumrasvin, 1992 from India, with revised key to Indian species of the genus *Stomorhina* (Diptera: Calliphoridae). Far Eastern Entomologist.

[B7073181] Bharti Meenakshi, Bunchu Nophawan (2016). Three new records of the genus *Isomyia* (Walker, 1859) (Diptera: Calliphoridae) from India, with a revised key to the known Indian species. Japanese Journal of Systematic Entomology.

[B7073279] Buenaventura Eliana, Lloyd Michael W., Perilla López Juan Manuel, González Vanessa L., Thomas-Cabianca Arianna, Dikow Torsten (2020). Protein-encoding ultra conserved elements provide a new phylogenomic perspective of Oestroidea flies (Diptera: Calyptratae). Systematic Entomology.

[B7073053] Bunchu Nophawan, Sukontason Kom, Sanit Sangob, Chidburee Polprecha, Kurahashi Hiromu, Sukontason Kabkaew L. (2012). Occurrence of blow fly species (Diptera: Calliphoridae) in Phitsanulok Province, Northern Thailand.. Tropical Biomedicine.

[B7073044] Cerretti Pierfilippo, Pape Thomas (2012). Phylogenetics and taxonomy of *Ventrops* - The largest genus of Afrotropical Rhinophoridae (Diptera). Invertebrate Systematics.

[B7073129] Cerretti Pierfilippo, Stireman John O., Pape Thomas, O’Hara James E., Marinho Marco A. T., Rognes Knut, Grimaldi David A. (2017). First fossil of an oestroid fly (Diptera: Calyptratae: Oestroidea) and the dating of oestroid divergences. PLOS One.

[B7073073] Cerretti Pierfilippo, Stireman John O., Badano Davide, Gisondi Silvia, Rognes Knut, Giudice Giuseppe Lo, Pape Thomas (2019). Reclustering the cluster flies (Diptera: Oestroidea, Polleniidae). Systematic Entomology.

[B7073224] Cuthbertson A (1933). The habits and life histories of some Diptera in Southern Rhodesia. Proceedings and Transaction of the Rhodesia Scientific Association.

[B7073112] Cuthbertson Alexander (1934). Biological notes on some Diptera in Southern Rhodesia. Proceedings and Transaction of the Rhodesia Scientific Association.

[B7073064] Cuthbertson Alexander (1935). Biological notes on some Diptera in Southern Rhodesia. Occasional Papers of the National Museum of Southern Rhodesia.

[B7073233] Cuthbertson Alexander (1938). Biological notes on some Diptera in Southern Rhodesia. Transactions of the Rhodesia Scientific Association.

[B7073026] Cuthbertson Alexander (1939). Biological notes on some Diptera in Southern Rhodesia. Transactions of the Rhodesia Scientific Association.

[B7073514] Dawah Hassan Ali, Abdullah Mohammed A., Ahmad Syed Kamran (2019). An overview of the Calliphoridae (Diptera) of Saudi Arabia with new records and updated list of species. Journal of the Entomological Research Society.

[B7073163] Dear J. P. (1977). A revision of Australian rhiniinae (diptera: Calliphoridae). Australian Journal of Zoology.

[B7073242] Deeming J C (1996). The Calliphoridae (Diptera: Cyclorrhapha) of Oman. Fauna Saudi Arab.

[B7073141] Deeming J C, van Harten A. (2008). Arthropod fauna of the UAE, Volume 1.

[B7073094] Drees Michael (1998). Ein aktueller Nachweis von *Stomorhinalunata* (F.) (Diptera: Calliphoridae) im mittelern Ruhrtal. Decheniana, Bonn.

[B7073154] El-Hawagry M. S., El-Azab S. A. (2019). Catalog of the Calliphoridae, Rhiniidae, and Sarcophagidae of Egypt (Diptera: Oestroidea). Egyptian Journal of Biological Pest Control.

[B7073085] Engel E O, Cuthbertson Alexander (1937). On the biology of some Rhodesian Diptera, together with descriptions of three species of Asilidae new to science. Transactions of the Rhodesia Scientific Association.

[B7073303] Erzinçlioglu Y. Z. (1984). Studies on the morphology and taxonomy of the immature stages of Calliphoridae, with analysis of phylogenetic relationships within the family, and between it and other groups in the Cyclorrhapha (Diptera).

[B8105488] Evenhuis N. L., Pape T. Systema Dipterorum, Version 3.9. http://diptera.org/.

[B7073121] Ferrar Paul (1987). A guide to the breeding habits and immature stages of DipteraCyclorrhapha. (Part 1, 2).

[B7071339] González-Mora MD, Peris Salvador V (1988). Los Calliphoridae de España: 1 Rhiniinae y Chrysomyinae (Diptera). Eos: Revista Española de Entomología.

[B7070785] Greathead D J (1963). A review of the insect enemies of Acridoidea (Orthoptera). Transactions of the Royal Entomological Society of London.

[B7070983] Hall D G (1947). The blowflies of North America.

[B7070710] Hansen M. D., Olsen K., Jensen T. S. (2015). Nye arter i Danmark – Terrestriske arthropoder og vertebrater.

[B7073216] Hardy D. E. (1981). Insects of Hawaii: A manual of the insects of the Hawaiian Islands, including an enumeration of the species and notes on their origin, distribution, hosts, parasites, etc. Volume 14, Diptera: Cyclorrhapha IV, Series Schizophora Section Calyptratae..

[B7071049] Hassan Muhammad, Bodlah Imran, Bharti Meenakshi, Mahmood Khalid (2018). An updated checklist of blow fly fauna (Diptera: Calliphoridae) of Pakistan with new records for the country. Halteres.

[B7071720] Hulley P E (1983). A survey of the flies breeding in poultry manure, and their potential natural enemies. Journal of the Entomological Society of Southern Africa.

[B7070902] Janion-Scheepers Charlene, Measey John, Braschler Brigitte, Chown Steven L., Coetzee Louise, Colville Jonathan F., Dames Joanna, Davies Andrew B., Davies Sarah J., Davis Adrian L. V., Dippenaar-Schoeman Ansie S., Duffy Grant A., Fourie Driekie, Griffiths Charles, Haddad Charles R., Hamer Michelle, Herbert David G., Hugo-Coetzee Elizabeth A., Jacobs Adriaana, Jacobs Karin, Rensburg Candice Jansen van, Lamani Siviwe, Lotz Leon N., Louw Schalk vd M., Lyle Robin, Malan Antoinette P., Marais Mariette, Neethling Jan Andries, Nxele Thembeka C., Plisko Danuta J., Prendini Lorenzo, Rink Ariella N., Swart Antoinette, Theron Pieter, Truter Mariette, Ueckermann Eddie, Uys Vivienne M., Villet Martin H., Willows-Munro Sandi, Wilson John R. U. (2016). Soil biota in a megadiverse country: Current knowledge and future research directions in South Africa. Pedobiologia - Journal of Soil Ecology.

[B8122857] Kirk-Spriggs AH, Stuckenberg BR, Bickel D, Pape T, Meier R (2010). Diptera Diversity: Status, Challenges and Tools.

[B7070803] Kirk-Spriggs Ashley H, Kirk-spriggs Ashley H, Sinclair Bradley J. (2017). Manual of Afrotropical Diptera.

[B7070991] Kirk-Spriggs Ashley H, Muller Burgert S (2017). Biogeography of Diptera. Manual of Afrotropical Diptera.

[B7071357] Kurahashi Hiromu (1986). Blow flies of medical importance in New Guinea, Bismarck Archipelago and Bougainville Island (Diptera: Calliphoridae) Part I. Genera *Calliphora*, *Tainanina*, *Polleniopsis* and *Melinda*. Esakia.

[B7071460] Kurahashi Hiromu (2001). Four new species of the blow fly genus *Thoracites* Brauer & Bergenstamm (Diptera: Calliphoridae: Rhininae), with a key to all species. Cimbebasia.

[B7071486] Kurahashi Hiromu, Chowanadisai Laojana (2001). Blowflies (Insecta: Diptera: Calliphoridae) from Indochina. Species Diversity.

[B7071266] Kurahashi Hiromu, Kirk-Spriggs Ashley H. (2006). The Calliphoridae of Namibia (Diptera: Oestroidea). Zootaxa.

[B7072758] Kutty Sujatha Narayanan, Pape Thomas, Wiegmann Brian M., Meier Rudolf (2010). Molecular phylogeny of the Calyptratae (Diptera: Cyclorrhapha) with an emphasis on the superfamily Oestroidea and the position of Mystacinobiidae and McAlpine's fly. Systematic Entomology.

[B7071618] Kutty Sujatha Narayanan, Meusemann Karen, Bayless Keith M., Marinho Marco A. T., Pont Adrian C., Zhou Xin, Misof Bernhard, Wiegmann Brian M., Yeates David, Cerretti Pierfilippo, Meier Rudolf, Pape Thomas (2019). Phylogenomic analysis of Calyptratae: resolving the phylogenetic relationships within a major radiation of Diptera. Cladistics.

[B7070875] Lehrer Andy Z (2007). Stomorhiniinae n. sfam. Une nouvelle sous-famille de Calliphoridae (Diptera) et révision de ses taxons. Fragmenta Dipterologica.

[B7072232] Lehrer Andy Z (2007). Deux nouvelles espèces afrotropicales du genre *Trichoberia* Townsend (Diptera, Calliphoridae). Fragmenta Dipterologica.

[B7070974] Lehrer Andy Z (2009). Deux espèces orientales nouvelles du genre *Isomyia* Walker (Diptera, Calliphoridae). Fragmenta Dipterologica.

[B7071755] Lehrer Andy Z (2010). A propos de *Cosminaundulata* Malloch et description d' une espèce affine nouvelle du Kenya. Fragmenta Dipterologica.

[B7071085] Lehrer Andy Z (2011). Recueil de Calliphoridae décrits ou revus (Insecta, Diptera). Entomologica: Annali di Entomologia Generale ed Applicata.

[B7071348] Lewis D. J. (1955). Calliphoridae of medical interest in the Sudan. Bulletin of the Entomological Society of Egypt.

[B7071808] Lutovinovas Erikas, Kinduris Rimvydas (2018). *Stomorhinalunata* (Fabricius, 1805) - new to the fauna of Lithuania (Diptera: Rhiniidae). Bulletin of the Lithuanian Entomological Society.

[B7071375] Malloch J R (1926). Exotic Muscaridae (Diptera).-XVII..

[B7071392] Marinho Marco Antonio Tonus, Wolff Marta, Ramos-Pastrana Yardany, de Azeredo-Espin Ana Maria Lima, Amorim Dalton de Souza (2016). The first phylogenetic study of Mesembrinellidae (Diptera: Oestroidea) based on molecular data: Clades and congruence with morphological characters. Cladistics.

[B7073501] Martínez-Sánchez Anabel, Rognes Knut, Báez Marcos, Carles-Tolrá Hjorth-Andersen Miguel (2002). Catálogo de los Diptera de Esapaña, Portugal y Andorra (Insecta).

[B7071424] Meier Rudolf, Kotrba Marion, Ferrar Paul (1999). Ovoviviparity and viviparity in the Diptera. Biological Reviews.

[B7071000] Mucina L., Rutherford M. C, Mucina L., Rutherford M. C (2006). The vegetation of South Africa, Lesotho and Swaziland.

[B7070816] Nandi B C (2002). Blow flies (Diptera: Calliphoridae) of West Bengal, India with a note on their biodiversity. Records of the Zoological Survey of India.

[B7071575] Nandi B C (2004). Checklist of Calliphoridae (Diptera) of India. Records of the Zoological Survey of India.

[B7071513] Pape Thomas, Arnaud Paul H. (2001). *Bezzimyia* - a genus of native New World rhinophoridae (Insecta, Diptera). Zoologica Scripta.

[B7071635] Pape Thomas, Blagoderov Vladimir, Mostovski Mikhail B (2011). Order Diptera Linnaeus, 1758. In: Zhang, Z.-Q. (Ed.) Animal biodiversity: An outline of higher-level classification and survey of taxonomic richness. Zootaxa.

[B7071653] Peris Salvador V (1951). Descripciones preliminares de nuevos Rhiniini (Dipt. Calliphoridae). Eos: Revista Española de Entomología.

[B7070854] Peris Salvador V (1952). Notas sobre Rhiniini con descripción de nuevas formas. Anales de la Estación Experimental de Aula Dei.

[B7071531] Peris Salvador V (1952). La subfamilia Rhiniinae (Dipt. Calliphoridae). Anales de la Estación Experimental Aula Dei.

[B7071194] Peris Salvador V (1956). Nuevas notas sobre Rhiniini con descripciones de formas nuevas (Dipt., Calliphoridae). Eos: Revista Española de Entomología.

[B7070794] Peris Salvador V (1960). Notas dipterológicas. Graellsia.

[B7071103] Peris Salvador V (1992). A preliminary key to the World genera of the subfamilies Toxotarsinae, Chrysomyinae and Rhiniinae (Diptera, Calliphoridae). Bolletin de la Real Sociedad Española de Historia Natural, Sección Biología.

[B7071593] Phillips Laura, Spear Thomas (2017). Oxford Research Encyclopedia of African History.

[B7071121] Pont Adrian Charles, Crosskey R W (1980). Catalogue of the Diptera of the Afrotropical Region.

[B7071566] Prado e Castro Catarina, Arnaldos María Isabel, García María Dolores (2010). Additions to the Calliphoridae (Diptera) fauna from Portugal, with description of new records. Boletín de la Asociación Española de Entomología.

[B7071606] Prado e Castro Catarina, Szpila Krzysztof, Martínez-Sánchez Anabel, Rego Carla, Silva Isamberto, Serrano Artur R. M., Boieiro Mário (2016). The blowflies of the Madeira archipelago: Species diversity, distribution and identification (Diptera, Calliphoridae s.l.). ZooKeys.

[B7071239] Rickenbach A., Hamon J., Ovazza M. (1962). Calliphoridae de Haute Volta et de Côte d’Ivoire. Bulletin de la Societe Entomologique de France.

[B7070727] Rognes Knut (1991). Blowflies (Diptera, Calliphoridae) of Fennoscandia and Denmark. Fauna Entomologica Scandinavica.

[B7071221] Rognes K. (1997). The Calliphoridae (blowflies) (Diptera: Oestroidea) are not a monophyletic group. Cladistics.

[B7071504] Rognes Knut (2002). Blowflies (Diptera, Calliphoridae) of Israel and adjacent areas, including a new species from Tunisia. Entomologica Scandinavica Supplements.

[B7071702] Rognes Knut, Paterson H E H (2005). *Chrysomyachloropyga* (Wiedemann, 1818) and *C.putoria* (Wiedemann, 1830) (Diptera: Calliphoridae) are two different species. African Entomology.

[B7071293] Rognes Knut (2009). Revision of the Oriental species of the *Bengalia peuhi* species- group (Diptera, Calliphoridae). Zootaxa.

[B7070367] Rognes Knut (2012). Revision of the Afrotropical species of the *Bengaliapeuhi* species-group, including a species reassigned to the *B.spinifemorata* species-group (Diptera, Calliphoridae), with notes on the identity of Ochromyiapetersiana Loew, 1852 (Diptera, Rhiniidae). Zootaxa.

[B7071022] Rognes Knut (2013). A new species in the genus *Pseudorhyncomyia* Peris, 1952 and the identity of *P.deserticola* Zumpt and Argo, 1978 (Diptera, Rhiniidae). Zootaxa.

[B7070965] Séguy E (1949). Les Calliphorides Thelychaetiformes du museum de Paris. Revista Brasileira de Biologia.

[B7071433] Séguy E (1958). Les calliphores africaines du Museum (Dipteres). Bulletin de l'Institut Français d'Afrique Noire. Série A.

[B7071040] Setyaningrum Haris, Al Dhafer Hathal (2014). The Calliphoridae the blow flies (Diptera: Oestroidea) of Kingdom of Saudi Arabia. Egyptian Academic Journal of Biological Sciences. A, Entomology.

[B7070718] Singh Baneshwar, Wells Jeffrey D. (2013). Molecular systematics of the Calliphoridae (Diptera: Oestroidea): Evidence from one mitochondrial and three nuclear genes. Journal of Medical Entomology.

[B7071775] Soós Á, Papp L, Soós Á, Papp L (1986). Catalogue of Palaearctic Diptera.

[B7071495] Šuláková Hana, Barták Miroslav, Vanek Jan (2014). Bzucivkovití (Diptera, Calliphoridae) ceské cásti Krkonoš. Opera Corcontica.

[B7071275] Szpila Krzysztof (2000). Three species of Calliphoridae (Diptera) new to the Polish fauna. Polskie Pismo Entomologiczne.

[B7071662] Ta Huy Thinh (2004). Altitudinal distribution of the Muscidae, Calliphoridae and Sarcophagidae in Vietnam. Part 2: the species at altitude over 1200 m. Tap Chi Sinh Hoc.

[B7071817] Thomas-Cabianca Arianna, Martínez-Sánchez Anabel, Villet Martin H, Rojo Santos (2021). Revision of the Afrotropical genus *Fainia* Zumpt, 1958, with notes on the morphology of Rhiniidae subfamilies (Diptera, Oestroidea). ZooKeys.

[B7071076] van Aartsen Bob (1997). Nieuwe en zeldzame vliegen voor de Nederlandse fauna (Diptera). Nederlandse Faunistische Mededelingen.

[B7071203] Verves Yuriy G. (2005). A catalogue of Oriental Calliphoridae (Diptera). International Journal of Dipterological Research.

[B8089300] Verves Yury G., Khrokalo Lyudmila (2020). Review of the taxa of Calliphoridae and Sarcophagidae (Diptera) studied by late Prof. Andy Z. LEHRER. Priamus Supplement.

[B7071522] Verves Yu G (2003). A preliminary list of species of Calliphoridae and Sarcophagidae (Diptera) of the Republic of Seychelles.. Phelsuma.

[B7071311] Verves Y G (2007). The new faunistic data on Calliphoridae and Sarcophagidae (Diptera) of the Republic of Seychelles. Phelsuma.

[B7071212] Villeneuve J. (1917). Contribution a l'étude des espèces africaines du genre *Thelychaeta* Brauer-Berg.. Annales de la Société Entomologique de France.

[B7071185] Villeneuve J. (1920). Étude de quelques myodaires supérieurs (recueillis par le Dr Brauns, Willowmore, Cap).. Revue Zoologique Africaine.

[B7071442] Williams K. A., Richards C. S., Villet M. H. (2014). Predicting the geographic distribution of *Luciliasericata* and *Luciliacuprina* (Diptera: Calliphoridae) in South Africa. African Invertebrates.

[B7070947] Woodley N E, Hilburn D J (1994). The Diptera of Bermuda. Contributions of the American Entomological Institute.

[B7071094] Yang Shih Tsai, Kurahashi Hiromu, Shiao Shiuh Feng (2014). Keys to the blow flies of Taiwan, with a checklist of recorded species and the description of a new species of *Paradichosia* Senior-White (Diptera, Calliphoridae). ZooKeys.

[B8083863] Yan Liping, Pape Thomas, Meusemann Karen, Kutty Sujatha Narayanan, Meier Rudolf, Bayless Keith M., Zhang Dong (2021). Monophyletic blowflies revealed by phylogenomics. BMC Biology.

[B7073172] Zumpt Fritz (1956). Calliphoridae (Diptera Cyclorrapha) Part I: Calliphorini and Chrysomyiini. Exploratie van het Nationaal Albert Park. Zending G. F. de Witte (1933-1935). [III] +.

[B7070956] Zumpt Fritz (1957). Four new Rhiniini from Madagascar (Diptera: Calliphoridae). Le Naturaliste Malgache.

[B7073017] Zumpt Fritz (1958). Calliphoridae (Diptera Cyclorrapha) Part II: Rhiniini. Exploration du Parc National Albert, Mission G. F. de Witte (1933-1935). [III] +.

[B7071284] Zumpt Fritz (1962). The Calliphoridae of the Madagascan region (Diptera) Part I. Calliphorinae. Verhandlungen der Naturforschenden Gesellschaft in Basel.

[B7071671] Zumpt Fritz (1962). Notes on Calliphoridae from the Ethiopian region, with descriptions of three new species (Diptera). Journal of the Entomological Society of Southern Africa.

[B7071320] Zumpt Fritz (1965). Myiasis in man and animals in the Old World.

[B7070884] Zumpt Fritz, Stimie Marianne (1965). Notes on Calliphoridae of the Ethiopian region, with descriptions of eight new species (Diptera). Annals of the Natal Museum.

[B7071738] Zumpt Fritz (1967). Six new species of Calliphoridae and Sarcophagidae (Diptera, Calyptratae) from the Ethiopian region.

[B7070767] Zumpt Fritz (1972). Notes on Diptera (Sarcophagidae, Calliphoridae) from the Ethiopian geographical region. Zeitschrift Für Angewandte Zoologie.

[B7071680] Zumpt Fritz (1972). *Thoracites* B.B. in the Ethiopian region, with descriptions of two new species (Diptera: Sarcophagidae). Journal of the New York Entomological Society.

[B7071746] Zumpt Fritz (1974). Description of *Rhyncomyabotswanae* n. sp. and notes on *R.peraequa* [sic] Villeneuve and *R.pursina* [sic] Séguy from the Ethiopian geographical region (Diptera: Calliphoridae, Rhiniinae). Zeitschrift für Angewandte Zoologie.

[B7072187] Zumpt Fritz, Argo Dorothee (1978). Description of a new species of *Pseudorhyncomyia* Peris from South West Africa (Diptera: Calliphoridae). Bulletin et Annales de la Société Royale Belge d'Entomologie.

[B7071469] Zumpt Fritz (1981). A new species of *Rhyncomya* Robineau-Desvoidy from South Africa (Diptera: Calliphoridae, Rhiniinae). Annals of the Natal Museum.

